# Supplementary material for: Phosphorylation of phase‐separated p62 bodies by ULK1 activates a redox‐independent stress response
Source: EMBO J. 2023 Jun 12;42(14):e113349. doi: 10.15252/embj.2022113349 (PMC10350833; doi:10.15252/embj.2022113349)
Supplement: Supplementary file 1 — Appendix [file EMBJ-42-e113349-s002.pdf]

Phosphorylation of phase-separated p62 bodies by ULK1 activates a redox-independent stress response

Ryo Ikeda, Daisuke Noshiro, Hideaki Morishita, Shuhei Takada, Shun Kageyama, Yuko Fujioka, Tomoko Funakoshi, Satoko Komatsu-Hirota, Ritsuko Arai, Elena Ryzhii, Manabu Abe, Tomoaki Koga, Hozumi Motohashi, Mitsuyoshi Nakao, Kenji Sakimura, Arata Horii, Satoshi Waguri, Yoshinobu Ichimura, Nobuo N Noda and Masaaki Komatsu

Appendix Contents

Appendix Figure S1	Page 2
Appendix Figure S2	Page 3
Appendix Figure S3	Page 4
Appendix Figure S4	Page 5

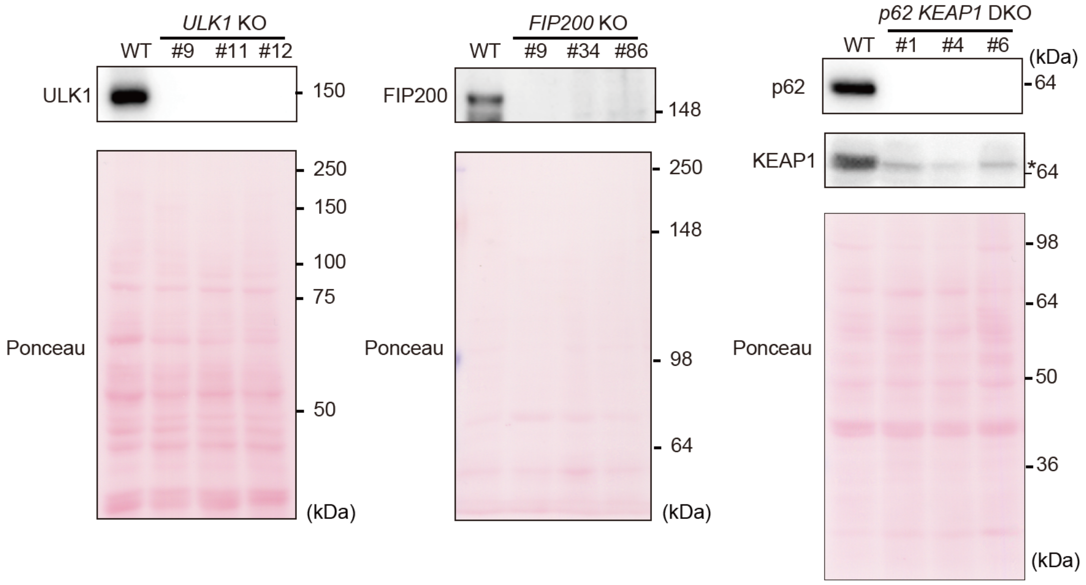

Appendix Figure S1 Generation of *ULK1* knockout , *FIP200* knockout, and *p62/KEAP1* double-knockout cell lines

Immunoblot analysis. The indicated genotype cell lines were lysed, then subjected to SDS-PAGE followed by immunoblot analysis with the indicated antibodies. The asterisk indicates non-specific bands.

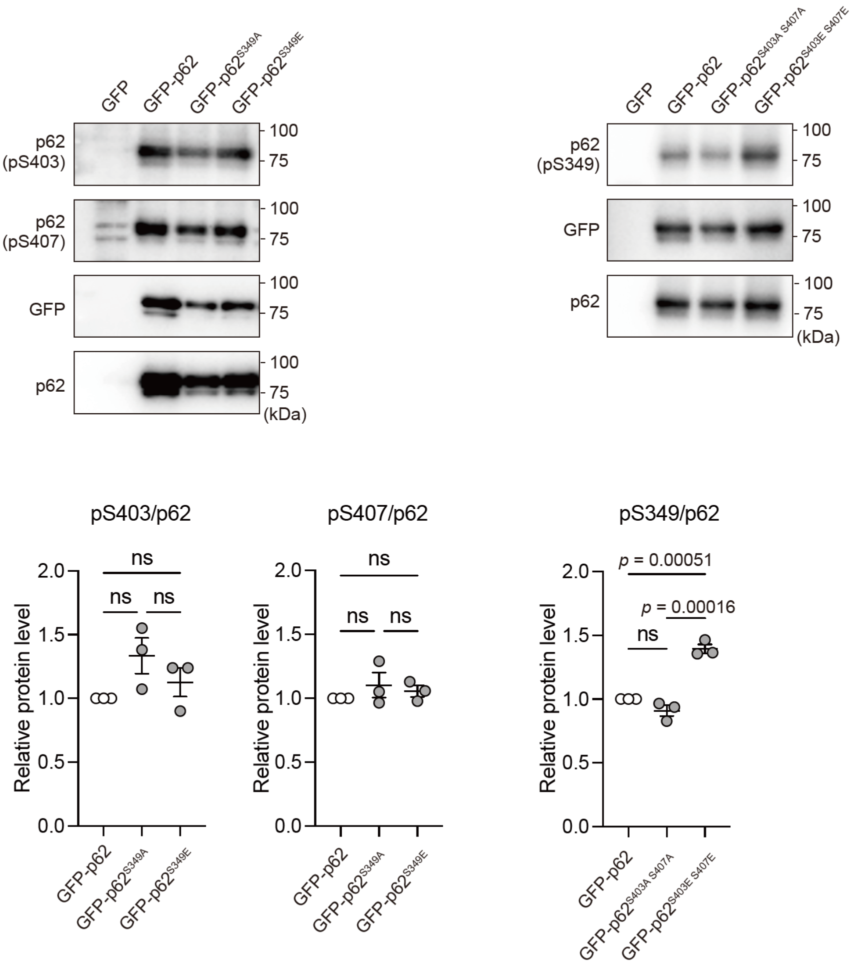

Appendix Figure S2 The phosphorylation of the UBA domain of p62 promotes the phosphorylation of Ser349 of p62

Immunoblot analysis. GFP-tagged Wild-type p62, $p62^{S349E}$, $p62^{S349A}$, $p62^{S403E\ S407E}$ or $p62^{S403A\ S407A}$ mutant was transfected into *p62*-deficient Huh-1 cells. Twenty-four hours after transfection, the cell lysates were subjected to immunoblot analysis with the indicated antibodies. Data shown are representative of three separate experiments. Bar graphs show the results of quantitative densitometric analysis of the Ser349-, Ser403-, or Ser407-phosphorylated p62 form relative to total p62 (n = 3). Data are means ± s.e. Statistical analysis was performed by Šidák’s test after one-way ANOVA.

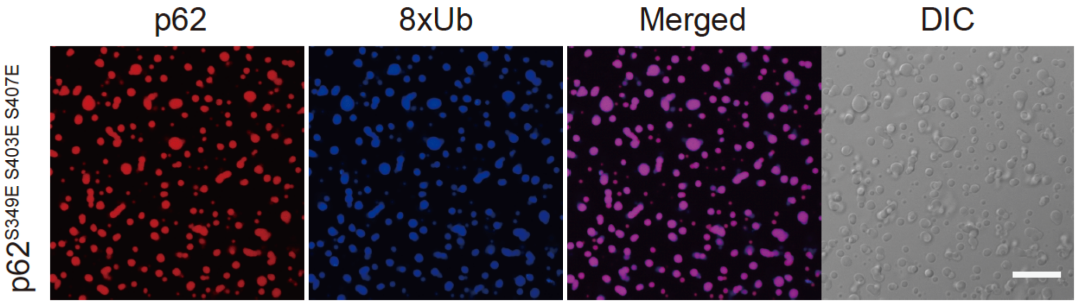

Appendix Figure S3 *In vitro* formation of p62$^{S349E\ S403E\ S407E}$-8xUb condensates

10 µM mCherry-p62$^{S349E\ S403E\ S407E}$ was mixed with 10 µM SNAP-8xUb labeled with SNAP-Surface 649. Scale bar: 20 µm.

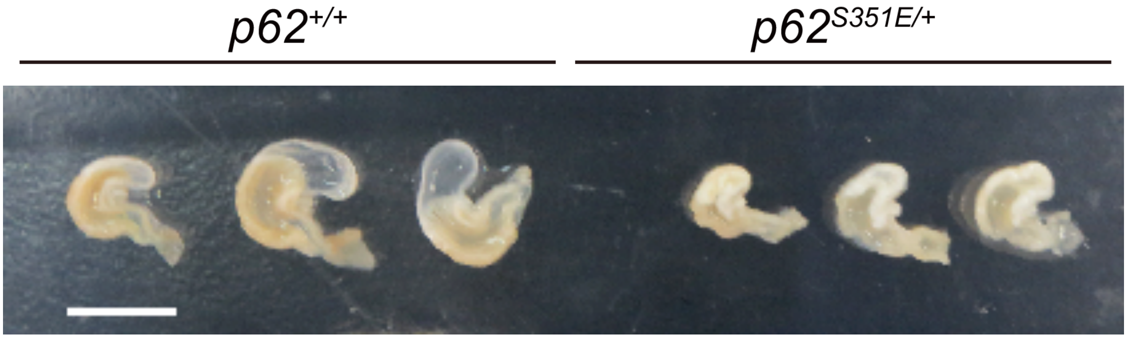

Appendix Figure S4 Gross anatomy of the stomach of *p62*$^{+/+}$ and *p62*$^{S351E/+}$ mice

The forestomach of *p62*^{S351E} heterozygotes was obviously thickened compared with that of wild-type mice. Scale bar: 1cm.
