# Supplementary material for: Phosphorylation of phase‐separated p62 bodies by ULK1 activates a redox‐independent stress response
Source: EMBO J. 2023 Jun 12;42(14):e113349. doi: 10.15252/embj.2022113349 (PMC10350833; doi:10.15252/embj.2022113349)
Supplement: Supplementary file 2 — Expanded View Figures PDF [file EMBJ-42-e113349-s006.pdf]

Expanded View Figures

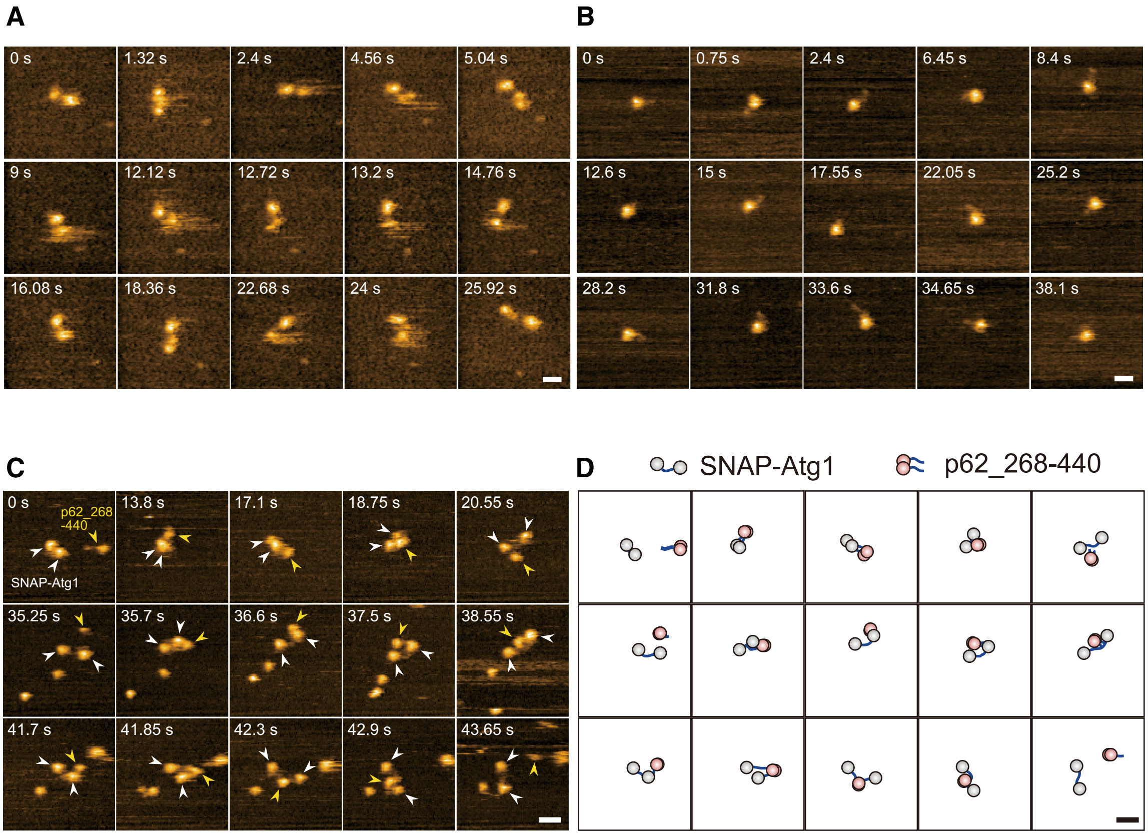

Figure EV1. HS-AFM observation of SNAP-ULK1 and p62 (268–440 aa), and complex of SNAP-Atg1/p62 (268–440 aa).

A, B Successive HS-AFM images of SNAP-ULK1 (A) and p62_268–440 (B). Height scale: 0–4.4 nm (A), 0–3.4 nm (B); scale bar: 20 nm (A, B).

C Successive HS-AFM images of p62_268–440 with SNAP-Atg1. Height scale: 0–3.6 nm; scale bar: 30 nm.

D Schematics showing the molecular characteristics determined by HS-AFM. Gray spheres, globular domains consisting of N-terminal KD and C-terminal MIT of Atg1; pink spheres, globular domains consisting of C-terminal UBA domain of p62; blue thick solid lines, IDRs.

Source data are available online for this figure.

© 2023 The Authors

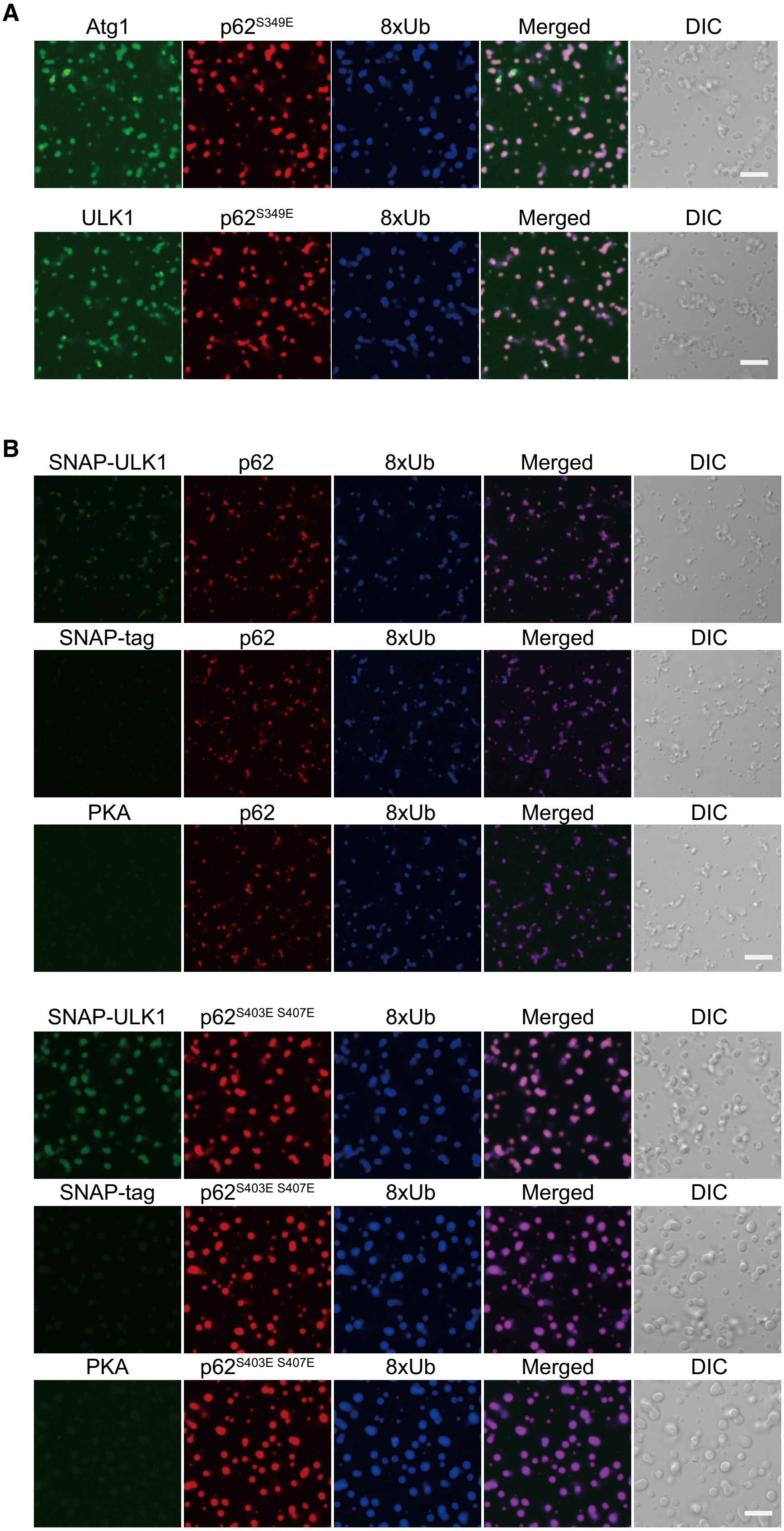

Figure EV2. Specific localization of Atg1 and ULK1 on p62 condensates.

A *In vitro* LLPS assay with p62^{S349E}, 8xUb, and Atg1 or ULK1. 10 μM mCherry-p62^{S349E} and 10 μM 8xUb labeled with Alexa Fluor 649 were mixed with 0.2 μM SNAP-Atg1, or SNAP-ULK1 labeled with Alexa Fluor 488 and observed by fluorescence microscopy. Scale bars: 10 μm.

B Higher enrichment of ULK1 in p62/p62$^{S403E\ S407E}$ condensates compared to SNAP-tag and protein kinase A (PKA). 10 μM mCherry-p62 wild-type/mCherry-p62$^{S403E\ S407E}$, 10 μM SNAP-8xUb labeled with Alexa Fluor 649 and 0.2 μM SNAP-ULK1/SNAP-tag/PKA labeled with Alexa Fluor 488 were mixed and observed by fluorescence microscopy. All sets of images were obtained with the same laser power setting. Scale bars: 10 μm.

Source data are available online for this figure.

 © 2023 The Authors

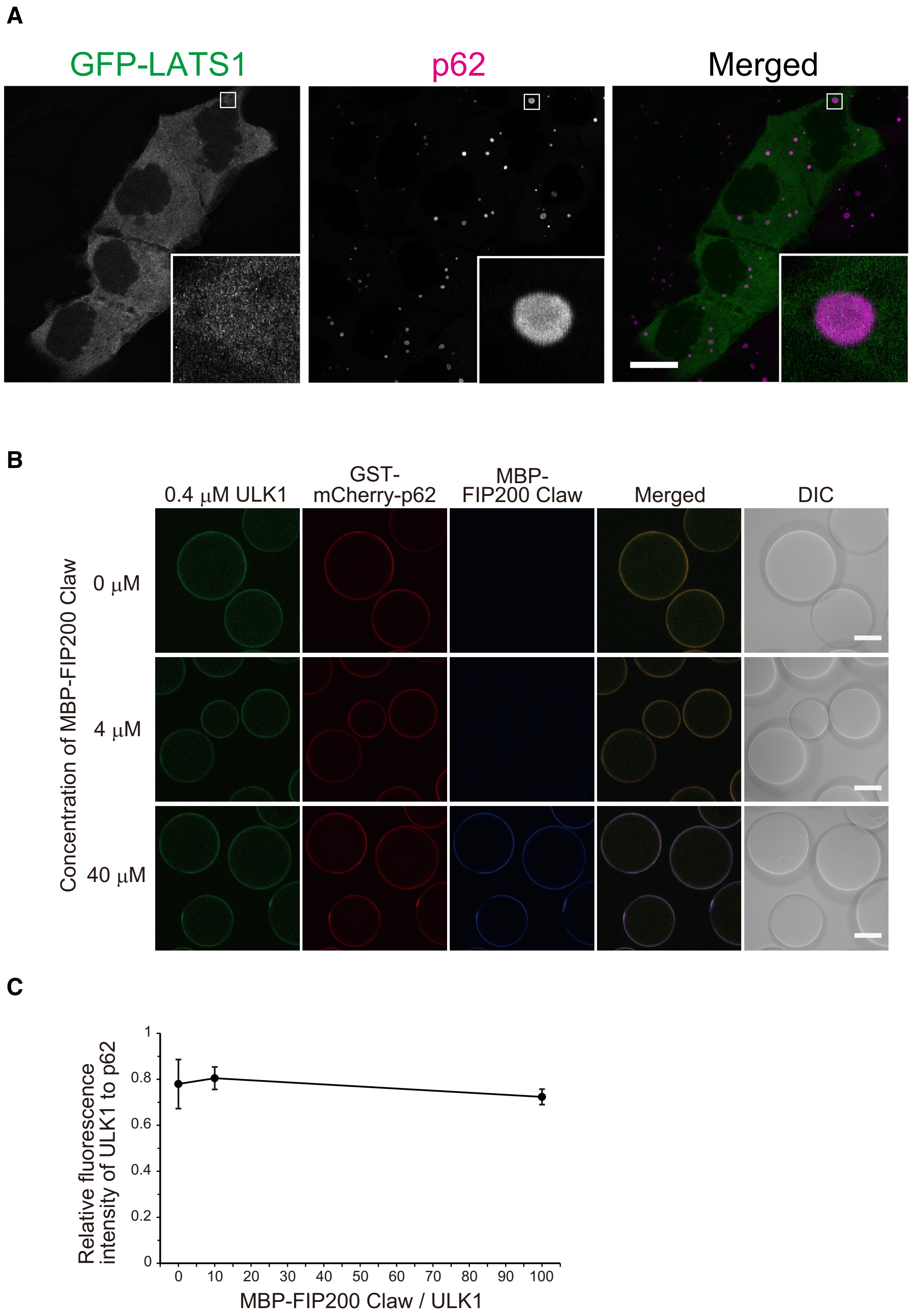

Figure EV3.

© 2023 The Authors

Figure EV3. GFP-tagged LATS does not localize on p62 bodies.

A Immunofluorescence microscopy. Huh-1 cells expressing GFP-LATS1 were immunostained with anti-p62 antibody. Each inset is a magnified image of the boxed region. Scale bar: 20 μm.

B *In vitro* LLPS assay. 0.4 μM SNAP-tagged ULK1 labeled with Alexa Fluor 488 and 0–40 μM MBP-tagged FIP200 Claw domain (aa 1490–1594) labeled with Alexa Fluor 647 C2 maleimide was co-incubated with GST-accept beads coated with GST-tagged mCherry-p62. All sets of images of the beads were obtained by fluorescence microscopy with the same laser power setting. Scale bars: 40 μm.

C Relative fluorescence intensities of ULK1 (Alexa Fluor 488) to p62 (mCherry) were plotted against the molar ratio of MBP-FIP200 Claw to ULK1. Fluorescence intensities of ULK1 and p62 on the beads were measured and analyzed by Fiji. Average values and ± s.d. are shown ($n = 3$).

Source data are available online for this figure.

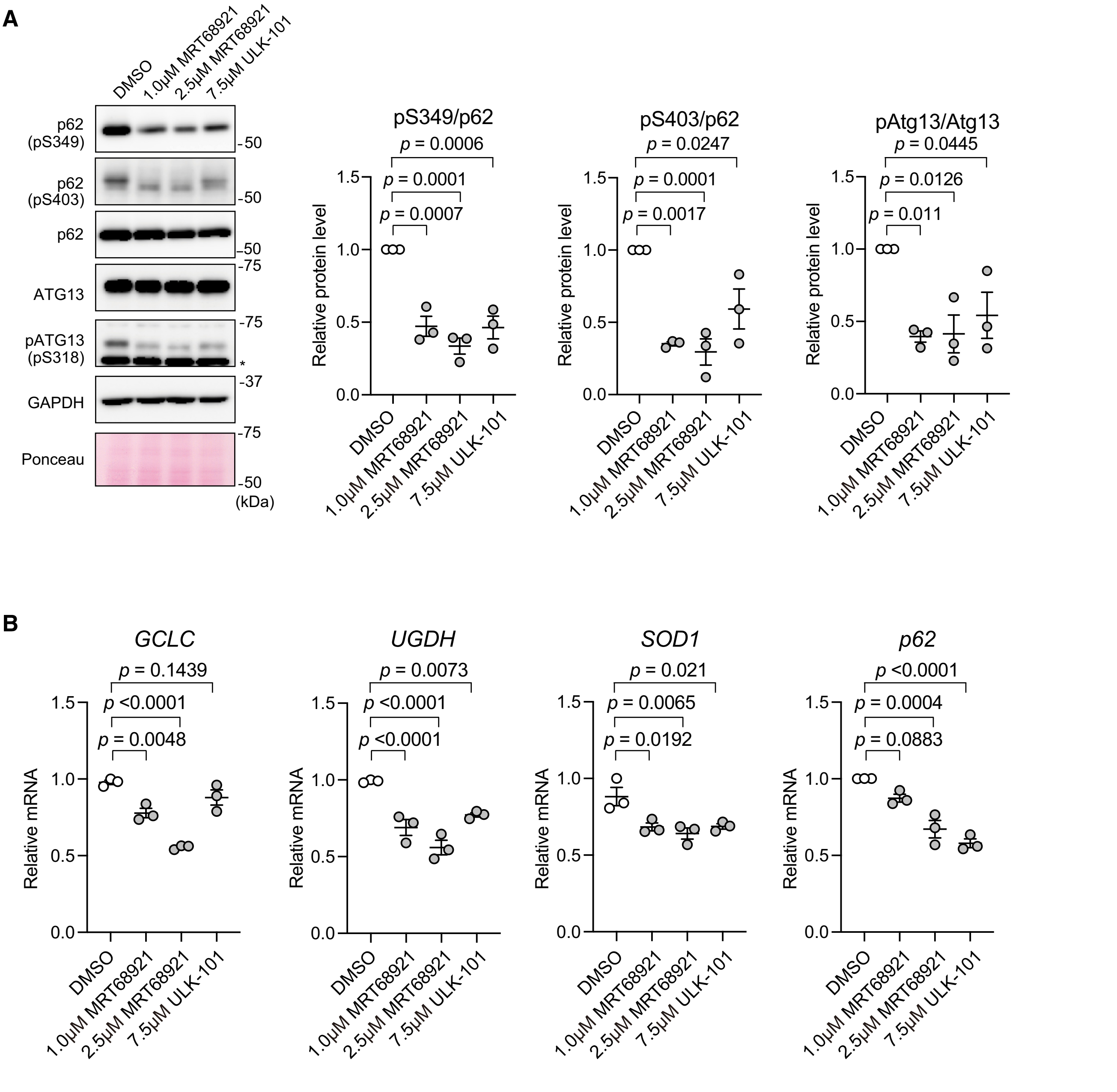

Figure EV4.

© 2023 The Authors

Figure EV4. Effects of ULK1, the ULK2 inhibitor MRT68291, and ULK-101 on p62 phosphorylation.

A Immunoblot analysis. Huh-1 cells were treated with 1 μM MRT68921 or 7.5 μM ULK-101 for 6 h, and the cytosolic and nuclear fractions were subjected to immunoblot analysis with the indicated antibodies. The asterisk indicates non-specific bands. Data shown are representative of three separate experiments. Bar graphs show the results of quantitative densitometric analysis of S349- or S403-phosphorylated p62 forms relative to total p62 (n = 3), and of S318-phosphorylated ATG13 relative to total ATG13 (n = 3). Data are means ± s.e. Statistical analysis was performed by Welch's t-test.

B Gene expression of NRF2 targets. Total RNAs were prepared from Huh-1 cells treated as shown in a. Values were normalized against the amount of mRNA in non-treated Huh-1 cells. qRT-PCR analyses of each biological sample were performed as technical replicates. Statistical analysis was performed by two-sided Welch's t-test.

Source data are available online for this figure.

Figure EV5. Analysis of mice lacking p62 phosphorylation at S351.

A External appearance of $p62^{+/+}$, $p62^{S351A/+}$, and $p62^{S351A/S351A}$ mice at postnatal day (P) 19.

B, C Body weight (g) (B) and liver weight (% of body weight) (C) of $p62^{+/+}$ (n = 3), $p62^{S351A/+}$ (n = 6), and $p62^{S351A/S351A}$ mice (n = 3) at P19. Data are means ± s.e. Statistical analysis was performed by Tukey's test after one-way ANOVA.

D Gene expression of NRF2 targets. Total RNAs were prepared from mouse livers of $p62^{+/+}$ (n = 3), $p62^{S351A/+}$ (n = 6), and $p62^{S351A/S351A}$ mice (n = 3) at P19. Data are means ± s.e. Statistical analysis was performed by Tukey's test after one-way ANOVA.

E Immunoblot analysis of $p62^{+/+}$ (n = 3), $p62^{S351A/+}$ (n = 6), and $p62^{S351A/S351A}$ mice (n = 3) at P19. Liver homogenates were subjected to immunoblot analysis with the indicated antibodies. Bar graphs show the results of quantitative densitometric analysis. Data are means ± s.e. Statistical analysis was performed by Tukey's test after one-way ANOVA. Note that both faster and slower migrating bands in the KEAP1 blot represent translational products from the *Keap1* gene.

F Hematoxylin and eosin (HE) staining of livers from $p62^{+/+}$, $p62^{S351A/+}$, and $p62^{S351A/S351A}$ mice at P19. Scale bars, 100 μm (low magnification panels), and 10 μm (high magnification panels).

G Serum levels of aspartate aminotransferase (AST), alanine aminotransferase (ALT), glucose, total cholesterol, blood urea nitrogen (BUN), and creatinine from $p62^{+/+}$ (n = 3), $p62^{S351A/+}$ (n = 6), and $p62^{S351A/S351A}$ mice (n = 3) at P19 were measured. IU/l, international units/liter. Data are means ± s.e. Statistical analysis was performed by Tukey's test after one-way ANOVA.

© 2023 The Authors

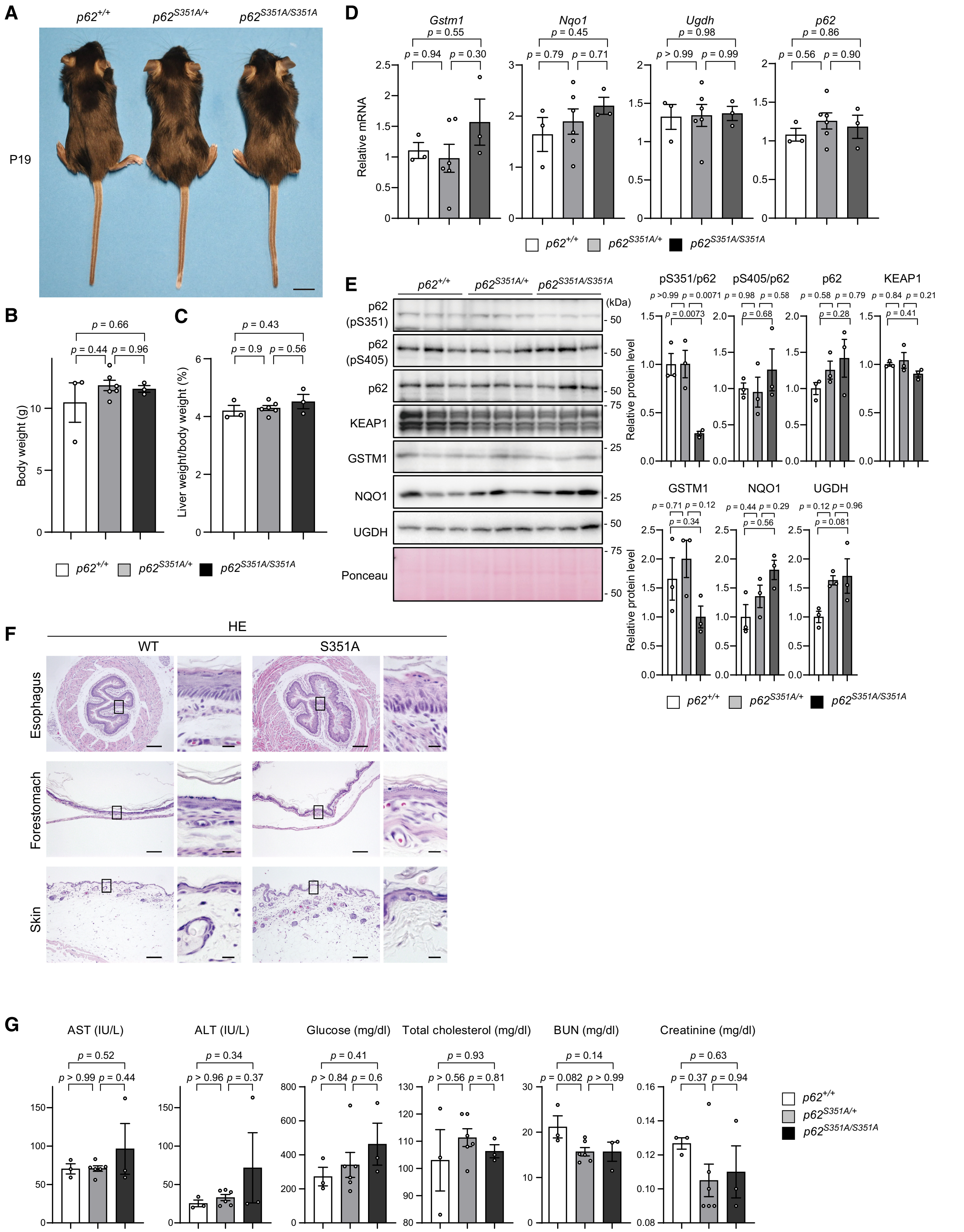
A
p62+/+
p62S351A/+
p62S351A/S351A
P19
B
Body weight (g)
C
Liver weight/body weight (%)
D
Gstm1
Nqo1
Ugdh
p62
Relative mRNA
E
p62 (pS351)
p62 (pS405)
p62
KEAP1
GSTM1
NQO1
UGDH
Ponceau
(kDa)
pS351/p62
pS405/p62
KEAP1
GSTM1
NQO1
UGDH
Relative protein level
F
HE
WT
S351A
Esophagus
Forestomach
Skin
G
AST (IU/L)
ALT (IU/L)
Glucose (mg/dl)
Total cholesterol (mg/dl)
BUN (mg/dl)
Creatinine (mg/dl)

Figure EV5.
