# Supplementary material for: Phosphorylation of phase‐separated p62 bodies by ULK1 activates a redox‐independent stress response
Source: EMBO J. 2023 Jun 12;42(14):e113349. doi: 10.15252/embj.2022113349 (PMC10350833; doi:10.15252/embj.2022113349)
Supplement: Supplementary file 3 — Movie EV1 [file EMBJ-42-e113349-s020.zip › EMBOJ-2022-113349_Movie EV1/Movie EV1_Legend.docx]

Movie EV1

HS-AFM movie of SNAP-ULK1. The images were acquired at 8.33 fps. Height scale: 0–4.5 nm. Scale bar: 20 nm.
