# Supplementary material for: Phosphorylation of phase‐separated p62 bodies by ULK1 activates a redox‐independent stress response
Source: EMBO J. 2023 Jun 12;42(14):e113349. doi: 10.15252/embj.2022113349 (PMC10350833; doi:10.15252/embj.2022113349)
Supplement: Supplementary file 4 — Movie EV2 [file EMBJ-42-e113349-s018.zip › EMBOJ-2022-113349_Movie EV2/Movie EV2_Legend.docx]

Movie EV2

HS-AFM movie of p62_268–440. The images were acquired at 6.67 fps. Height scale: 0–3.5 nm. Scale bar: 20 nm.
