# Supplementary material for: Phosphorylation of phase‐separated p62 bodies by ULK1 activates a redox‐independent stress response
Source: EMBO J. 2023 Jun 12;42(14):e113349. doi: 10.15252/embj.2022113349 (PMC10350833; doi:10.15252/embj.2022113349)
Supplement: Supplementary file 5 — Movie EV3 [file EMBJ-42-e113349-s010.zip › EMBOJ-2022-113349_Movie EV3/Movie EV3_Legend.docx]

Movie EV3

HS-AFM movie of p62_268–440 with SNAP-ULK1. The images were acquired at 8.33 fps. Height scale: 0–4 nm. Scale bar: 20 nm.
