# Supplementary material for: Phosphorylation of phase‐separated p62 bodies by ULK1 activates a redox‐independent stress response
Source: EMBO J. 2023 Jun 12;42(14):e113349. doi: 10.15252/embj.2022113349 (PMC10350833; doi:10.15252/embj.2022113349)
Supplement: Supplementary file 7 — Movie EV5 [file EMBJ-42-e113349-s004.zip › EMBOJ-2022-113349_Movie EV5/Movie EV5_Legend.docx]

Movie EV5

Representative time-lapse image of mCherry-KEAP1 after photobleaching of whole GFP-p62S349E body (Scale bar: 2 µm).
