# Supplementary material for: Phosphorylation of phase‐separated p62 bodies by ULK1 activates a redox‐independent stress response
Source: EMBO J. 2023 Jun 12;42(14):e113349. doi: 10.15252/embj.2022113349 (PMC10350833; doi:10.15252/embj.2022113349)
Supplement: Supplementary file 9 — Movie EV7 [file EMBJ-42-e113349-s013.zip › EMBOJ-2022-113349_Movie EV7/Movie EV7_Legend.docx]

Movie EV7

Representative time-lapse image showing fluorescence loss of mCherry-KEAP1 in GFP-p62S349E bodies after photobleaching over a large area of cells (Scale bar: 20 µm).
