# Supplementary material for: Phosphorylation of phase‐separated p62 bodies by ULK1 activates a redox‐independent stress response
Source: EMBO J. 2023 Jun 12;42(14):e113349. doi: 10.15252/embj.2022113349 (PMC10350833; doi:10.15252/embj.2022113349)
Supplement: Supplementary file 11 — Movie EV9 [file EMBJ-42-e113349-s012.zip › EMBOJ-2022-113349_Movie EV9/Movie EV9_Legend.docx]

Movie EV9

Representative time-lapse image of mCherry-KEAP1 after photobleaching of the central portion of p62S349E body (Scale bar: 2 µm).
