# Supplementary figures and images for: Phosphorylation of phase‐separated p62 bodies by ULK1 activates a redox‐independent stress response (part 2 of 3)
Source: EMBO J. 2023 Jun 12;42(14):e113349. doi: 10.15252/embj.2022113349 (PMC10350833; doi:10.15252/embj.2022113349)

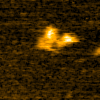

Supplement: Supplementary file 15 — Source Data for Figure 1 [file EMBJ-42-e113349-s003.zip › EMBOJ-2022-113349_SourceDataForFigure 1/1D/1D (104).bmp]

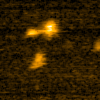

Supplement: Supplementary file 15 — Source Data for Figure 1 [file EMBJ-42-e113349-s003.zip › EMBOJ-2022-113349_SourceDataForFigure 1/1D/1D (15).bmp]

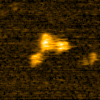

Supplement: Supplementary file 15 — Source Data for Figure 1 [file EMBJ-42-e113349-s003.zip › EMBOJ-2022-113349_SourceDataForFigure 1/1D/1D (148).bmp]

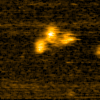

Supplement: Supplementary file 15 — Source Data for Figure 1 [file EMBJ-42-e113349-s003.zip › EMBOJ-2022-113349_SourceDataForFigure 1/1D/1D (113).bmp]

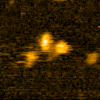

Supplement: Supplementary file 15 — Source Data for Figure 1 [file EMBJ-42-e113349-s003.zip › EMBOJ-2022-113349_SourceDataForFigure 1/1D/1D (144).bmp]

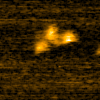

Supplement: Supplementary file 15 — Source Data for Figure 1 [file EMBJ-42-e113349-s003.zip › EMBOJ-2022-113349_SourceDataForFigure 1/1D/1D (118).bmp]

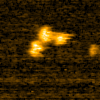

Supplement: Supplementary file 15 — Source Data for Figure 1 [file EMBJ-42-e113349-s003.zip › EMBOJ-2022-113349_SourceDataForFigure 1/1D/1D (132).bmp]

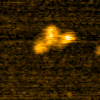

Supplement: Supplementary file 15 — Source Data for Figure 1 [file EMBJ-42-e113349-s003.zip › EMBOJ-2022-113349_SourceDataForFigure 1/1D/1D (109).bmp]

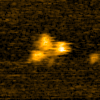

Supplement: Supplementary file 15 — Source Data for Figure 1 [file EMBJ-42-e113349-s003.zip › EMBOJ-2022-113349_SourceDataForFigure 1/1D/1D (134).bmp]

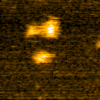

Supplement: Supplementary file 15 — Source Data for Figure 1 [file EMBJ-42-e113349-s003.zip › EMBOJ-2022-113349_SourceDataForFigure 1/1D/1D (19).bmp]

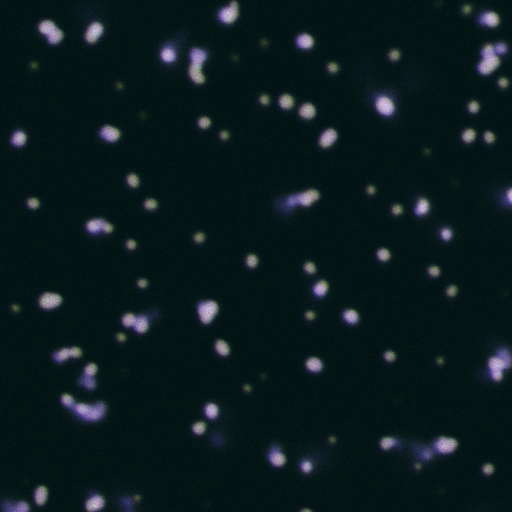

Supplement: Supplementary file 16 — Source Data for Figure 2 [file EMBJ-42-e113349-s016.zip › EMBOJ-2022-113349_SourceDataForFigure 2/2B/2B_Atg1-p62WT (4).tif]

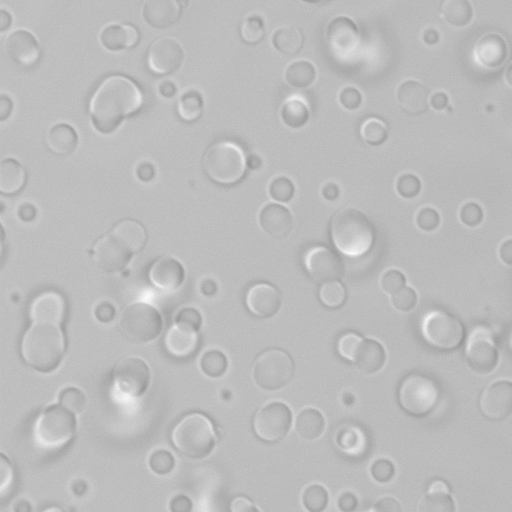

Supplement: Supplementary file 16 — Source Data for Figure 2 [file EMBJ-42-e113349-s016.zip › EMBOJ-2022-113349_SourceDataForFigure 2/2B/2B_ULK1-p62S403E S407E (5).tif]

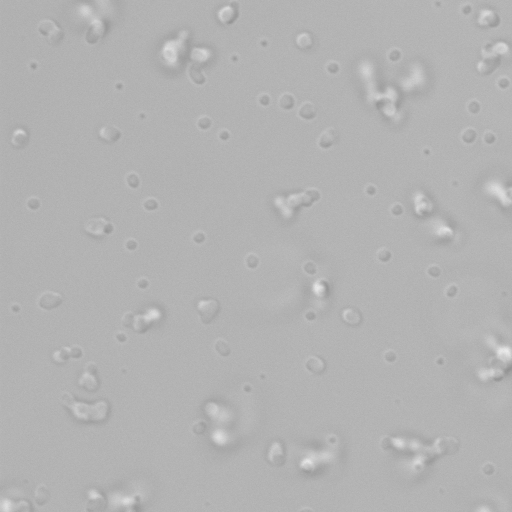

Supplement: Supplementary file 16 — Source Data for Figure 2 [file EMBJ-42-e113349-s016.zip › EMBOJ-2022-113349_SourceDataForFigure 2/2B/2B_Atg1-p62WT (5).tif]

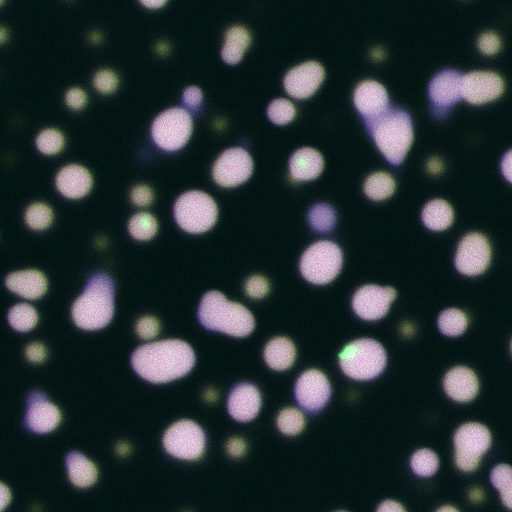

Supplement: Supplementary file 16 — Source Data for Figure 2 [file EMBJ-42-e113349-s016.zip › EMBOJ-2022-113349_SourceDataForFigure 2/2B/2B_Atg1-p62S403E S407E (4).tif]

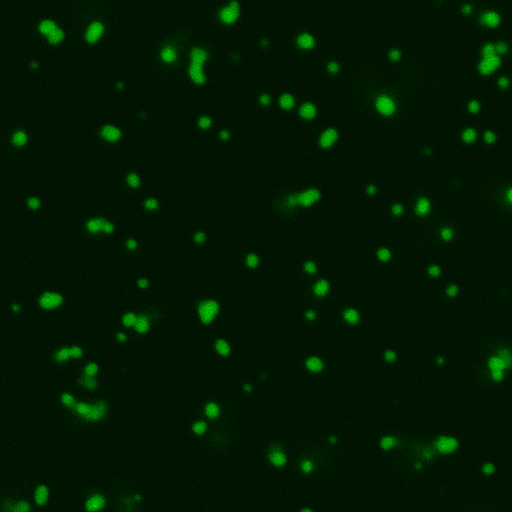

Supplement: Supplementary file 16 — Source Data for Figure 2 [file EMBJ-42-e113349-s016.zip › EMBOJ-2022-113349_SourceDataForFigure 2/2B/2B_Atg1-p62WT (1).tif]

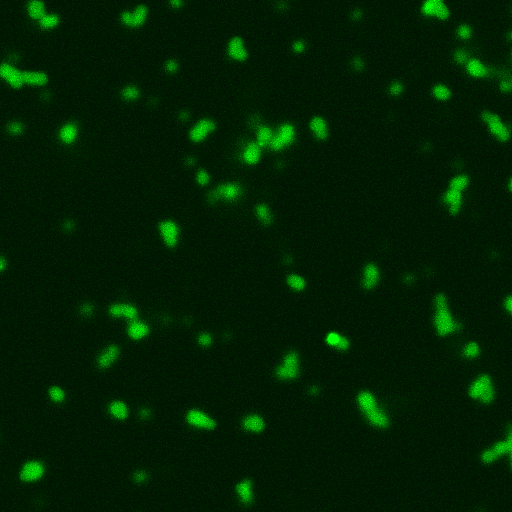

Supplement: Supplementary file 16 — Source Data for Figure 2 [file EMBJ-42-e113349-s016.zip › EMBOJ-2022-113349_SourceDataForFigure 2/2B/2B_ULK1-p62WT (1).tif]

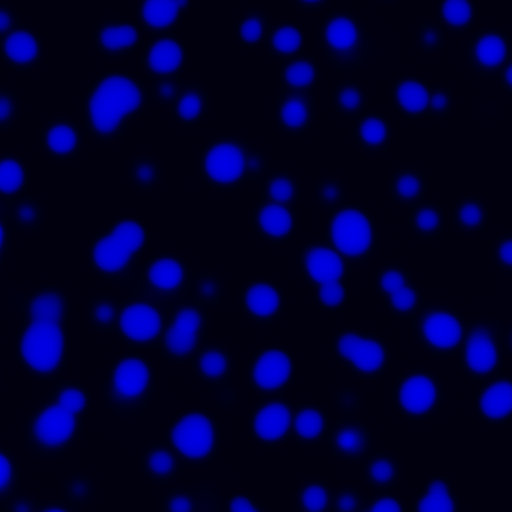

Supplement: Supplementary file 16 — Source Data for Figure 2 [file EMBJ-42-e113349-s016.zip › EMBOJ-2022-113349_SourceDataForFigure 2/2B/2B_ULK1-p62S403E S407E (3).tif]

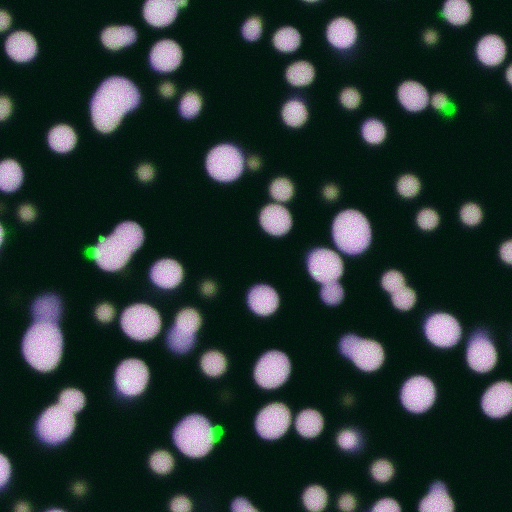

Supplement: Supplementary file 16 — Source Data for Figure 2 [file EMBJ-42-e113349-s016.zip › EMBOJ-2022-113349_SourceDataForFigure 2/2B/2B_ULK1-p62S403E S407E (4).tif]

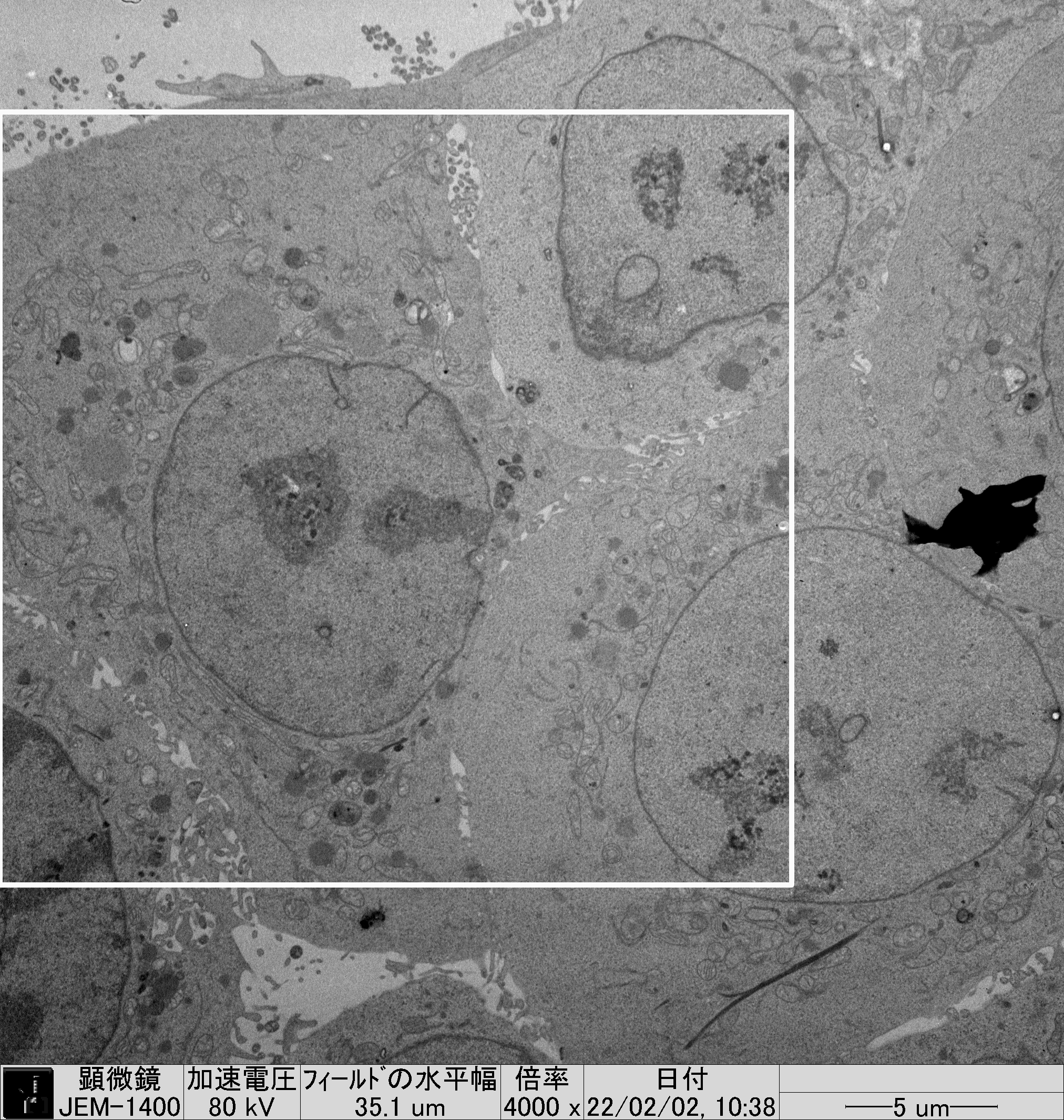

Supplement: Supplementary file 16 — Source Data for Figure 2 [file EMBJ-42-e113349-s016.zip › EMBOJ-2022-113349_SourceDataForFigure 2/2E/EM.tif]

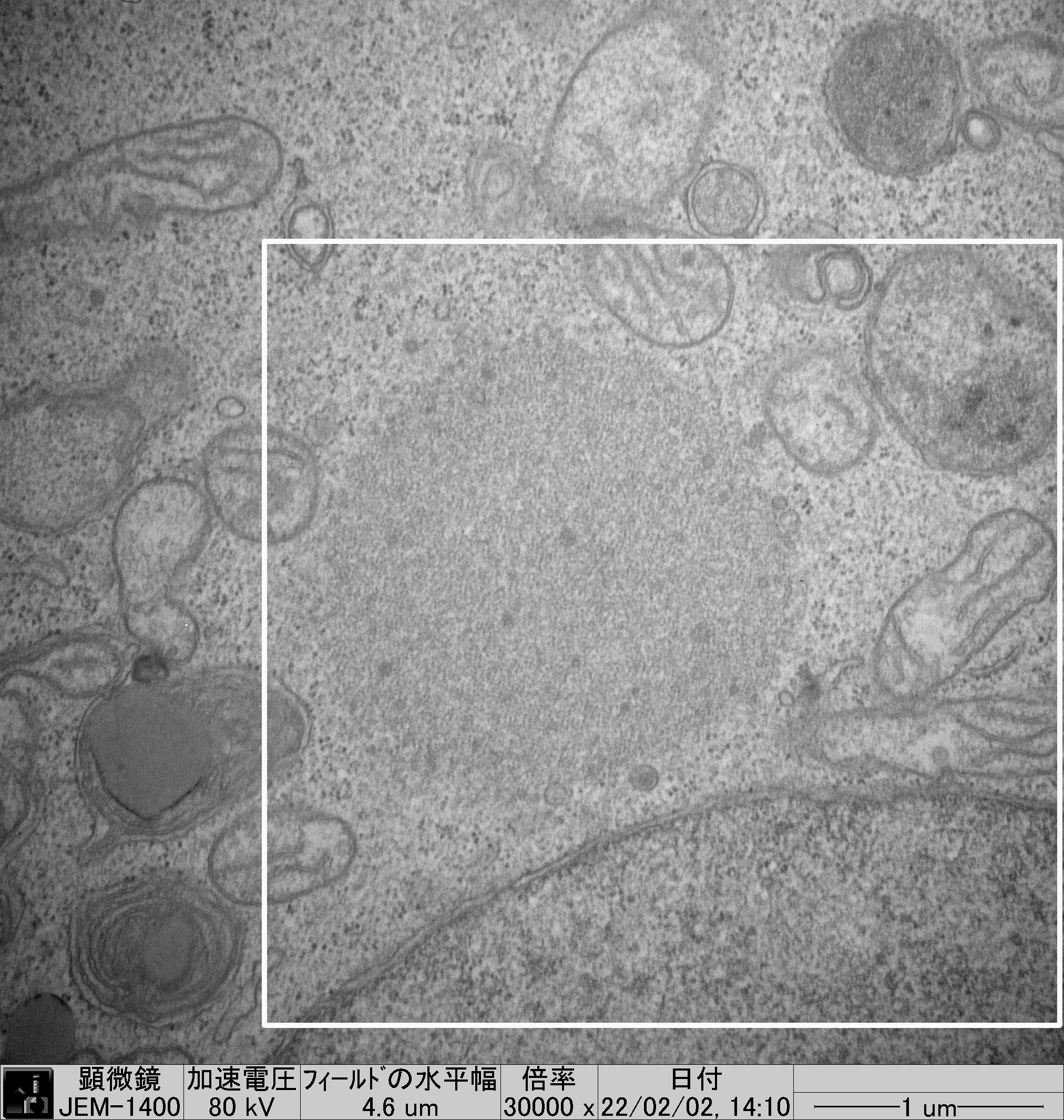

Supplement: Supplementary file 16 — Source Data for Figure 2 [file EMBJ-42-e113349-s016.zip › EMBOJ-2022-113349_SourceDataForFigure 2/2E/EM1.tif]

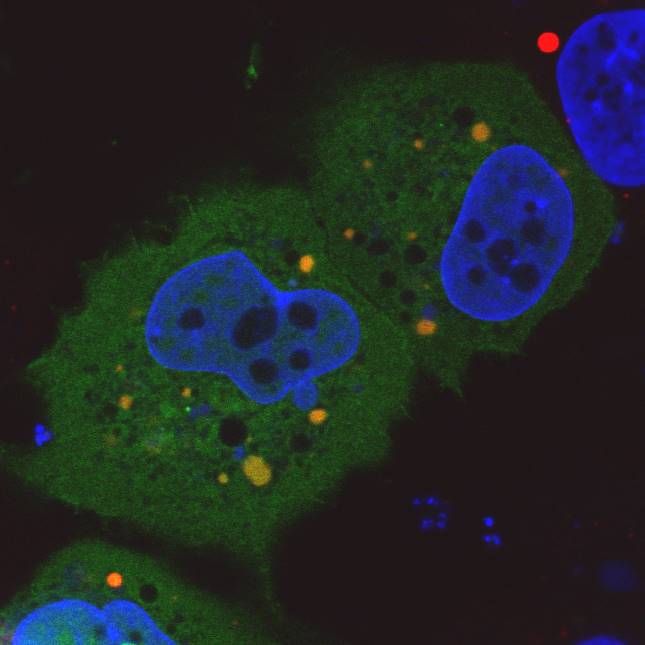

Supplement: Supplementary file 16 — Source Data for Figure 2 [file EMBJ-42-e113349-s016.zip › EMBOJ-2022-113349_SourceDataForFigure 2/2D/2D_GFPULK1p62_wild type_merged.jpg]

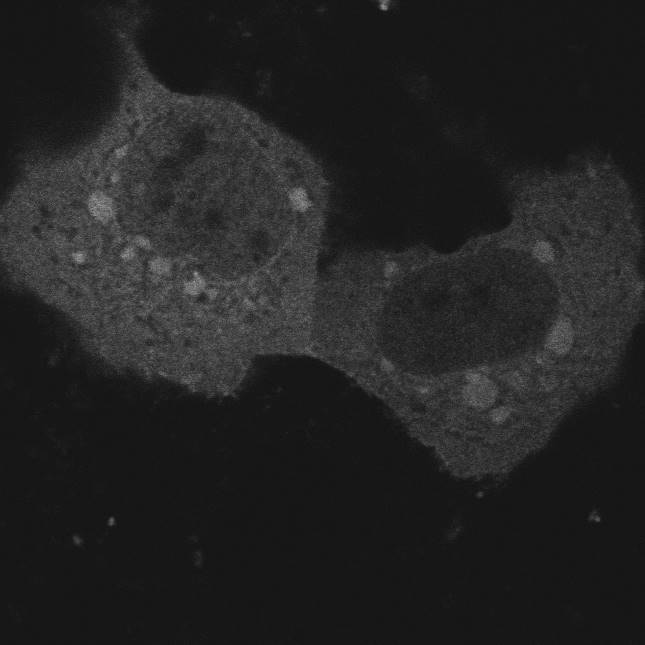

Supplement: Supplementary file 16 — Source Data for Figure 2 [file EMBJ-42-e113349-s016.zip › EMBOJ-2022-113349_SourceDataForFigure 2/2D/2D_GFPULK1p62_FIP200KO_GFP.jpg]

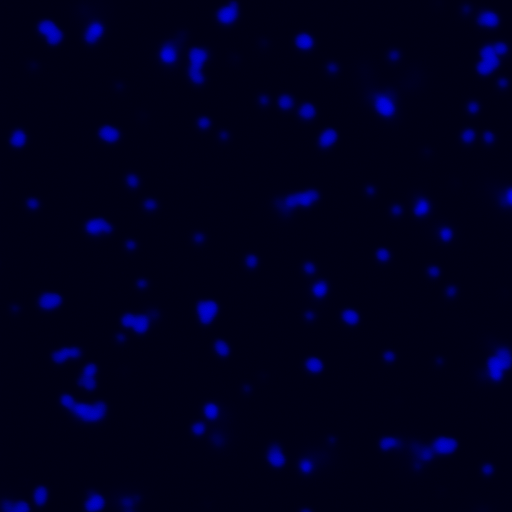

Supplement: Supplementary file 16 — Source Data for Figure 2 [file EMBJ-42-e113349-s016.zip › EMBOJ-2022-113349_SourceDataForFigure 2/2B/2B_Atg1-p62WT (3).tif]

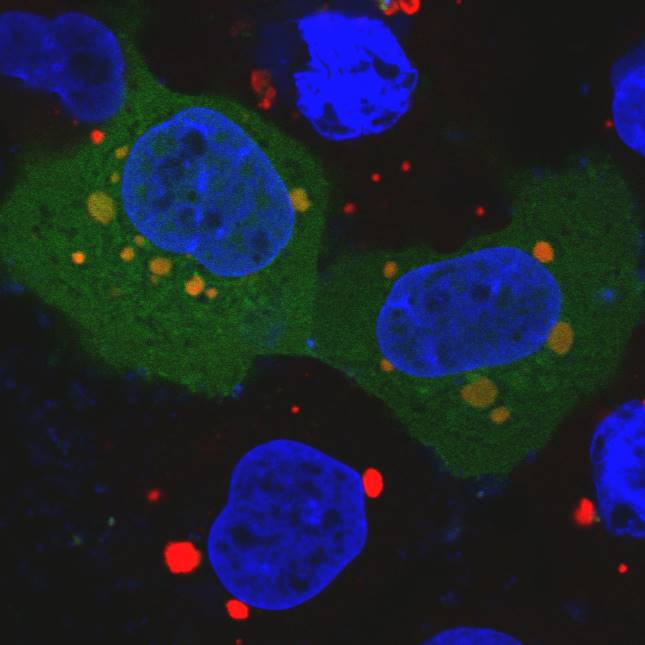

Supplement: Supplementary file 16 — Source Data for Figure 2 [file EMBJ-42-e113349-s016.zip › EMBOJ-2022-113349_SourceDataForFigure 2/2D/2D_GFPULK1p62_FIP200KO_merged.jpg]

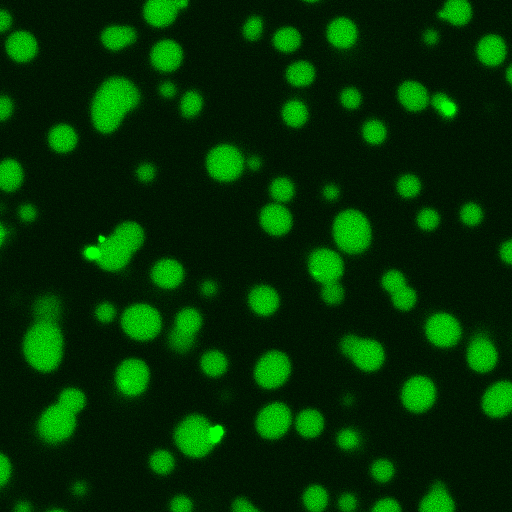

Supplement: Supplementary file 16 — Source Data for Figure 2 [file EMBJ-42-e113349-s016.zip › EMBOJ-2022-113349_SourceDataForFigure 2/2B/2B_ULK1-p62S403E S407E (1).tif]

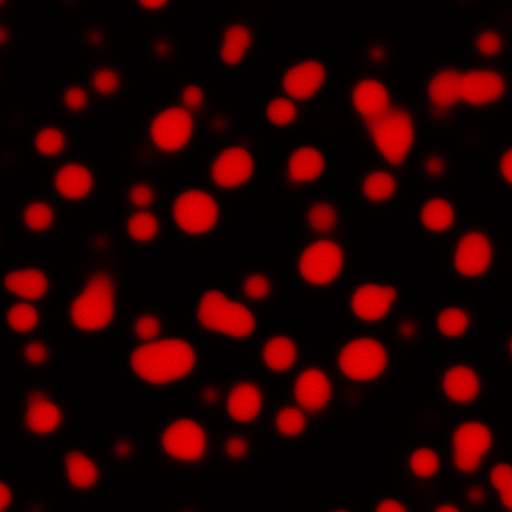

Supplement: Supplementary file 16 — Source Data for Figure 2 [file EMBJ-42-e113349-s016.zip › EMBOJ-2022-113349_SourceDataForFigure 2/2B/2B_Atg1-p62S403E S407E (2).tif]

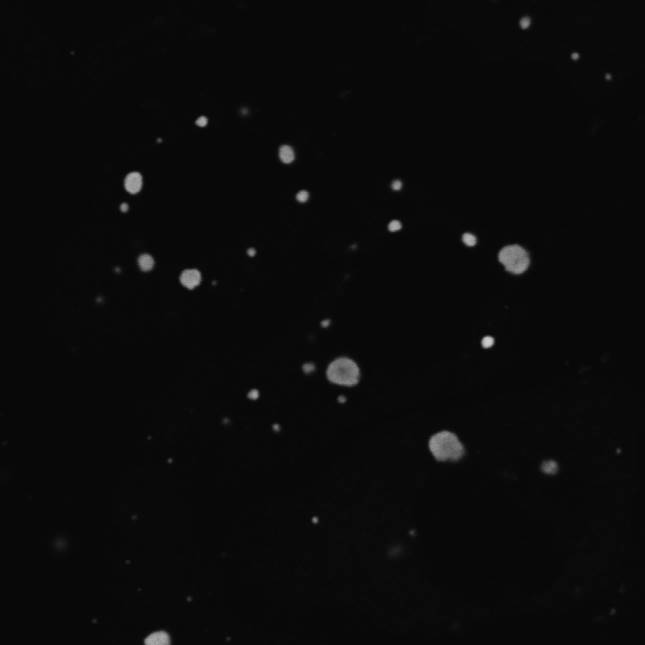

Supplement: Supplementary file 16 — Source Data for Figure 2 [file EMBJ-42-e113349-s016.zip › EMBOJ-2022-113349_SourceDataForFigure 2/2D/2D_GFPULK2p62_FIP200KO_p62.jpg]

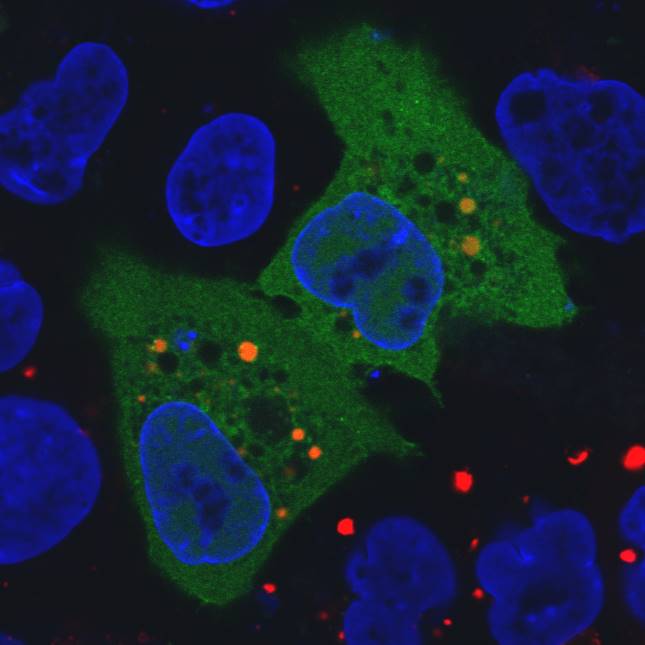

Supplement: Supplementary file 16 — Source Data for Figure 2 [file EMBJ-42-e113349-s016.zip › EMBOJ-2022-113349_SourceDataForFigure 2/2D/2D_GFPULK2p62_wild type_merged.jpg]

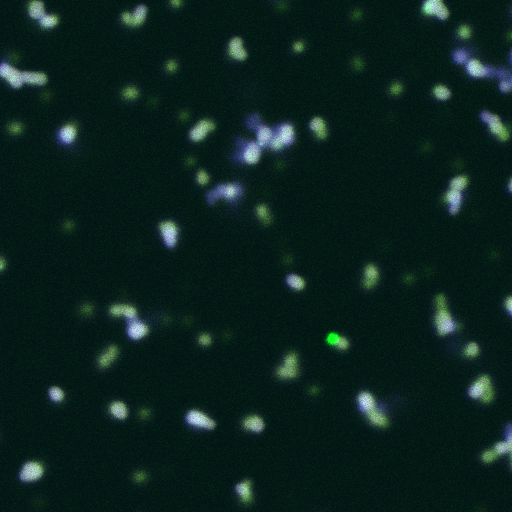

Supplement: Supplementary file 16 — Source Data for Figure 2 [file EMBJ-42-e113349-s016.zip › EMBOJ-2022-113349_SourceDataForFigure 2/2B/2B_ULK1-p62WT (4).tif]

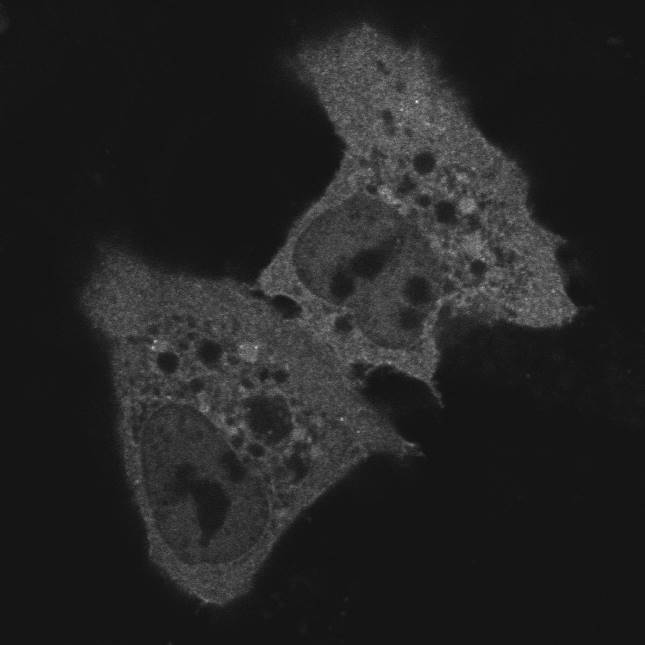

Supplement: Supplementary file 16 — Source Data for Figure 2 [file EMBJ-42-e113349-s016.zip › EMBOJ-2022-113349_SourceDataForFigure 2/2D/2D_GFPULK2p62_wild type_GFP.jpg]

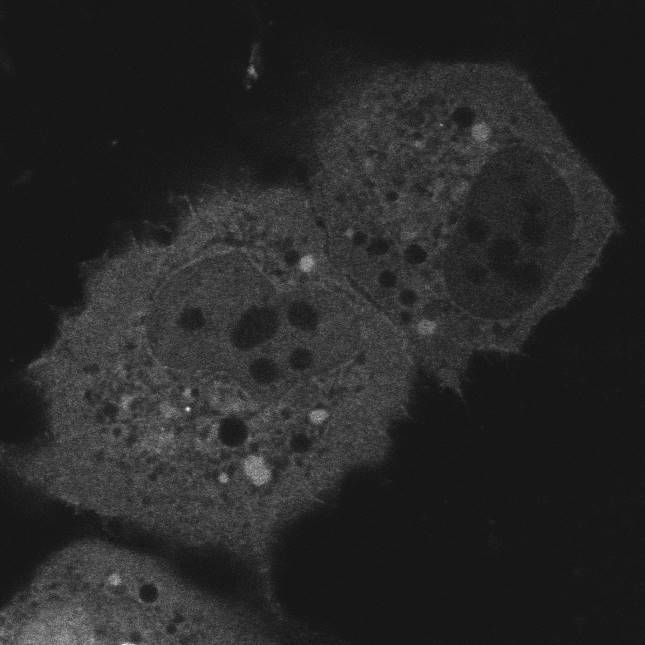

Supplement: Supplementary file 16 — Source Data for Figure 2 [file EMBJ-42-e113349-s016.zip › EMBOJ-2022-113349_SourceDataForFigure 2/2D/2D_GFPULK1p62_wild type_GFP.jpg]

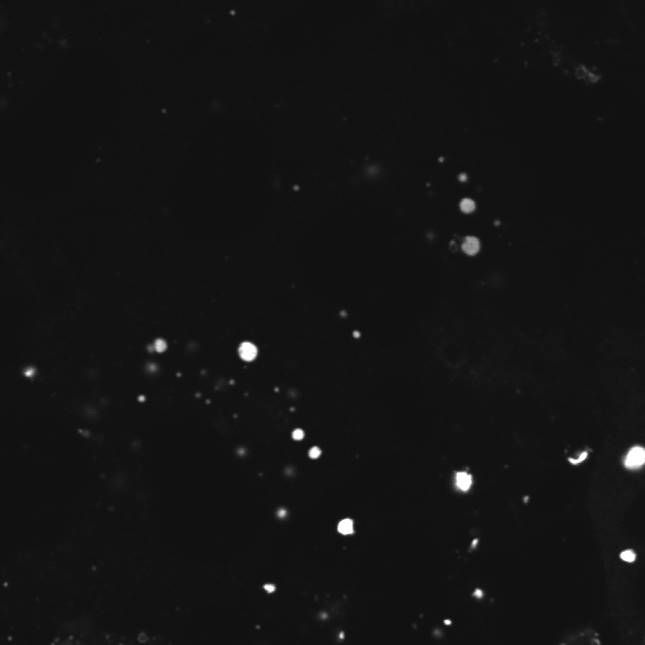

Supplement: Supplementary file 16 — Source Data for Figure 2 [file EMBJ-42-e113349-s016.zip › EMBOJ-2022-113349_SourceDataForFigure 2/2D/2D_GFPULK2p62_wild type_p62.jpg]

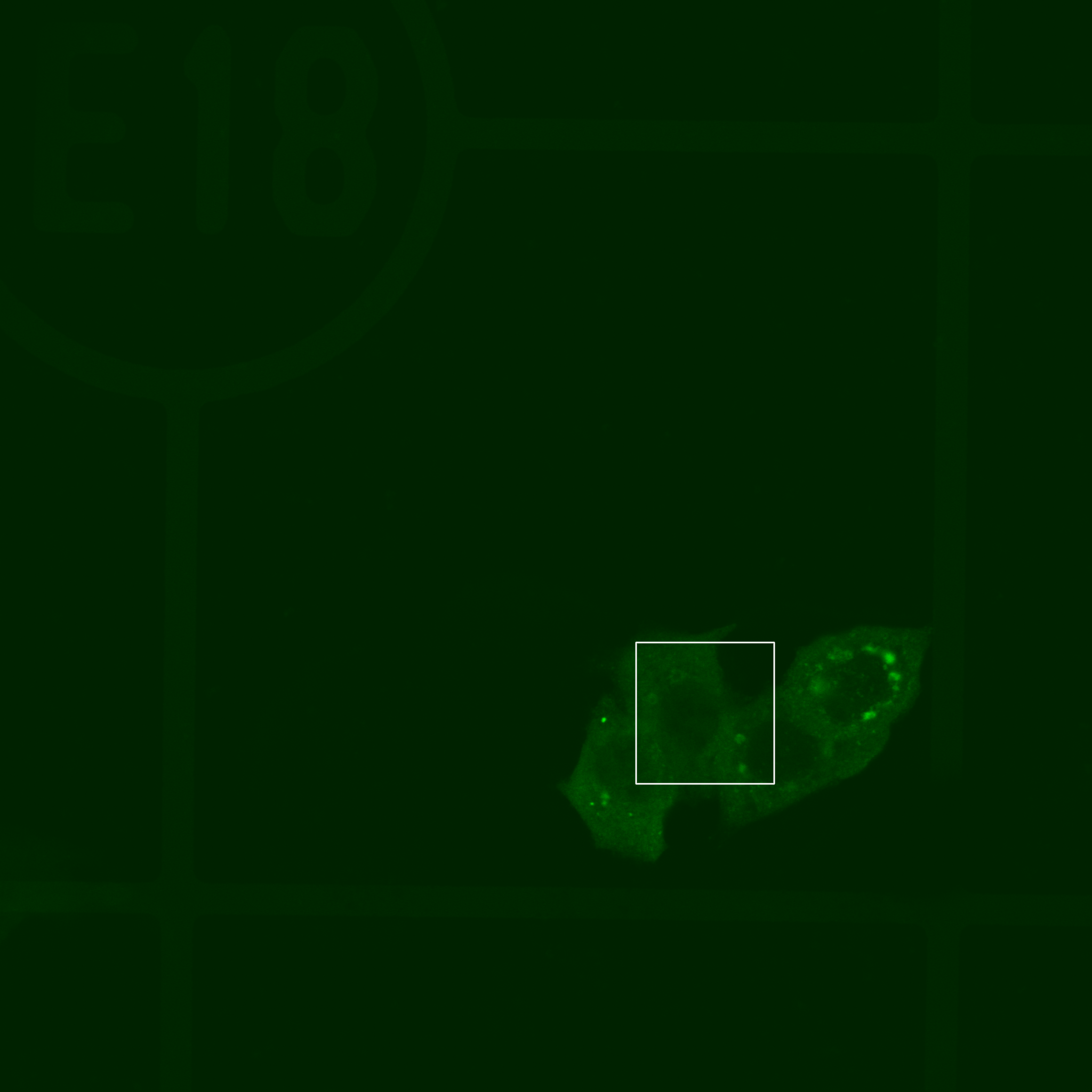

Supplement: Supplementary file 16 — Source Data for Figure 2 [file EMBJ-42-e113349-s016.zip › EMBOJ-2022-113349_SourceDataForFigure 2/2E/GFP-ULK1.tif]

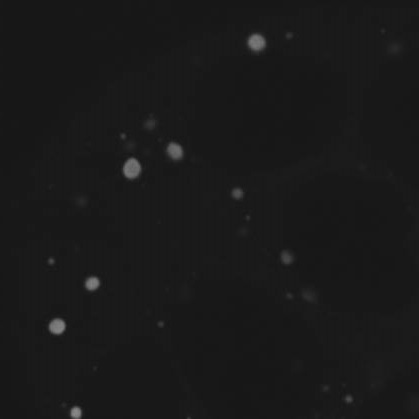

Supplement: Supplementary file 16 — Source Data for Figure 2 [file EMBJ-42-e113349-s016.zip › EMBOJ-2022-113349_SourceDataForFigure 2/2C/2C_ULK1p62_wild type_p62.jpg]

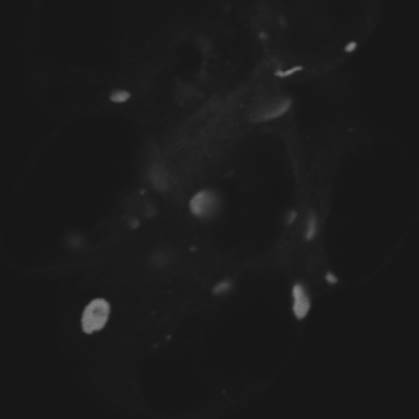

Supplement: Supplementary file 16 — Source Data for Figure 2 [file EMBJ-42-e113349-s016.zip › EMBOJ-2022-113349_SourceDataForFigure 2/2C/2C_ULK1p62_FIP200KO_p62.jpg]

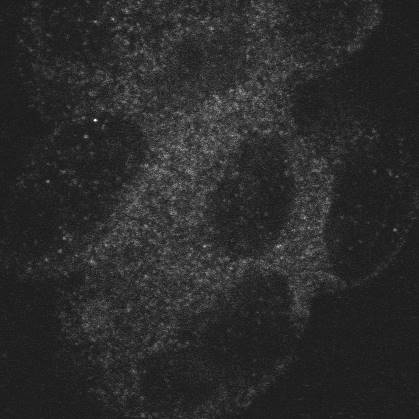

Supplement: Supplementary file 16 — Source Data for Figure 2 [file EMBJ-42-e113349-s016.zip › EMBOJ-2022-113349_SourceDataForFigure 2/2C/2C_ULK1p62_FIP200KO_ULK1.jpg]

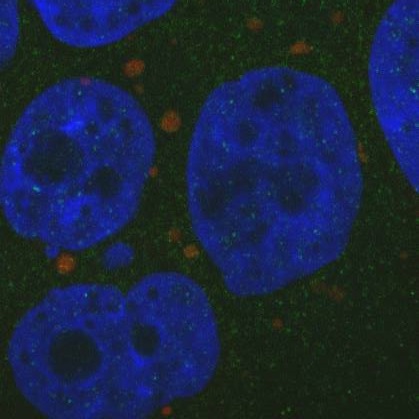

Supplement: Supplementary file 16 — Source Data for Figure 2 [file EMBJ-42-e113349-s016.zip › EMBOJ-2022-113349_SourceDataForFigure 2/2C/2C_ULK1p62_ULK1KO_merged.jpg]

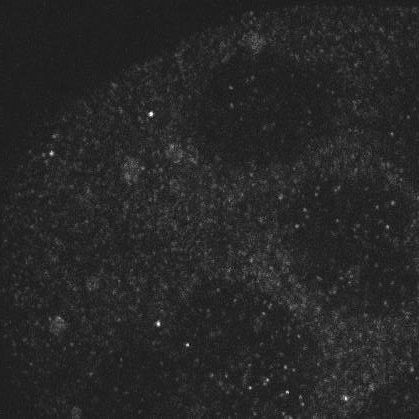

Supplement: Supplementary file 16 — Source Data for Figure 2 [file EMBJ-42-e113349-s016.zip › EMBOJ-2022-113349_SourceDataForFigure 2/2C/2C_ULK1p62_wild type_ULK1.jpg]

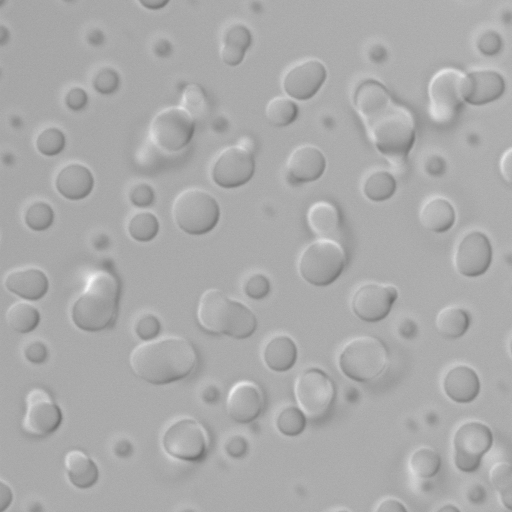

Supplement: Supplementary file 16 — Source Data for Figure 2 [file EMBJ-42-e113349-s016.zip › EMBOJ-2022-113349_SourceDataForFigure 2/2B/2B_Atg1-p62S403E S407E (5).tif]

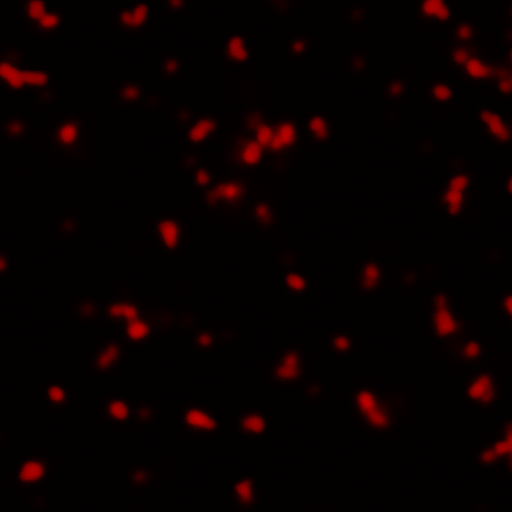

Supplement: Supplementary file 16 — Source Data for Figure 2 [file EMBJ-42-e113349-s016.zip › EMBOJ-2022-113349_SourceDataForFigure 2/2B/2B_ULK1-p62WT (2).tif]

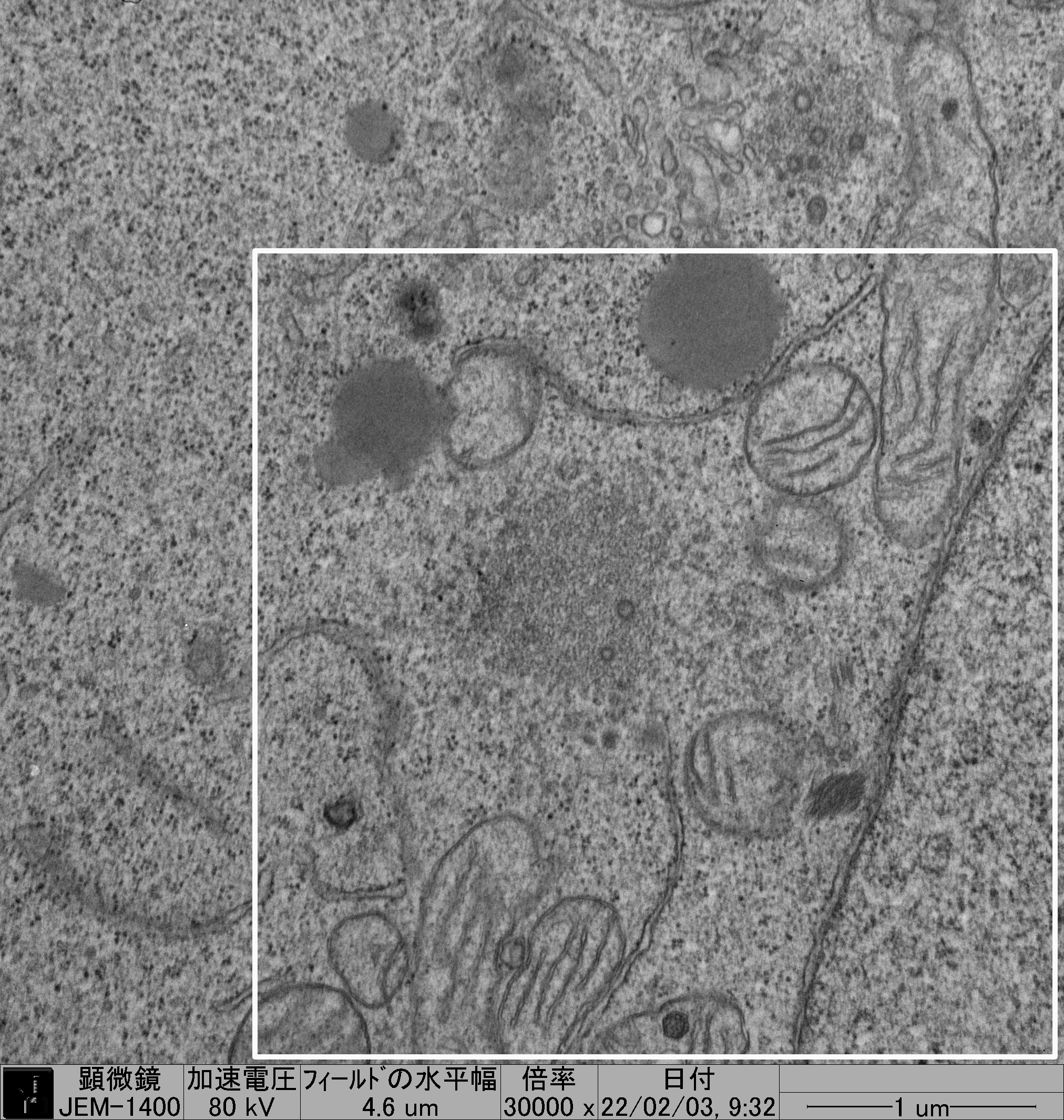

Supplement: Supplementary file 16 — Source Data for Figure 2 [file EMBJ-42-e113349-s016.zip › EMBOJ-2022-113349_SourceDataForFigure 2/2E/EM2_.tif]

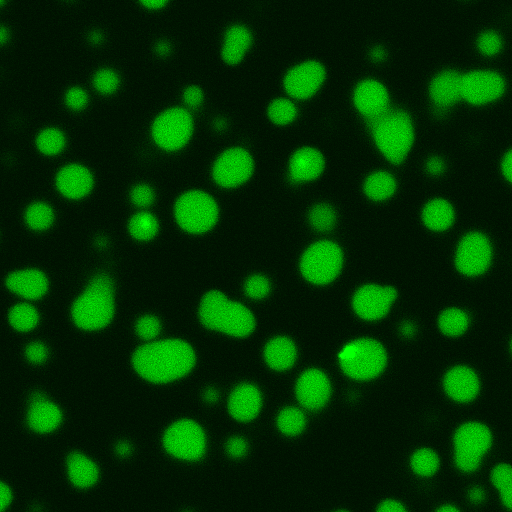

Supplement: Supplementary file 16 — Source Data for Figure 2 [file EMBJ-42-e113349-s016.zip › EMBOJ-2022-113349_SourceDataForFigure 2/2B/2B_Atg1-p62S403E S407E (1).tif]

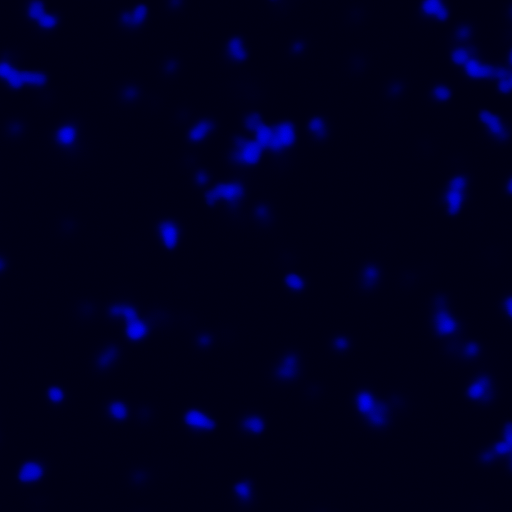

Supplement: Supplementary file 16 — Source Data for Figure 2 [file EMBJ-42-e113349-s016.zip › EMBOJ-2022-113349_SourceDataForFigure 2/2B/2B_ULK1-p62WT (3).tif]

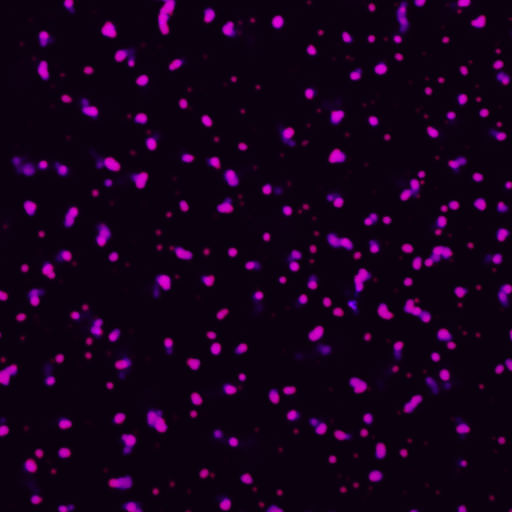

Supplement: Supplementary file 16 — Source Data for Figure 2 [file EMBJ-42-e113349-s016.zip › EMBOJ-2022-113349_SourceDataForFigure 2/2A/2A_p62S349E (3).tif]

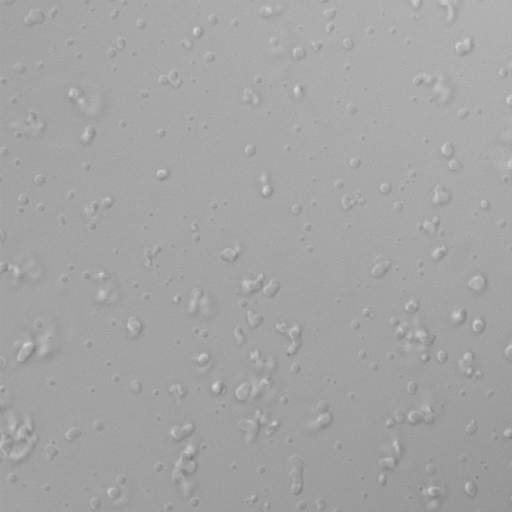

Supplement: Supplementary file 16 — Source Data for Figure 2 [file EMBJ-42-e113349-s016.zip › EMBOJ-2022-113349_SourceDataForFigure 2/2A/2A_p62WT (4).tif]

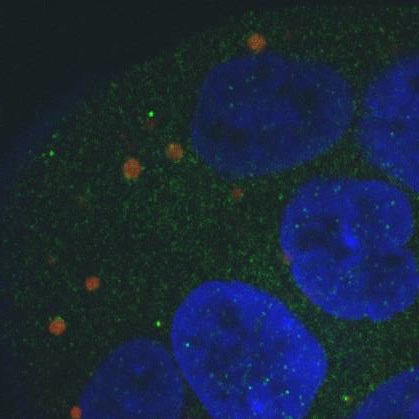

Supplement: Supplementary file 16 — Source Data for Figure 2 [file EMBJ-42-e113349-s016.zip › EMBOJ-2022-113349_SourceDataForFigure 2/2C/2C_ULK1p62_wild type_merged.jpg]

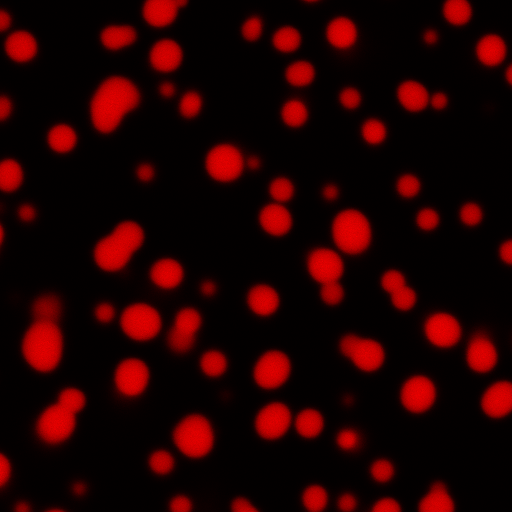

Supplement: Supplementary file 16 — Source Data for Figure 2 [file EMBJ-42-e113349-s016.zip › EMBOJ-2022-113349_SourceDataForFigure 2/2B/2B_ULK1-p62S403E S407E (2).tif]

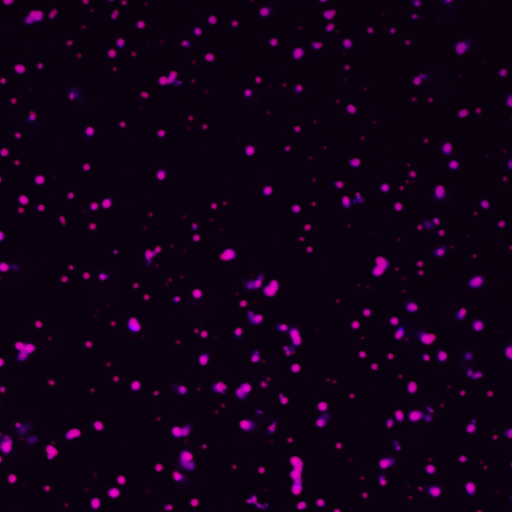

Supplement: Supplementary file 16 — Source Data for Figure 2 [file EMBJ-42-e113349-s016.zip › EMBOJ-2022-113349_SourceDataForFigure 2/2A/2A_p62WT (3).tif]

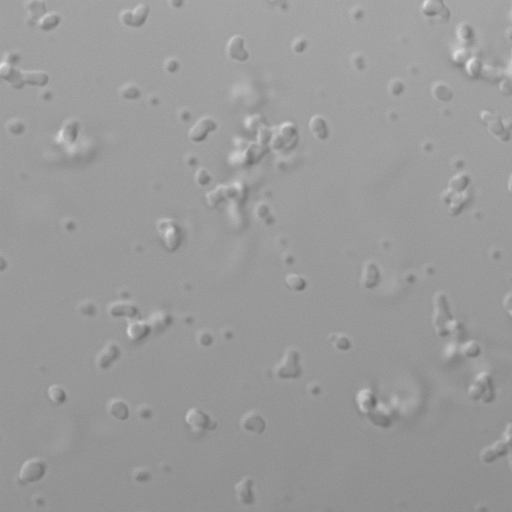

Supplement: Supplementary file 16 — Source Data for Figure 2 [file EMBJ-42-e113349-s016.zip › EMBOJ-2022-113349_SourceDataForFigure 2/2B/2B_ULK1-p62WT (5).tif]

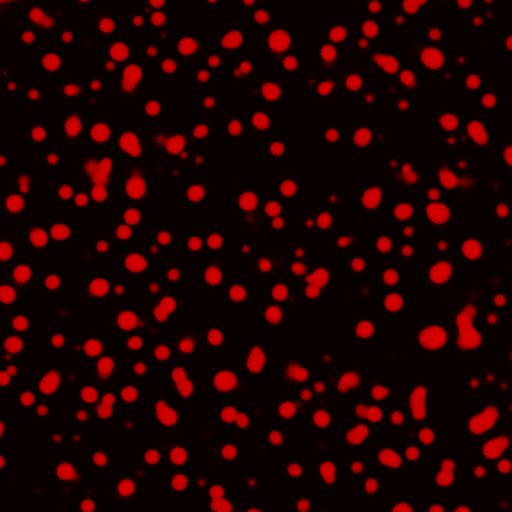

Supplement: Supplementary file 16 — Source Data for Figure 2 [file EMBJ-42-e113349-s016.zip › EMBOJ-2022-113349_SourceDataForFigure 2/2A/2A_p62S403E S407E (1).tif]

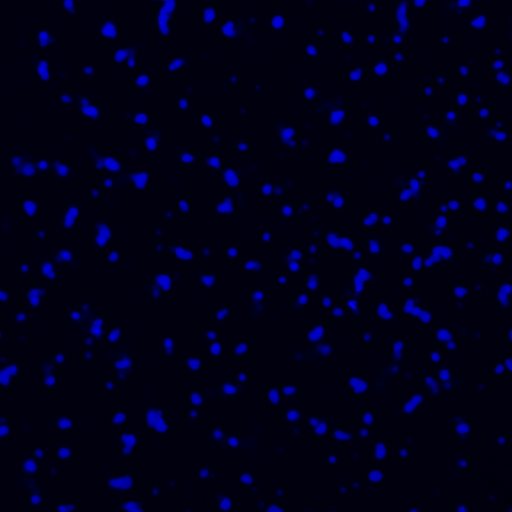

Supplement: Supplementary file 16 — Source Data for Figure 2 [file EMBJ-42-e113349-s016.zip › EMBOJ-2022-113349_SourceDataForFigure 2/2A/2A_p62S349E (2).tif]

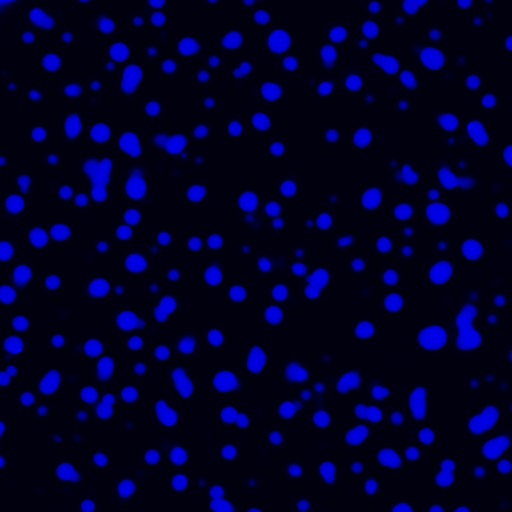

Supplement: Supplementary file 16 — Source Data for Figure 2 [file EMBJ-42-e113349-s016.zip › EMBOJ-2022-113349_SourceDataForFigure 2/2A/2A_p62S403E S407E (2).tif]

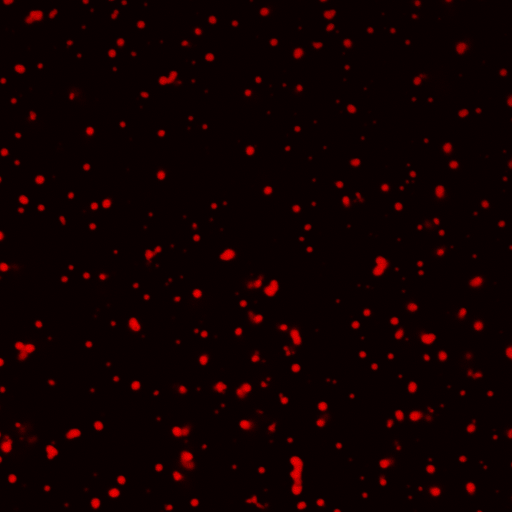

Supplement: Supplementary file 16 — Source Data for Figure 2 [file EMBJ-42-e113349-s016.zip › EMBOJ-2022-113349_SourceDataForFigure 2/2A/2A_p62WT (1).tif]

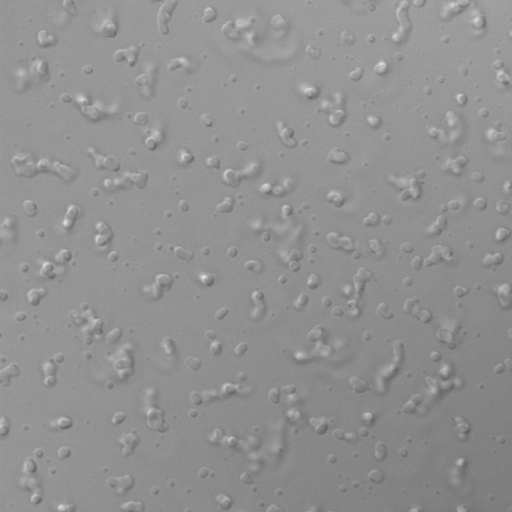

Supplement: Supplementary file 16 — Source Data for Figure 2 [file EMBJ-42-e113349-s016.zip › EMBOJ-2022-113349_SourceDataForFigure 2/2A/2A_p62S349E (4).tif]

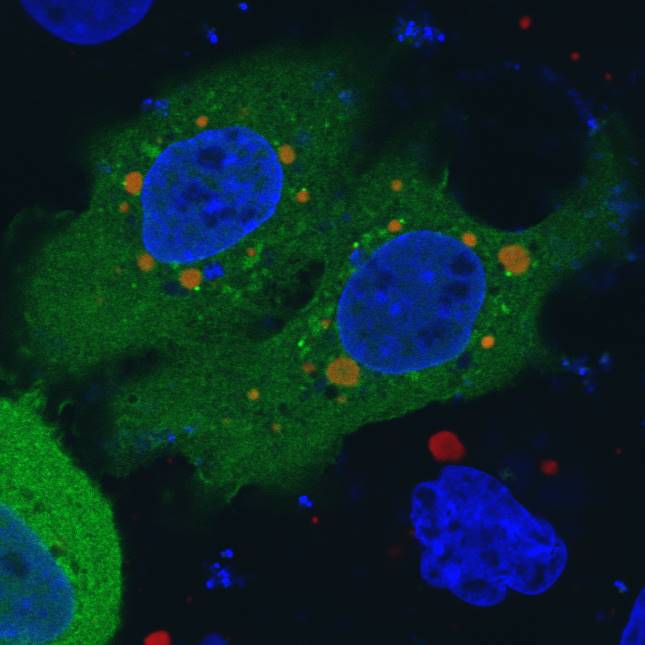

Supplement: Supplementary file 16 — Source Data for Figure 2 [file EMBJ-42-e113349-s016.zip › EMBOJ-2022-113349_SourceDataForFigure 2/2D/2D_GFPULK2p62_FIP200KO_merged.jpg]

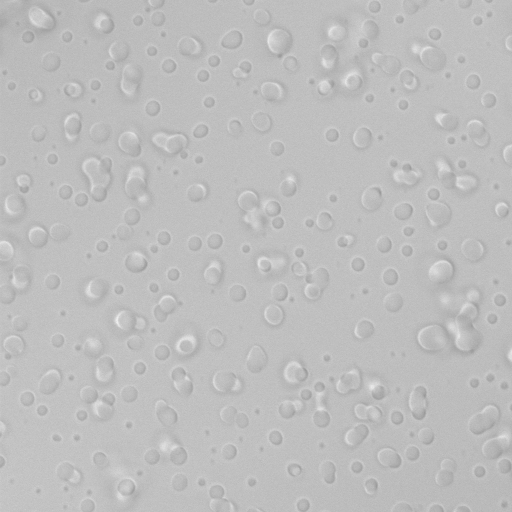

Supplement: Supplementary file 16 — Source Data for Figure 2 [file EMBJ-42-e113349-s016.zip › EMBOJ-2022-113349_SourceDataForFigure 2/2A/2A_p62S403E S407E (4).tif]

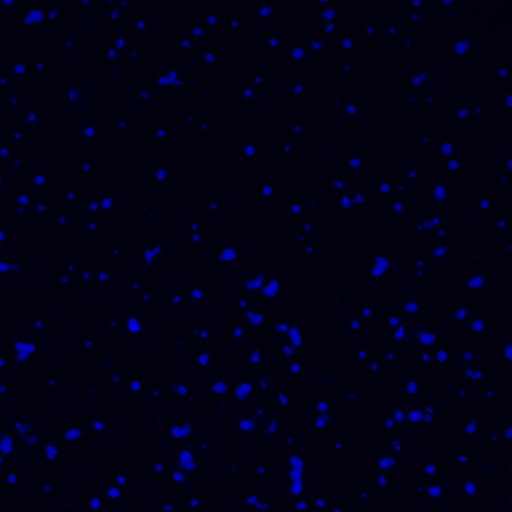

Supplement: Supplementary file 16 — Source Data for Figure 2 [file EMBJ-42-e113349-s016.zip › EMBOJ-2022-113349_SourceDataForFigure 2/2A/2A_p62WT (2).tif]

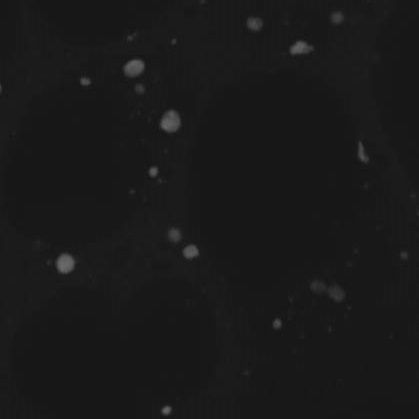

Supplement: Supplementary file 16 — Source Data for Figure 2 [file EMBJ-42-e113349-s016.zip › EMBOJ-2022-113349_SourceDataForFigure 2/2C/2C_ULK1p62_ULK1KO_p62.jpg]

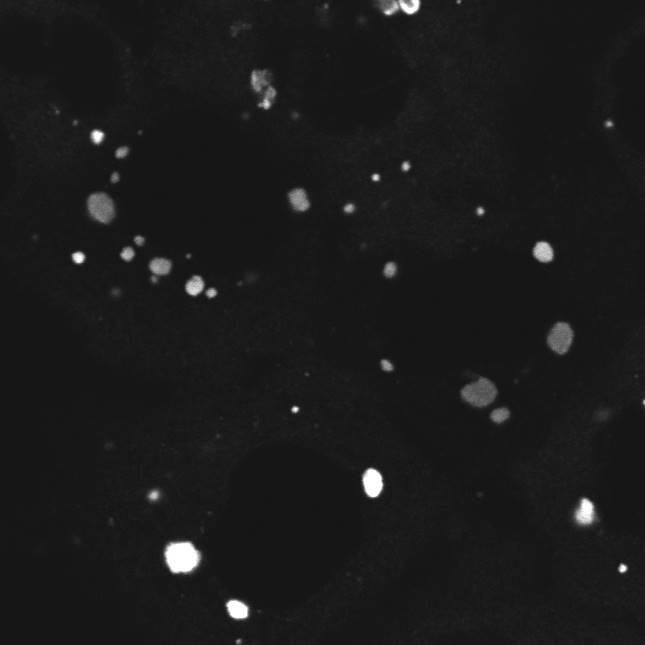

Supplement: Supplementary file 16 — Source Data for Figure 2 [file EMBJ-42-e113349-s016.zip › EMBOJ-2022-113349_SourceDataForFigure 2/2D/2D_GFPULK1p62_FIP200KO_p62.jpg]

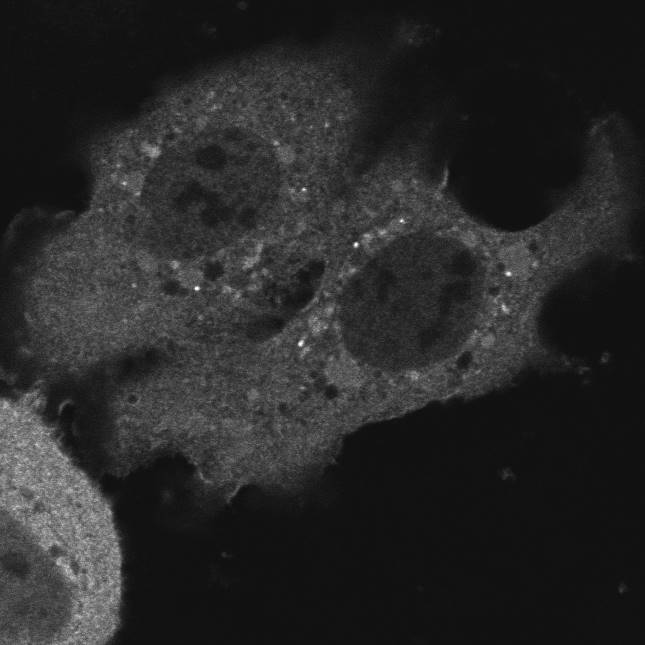

Supplement: Supplementary file 16 — Source Data for Figure 2 [file EMBJ-42-e113349-s016.zip › EMBOJ-2022-113349_SourceDataForFigure 2/2D/2D_GFPULK2p62_FIP200KO_GFP.jpg]

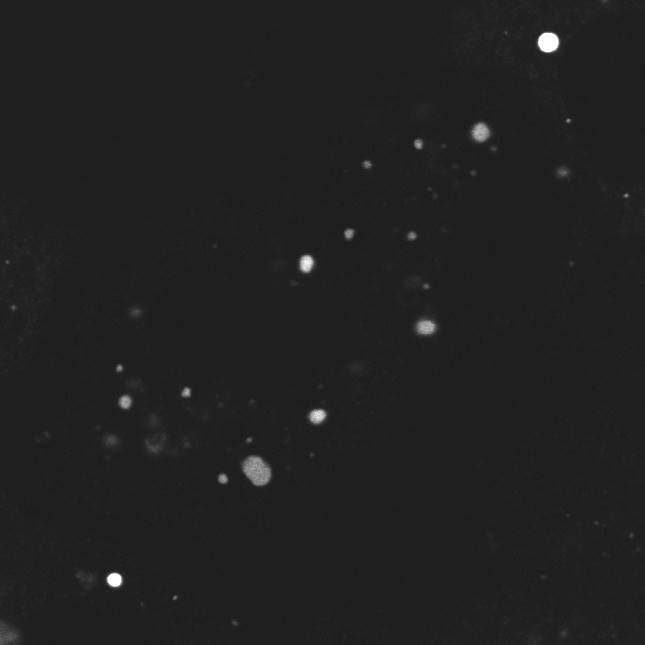

Supplement: Supplementary file 16 — Source Data for Figure 2 [file EMBJ-42-e113349-s016.zip › EMBOJ-2022-113349_SourceDataForFigure 2/2D/2D_GFPULK1p62_wild type_p62.jpg]

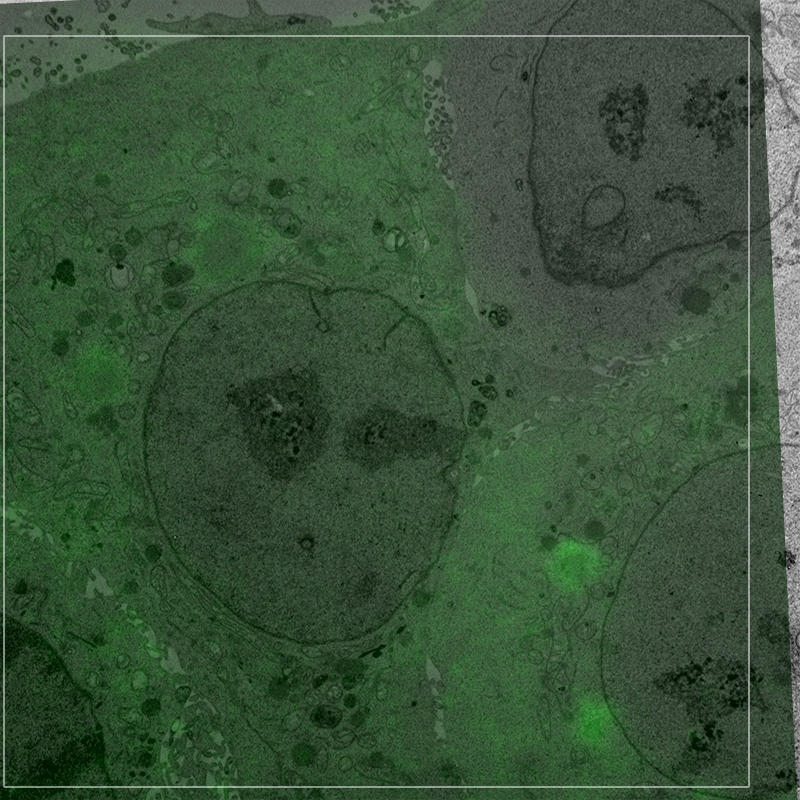

Supplement: Supplementary file 16 — Source Data for Figure 2 [file EMBJ-42-e113349-s016.zip › EMBOJ-2022-113349_SourceDataForFigure 2/2E/CLEM.tif]

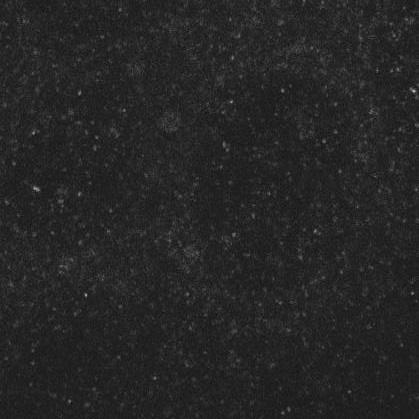

Supplement: Supplementary file 16 — Source Data for Figure 2 [file EMBJ-42-e113349-s016.zip › EMBOJ-2022-113349_SourceDataForFigure 2/2C/2C_ULK1p62_ULK1KO_ULK1.jpg]

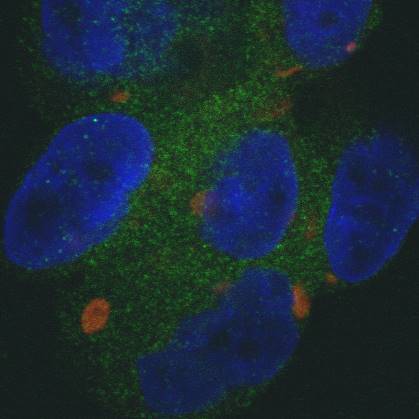

Supplement: Supplementary file 16 — Source Data for Figure 2 [file EMBJ-42-e113349-s016.zip › EMBOJ-2022-113349_SourceDataForFigure 2/2C/2C_ULK1p62_FIP200KO_merged.jpg]

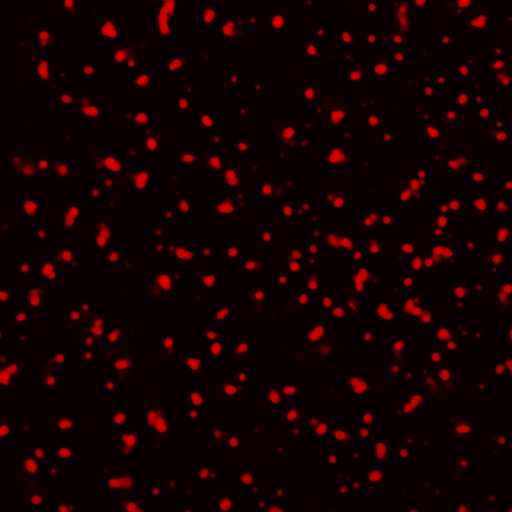

Supplement: Supplementary file 16 — Source Data for Figure 2 [file EMBJ-42-e113349-s016.zip › EMBOJ-2022-113349_SourceDataForFigure 2/2A/2A_p62S349E (1).tif]

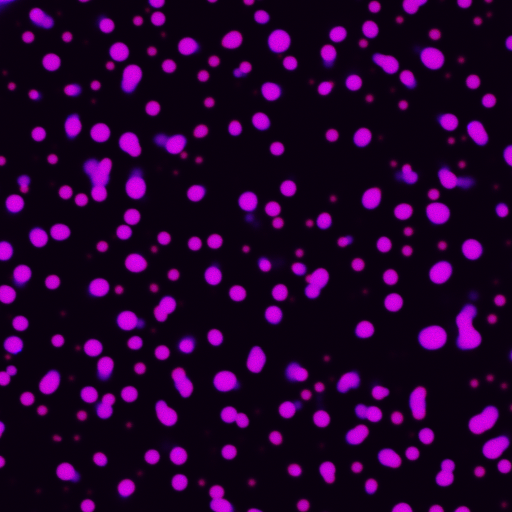

Supplement: Supplementary file 16 — Source Data for Figure 2 [file EMBJ-42-e113349-s016.zip › EMBOJ-2022-113349_SourceDataForFigure 2/2A/2A_p62S403E S407E (3).tif]

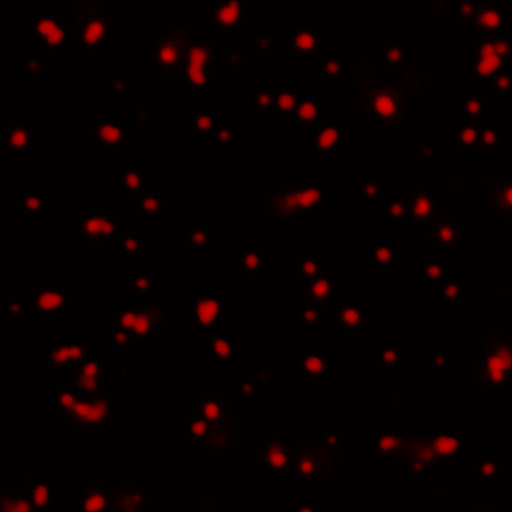

Supplement: Supplementary file 16 — Source Data for Figure 2 [file EMBJ-42-e113349-s016.zip › EMBOJ-2022-113349_SourceDataForFigure 2/2B/2B_Atg1-p62WT (2).tif]

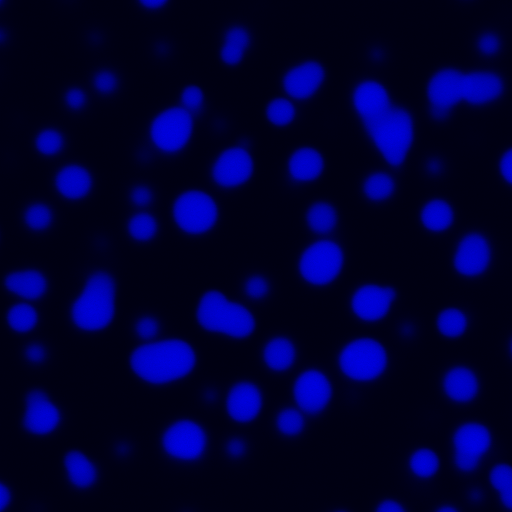

Supplement: Supplementary file 16 — Source Data for Figure 2 [file EMBJ-42-e113349-s016.zip › EMBOJ-2022-113349_SourceDataForFigure 2/2B/2B_Atg1-p62S403E S407E (3).tif]

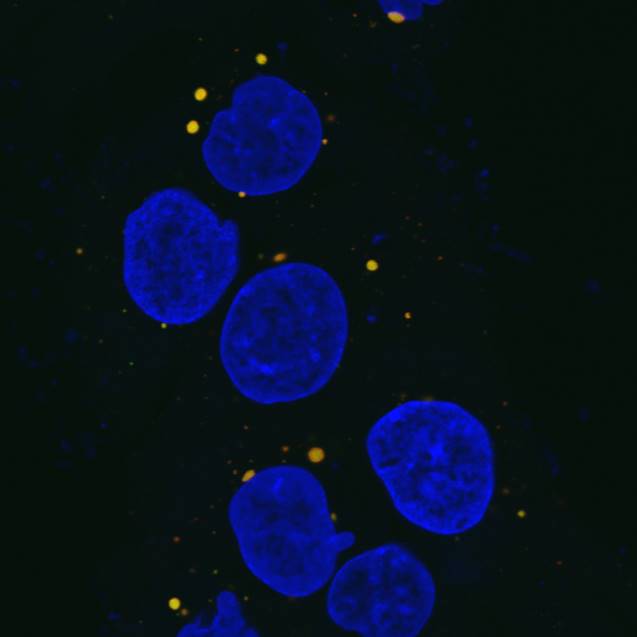

Supplement: Supplementary file 17 — Source Data for Figure 3 [file EMBJ-42-e113349-s008.zip › EMBOJ-2022-113349_SourceDataForFigure 3/3C/3C_KEAP1p62_DMSO_merged.jpg]

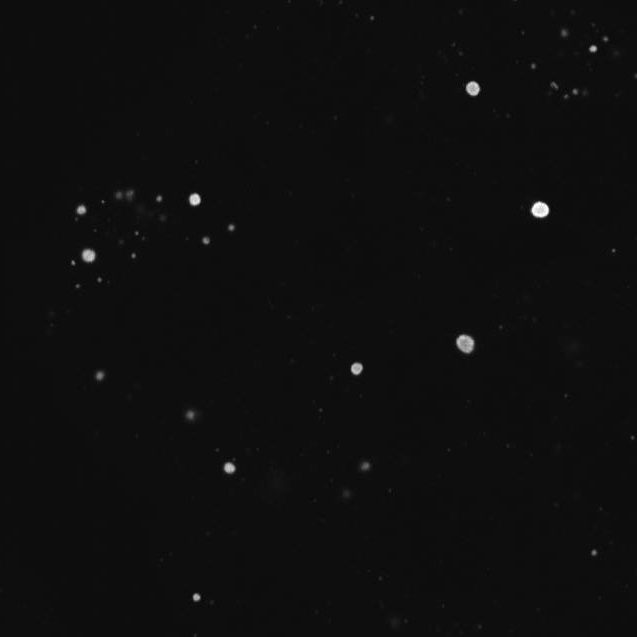

Supplement: Supplementary file 17 — Source Data for Figure 3 [file EMBJ-42-e113349-s008.zip › EMBOJ-2022-113349_SourceDataForFigure 3/3C/3C_pS349p62_DMSO_pS349.jpg]

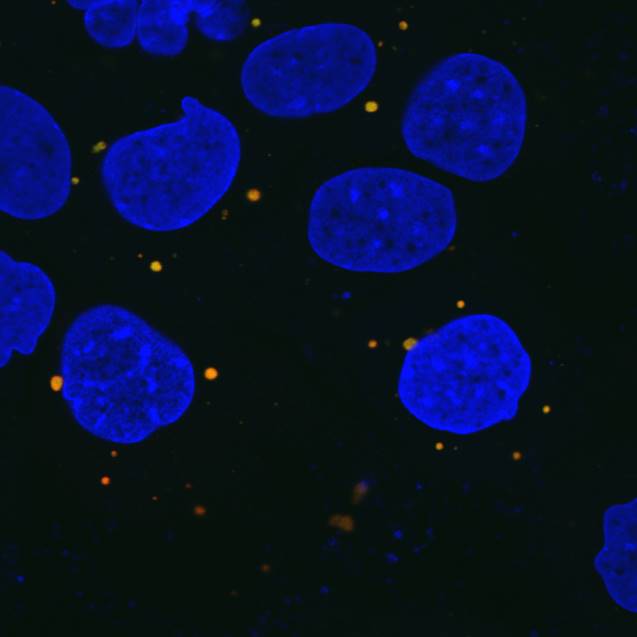

Supplement: Supplementary file 17 — Source Data for Figure 3 [file EMBJ-42-e113349-s008.zip › EMBOJ-2022-113349_SourceDataForFigure 3/3C/3C_KEAP1p62_MRT68921_merged.jpg]

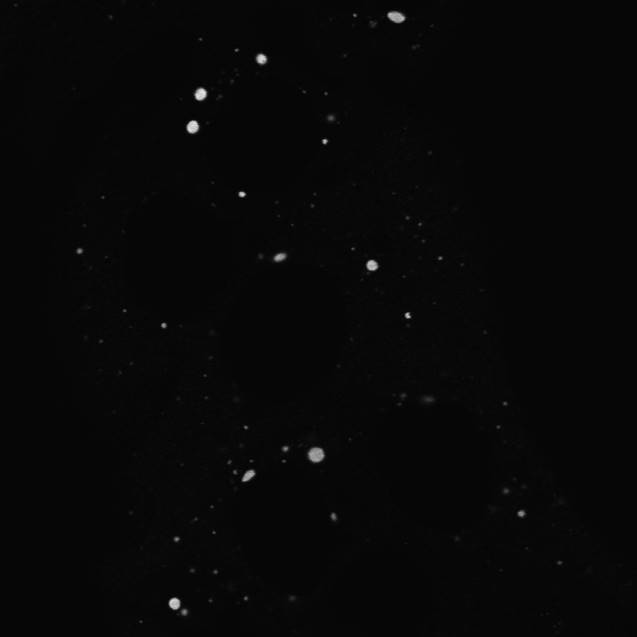

Supplement: Supplementary file 17 — Source Data for Figure 3 [file EMBJ-42-e113349-s008.zip › EMBOJ-2022-113349_SourceDataForFigure 3/3C/3C_KEAP1p62_DMSO_p62.jpg]

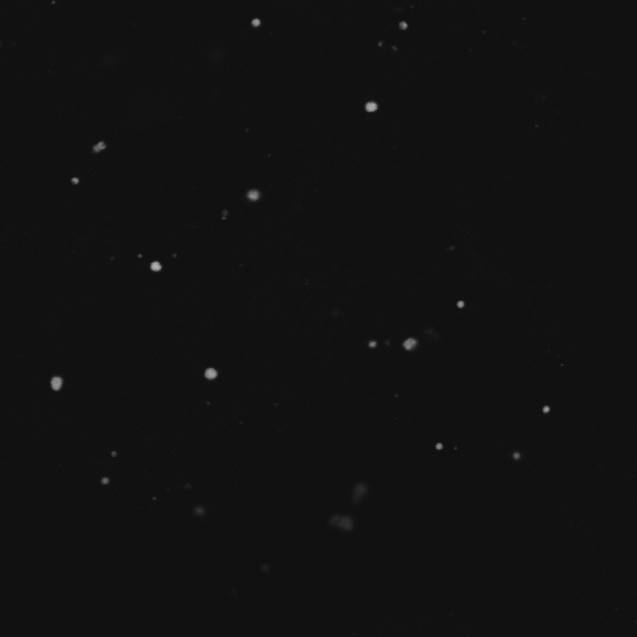

Supplement: Supplementary file 17 — Source Data for Figure 3 [file EMBJ-42-e113349-s008.zip › EMBOJ-2022-113349_SourceDataForFigure 3/3C/3C_KEAP1p62_MRT68921_KEAP1.jpg]

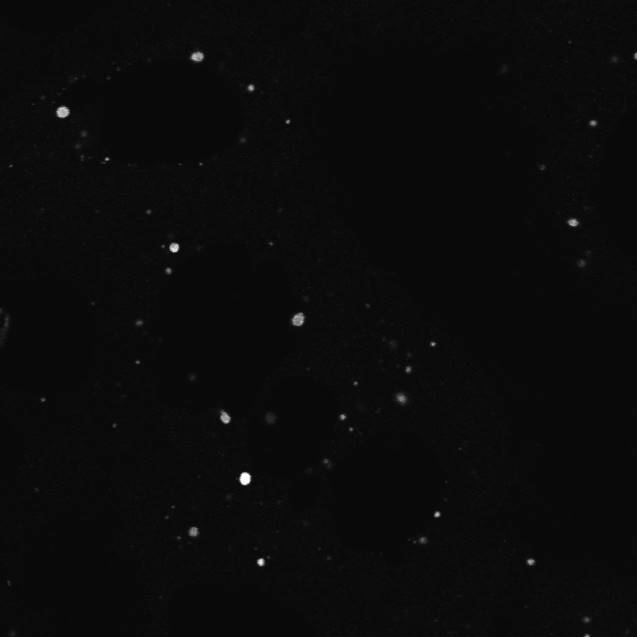

Supplement: Supplementary file 17 — Source Data for Figure 3 [file EMBJ-42-e113349-s008.zip › EMBOJ-2022-113349_SourceDataForFigure 3/3C/3C_pS349p62_MRT68921_p62.jpg]

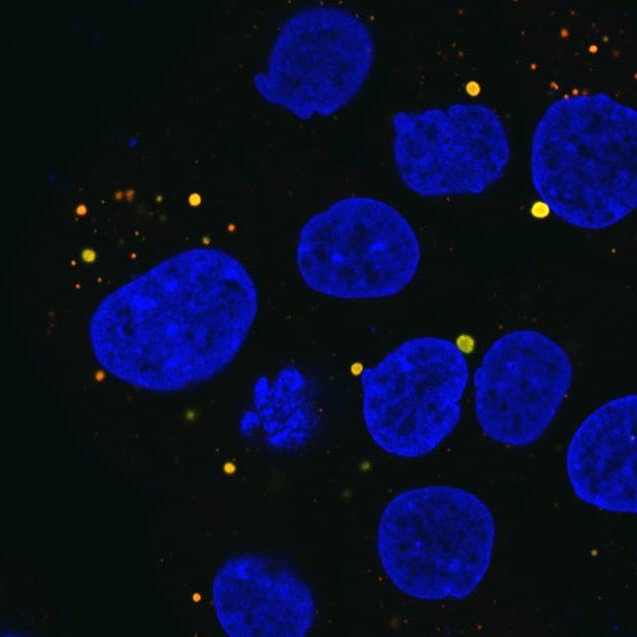

Supplement: Supplementary file 17 — Source Data for Figure 3 [file EMBJ-42-e113349-s008.zip › EMBOJ-2022-113349_SourceDataForFigure 3/3C/3C_pS349p62_DMSO_merged.jpg]

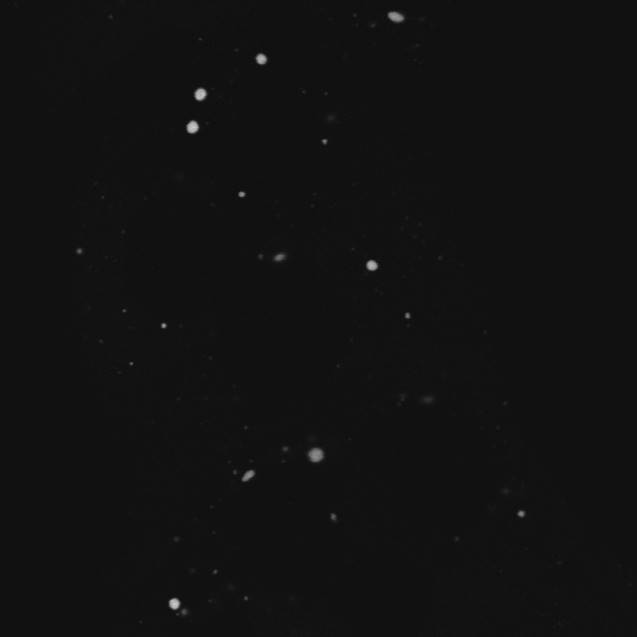

Supplement: Supplementary file 17 — Source Data for Figure 3 [file EMBJ-42-e113349-s008.zip › EMBOJ-2022-113349_SourceDataForFigure 3/3C/3C_KEAP1p62_DMSO_KEAP1.jpg]

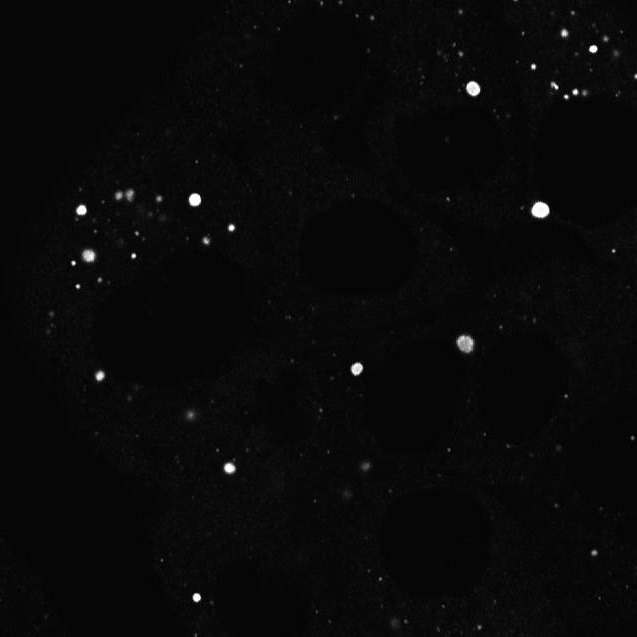

Supplement: Supplementary file 17 — Source Data for Figure 3 [file EMBJ-42-e113349-s008.zip › EMBOJ-2022-113349_SourceDataForFigure 3/3C/3C_pS349p62_DMSO_p62.jpg]

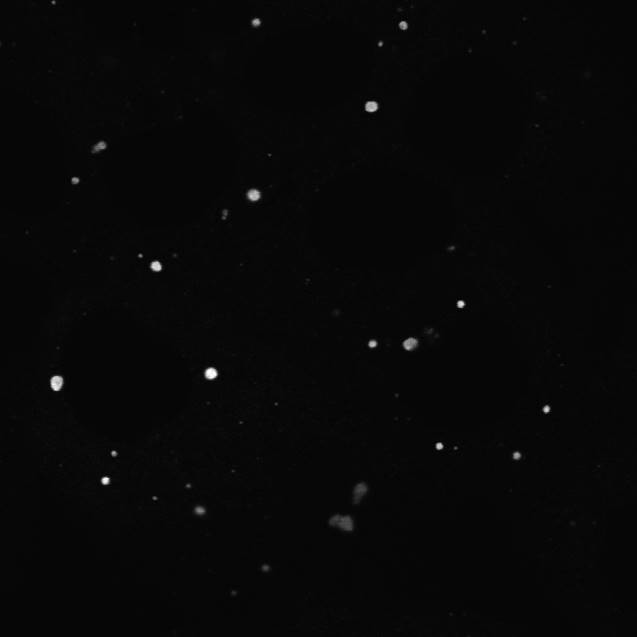

Supplement: Supplementary file 17 — Source Data for Figure 3 [file EMBJ-42-e113349-s008.zip › EMBOJ-2022-113349_SourceDataForFigure 3/3C/3C_KEAP1p62_MRT68921_p62.jpg]

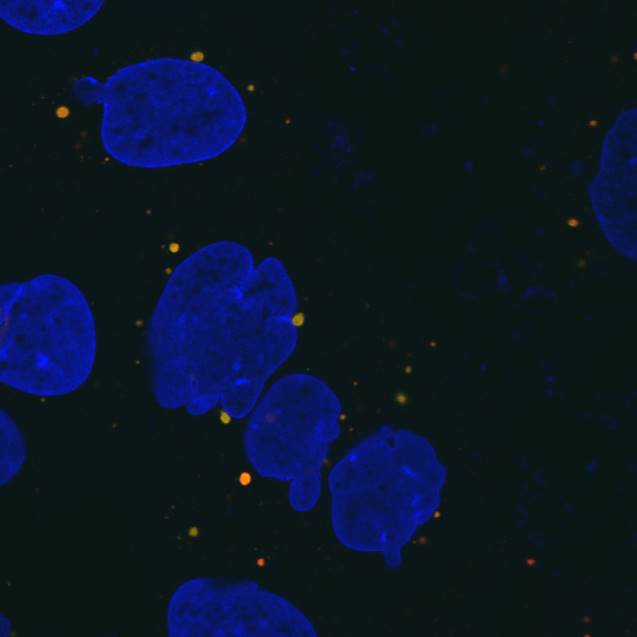

Supplement: Supplementary file 17 — Source Data for Figure 3 [file EMBJ-42-e113349-s008.zip › EMBOJ-2022-113349_SourceDataForFigure 3/3C/3C_pS349p62_MRT68921_merged.jpg]

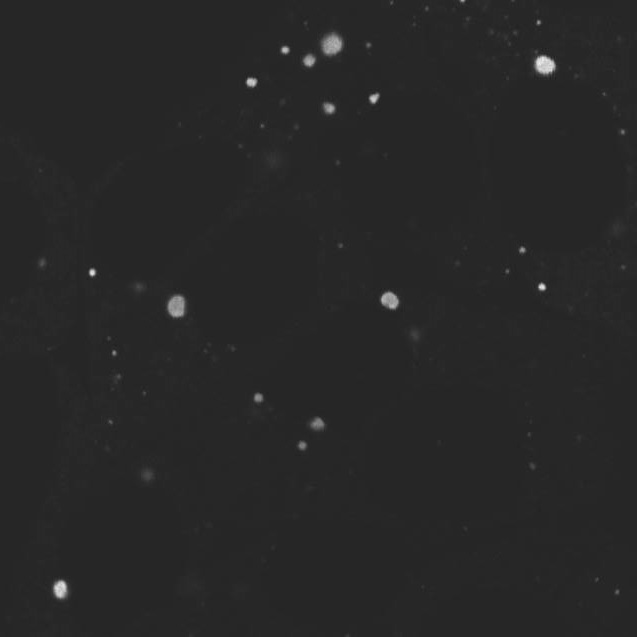

Supplement: Supplementary file 17 — Source Data for Figure 3 [file EMBJ-42-e113349-s008.zip › EMBOJ-2022-113349_SourceDataForFigure 3/3B/3B_S403,p62_DMSO_p62.jpg]

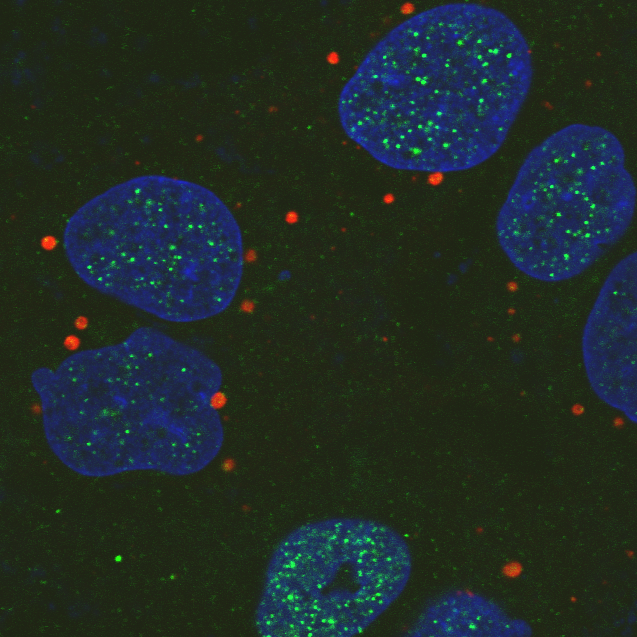

Supplement: Supplementary file 17 — Source Data for Figure 3 [file EMBJ-42-e113349-s008.zip › EMBOJ-2022-113349_SourceDataForFigure 3/3B/3C_S403,p62_MRT68921_merged.bmp]

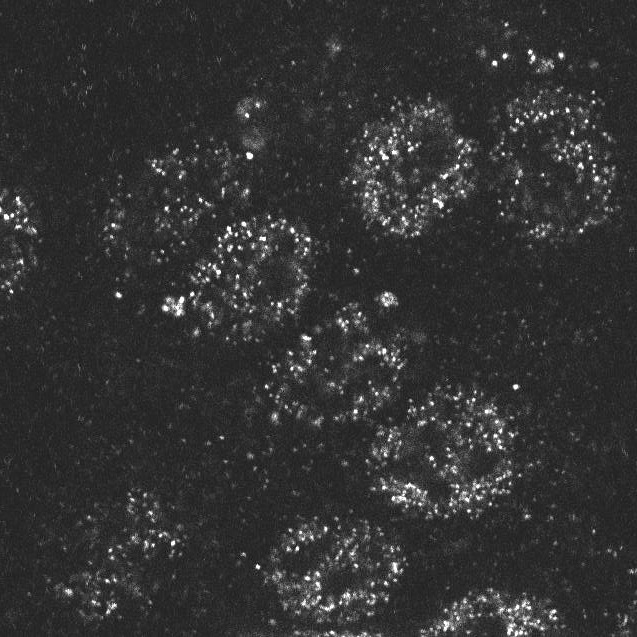

Supplement: Supplementary file 17 — Source Data for Figure 3 [file EMBJ-42-e113349-s008.zip › EMBOJ-2022-113349_SourceDataForFigure 3/3B/3B_S403,p62_DMSO_S403.jpg]

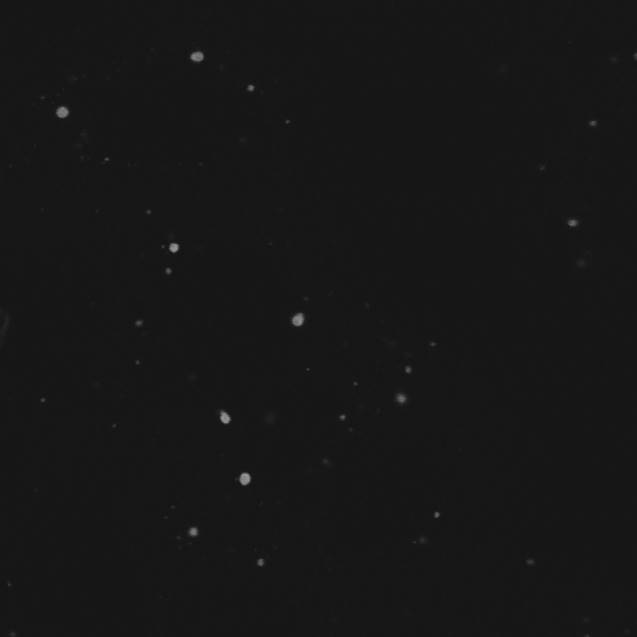

Supplement: Supplementary file 17 — Source Data for Figure 3 [file EMBJ-42-e113349-s008.zip › EMBOJ-2022-113349_SourceDataForFigure 3/3C/3C_pS349p62_MRT68921_pS349.jpg]

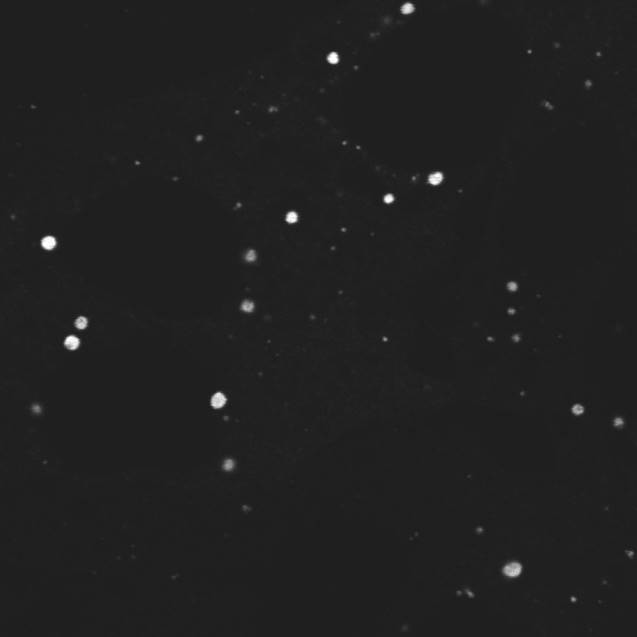

Supplement: Supplementary file 17 — Source Data for Figure 3 [file EMBJ-42-e113349-s008.zip › EMBOJ-2022-113349_SourceDataForFigure 3/3B/3C_S403,p62_MRT68921_p62.jpg]

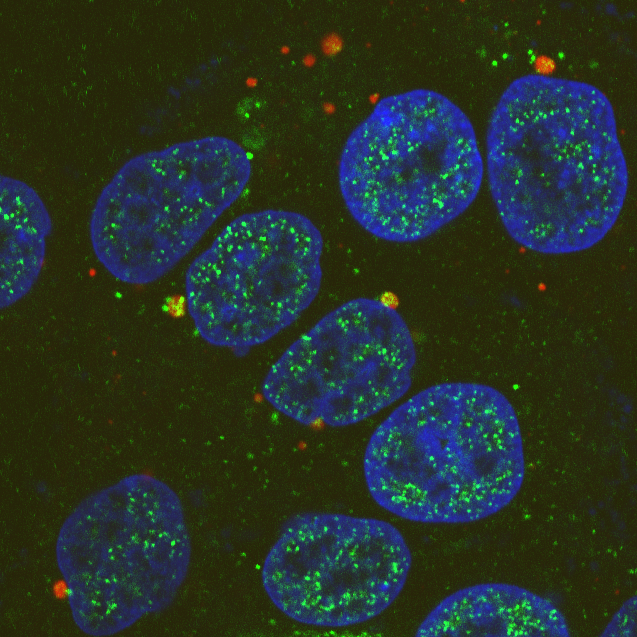

Supplement: Supplementary file 17 — Source Data for Figure 3 [file EMBJ-42-e113349-s008.zip › EMBOJ-2022-113349_SourceDataForFigure 3/3B/3B_S403,p62_DMSO_merged.bmp]

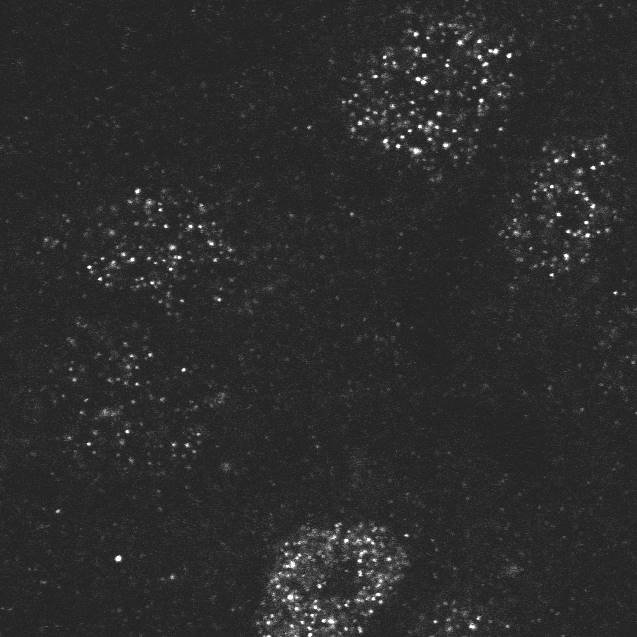

Supplement: Supplementary file 17 — Source Data for Figure 3 [file EMBJ-42-e113349-s008.zip › EMBOJ-2022-113349_SourceDataForFigure 3/3B/3C_S403,p62_MRT68921_S403.jpg]

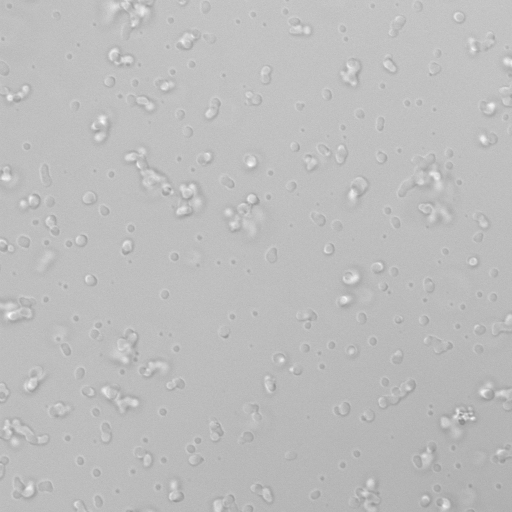

Supplement: Supplementary file 18 — Source Data for Figure 4 [file EMBJ-42-e113349-s019.zip › EMBOJ-2022-113349_SourceDataForFigure 4/4D/4D_Keap1-p62WT (5).tif]

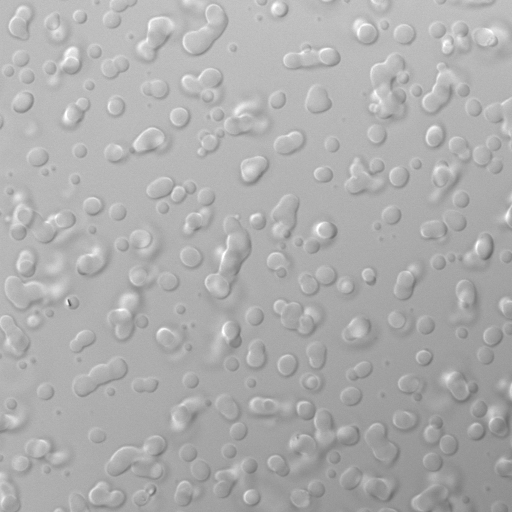

Supplement: Supplementary file 18 — Source Data for Figure 4 [file EMBJ-42-e113349-s019.zip › EMBOJ-2022-113349_SourceDataForFigure 4/4D/4D_Keap1-p62S403E S407E (5).tif]

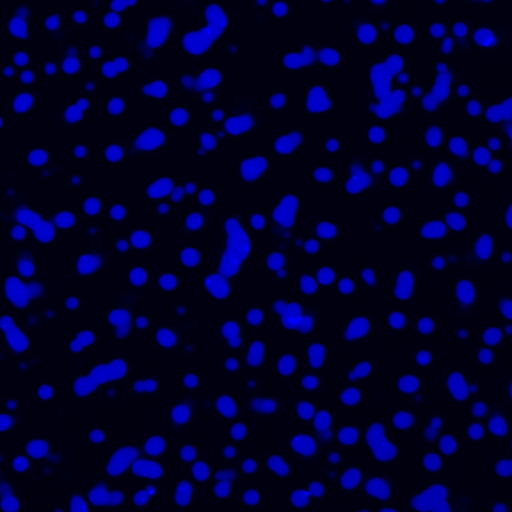

Supplement: Supplementary file 18 — Source Data for Figure 4 [file EMBJ-42-e113349-s019.zip › EMBOJ-2022-113349_SourceDataForFigure 4/4D/4D_Keap1-p62S403E S407E (3).tif]

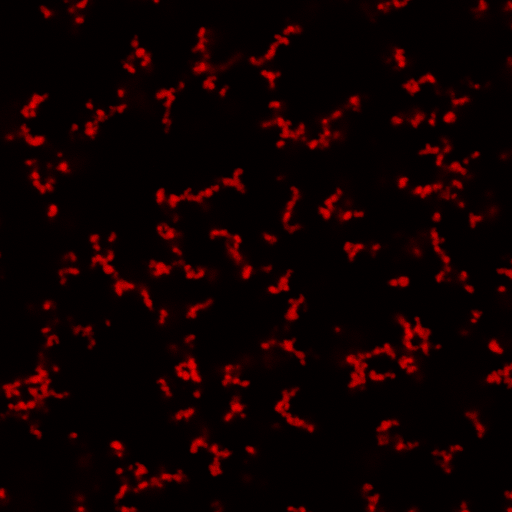

Supplement: Supplementary file 18 — Source Data for Figure 4 [file EMBJ-42-e113349-s019.zip › EMBOJ-2022-113349_SourceDataForFigure 4/4D/4D_Keap1-p62S349E (2).tif]

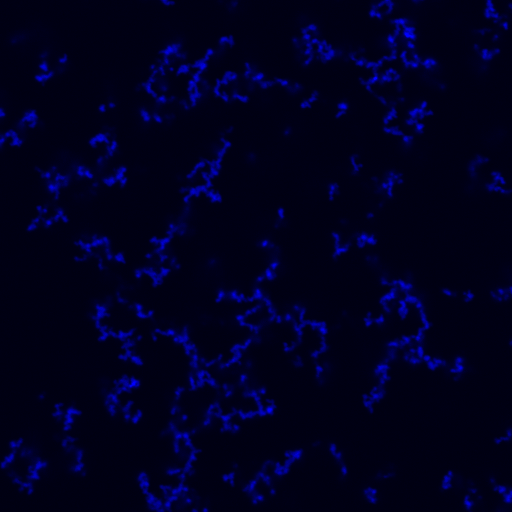

Supplement: Supplementary file 18 — Source Data for Figure 4 [file EMBJ-42-e113349-s019.zip › EMBOJ-2022-113349_SourceDataForFigure 4/4D/4D_Keap1-p62S349E S403E S407E (3).tif]

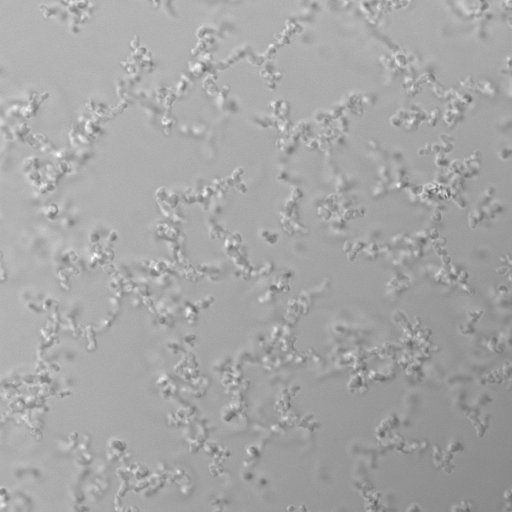

Supplement: Supplementary file 18 — Source Data for Figure 4 [file EMBJ-42-e113349-s019.zip › EMBOJ-2022-113349_SourceDataForFigure 4/4D/4D_Keap1-p62S349E (5).tif]

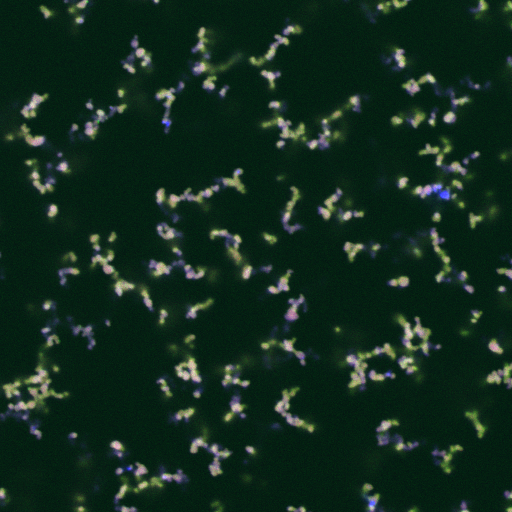

Supplement: Supplementary file 18 — Source Data for Figure 4 [file EMBJ-42-e113349-s019.zip › EMBOJ-2022-113349_SourceDataForFigure 4/4D/4D_Keap1-p62S349E (4).tif]

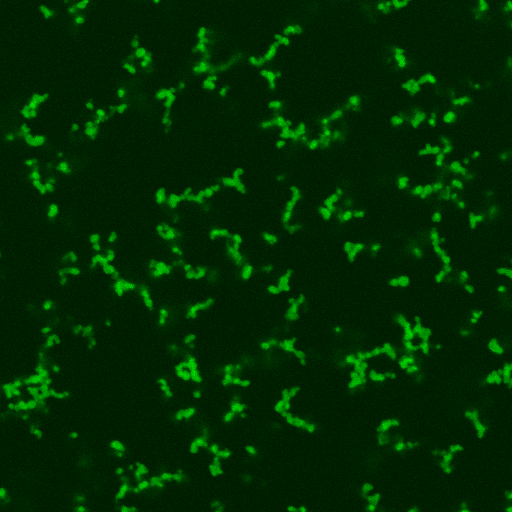

Supplement: Supplementary file 18 — Source Data for Figure 4 [file EMBJ-42-e113349-s019.zip › EMBOJ-2022-113349_SourceDataForFigure 4/4D/4D_Keap1-p62S349E (1).tif]

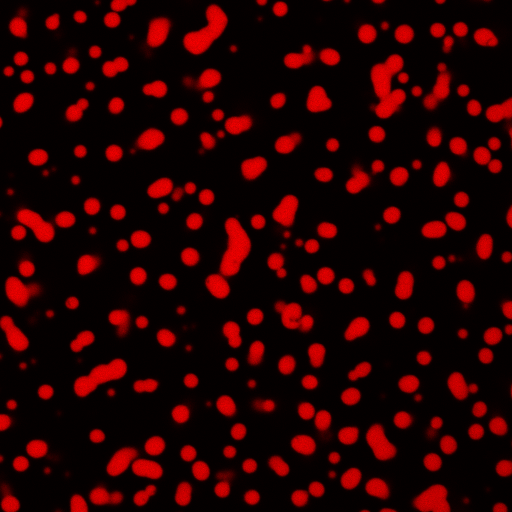

Supplement: Supplementary file 18 — Source Data for Figure 4 [file EMBJ-42-e113349-s019.zip › EMBOJ-2022-113349_SourceDataForFigure 4/4D/4D_Keap1-p62S403E S407E (2).tif]

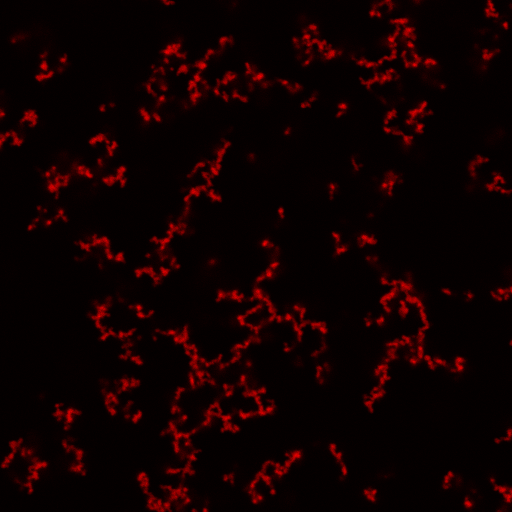

Supplement: Supplementary file 18 — Source Data for Figure 4 [file EMBJ-42-e113349-s019.zip › EMBOJ-2022-113349_SourceDataForFigure 4/4D/4D_Keap1-p62S349E S403E S407E (2).tif]

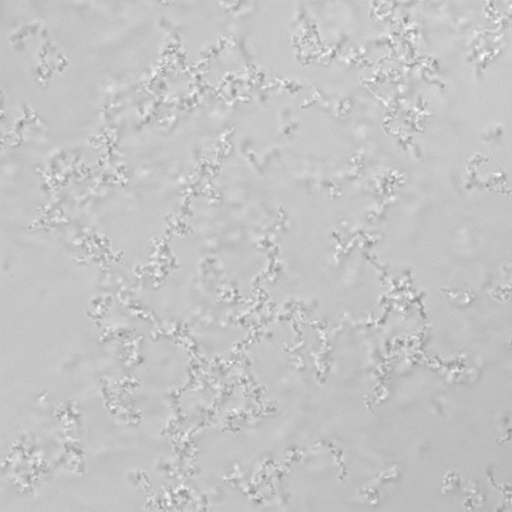

Supplement: Supplementary file 18 — Source Data for Figure 4 [file EMBJ-42-e113349-s019.zip › EMBOJ-2022-113349_SourceDataForFigure 4/4D/4D_Keap1-p62S349E S403E S407E (5).tif]

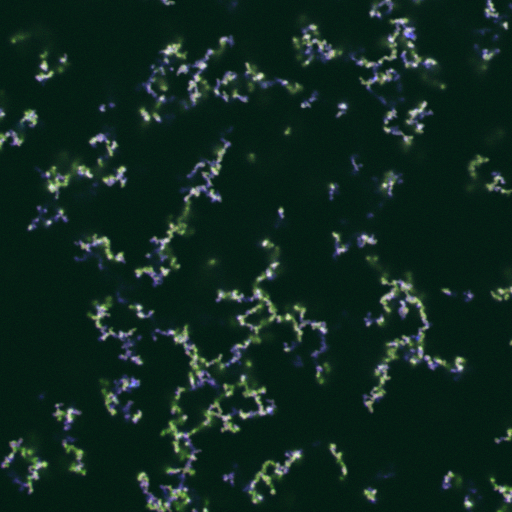

Supplement: Supplementary file 18 — Source Data for Figure 4 [file EMBJ-42-e113349-s019.zip › EMBOJ-2022-113349_SourceDataForFigure 4/4D/4D_Keap1-p62S349E S403E S407E (4).tif]

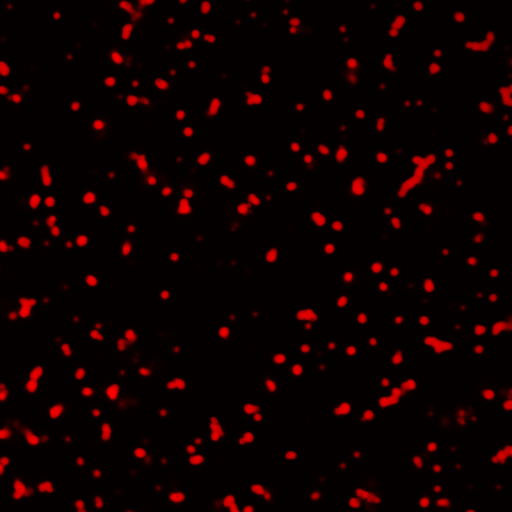

Supplement: Supplementary file 18 — Source Data for Figure 4 [file EMBJ-42-e113349-s019.zip › EMBOJ-2022-113349_SourceDataForFigure 4/4D/4D_Keap1-p62WT (2).tif]

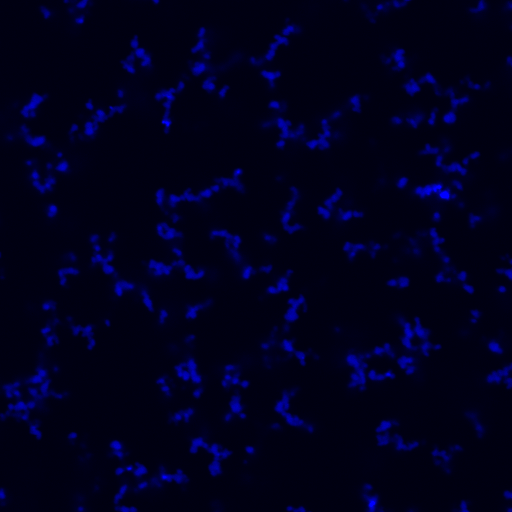

Supplement: Supplementary file 18 — Source Data for Figure 4 [file EMBJ-42-e113349-s019.zip › EMBOJ-2022-113349_SourceDataForFigure 4/4D/4D_Keap1-p62S349E (3).tif]
