# Supplementary figures and images for: Phosphorylation of phase‐separated p62 bodies by ULK1 activates a redox‐independent stress response (part 1 of 3)
Source: EMBO J. 2023 Jun 12;42(14):e113349. doi: 10.15252/embj.2022113349 (PMC10350833; doi:10.15252/embj.2022113349)

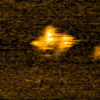

Supplement: Supplementary file 15 — Source Data for Figure 1 [file EMBJ-42-e113349-s003.zip › EMBOJ-2022-113349_SourceDataForFigure 1/1D/1D (97).bmp]

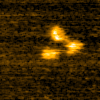

Supplement: Supplementary file 15 — Source Data for Figure 1 [file EMBJ-42-e113349-s003.zip › EMBOJ-2022-113349_SourceDataForFigure 1/1D/1D (79).bmp]

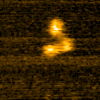

Supplement: Supplementary file 15 — Source Data for Figure 1 [file EMBJ-42-e113349-s003.zip › EMBOJ-2022-113349_SourceDataForFigure 1/1D/1D (75).bmp]

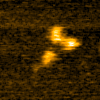

Supplement: Supplementary file 15 — Source Data for Figure 1 [file EMBJ-42-e113349-s003.zip › EMBOJ-2022-113349_SourceDataForFigure 1/1D/1D (81).bmp]

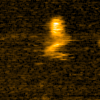

Supplement: Supplementary file 15 — Source Data for Figure 1 [file EMBJ-42-e113349-s003.zip › EMBOJ-2022-113349_SourceDataForFigure 1/1D/1D (58).bmp]

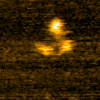

Supplement: Supplementary file 15 — Source Data for Figure 1 [file EMBJ-42-e113349-s003.zip › EMBOJ-2022-113349_SourceDataForFigure 1/1D/1D (74).bmp]

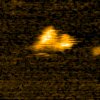

Supplement: Supplementary file 15 — Source Data for Figure 1 [file EMBJ-42-e113349-s003.zip › EMBOJ-2022-113349_SourceDataForFigure 1/1D/1D (96).bmp]

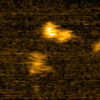

Supplement: Supplementary file 15 — Source Data for Figure 1 [file EMBJ-42-e113349-s003.zip › EMBOJ-2022-113349_SourceDataForFigure 1/1D/1D (6).bmp]

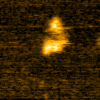

Supplement: Supplementary file 15 — Source Data for Figure 1 [file EMBJ-42-e113349-s003.zip › EMBOJ-2022-113349_SourceDataForFigure 1/1D/1D (62).bmp]

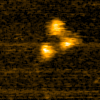

Supplement: Supplementary file 15 — Source Data for Figure 1 [file EMBJ-42-e113349-s003.zip › EMBOJ-2022-113349_SourceDataForFigure 1/1D/1D (78).bmp]

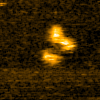

Supplement: Supplementary file 15 — Source Data for Figure 1 [file EMBJ-42-e113349-s003.zip › EMBOJ-2022-113349_SourceDataForFigure 1/1D/1D (80).bmp]

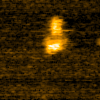

Supplement: Supplementary file 15 — Source Data for Figure 1 [file EMBJ-42-e113349-s003.zip › EMBOJ-2022-113349_SourceDataForFigure 1/1D/1D (59).bmp]

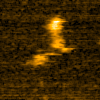

Supplement: Supplementary file 15 — Source Data for Figure 1 [file EMBJ-42-e113349-s003.zip › EMBOJ-2022-113349_SourceDataForFigure 1/1D/1D (54).bmp]

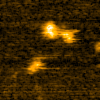

Supplement: Supplementary file 15 — Source Data for Figure 1 [file EMBJ-42-e113349-s003.zip › EMBOJ-2022-113349_SourceDataForFigure 1/1D/1D (7).bmp]

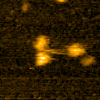

Supplement: Supplementary file 15 — Source Data for Figure 1 [file EMBJ-42-e113349-s003.zip › EMBOJ-2022-113349_SourceDataForFigure 1/1D/1D (348).bmp]

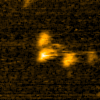

Supplement: Supplementary file 15 — Source Data for Figure 1 [file EMBJ-42-e113349-s003.zip › EMBOJ-2022-113349_SourceDataForFigure 1/1D/1D (332).bmp]

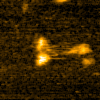

Supplement: Supplementary file 15 — Source Data for Figure 1 [file EMBJ-42-e113349-s003.zip › EMBOJ-2022-113349_SourceDataForFigure 1/1D/1D (349).bmp]

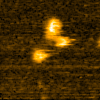

Supplement: Supplementary file 15 — Source Data for Figure 1 [file EMBJ-42-e113349-s003.zip › EMBOJ-2022-113349_SourceDataForFigure 1/1D/1D (39).bmp]

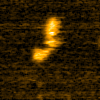

Supplement: Supplementary file 15 — Source Data for Figure 1 [file EMBJ-42-e113349-s003.zip › EMBOJ-2022-113349_SourceDataForFigure 1/1D/1D (34).bmp]

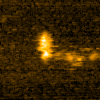

Supplement: Supplementary file 15 — Source Data for Figure 1 [file EMBJ-42-e113349-s003.zip › EMBOJ-2022-113349_SourceDataForFigure 1/1D/1D (313).bmp]

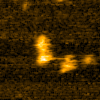

Supplement: Supplementary file 15 — Source Data for Figure 1 [file EMBJ-42-e113349-s003.zip › EMBOJ-2022-113349_SourceDataForFigure 1/1D/1D (345).bmp]

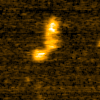

Supplement: Supplementary file 15 — Source Data for Figure 1 [file EMBJ-42-e113349-s003.zip › EMBOJ-2022-113349_SourceDataForFigure 1/1D/1D (33).bmp]

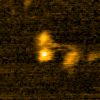

Supplement: Supplementary file 15 — Source Data for Figure 1 [file EMBJ-42-e113349-s003.zip › EMBOJ-2022-113349_SourceDataForFigure 1/1D/1D (333).bmp]

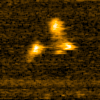

Supplement: Supplementary file 15 — Source Data for Figure 1 [file EMBJ-42-e113349-s003.zip › EMBOJ-2022-113349_SourceDataForFigure 1/1D/1D (72).bmp]

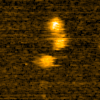

Supplement: Supplementary file 15 — Source Data for Figure 1 [file EMBJ-42-e113349-s003.zip › EMBOJ-2022-113349_SourceDataForFigure 1/1D/1D (55).bmp]

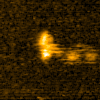

Supplement: Supplementary file 15 — Source Data for Figure 1 [file EMBJ-42-e113349-s003.zip › EMBOJ-2022-113349_SourceDataForFigure 1/1D/1D (312).bmp]

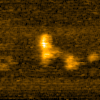

Supplement: Supplementary file 15 — Source Data for Figure 1 [file EMBJ-42-e113349-s003.zip › EMBOJ-2022-113349_SourceDataForFigure 1/1D/1D (319).bmp]

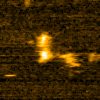

Supplement: Supplementary file 15 — Source Data for Figure 1 [file EMBJ-42-e113349-s003.zip › EMBOJ-2022-113349_SourceDataForFigure 1/1D/1D (324).bmp]

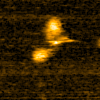

Supplement: Supplementary file 15 — Source Data for Figure 1 [file EMBJ-42-e113349-s003.zip › EMBOJ-2022-113349_SourceDataForFigure 1/1D/1D (38).bmp]

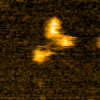

Supplement: Supplementary file 15 — Source Data for Figure 1 [file EMBJ-42-e113349-s003.zip › EMBOJ-2022-113349_SourceDataForFigure 1/1D/1D (43).bmp]

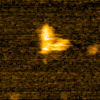

Supplement: Supplementary file 15 — Source Data for Figure 1 [file EMBJ-42-e113349-s003.zip › EMBOJ-2022-113349_SourceDataForFigure 1/1D/1D (289).bmp]

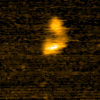

Supplement: Supplementary file 15 — Source Data for Figure 1 [file EMBJ-42-e113349-s003.zip › EMBOJ-2022-113349_SourceDataForFigure 1/1D/1D (63).bmp]

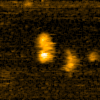

Supplement: Supplementary file 15 — Source Data for Figure 1 [file EMBJ-42-e113349-s003.zip › EMBOJ-2022-113349_SourceDataForFigure 1/1D/1D (328).bmp]

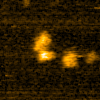

Supplement: Supplementary file 15 — Source Data for Figure 1 [file EMBJ-42-e113349-s003.zip › EMBOJ-2022-113349_SourceDataForFigure 1/1D/1D (335).bmp]

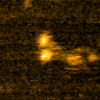

Supplement: Supplementary file 15 — Source Data for Figure 1 [file EMBJ-42-e113349-s003.zip › EMBOJ-2022-113349_SourceDataForFigure 1/1D/1D (323).bmp]

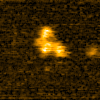

Supplement: Supplementary file 15 — Source Data for Figure 1 [file EMBJ-42-e113349-s003.zip › EMBOJ-2022-113349_SourceDataForFigure 1/1D/1D (305).bmp]

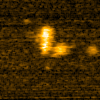

Supplement: Supplementary file 15 — Source Data for Figure 1 [file EMBJ-42-e113349-s003.zip › EMBOJ-2022-113349_SourceDataForFigure 1/1D/1D (299).bmp]

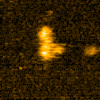

Supplement: Supplementary file 15 — Source Data for Figure 1 [file EMBJ-42-e113349-s003.zip › EMBOJ-2022-113349_SourceDataForFigure 1/1D/1D (261).bmp]

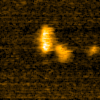

Supplement: Supplementary file 15 — Source Data for Figure 1 [file EMBJ-42-e113349-s003.zip › EMBOJ-2022-113349_SourceDataForFigure 1/1D/1D (298).bmp]

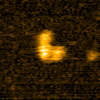

Supplement: Supplementary file 15 — Source Data for Figure 1 [file EMBJ-42-e113349-s003.zip › EMBOJ-2022-113349_SourceDataForFigure 1/1D/1D (309).bmp]

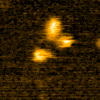

Supplement: Supplementary file 15 — Source Data for Figure 1 [file EMBJ-42-e113349-s003.zip › EMBOJ-2022-113349_SourceDataForFigure 1/1D/1D (42).bmp]

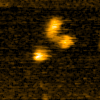

Supplement: Supplementary file 15 — Source Data for Figure 1 [file EMBJ-42-e113349-s003.zip › EMBOJ-2022-113349_SourceDataForFigure 1/1D/1D (48).bmp]

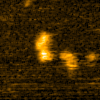

Supplement: Supplementary file 15 — Source Data for Figure 1 [file EMBJ-42-e113349-s003.zip › EMBOJ-2022-113349_SourceDataForFigure 1/1D/1D (329).bmp]

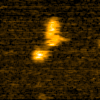

Supplement: Supplementary file 15 — Source Data for Figure 1 [file EMBJ-42-e113349-s003.zip › EMBOJ-2022-113349_SourceDataForFigure 1/1D/1D (35).bmp]

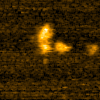

Supplement: Supplementary file 15 — Source Data for Figure 1 [file EMBJ-42-e113349-s003.zip › EMBOJ-2022-113349_SourceDataForFigure 1/1D/1D (282).bmp]

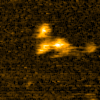

Supplement: Supplementary file 15 — Source Data for Figure 1 [file EMBJ-42-e113349-s003.zip › EMBOJ-2022-113349_SourceDataForFigure 1/1D/1D (241).bmp]

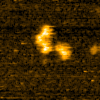

Supplement: Supplementary file 15 — Source Data for Figure 1 [file EMBJ-42-e113349-s003.zip › EMBOJ-2022-113349_SourceDataForFigure 1/1D/1D (294).bmp]

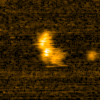

Supplement: Supplementary file 15 — Source Data for Figure 1 [file EMBJ-42-e113349-s003.zip › EMBOJ-2022-113349_SourceDataForFigure 1/1D/1D (308).bmp]

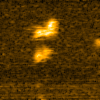

Supplement: Supplementary file 15 — Source Data for Figure 1 [file EMBJ-42-e113349-s003.zip › EMBOJ-2022-113349_SourceDataForFigure 1/1D/1D (23).bmp]

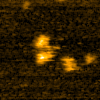

Supplement: Supplementary file 15 — Source Data for Figure 1 [file EMBJ-42-e113349-s003.zip › EMBOJ-2022-113349_SourceDataForFigure 1/1D/1D (344).bmp]

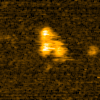

Supplement: Supplementary file 15 — Source Data for Figure 1 [file EMBJ-42-e113349-s003.zip › EMBOJ-2022-113349_SourceDataForFigure 1/1D/1D (304).bmp]

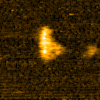

Supplement: Supplementary file 15 — Source Data for Figure 1 [file EMBJ-42-e113349-s003.zip › EMBOJ-2022-113349_SourceDataForFigure 1/1D/1D (260).bmp]

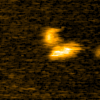

Supplement: Supplementary file 15 — Source Data for Figure 1 [file EMBJ-42-e113349-s003.zip › EMBOJ-2022-113349_SourceDataForFigure 1/1D/1D (200).bmp]

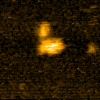

Supplement: Supplementary file 15 — Source Data for Figure 1 [file EMBJ-42-e113349-s003.zip › EMBOJ-2022-113349_SourceDataForFigure 1/1D/1D (240).bmp]

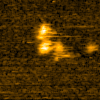

Supplement: Supplementary file 15 — Source Data for Figure 1 [file EMBJ-42-e113349-s003.zip › EMBOJ-2022-113349_SourceDataForFigure 1/1D/1D (231).bmp]

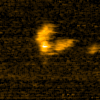

Supplement: Supplementary file 15 — Source Data for Figure 1 [file EMBJ-42-e113349-s003.zip › EMBOJ-2022-113349_SourceDataForFigure 1/1D/1D (283).bmp]

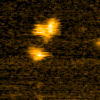

Supplement: Supplementary file 15 — Source Data for Figure 1 [file EMBJ-42-e113349-s003.zip › EMBOJ-2022-113349_SourceDataForFigure 1/1D/1D (25).bmp]

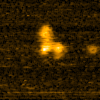

Supplement: Supplementary file 15 — Source Data for Figure 1 [file EMBJ-42-e113349-s003.zip › EMBOJ-2022-113349_SourceDataForFigure 1/1D/1D (277).bmp]

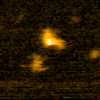

Supplement: Supplementary file 15 — Source Data for Figure 1 [file EMBJ-42-e113349-s003.zip › EMBOJ-2022-113349_SourceDataForFigure 1/1D/1D (165).bmp]

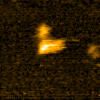

Supplement: Supplementary file 15 — Source Data for Figure 1 [file EMBJ-42-e113349-s003.zip › EMBOJ-2022-113349_SourceDataForFigure 1/1D/1D (236).bmp]

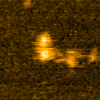

Supplement: Supplementary file 15 — Source Data for Figure 1 [file EMBJ-42-e113349-s003.zip › EMBOJ-2022-113349_SourceDataForFigure 1/1D/1D (325).bmp]

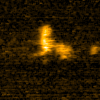

Supplement: Supplementary file 15 — Source Data for Figure 1 [file EMBJ-42-e113349-s003.zip › EMBOJ-2022-113349_SourceDataForFigure 1/1D/1D (295).bmp]

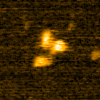

Supplement: Supplementary file 15 — Source Data for Figure 1 [file EMBJ-42-e113349-s003.zip › EMBOJ-2022-113349_SourceDataForFigure 1/1D/1D (159).bmp]

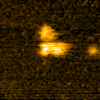

Supplement: Supplementary file 15 — Source Data for Figure 1 [file EMBJ-42-e113349-s003.zip › EMBOJ-2022-113349_SourceDataForFigure 1/1D/1D (270).bmp]

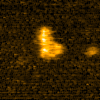

Supplement: Supplementary file 15 — Source Data for Figure 1 [file EMBJ-42-e113349-s003.zip › EMBOJ-2022-113349_SourceDataForFigure 1/1D/1D (257).bmp]

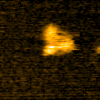

Supplement: Supplementary file 15 — Source Data for Figure 1 [file EMBJ-42-e113349-s003.zip › EMBOJ-2022-113349_SourceDataForFigure 1/1D/1D (220).bmp]

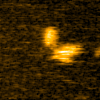

Supplement: Supplementary file 15 — Source Data for Figure 1 [file EMBJ-42-e113349-s003.zip › EMBOJ-2022-113349_SourceDataForFigure 1/1D/1D (201).bmp]

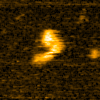

Supplement: Supplementary file 15 — Source Data for Figure 1 [file EMBJ-42-e113349-s003.zip › EMBOJ-2022-113349_SourceDataForFigure 1/1D/1D (163).bmp]

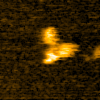

Supplement: Supplementary file 15 — Source Data for Figure 1 [file EMBJ-42-e113349-s003.zip › EMBOJ-2022-113349_SourceDataForFigure 1/1D/1D (187).bmp]

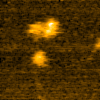

Supplement: Supplementary file 15 — Source Data for Figure 1 [file EMBJ-42-e113349-s003.zip › EMBOJ-2022-113349_SourceDataForFigure 1/1D/1D (22).bmp]

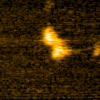

Supplement: Supplementary file 15 — Source Data for Figure 1 [file EMBJ-42-e113349-s003.zip › EMBOJ-2022-113349_SourceDataForFigure 1/1D/1D (173).bmp]

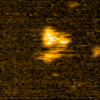

Supplement: Supplementary file 15 — Source Data for Figure 1 [file EMBJ-42-e113349-s003.zip › EMBOJ-2022-113349_SourceDataForFigure 1/1D/1D (169).bmp]

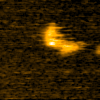

Supplement: Supplementary file 15 — Source Data for Figure 1 [file EMBJ-42-e113349-s003.zip › EMBOJ-2022-113349_SourceDataForFigure 1/1D/1D (217).bmp]

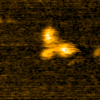

Supplement: Supplementary file 15 — Source Data for Figure 1 [file EMBJ-42-e113349-s003.zip › EMBOJ-2022-113349_SourceDataForFigure 1/1D/1D (186).bmp]

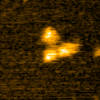

Supplement: Supplementary file 15 — Source Data for Figure 1 [file EMBJ-42-e113349-s003.zip › EMBOJ-2022-113349_SourceDataForFigure 1/1D/1D (190).bmp]

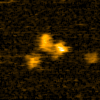

Supplement: Supplementary file 15 — Source Data for Figure 1 [file EMBJ-42-e113349-s003.zip › EMBOJ-2022-113349_SourceDataForFigure 1/1D/1D (145).bmp]

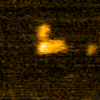

Supplement: Supplementary file 15 — Source Data for Figure 1 [file EMBJ-42-e113349-s003.zip › EMBOJ-2022-113349_SourceDataForFigure 1/1D/1D (237).bmp]

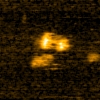

Supplement: Supplementary file 15 — Source Data for Figure 1 [file EMBJ-42-e113349-s003.zip › EMBOJ-2022-113349_SourceDataForFigure 1/1D/1D (153).bmp]

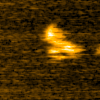

Supplement: Supplementary file 15 — Source Data for Figure 1 [file EMBJ-42-e113349-s003.zip › EMBOJ-2022-113349_SourceDataForFigure 1/1D/1D (216).bmp]

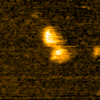

Supplement: Supplementary file 15 — Source Data for Figure 1 [file EMBJ-42-e113349-s003.zip › EMBOJ-2022-113349_SourceDataForFigure 1/1D/1D (172).bmp]

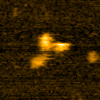

Supplement: Supplementary file 15 — Source Data for Figure 1 [file EMBJ-42-e113349-s003.zip › EMBOJ-2022-113349_SourceDataForFigure 1/1D/1D (149).bmp]

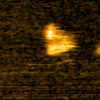

Supplement: Supplementary file 15 — Source Data for Figure 1 [file EMBJ-42-e113349-s003.zip › EMBOJ-2022-113349_SourceDataForFigure 1/1D/1D (221).bmp]

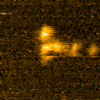

Supplement: Supplementary file 15 — Source Data for Figure 1 [file EMBJ-42-e113349-s003.zip › EMBOJ-2022-113349_SourceDataForFigure 1/1D/1D (266).bmp]

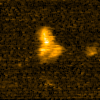

Supplement: Supplementary file 15 — Source Data for Figure 1 [file EMBJ-42-e113349-s003.zip › EMBOJ-2022-113349_SourceDataForFigure 1/1D/1D (256).bmp]

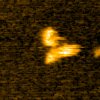

Supplement: Supplementary file 15 — Source Data for Figure 1 [file EMBJ-42-e113349-s003.zip › EMBOJ-2022-113349_SourceDataForFigure 1/1D/1D (191).bmp]

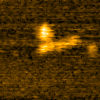

Supplement: Supplementary file 15 — Source Data for Figure 1 [file EMBJ-42-e113349-s003.zip › EMBOJ-2022-113349_SourceDataForFigure 1/1D/1D (227).bmp]

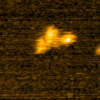

Supplement: Supplementary file 15 — Source Data for Figure 1 [file EMBJ-42-e113349-s003.zip › EMBOJ-2022-113349_SourceDataForFigure 1/1D/1D (108).bmp]

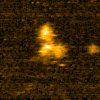

Supplement: Supplementary file 15 — Source Data for Figure 1 [file EMBJ-42-e113349-s003.zip › EMBOJ-2022-113349_SourceDataForFigure 1/1D/1D (276).bmp]

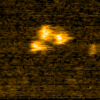

Supplement: Supplementary file 15 — Source Data for Figure 1 [file EMBJ-42-e113349-s003.zip › EMBOJ-2022-113349_SourceDataForFigure 1/1D/1D (125).bmp]

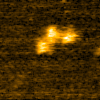

Supplement: Supplementary file 15 — Source Data for Figure 1 [file EMBJ-42-e113349-s003.zip › EMBOJ-2022-113349_SourceDataForFigure 1/1D/1D (124).bmp]

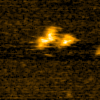

Supplement: Supplementary file 15 — Source Data for Figure 1 [file EMBJ-42-e113349-s003.zip › EMBOJ-2022-113349_SourceDataForFigure 1/1D/1D (105).bmp]

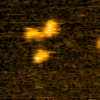

Supplement: Supplementary file 15 — Source Data for Figure 1 [file EMBJ-42-e113349-s003.zip › EMBOJ-2022-113349_SourceDataForFigure 1/1D/1D (18).bmp]

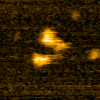

Supplement: Supplementary file 15 — Source Data for Figure 1 [file EMBJ-42-e113349-s003.zip › EMBOJ-2022-113349_SourceDataForFigure 1/1D/1D (164).bmp]

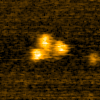

Supplement: Supplementary file 15 — Source Data for Figure 1 [file EMBJ-42-e113349-s003.zip › EMBOJ-2022-113349_SourceDataForFigure 1/1D/1D (133).bmp]

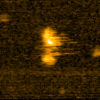

Supplement: Supplementary file 15 — Source Data for Figure 1 [file EMBJ-42-e113349-s003.zip › EMBOJ-2022-113349_SourceDataForFigure 1/1D/1D (168).bmp]

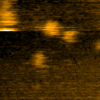

Supplement: Supplementary file 15 — Source Data for Figure 1 [file EMBJ-42-e113349-s003.zip › EMBOJ-2022-113349_SourceDataForFigure 1/1D/1D (14).bmp]

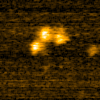

Supplement: Supplementary file 15 — Source Data for Figure 1 [file EMBJ-42-e113349-s003.zip › EMBOJ-2022-113349_SourceDataForFigure 1/1D/1D (129).bmp]

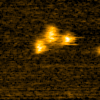

Supplement: Supplementary file 15 — Source Data for Figure 1 [file EMBJ-42-e113349-s003.zip › EMBOJ-2022-113349_SourceDataForFigure 1/1D/1D (112).bmp]

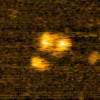

Supplement: Supplementary file 15 — Source Data for Figure 1 [file EMBJ-42-e113349-s003.zip › EMBOJ-2022-113349_SourceDataForFigure 1/1D/1D (152).bmp]

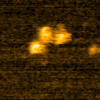

Supplement: Supplementary file 15 — Source Data for Figure 1 [file EMBJ-42-e113349-s003.zip › EMBOJ-2022-113349_SourceDataForFigure 1/1D/1D (128).bmp]
