# Supplementary material for: Phosphorylation of phase‐separated p62 bodies by ULK1 activates a redox‐independent stress response
Source: EMBO J. 2023 Jun 12;42(14):e113349. doi: 10.15252/embj.2022113349 (PMC10350833; doi:10.15252/embj.2022113349)
Supplement: Supplementary file 16 — Source Data for Figure 2 [file EMBJ-42-e113349-s016.zip › EMBOJ-2022-113349_SourceDataForFigure 2/2A/README_Fig 2A.docx]

Source files for Figure 2A

FV31S-SW software was used for generating the figure. Image size, 106.066 μm × 106.066 μm (512 × 512 pixel).
