# Supplementary material for: Phosphorylation of phase‐separated p62 bodies by ULK1 activates a redox‐independent stress response
Source: EMBO J. 2023 Jun 12;42(14):e113349. doi: 10.15252/embj.2022113349 (PMC10350833; doi:10.15252/embj.2022113349)
Supplement: Supplementary file 18 — Source Data for Figure 4 [file EMBJ-42-e113349-s019.zip › EMBOJ-2022-113349_SourceDataForFigure 4/4A/README_Fig 4A.docx]

Source files for Figure 4A

For high magnification pictures, cropped regions were used for generating the figure. Brightness/contrast of each figure was adjusted using the Photoshop software.
