# Supplementary figures and images for: Phosphorylation of phase‐separated p62 bodies by ULK1 activates a redox‐independent stress response (part 3 of 3)
Source: EMBO J. 2023 Jun 12;42(14):e113349. doi: 10.15252/embj.2022113349 (PMC10350833; doi:10.15252/embj.2022113349)

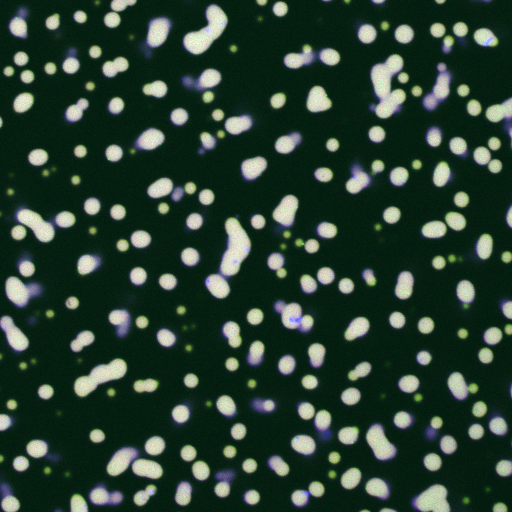

Supplement: Supplementary file 18 — Source Data for Figure 4 [file EMBJ-42-e113349-s019.zip › EMBOJ-2022-113349_SourceDataForFigure 4/4D/4D_Keap1-p62S403E S407E (4).tif]

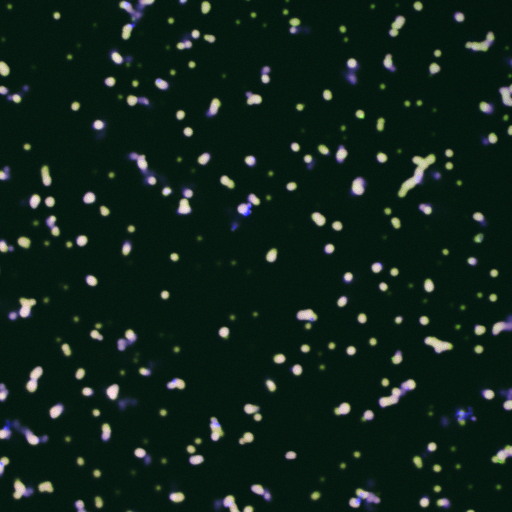

Supplement: Supplementary file 18 — Source Data for Figure 4 [file EMBJ-42-e113349-s019.zip › EMBOJ-2022-113349_SourceDataForFigure 4/4D/4D_Keap1-p62WT (4).tif]

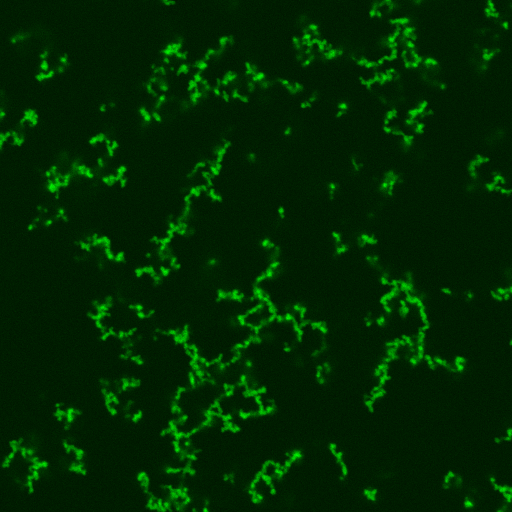

Supplement: Supplementary file 18 — Source Data for Figure 4 [file EMBJ-42-e113349-s019.zip › EMBOJ-2022-113349_SourceDataForFigure 4/4D/4D_Keap1-p62S349E S403E S407E (1).tif]

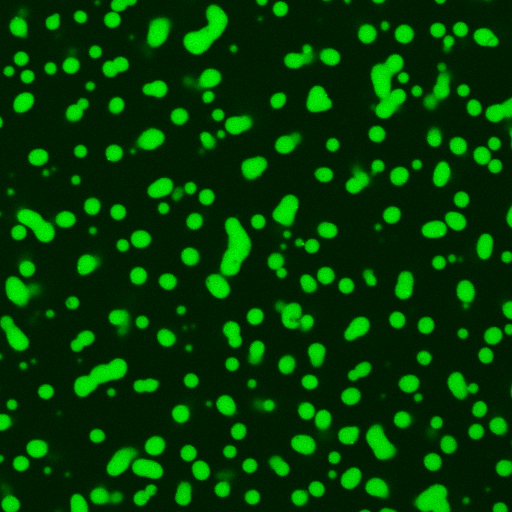

Supplement: Supplementary file 18 — Source Data for Figure 4 [file EMBJ-42-e113349-s019.zip › EMBOJ-2022-113349_SourceDataForFigure 4/4D/4D_Keap1-p62S403E S407E (1).tif]

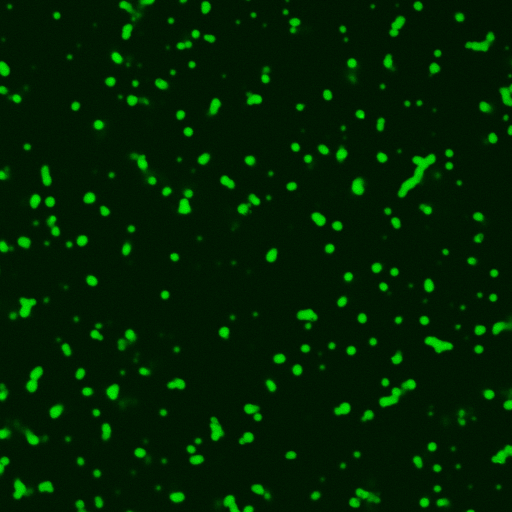

Supplement: Supplementary file 18 — Source Data for Figure 4 [file EMBJ-42-e113349-s019.zip › EMBOJ-2022-113349_SourceDataForFigure 4/4D/4D_Keap1-p62WT (1).tif]

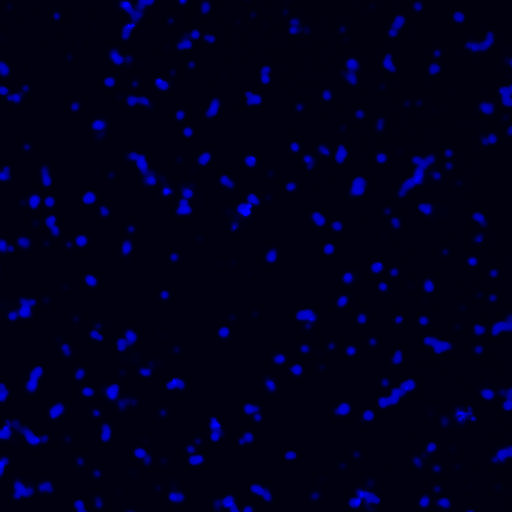

Supplement: Supplementary file 18 — Source Data for Figure 4 [file EMBJ-42-e113349-s019.zip › EMBOJ-2022-113349_SourceDataForFigure 4/4D/4D_Keap1-p62WT (3).tif]

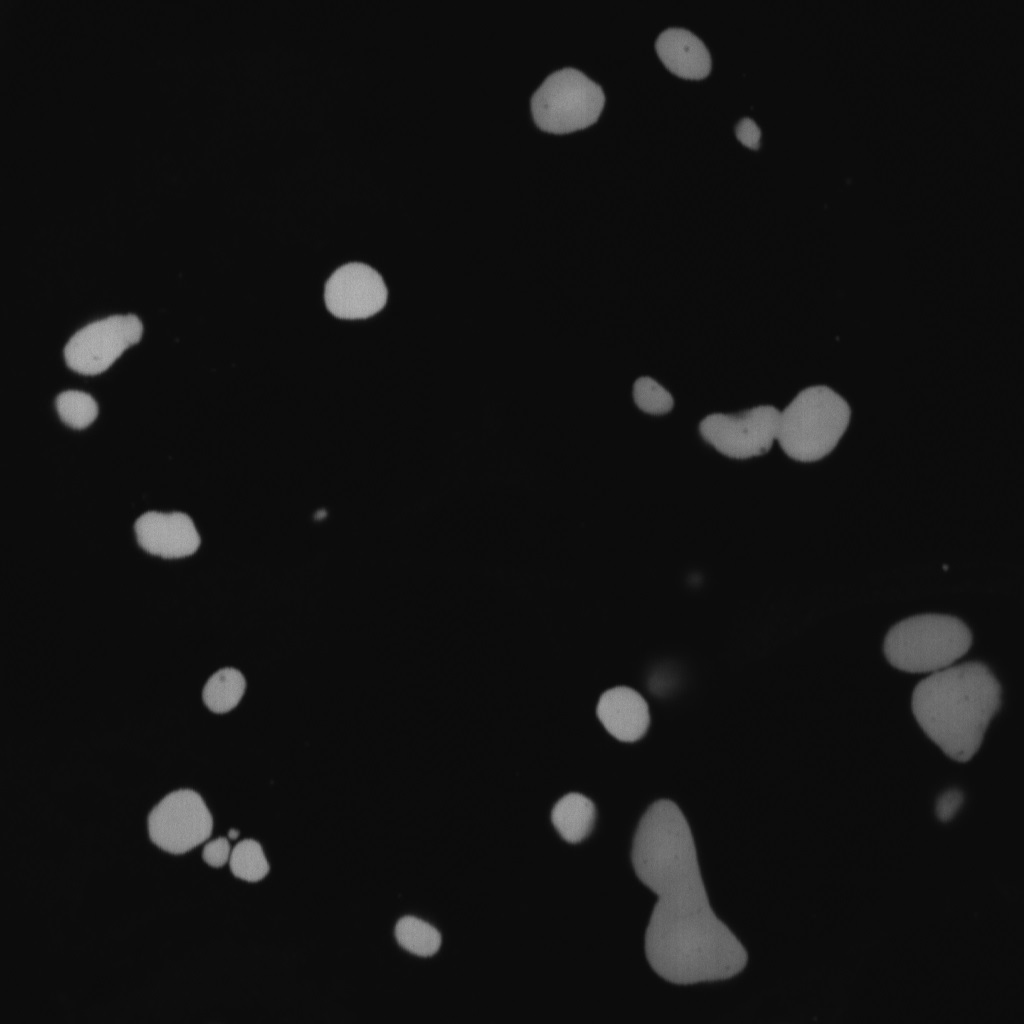

Supplement: Supplementary file 18 — Source Data for Figure 4 [file EMBJ-42-e113349-s019.zip › EMBOJ-2022-113349_SourceDataForFigure 4/4A/GFP-p62/4A (GFP-p62_mCherry-KEAP1)_GFP.jpg]

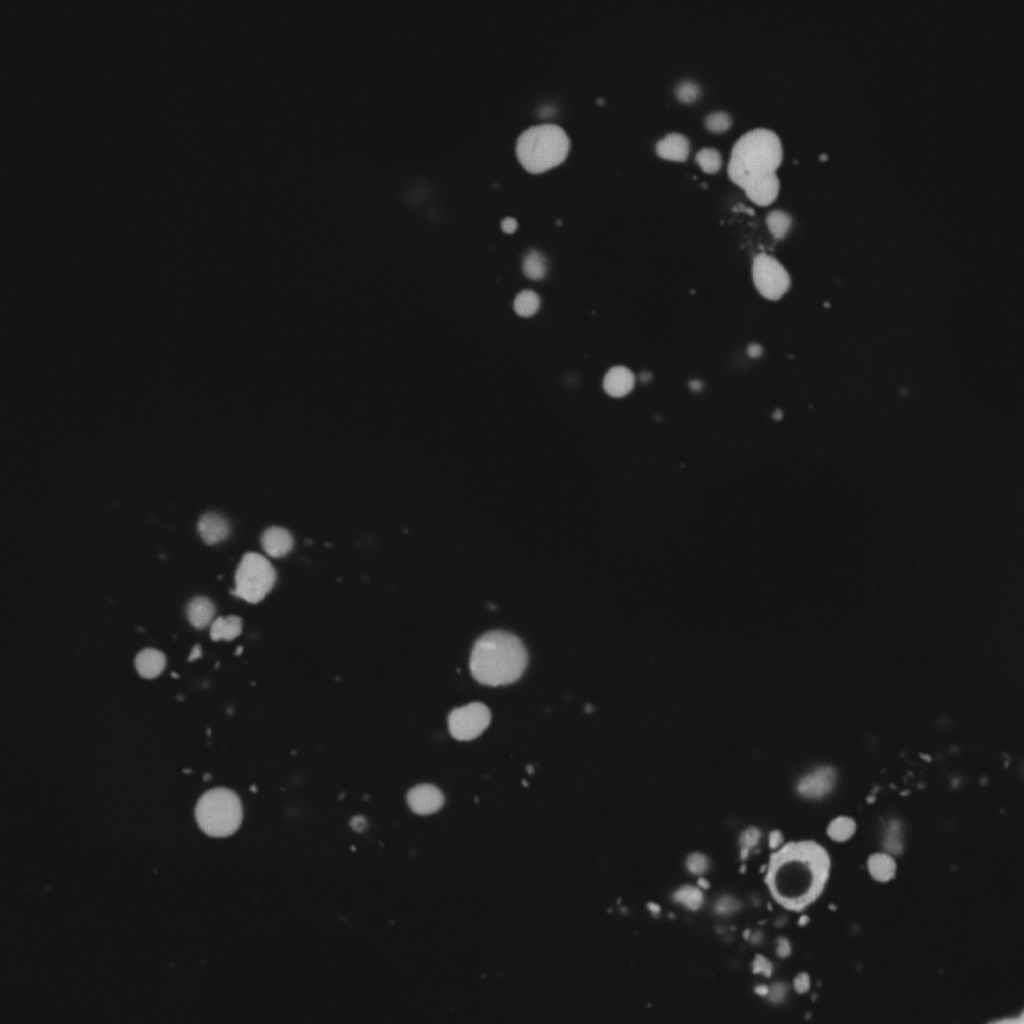

Supplement: Supplementary file 18 — Source Data for Figure 4 [file EMBJ-42-e113349-s019.zip › EMBOJ-2022-113349_SourceDataForFigure 4/4A/GFP-p62/4A (GFP-p62_mCherry)_GFP.jpg]

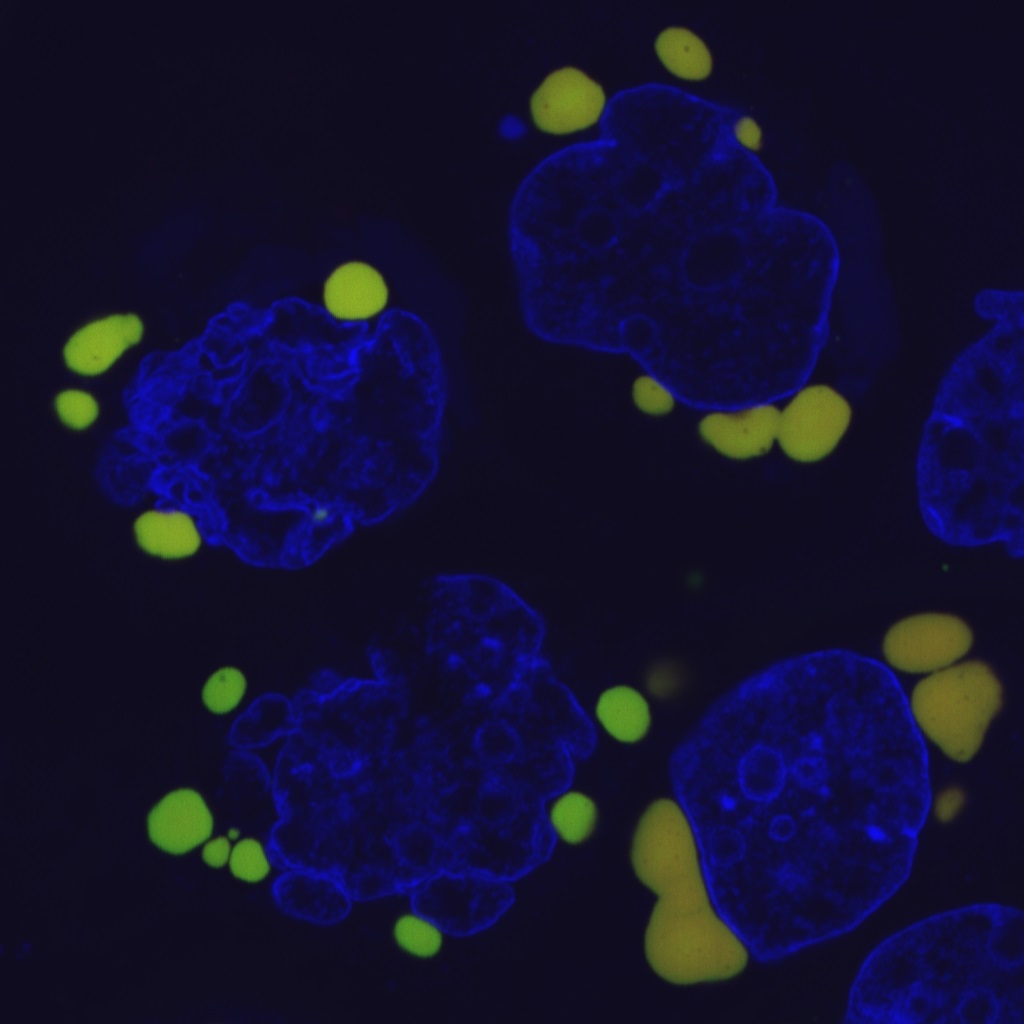

Supplement: Supplementary file 18 — Source Data for Figure 4 [file EMBJ-42-e113349-s019.zip › EMBOJ-2022-113349_SourceDataForFigure 4/4A/GFP-p62/4A (GFP-p62_mCherry-KEAP1)_merged.jpg]

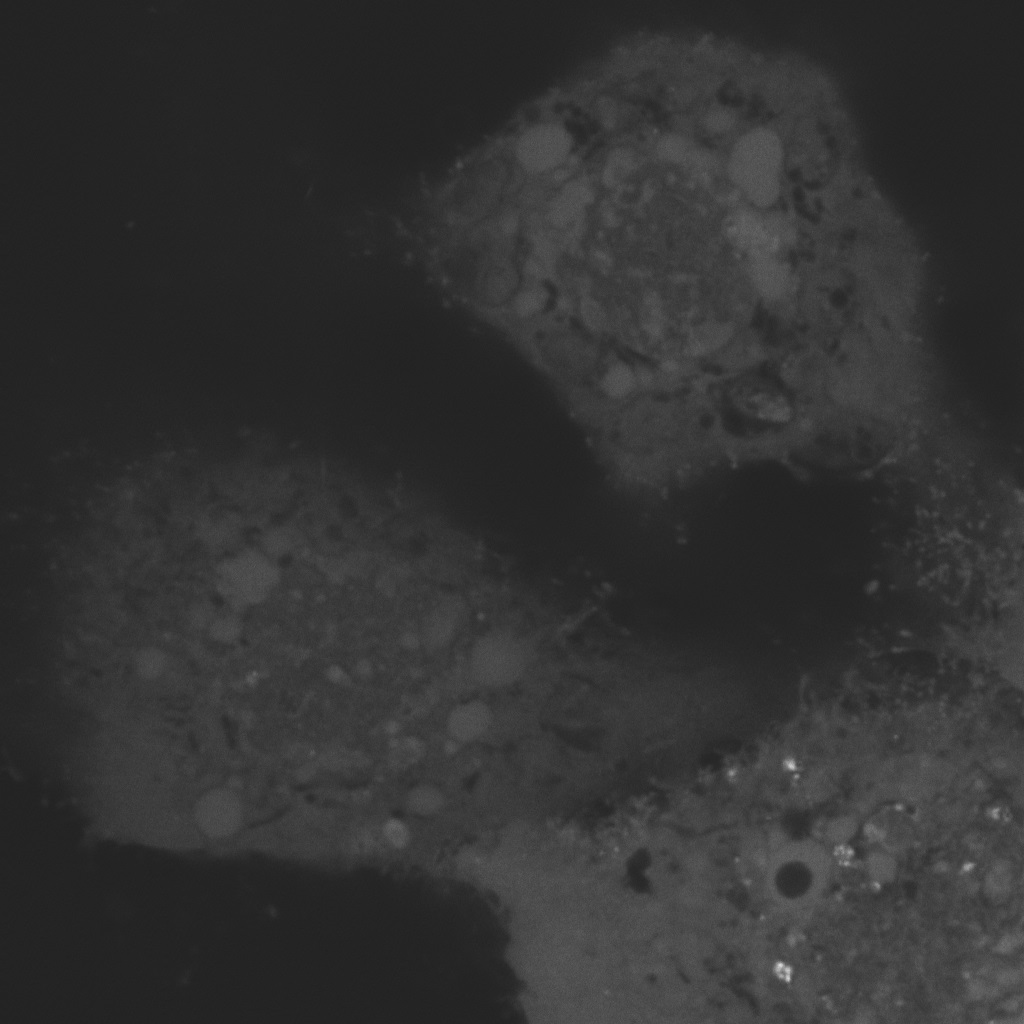

Supplement: Supplementary file 18 — Source Data for Figure 4 [file EMBJ-42-e113349-s019.zip › EMBOJ-2022-113349_SourceDataForFigure 4/4A/GFP-p62/4A (GFP-p62_mCherry)_mCherry.jpg]

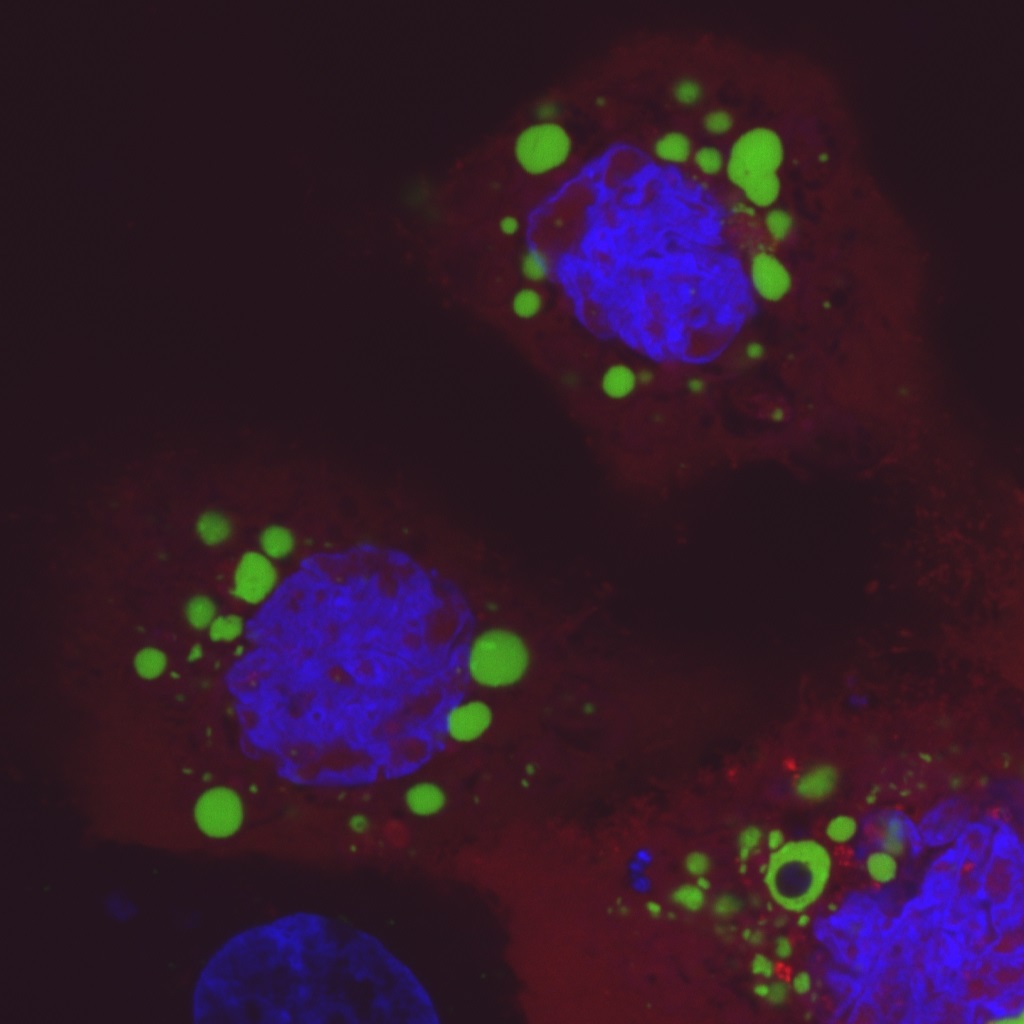

Supplement: Supplementary file 18 — Source Data for Figure 4 [file EMBJ-42-e113349-s019.zip › EMBOJ-2022-113349_SourceDataForFigure 4/4A/GFP-p62/4A (GFP-p62_mCherry)_merged.jpg]

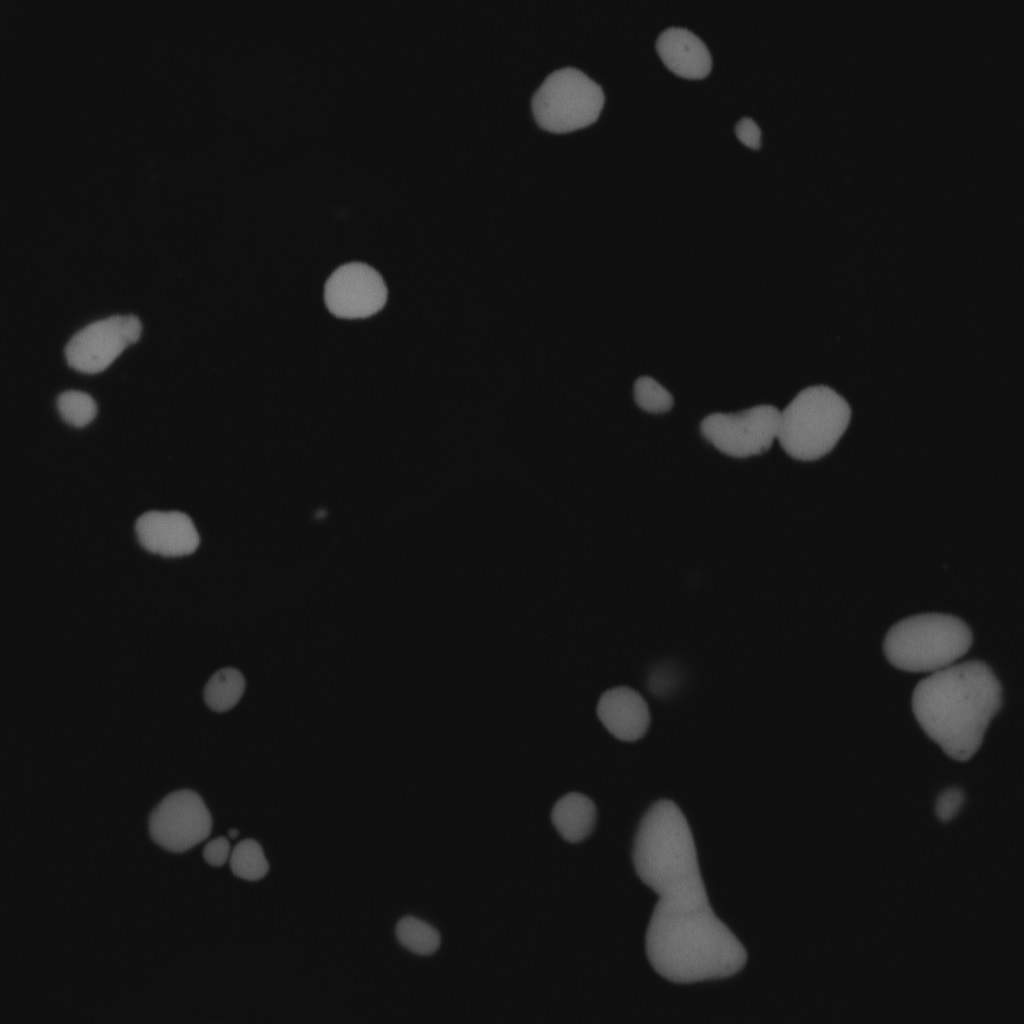

Supplement: Supplementary file 18 — Source Data for Figure 4 [file EMBJ-42-e113349-s019.zip › EMBOJ-2022-113349_SourceDataForFigure 4/4A/GFP-p62/4A (GFP-p62_mCherry-KEAP1)_mCherry.jpg]

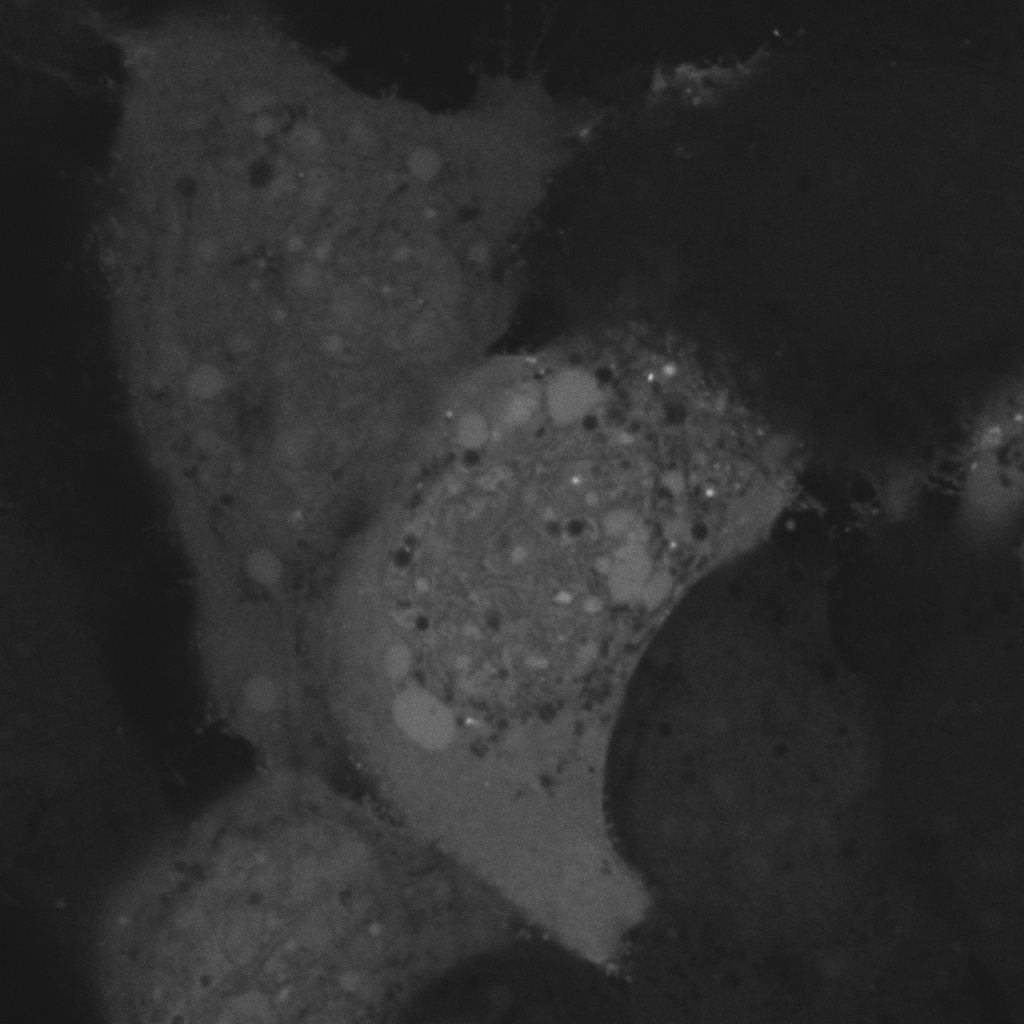

Supplement: Supplementary file 18 — Source Data for Figure 4 [file EMBJ-42-e113349-s019.zip › EMBOJ-2022-113349_SourceDataForFigure 4/4A/GFP-p62 S349A/4A (GFP-p62 S349A_mCherry)_mCherry.jpg]

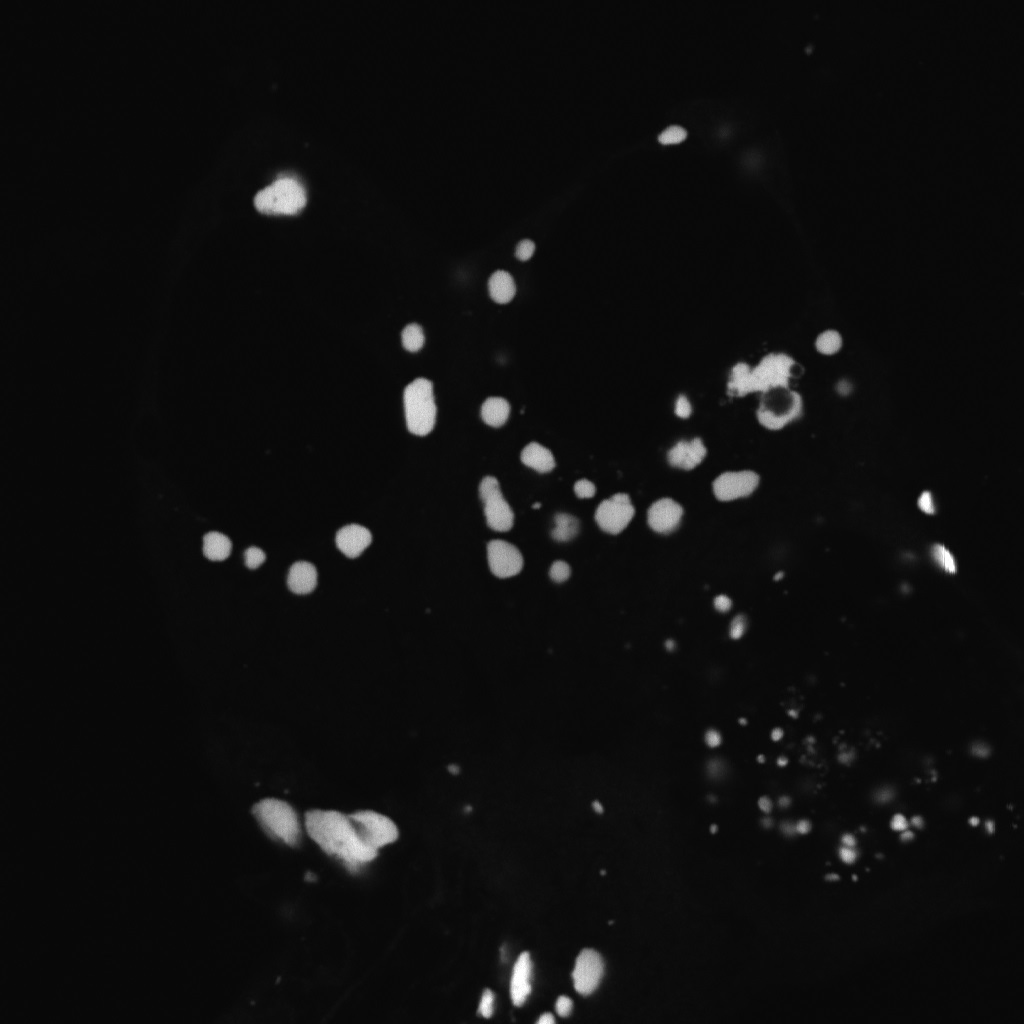

Supplement: Supplementary file 18 — Source Data for Figure 4 [file EMBJ-42-e113349-s019.zip › EMBOJ-2022-113349_SourceDataForFigure 4/4A/GFP-p62 T350A/4A (GFP-p62 T350A_mCherry-KEAP1)_GFP.jpg]

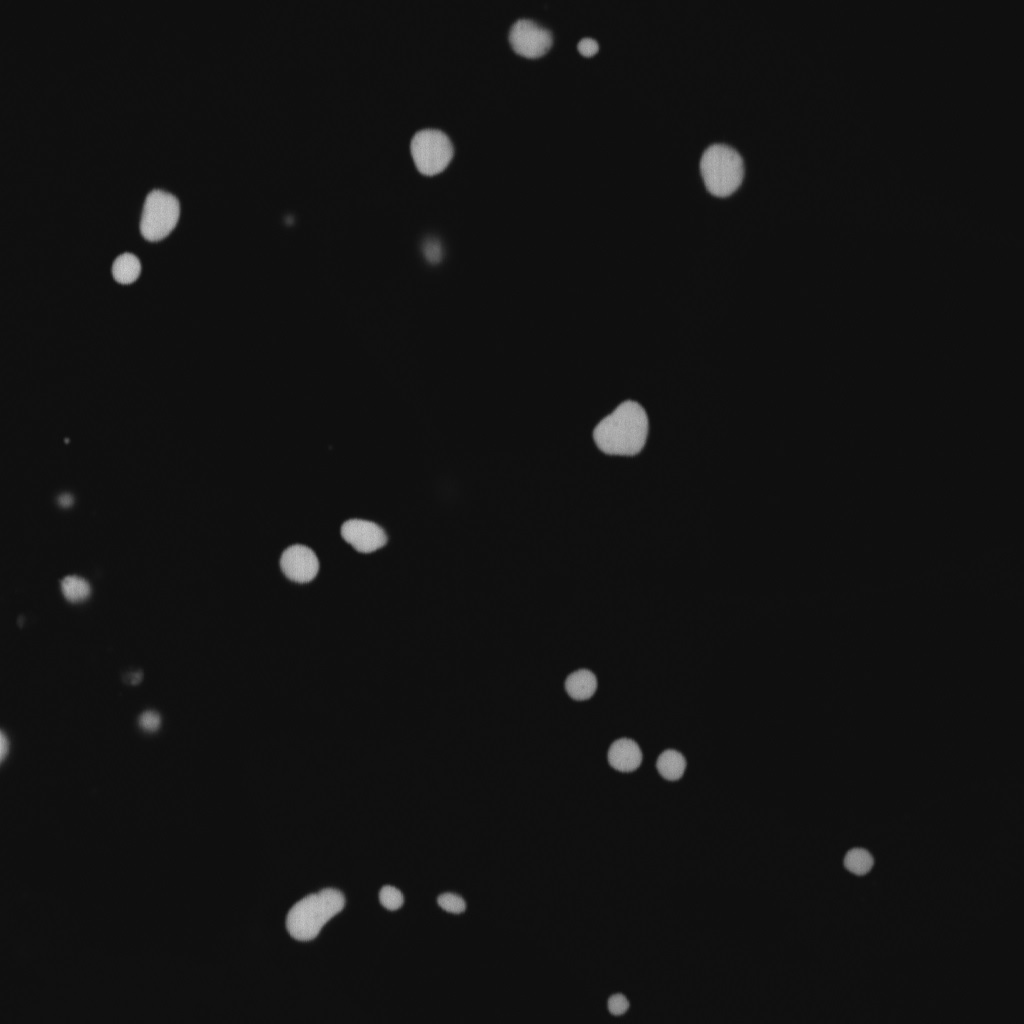

Supplement: Supplementary file 18 — Source Data for Figure 4 [file EMBJ-42-e113349-s019.zip › EMBOJ-2022-113349_SourceDataForFigure 4/4A/GFP-p62 S349A/4A (GFP-p62 S349A_mCherry-KEAP1)_GFP.jpg]

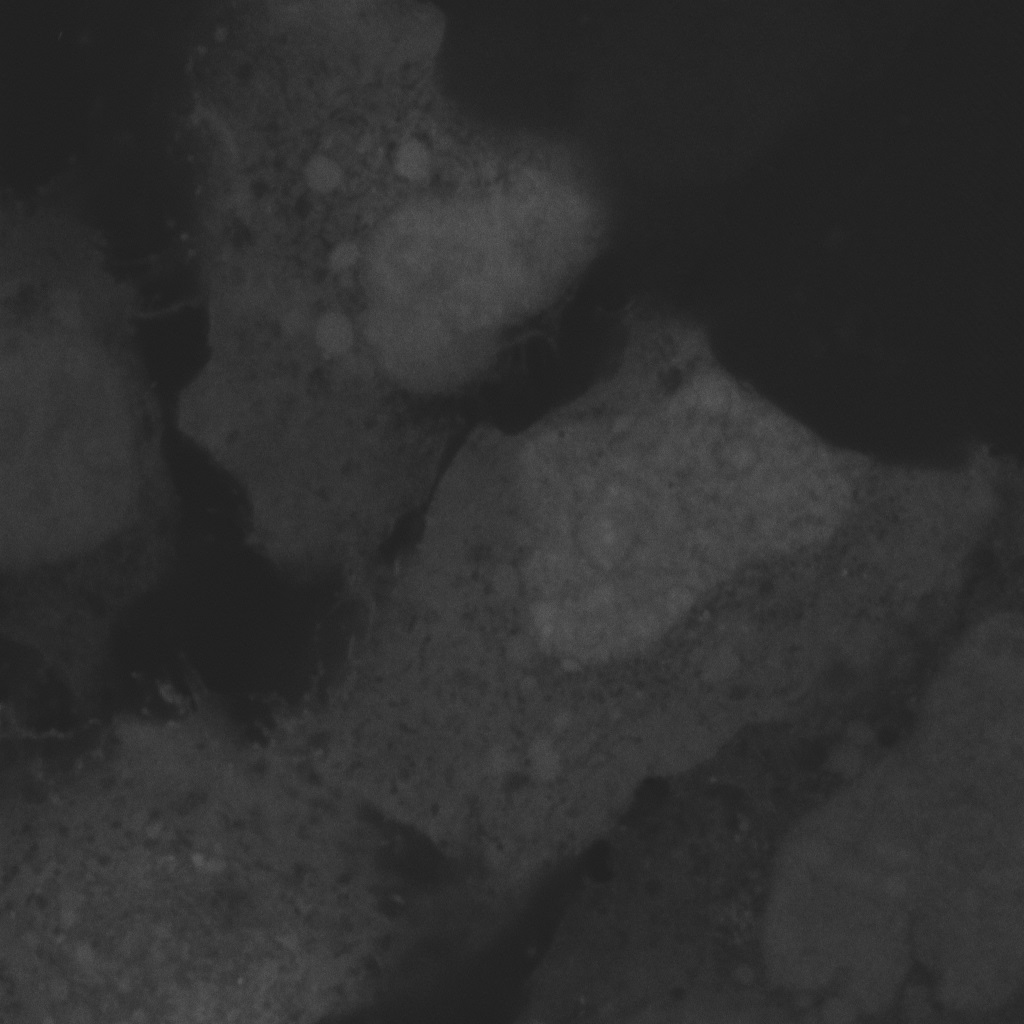

Supplement: Supplementary file 18 — Source Data for Figure 4 [file EMBJ-42-e113349-s019.zip › EMBOJ-2022-113349_SourceDataForFigure 4/4A/GFP-p62 S349E/4A (GFP-p62 S349E_mCherry)_mCherry.jpg]

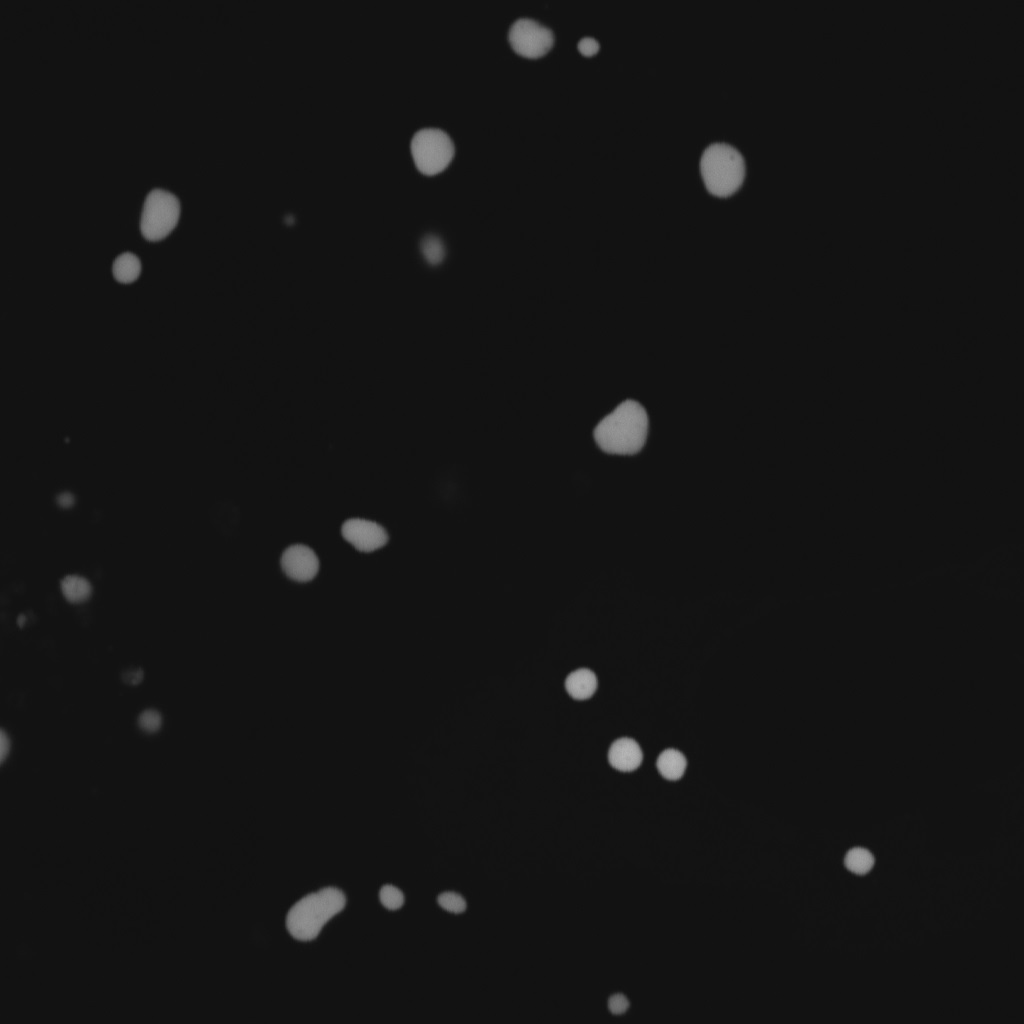

Supplement: Supplementary file 18 — Source Data for Figure 4 [file EMBJ-42-e113349-s019.zip › EMBOJ-2022-113349_SourceDataForFigure 4/4A/GFP-p62 S349A/4A (GFP-p62 S349A_mCherry-KEAP1)_mCherry.jpg]

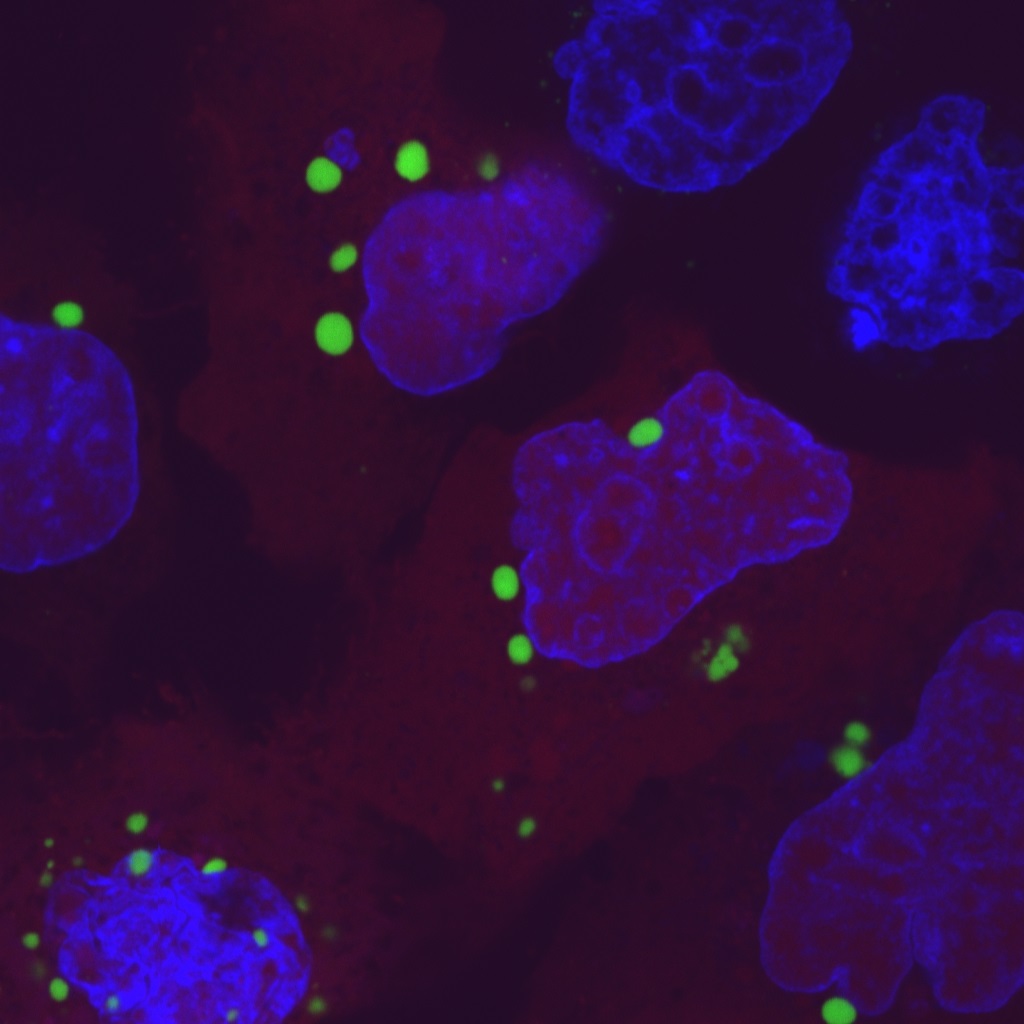

Supplement: Supplementary file 18 — Source Data for Figure 4 [file EMBJ-42-e113349-s019.zip › EMBOJ-2022-113349_SourceDataForFigure 4/4A/GFP-p62 S349E/4A (GFP-p62 S349E_mCherry)_merged.jpg]

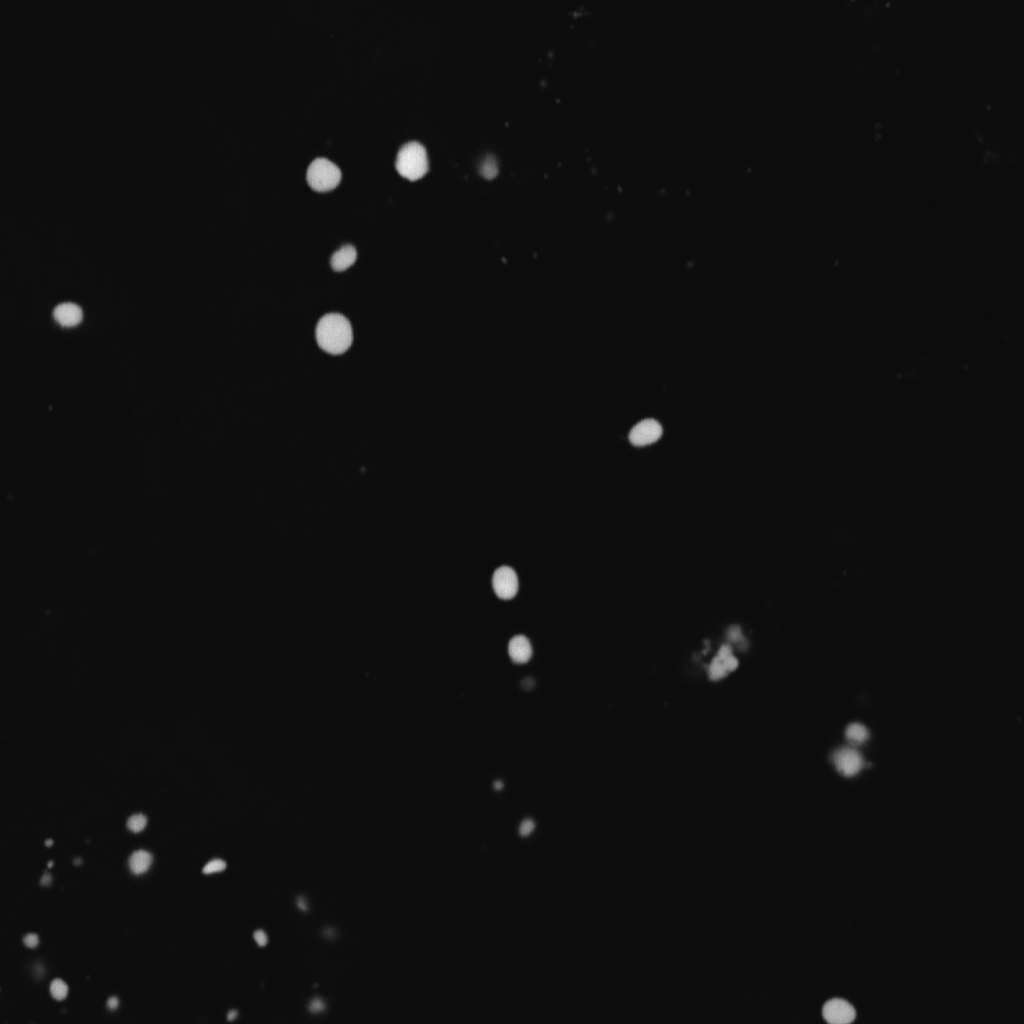

Supplement: Supplementary file 18 — Source Data for Figure 4 [file EMBJ-42-e113349-s019.zip › EMBOJ-2022-113349_SourceDataForFigure 4/4A/GFP-p62 S349E/4A (GFP-p62 S349E_mCherry)_GFP.jpg]

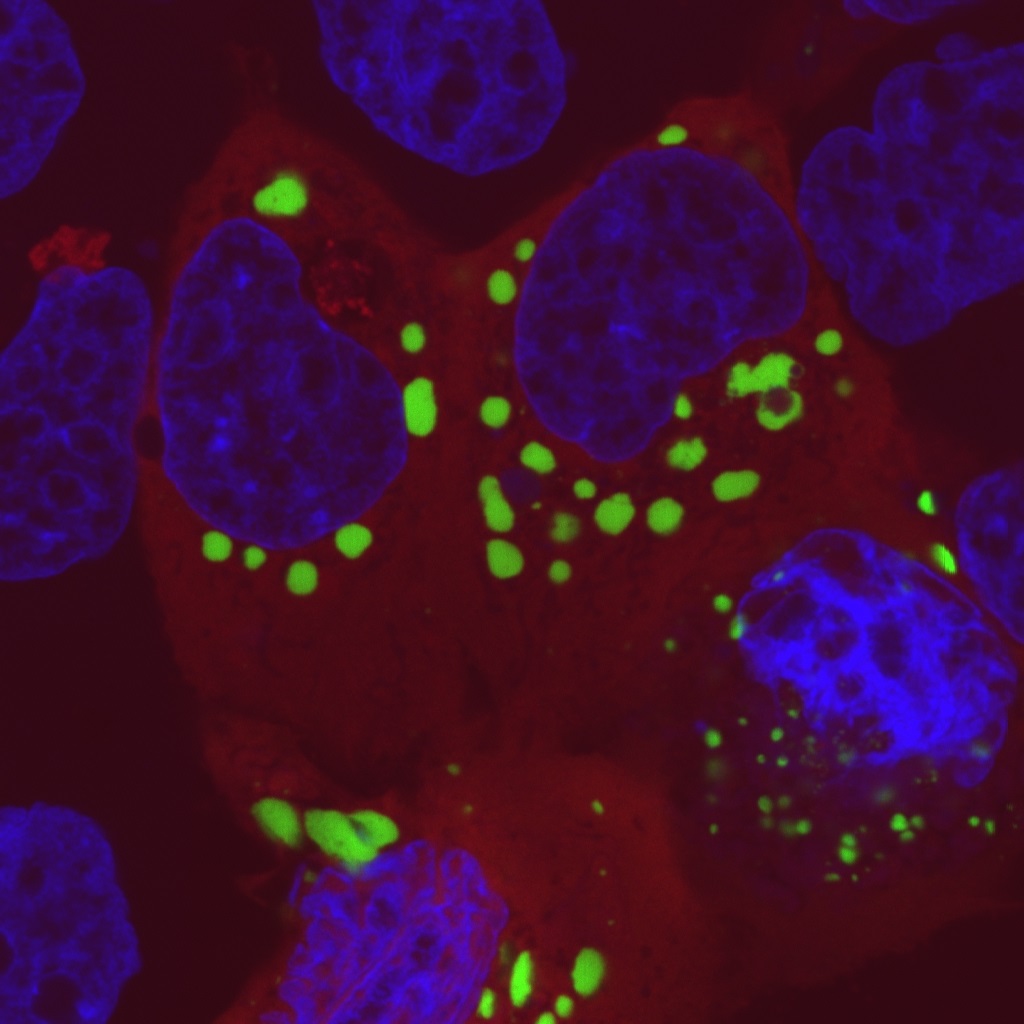

Supplement: Supplementary file 18 — Source Data for Figure 4 [file EMBJ-42-e113349-s019.zip › EMBOJ-2022-113349_SourceDataForFigure 4/4A/GFP-p62 T350A/4A (GFP-p62 T350A_mCherry-KEAP1)_merged.jpg]

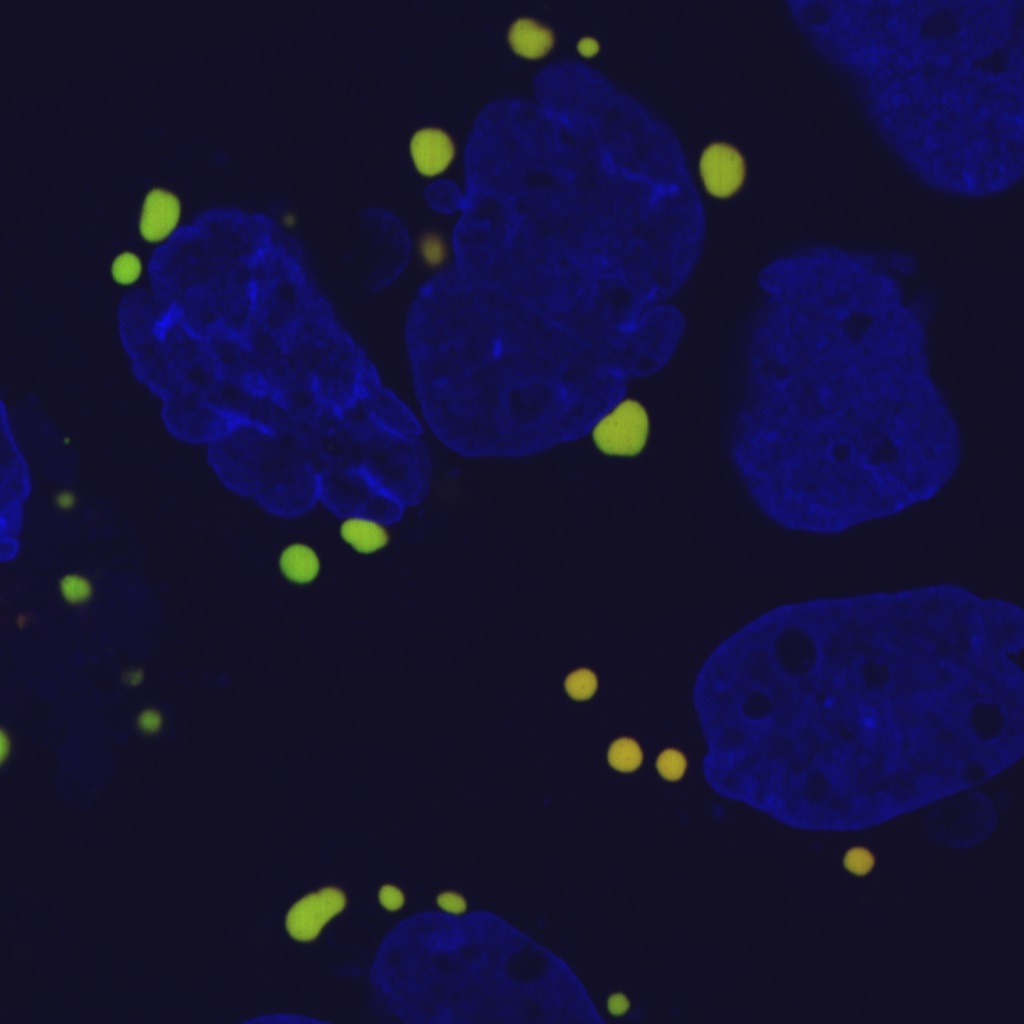

Supplement: Supplementary file 18 — Source Data for Figure 4 [file EMBJ-42-e113349-s019.zip › EMBOJ-2022-113349_SourceDataForFigure 4/4A/GFP-p62 S349A/4A (GFP-p62 S349A_mCherry-KEAP1)_merged.jpg]

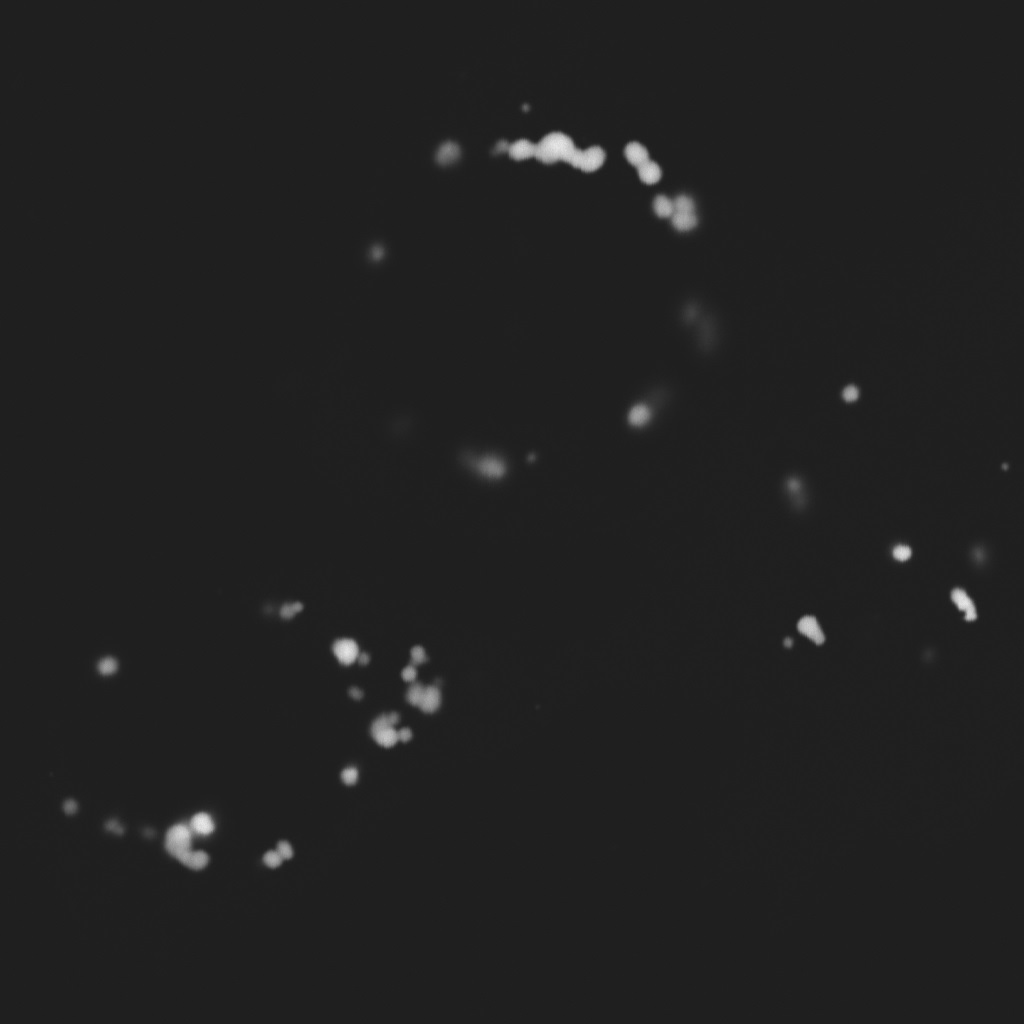

Supplement: Supplementary file 18 — Source Data for Figure 4 [file EMBJ-42-e113349-s019.zip › EMBOJ-2022-113349_SourceDataForFigure 4/4A/GFP-p62 S349E/4A (GFP-p62 S349E_mCherry-KEAP1)_mCherry.jpg]

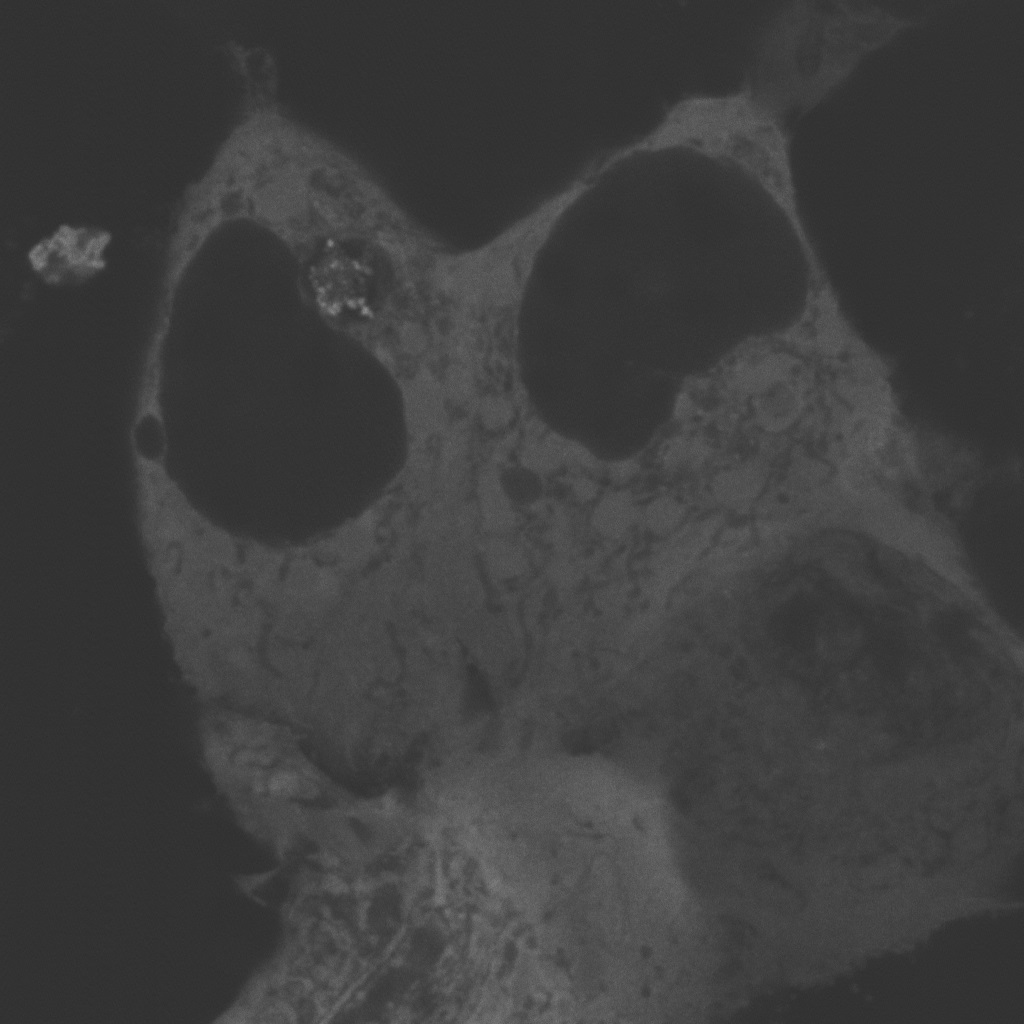

Supplement: Supplementary file 18 — Source Data for Figure 4 [file EMBJ-42-e113349-s019.zip › EMBOJ-2022-113349_SourceDataForFigure 4/4A/GFP-p62 T350A/4A (GFP-p62 T350A_mCherry-KEAP1)_mCherry.jpg]

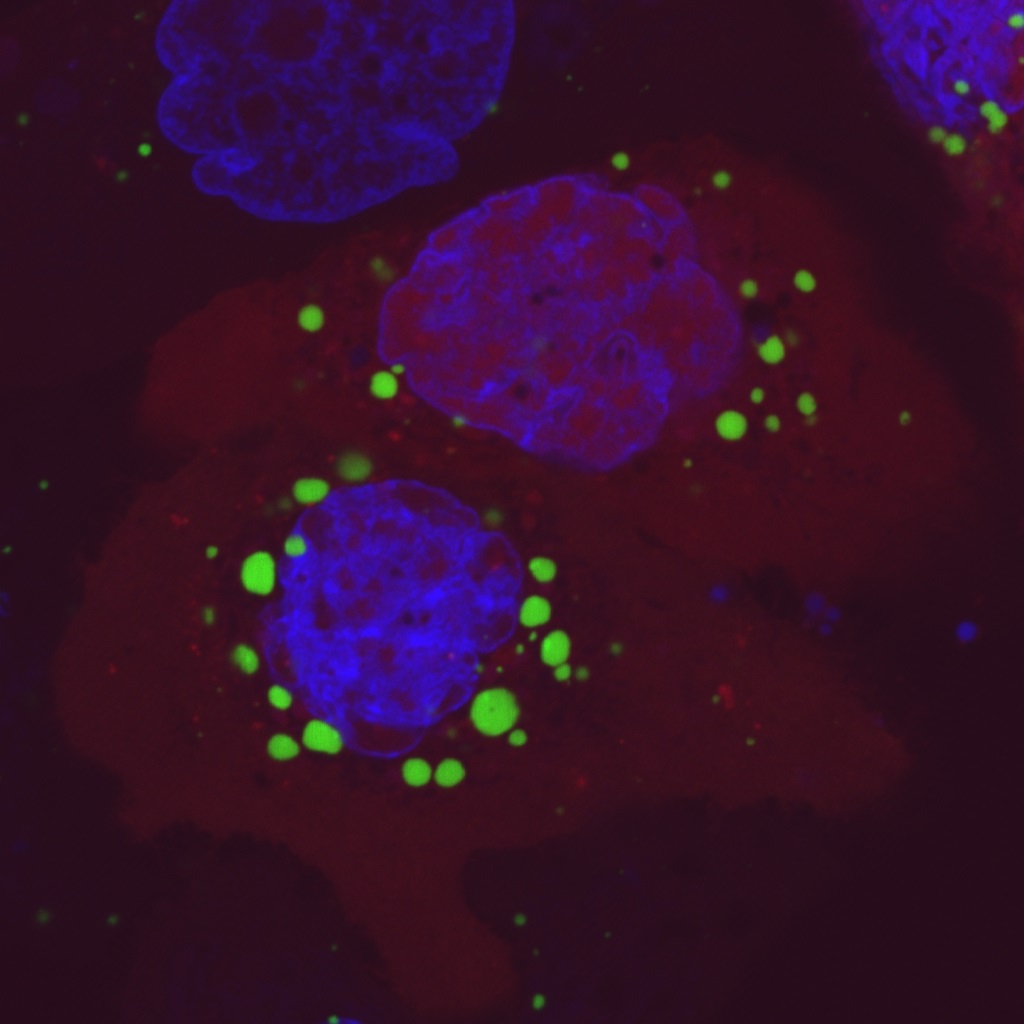

Supplement: Supplementary file 18 — Source Data for Figure 4 [file EMBJ-42-e113349-s019.zip › EMBOJ-2022-113349_SourceDataForFigure 4/4A/GFP-p62 T350A/4A (GFP-p62 T350A_mCherry)_merged.jpg]

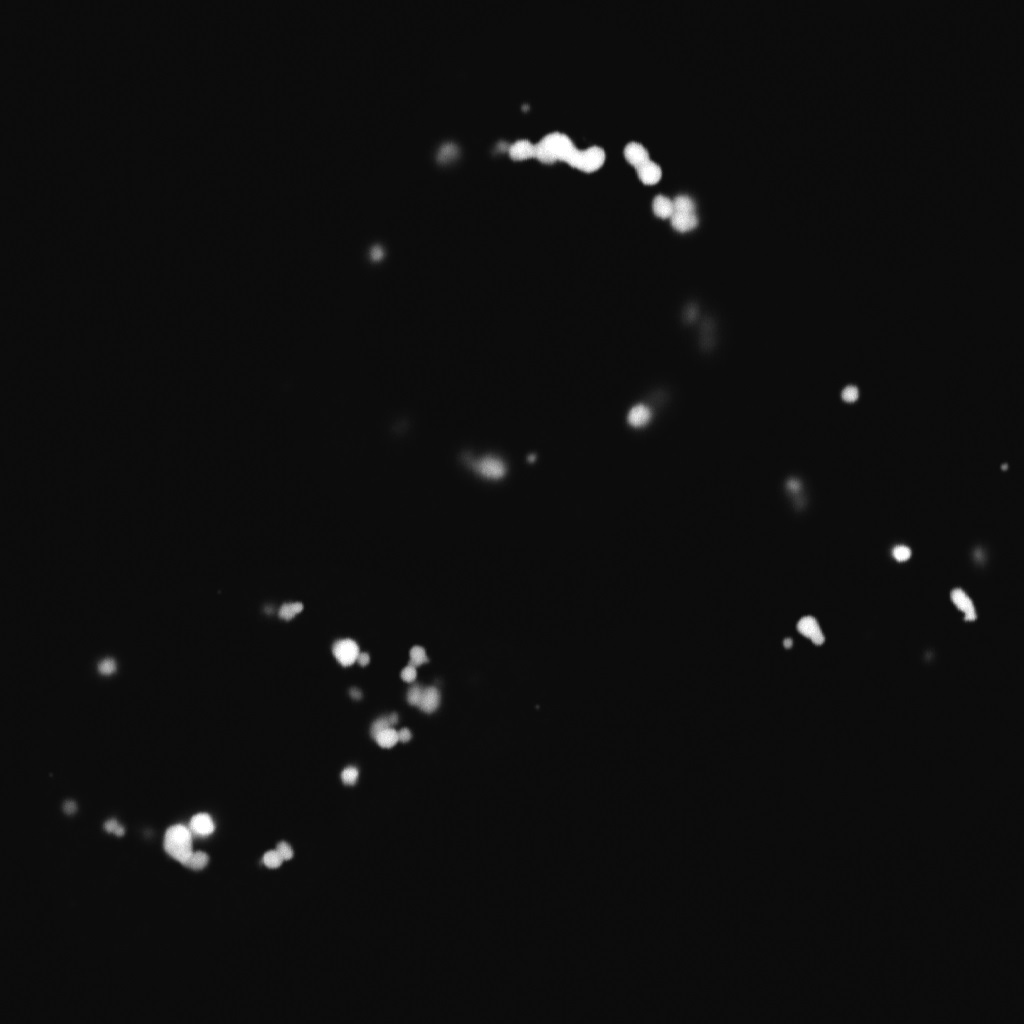

Supplement: Supplementary file 18 — Source Data for Figure 4 [file EMBJ-42-e113349-s019.zip › EMBOJ-2022-113349_SourceDataForFigure 4/4A/GFP-p62 S349E/4A (GFP-p62 S349E_mCherry-KEAP1)_GFP.jpg]

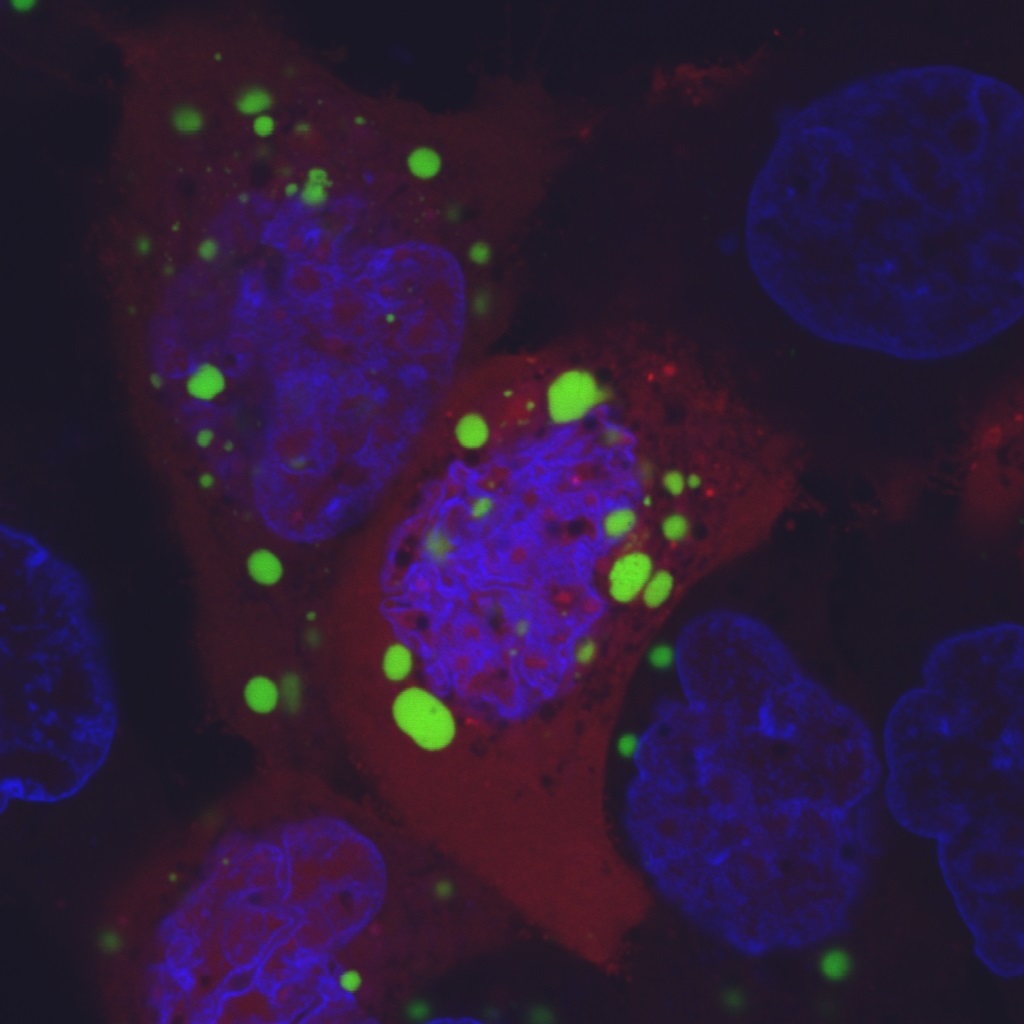

Supplement: Supplementary file 18 — Source Data for Figure 4 [file EMBJ-42-e113349-s019.zip › EMBOJ-2022-113349_SourceDataForFigure 4/4A/GFP-p62 S349A/4A (GFP-p62 S349A_mCherry)_merged.jpg]

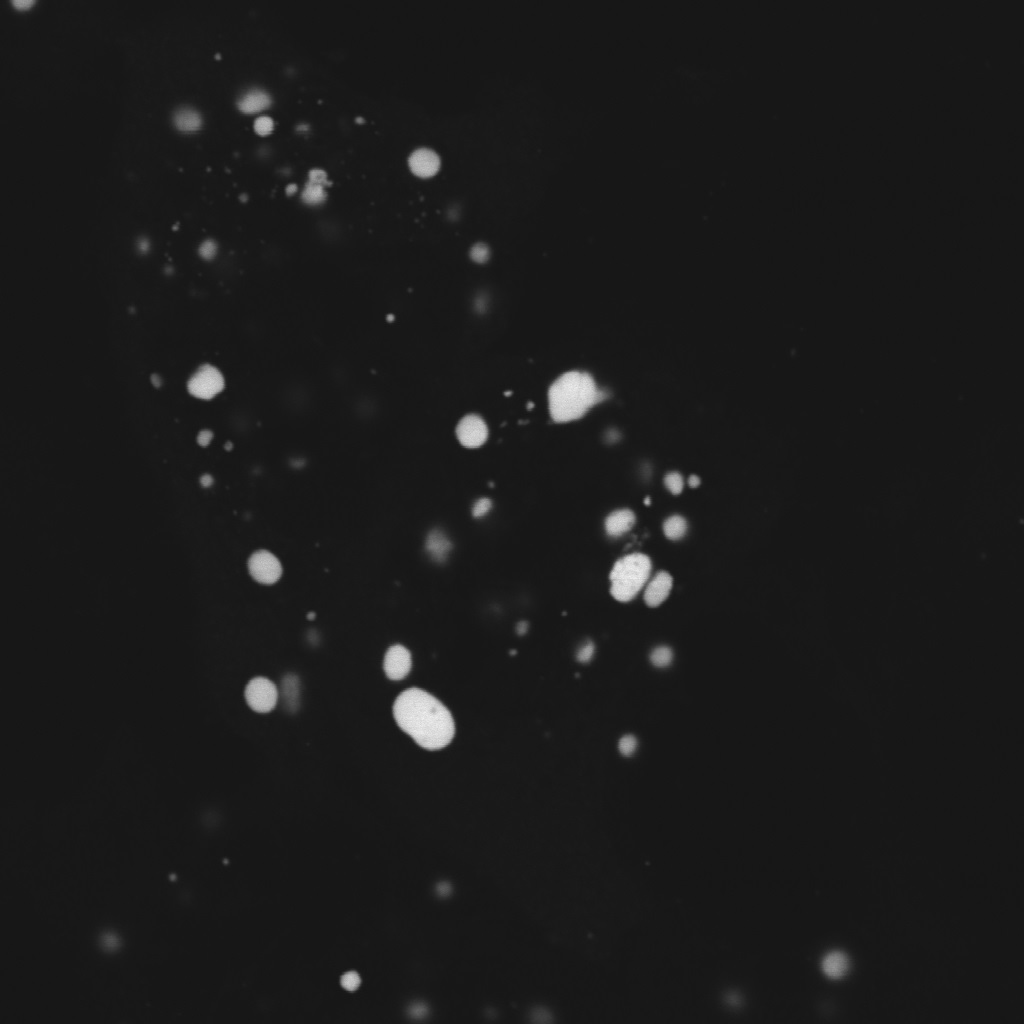

Supplement: Supplementary file 18 — Source Data for Figure 4 [file EMBJ-42-e113349-s019.zip › EMBOJ-2022-113349_SourceDataForFigure 4/4A/GFP-p62 S349A/4A (GFP-p62 S349A_mCherry)_GFP.jpg]

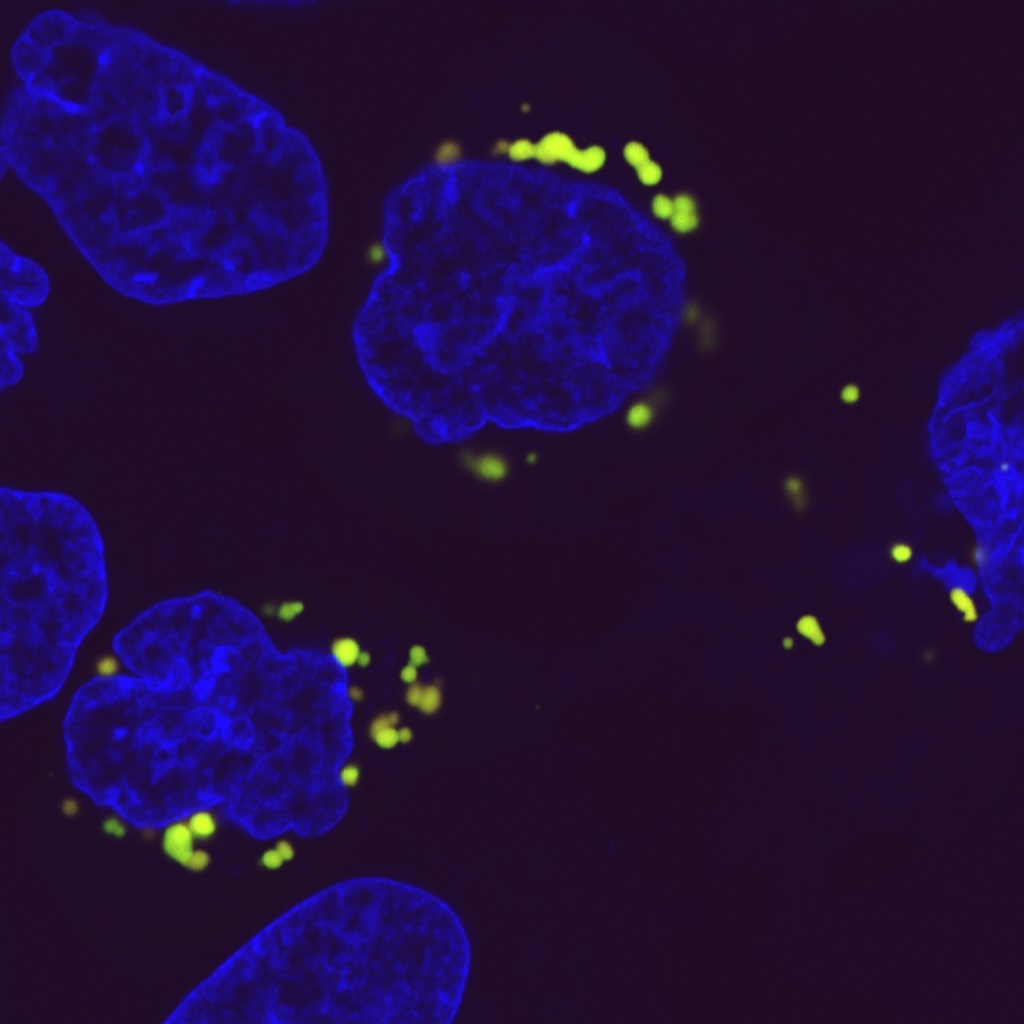

Supplement: Supplementary file 18 — Source Data for Figure 4 [file EMBJ-42-e113349-s019.zip › EMBOJ-2022-113349_SourceDataForFigure 4/4A/GFP-p62 S349E/4A (GFP-p62 S349E_mCherry-KEAP1)_merged.jpg]

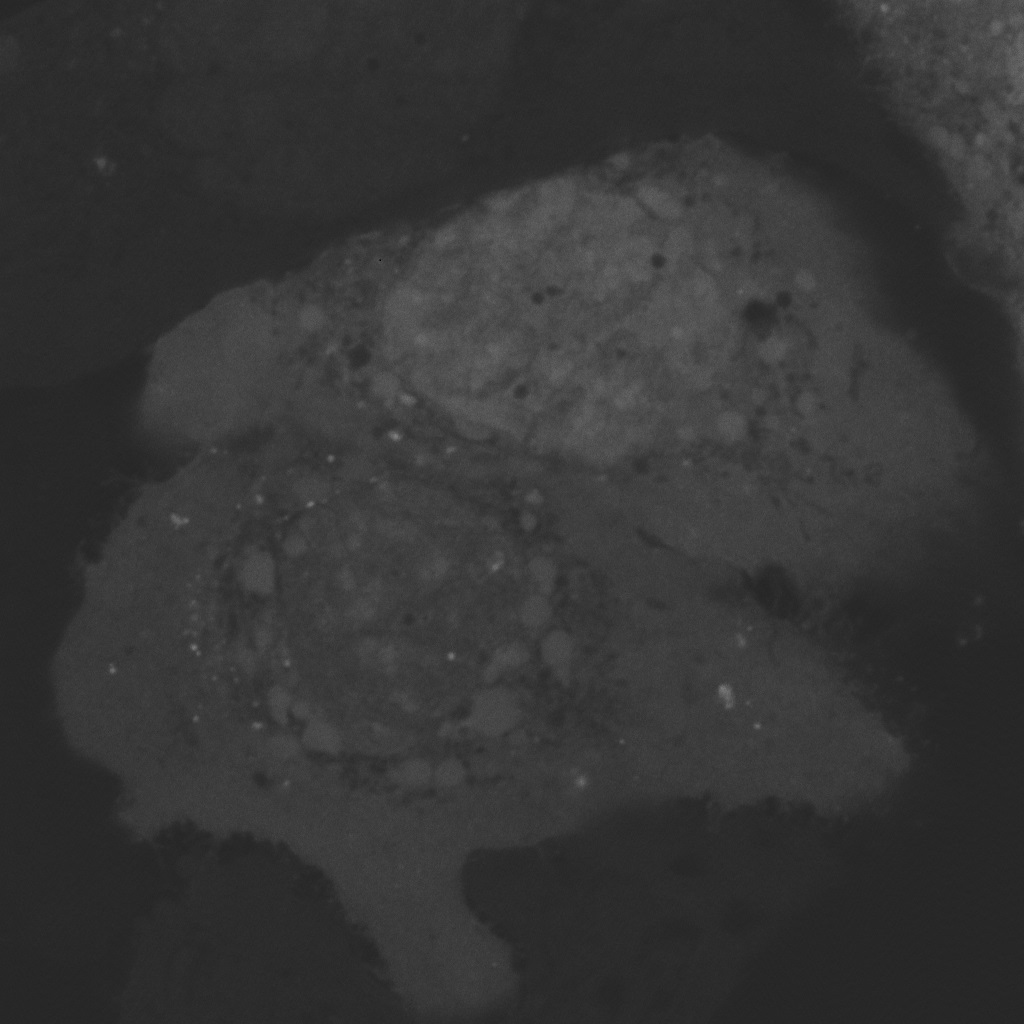

Supplement: Supplementary file 18 — Source Data for Figure 4 [file EMBJ-42-e113349-s019.zip › EMBOJ-2022-113349_SourceDataForFigure 4/4A/GFP-p62 T350A/4A (GFP-p62 T350A_mCherry)_mCherry.jpg]

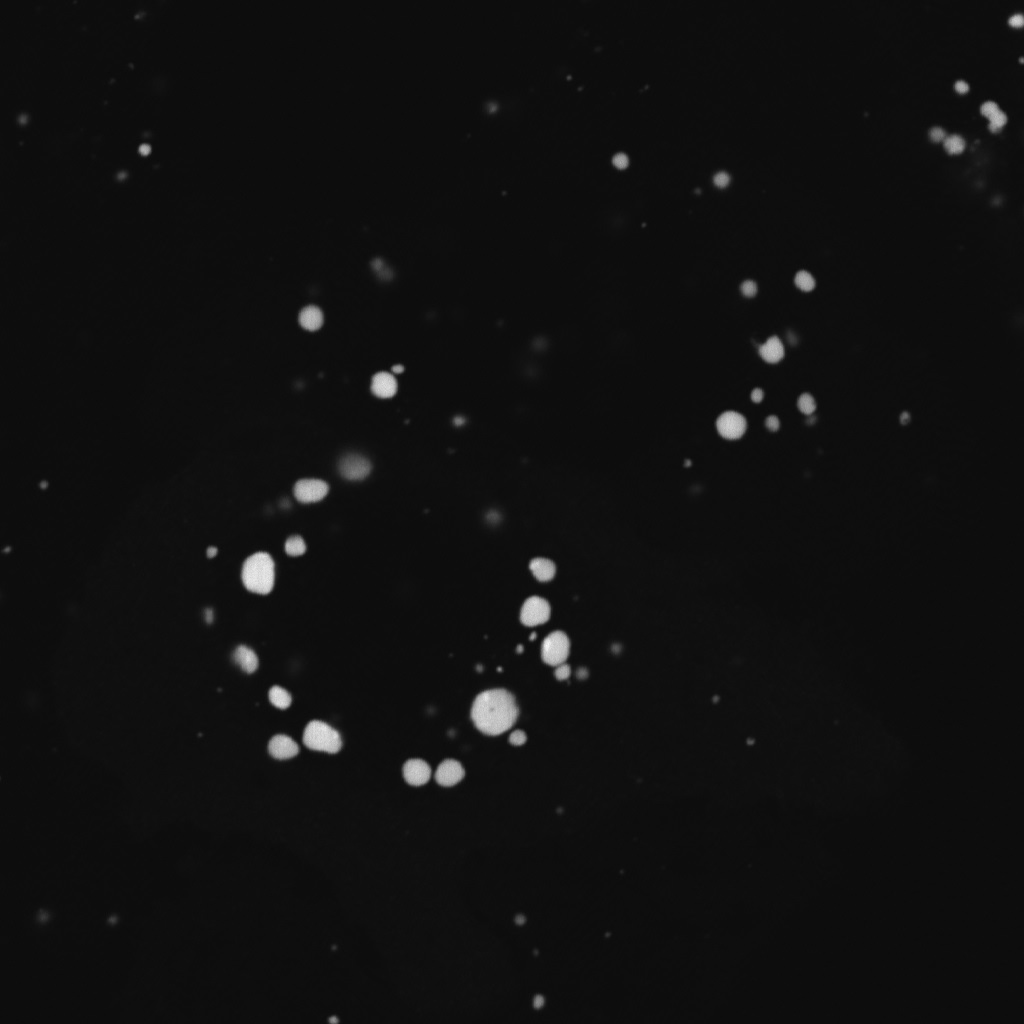

Supplement: Supplementary file 18 — Source Data for Figure 4 [file EMBJ-42-e113349-s019.zip › EMBOJ-2022-113349_SourceDataForFigure 4/4A/GFP-p62 T350A/4A (GFP-p62 T350A_mCherry)_GFP.jpg]

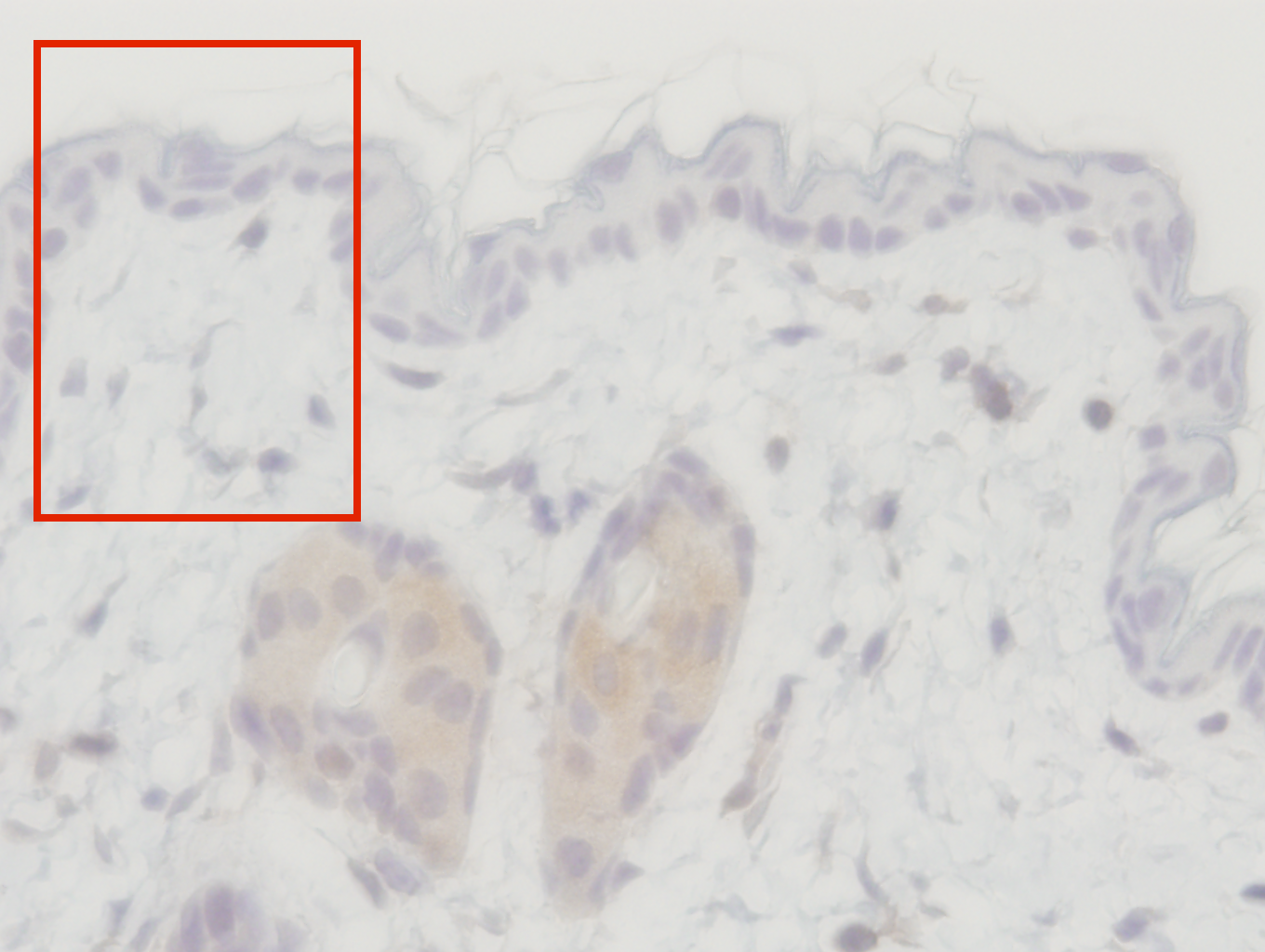

Supplement: Supplementary file 19 — Source Data for Figure 5 [file EMBJ-42-e113349-s009.zip › EMBOJ-2022-113349_SourceDataForFigure 5/5H/Skin_NQ01_6_ WT_X40mh_Marked.tif]

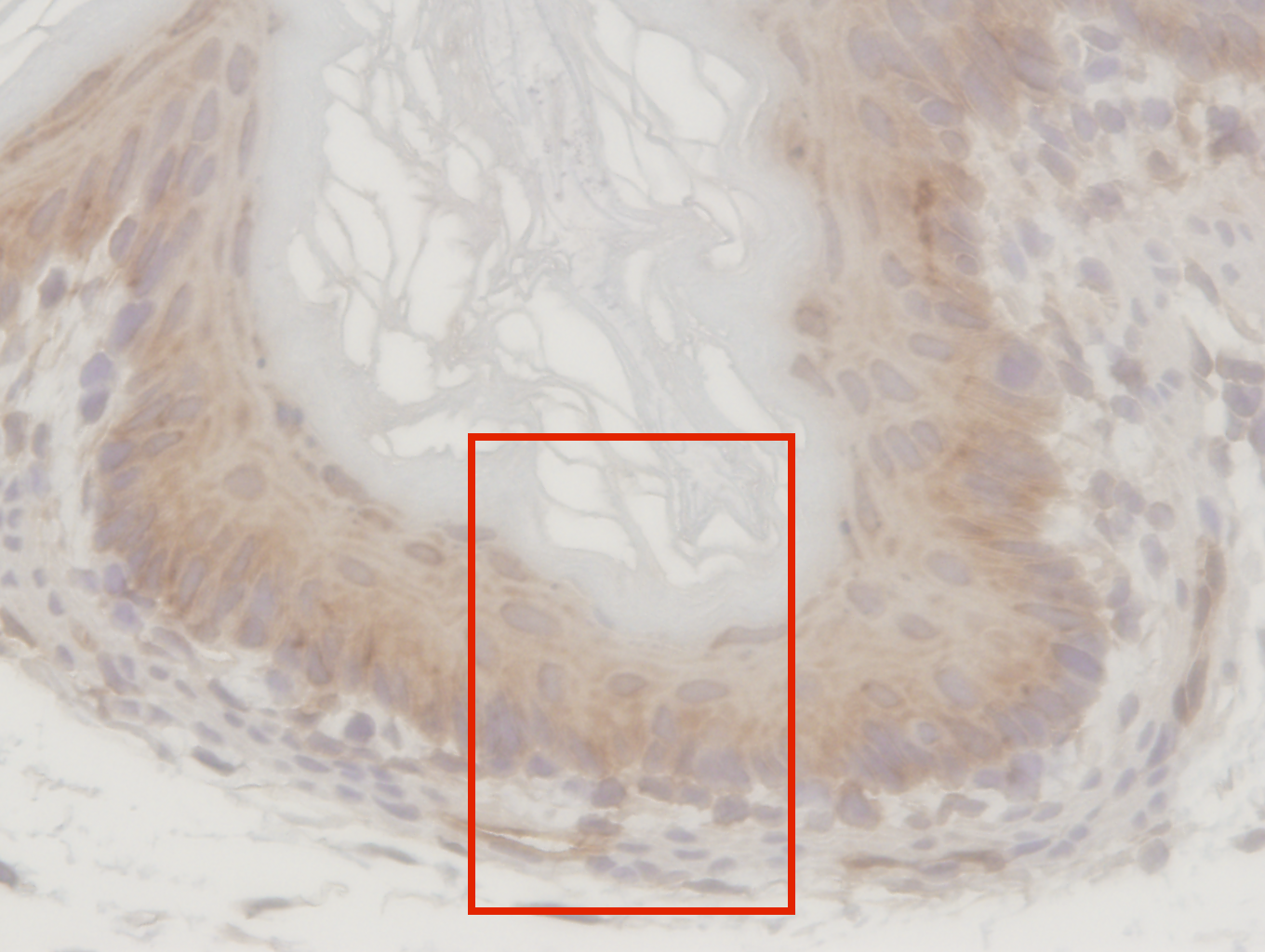

Supplement: Supplementary file 19 — Source Data for Figure 5 [file EMBJ-42-e113349-s009.zip › EMBOJ-2022-113349_SourceDataForFigure 5/5H/Esophagus_NQ01_7_ S531E_X40m_Marked.tif]

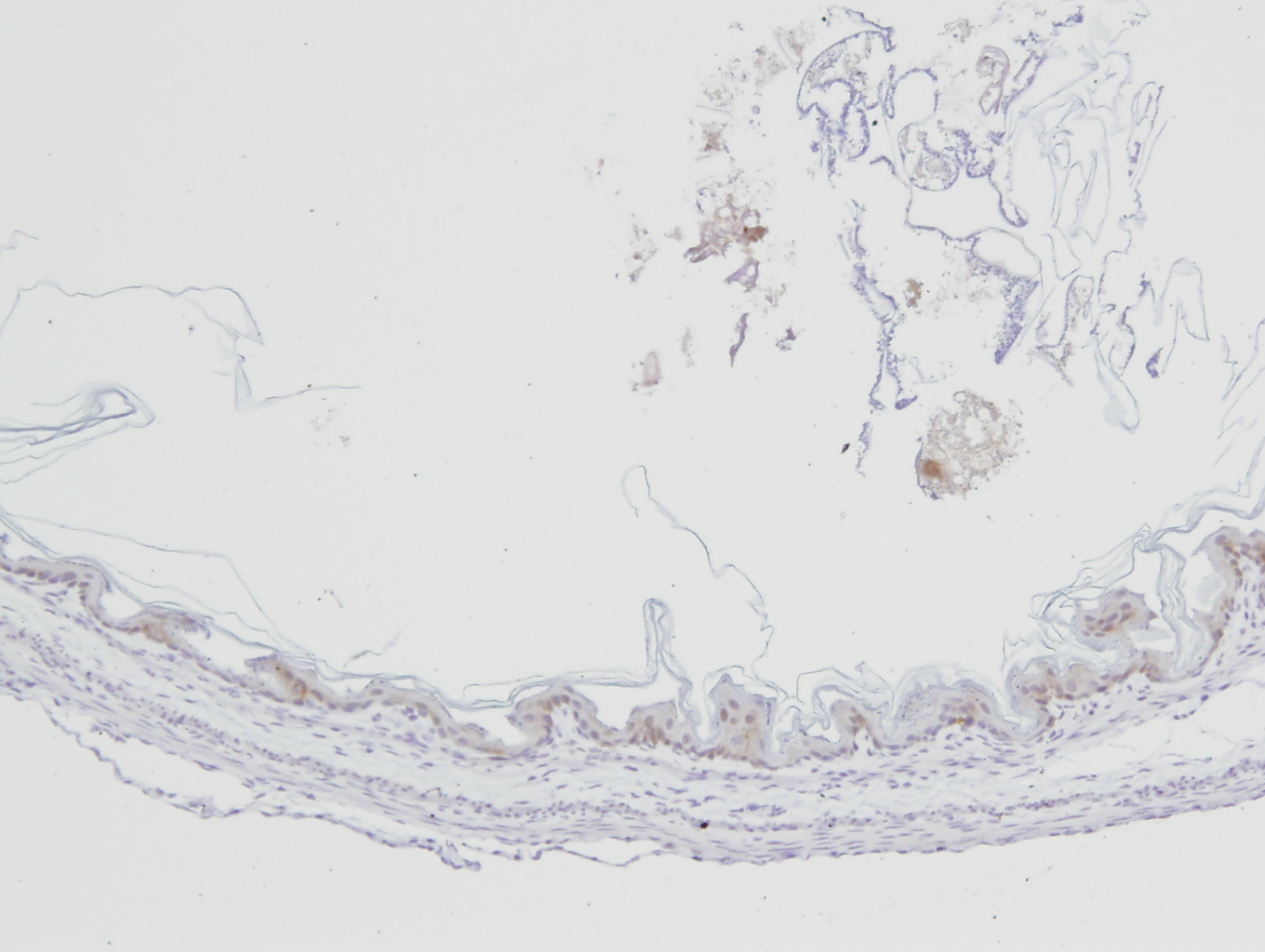

Supplement: Supplementary file 19 — Source Data for Figure 5 [file EMBJ-42-e113349-s009.zip › EMBOJ-2022-113349_SourceDataForFigure 5/5H/Forestomach_NQ01_6_WT_X10lh.tif]

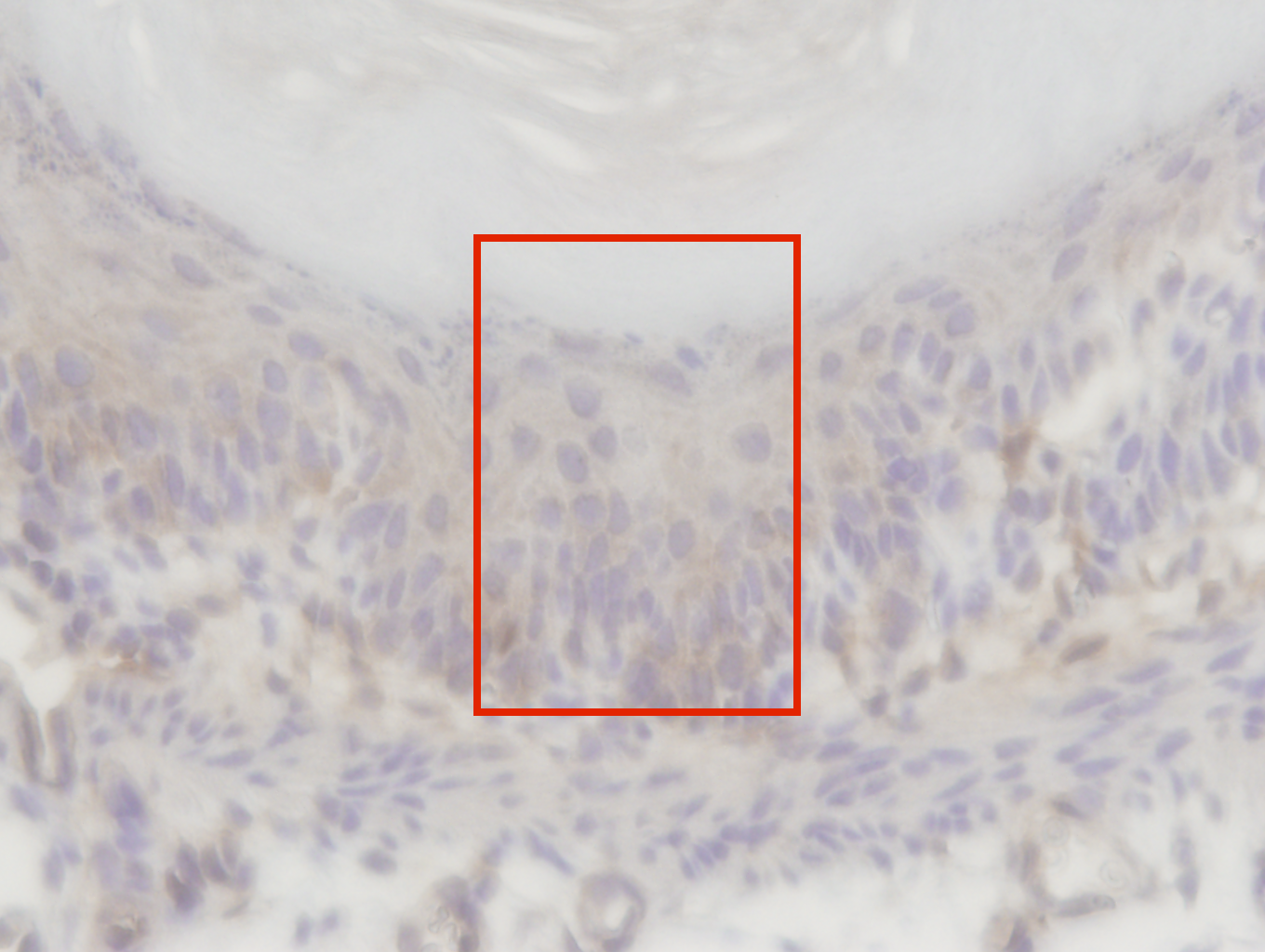

Supplement: Supplementary file 19 — Source Data for Figure 5 [file EMBJ-42-e113349-s009.zip › EMBOJ-2022-113349_SourceDataForFigure 5/5H/Forestomach_NQ01_7_ S351E_X40mh_Marked.tif]

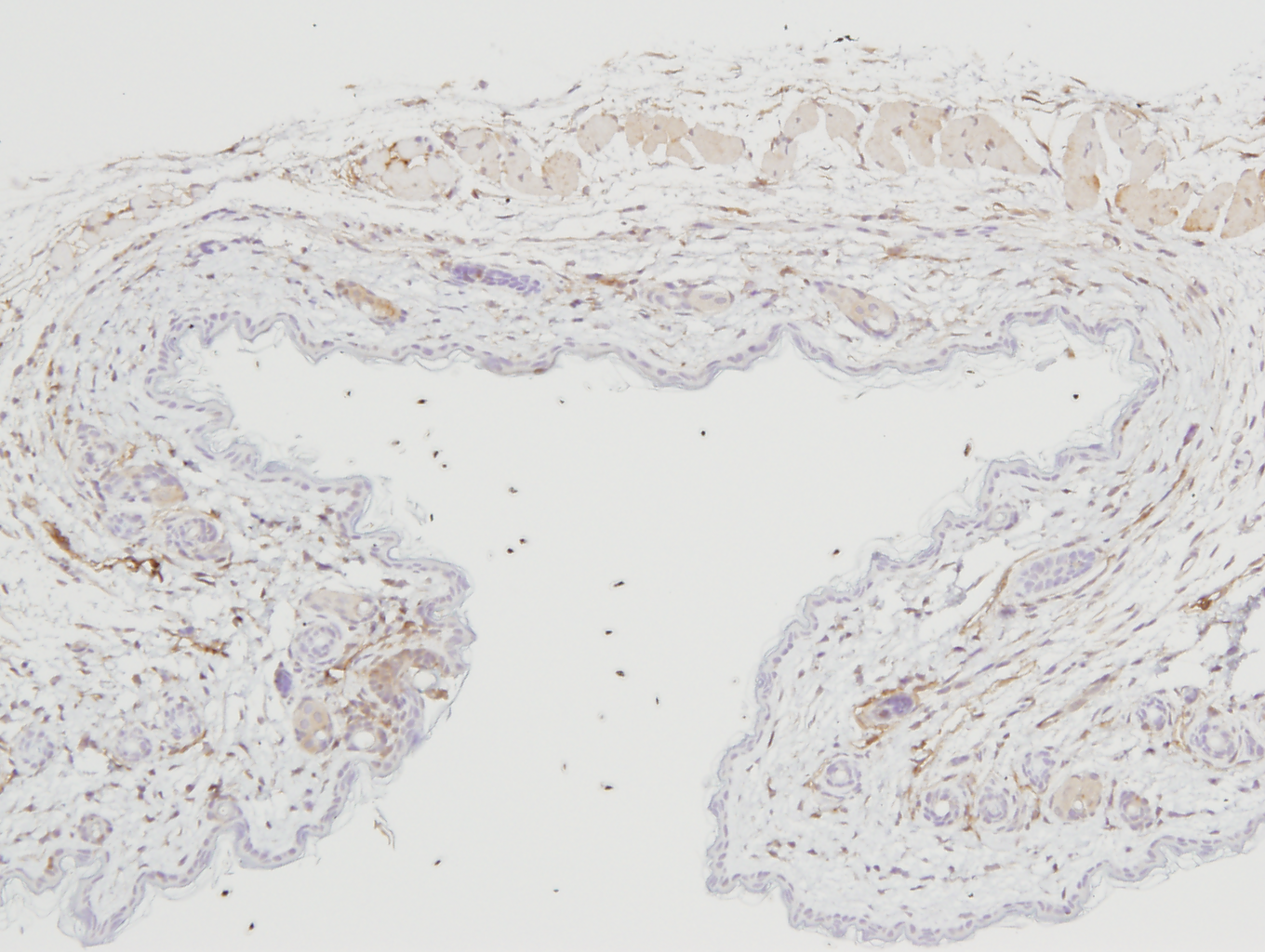

Supplement: Supplementary file 19 — Source Data for Figure 5 [file EMBJ-42-e113349-s009.zip › EMBOJ-2022-113349_SourceDataForFigure 5/5H/Skin_NQ01_7_ S351E_X10mh.tif]

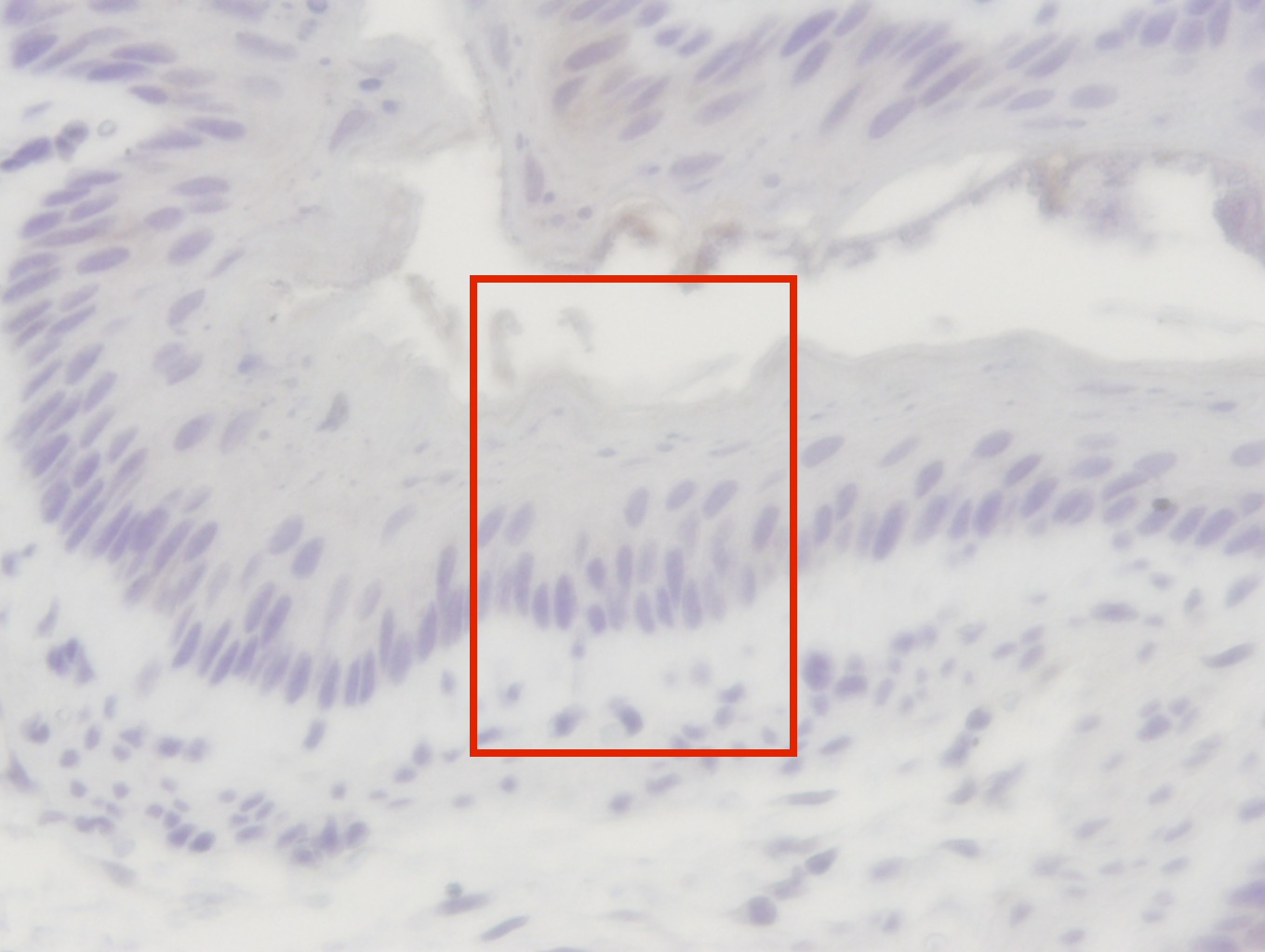

Supplement: Supplementary file 19 — Source Data for Figure 5 [file EMBJ-42-e113349-s009.zip › EMBOJ-2022-113349_SourceDataForFigure 5/5H/Esophagus_NQ01_6_ WT_X40m_Marked.tif]

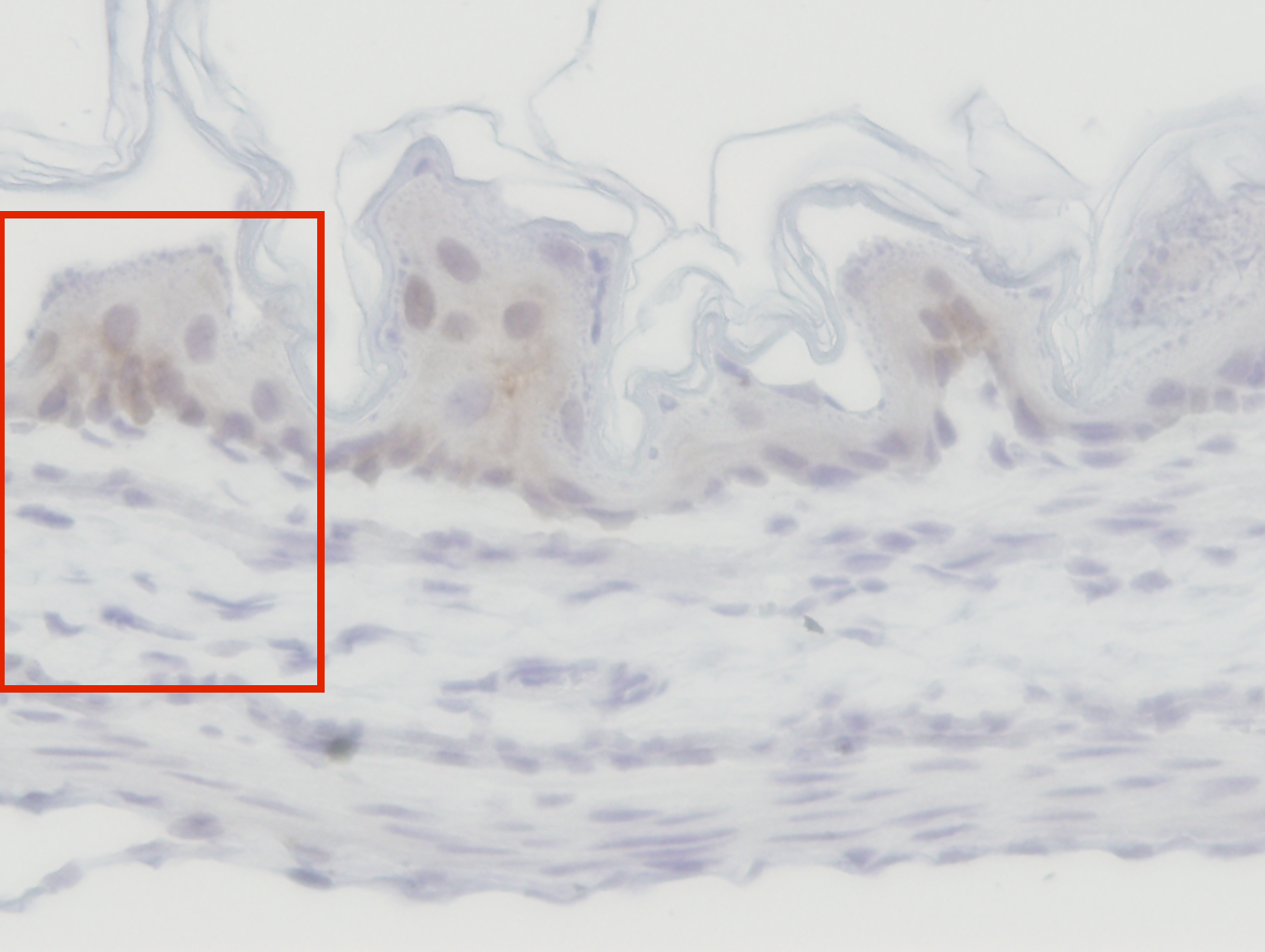

Supplement: Supplementary file 19 — Source Data for Figure 5 [file EMBJ-42-e113349-s009.zip › EMBOJ-2022-113349_SourceDataForFigure 5/5H/Forestomach_NQ01_6_WT_X40lh_Marked.tif]

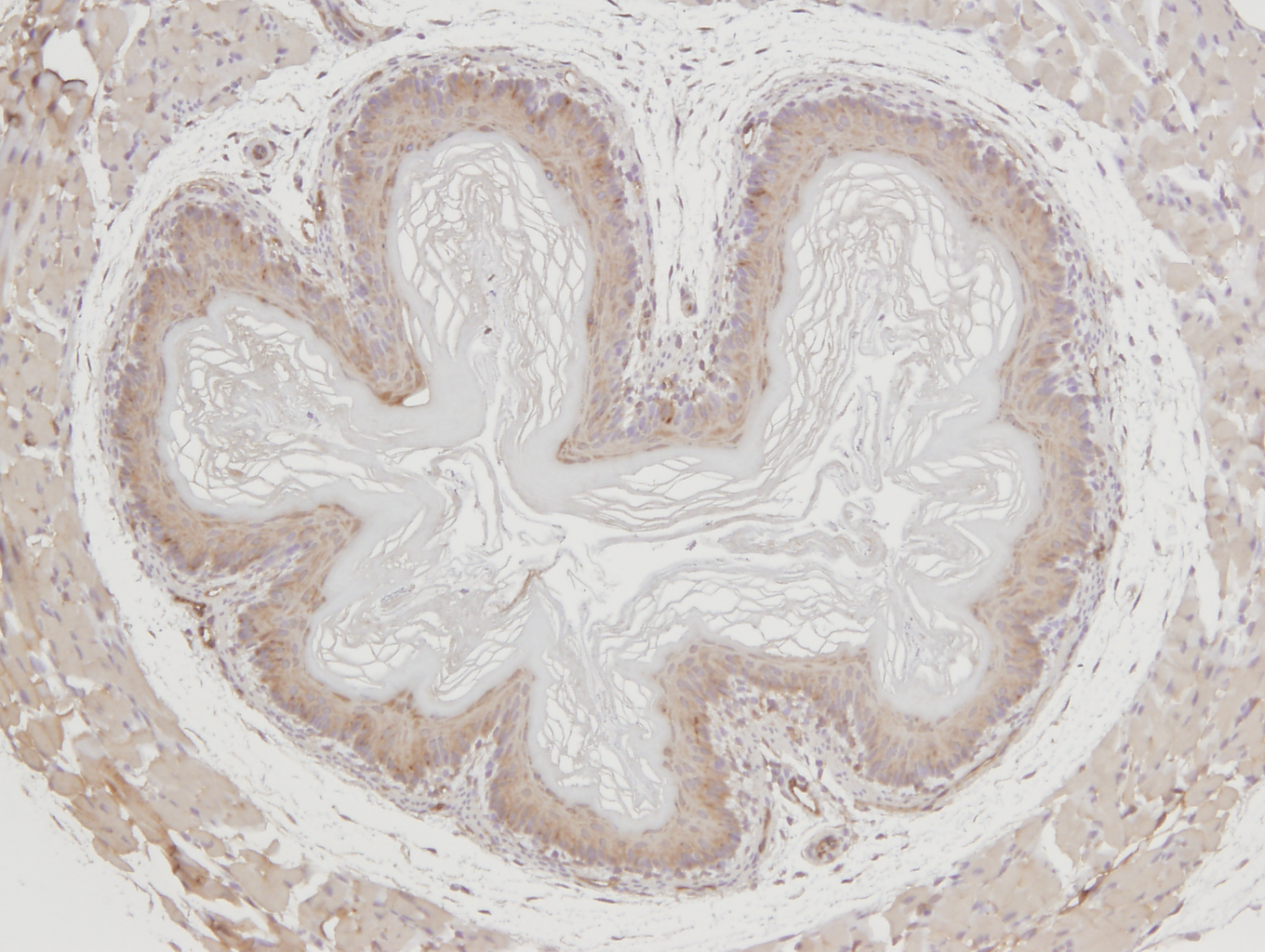

Supplement: Supplementary file 19 — Source Data for Figure 5 [file EMBJ-42-e113349-s009.zip › EMBOJ-2022-113349_SourceDataForFigure 5/5H/Esophagus_NQ01_7_ S531E_X10m.tif]

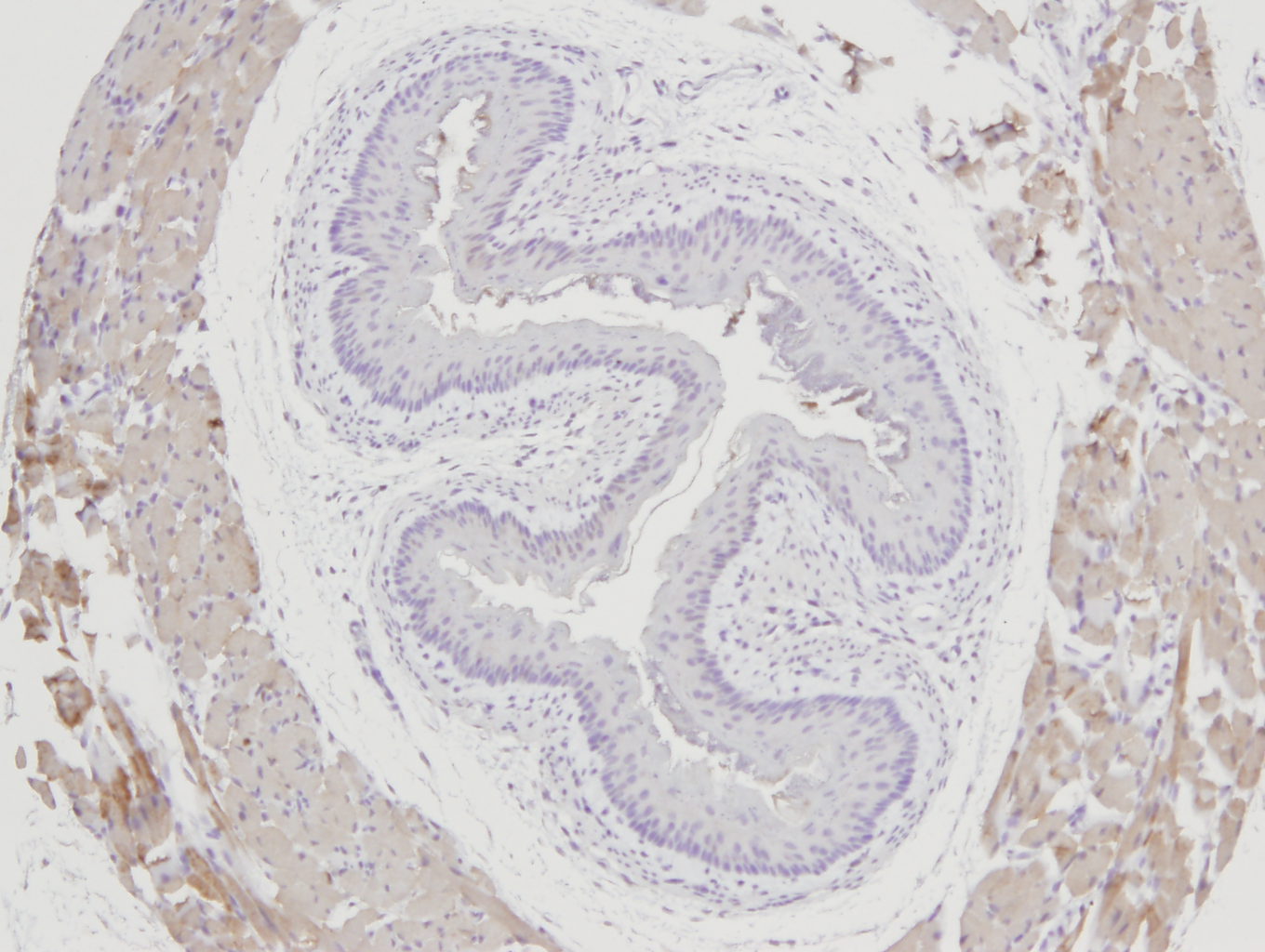

Supplement: Supplementary file 19 — Source Data for Figure 5 [file EMBJ-42-e113349-s009.zip › EMBOJ-2022-113349_SourceDataForFigure 5/5H/Esophagus_NQ01_6_ WT_X10m.tif]

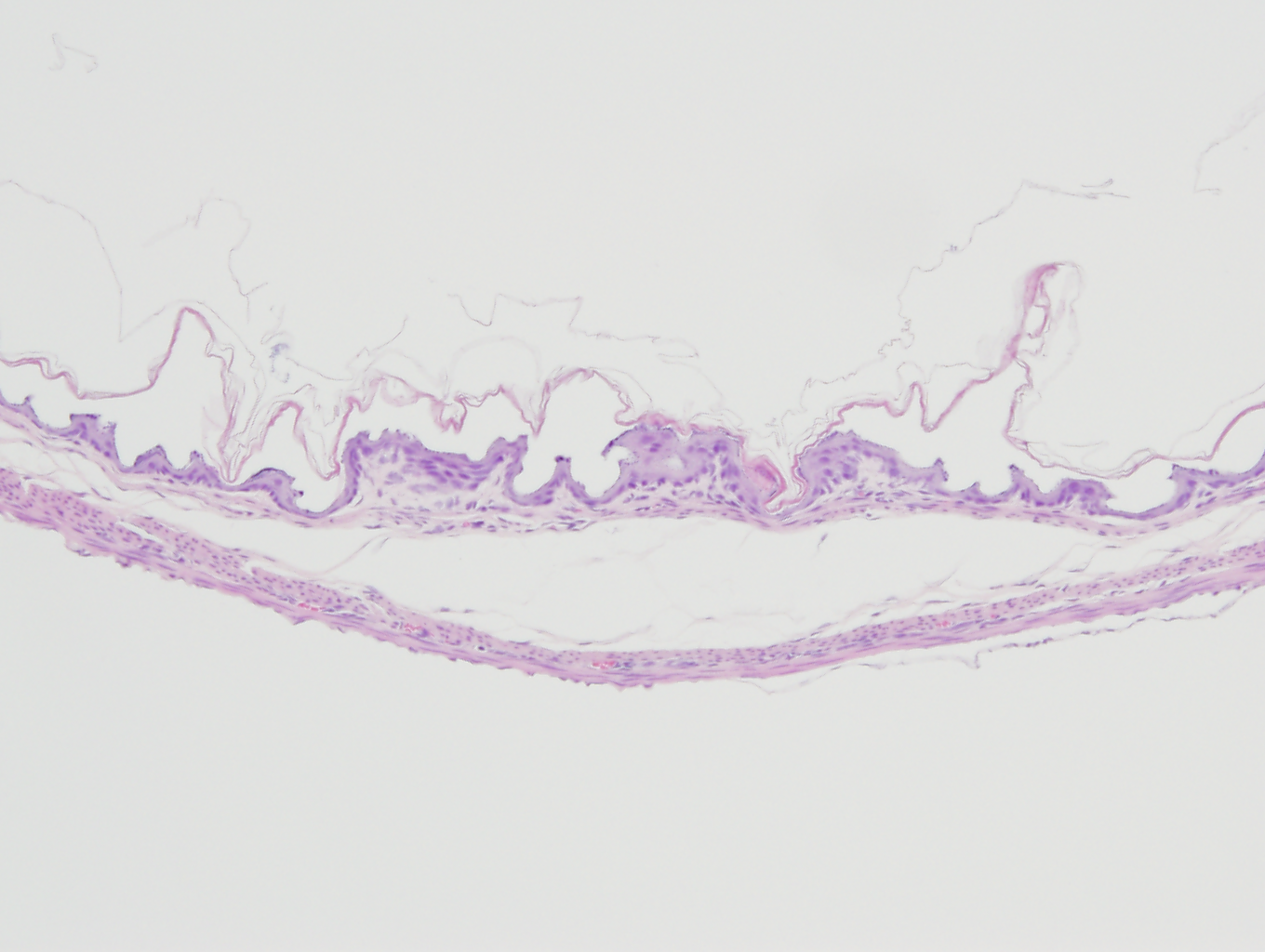

Supplement: Supplementary file 19 — Source Data for Figure 5 [file EMBJ-42-e113349-s009.zip › EMBOJ-2022-113349_SourceDataForFigure 5/5G/Forestomach_HE_6_ WT_X10mh.tif]

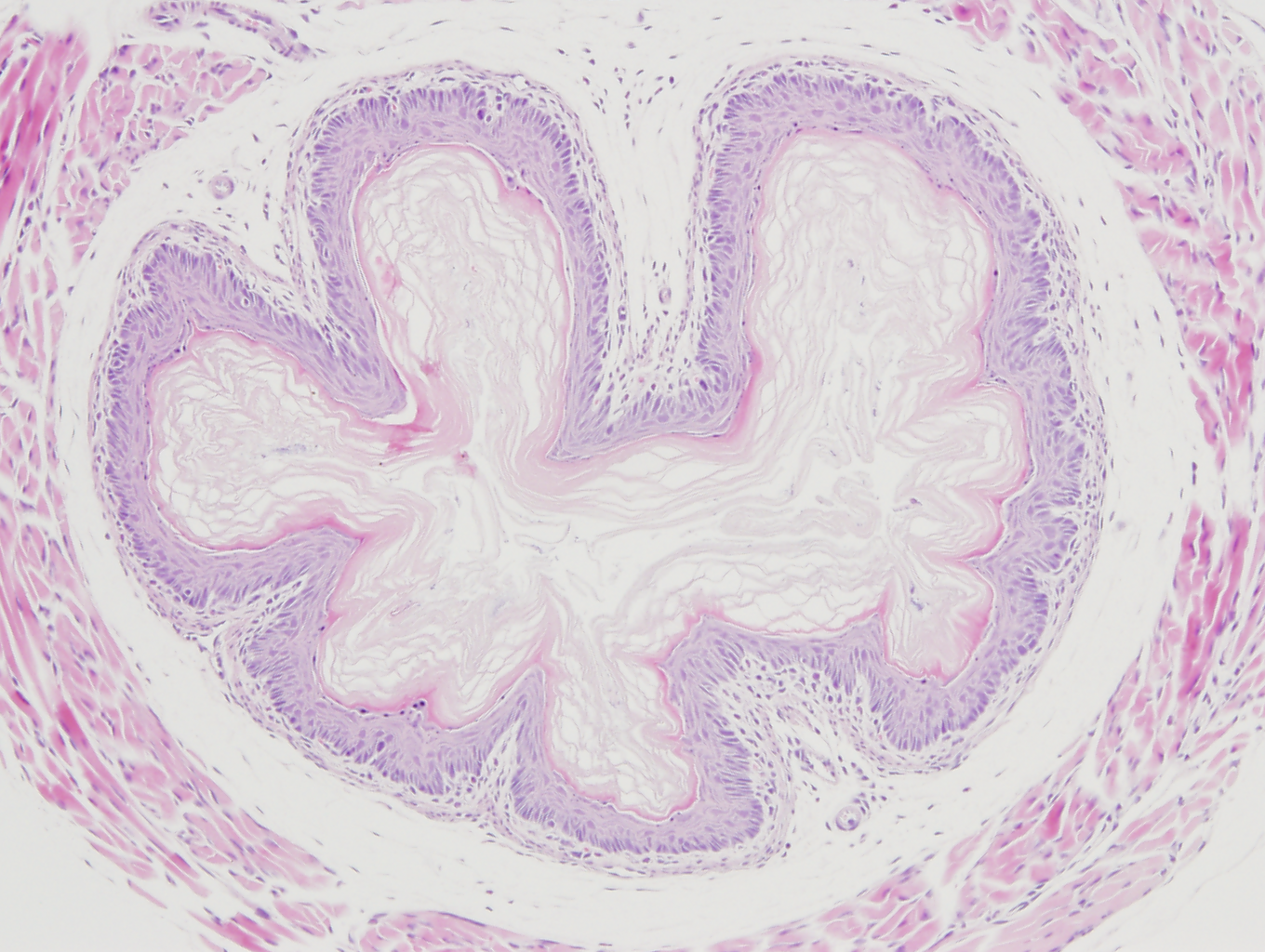

Supplement: Supplementary file 19 — Source Data for Figure 5 [file EMBJ-42-e113349-s009.zip › EMBOJ-2022-113349_SourceDataForFigure 5/5G/Esophagus_HE_7_ S531E_X10l.tif]

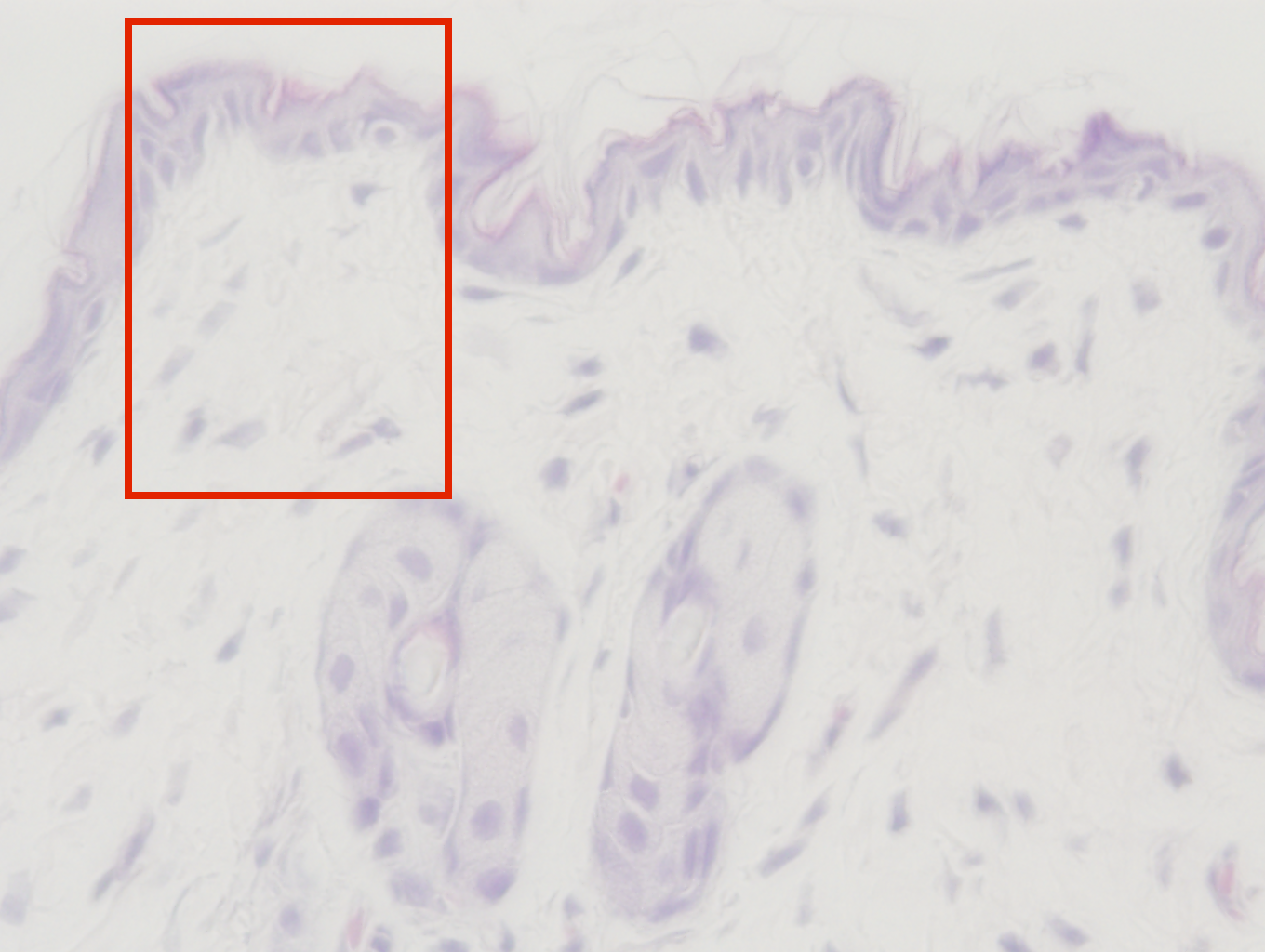

Supplement: Supplementary file 19 — Source Data for Figure 5 [file EMBJ-42-e113349-s009.zip › EMBOJ-2022-113349_SourceDataForFigure 5/5G/Skin_HE_6_ WT_X40mh_Marked.tif]

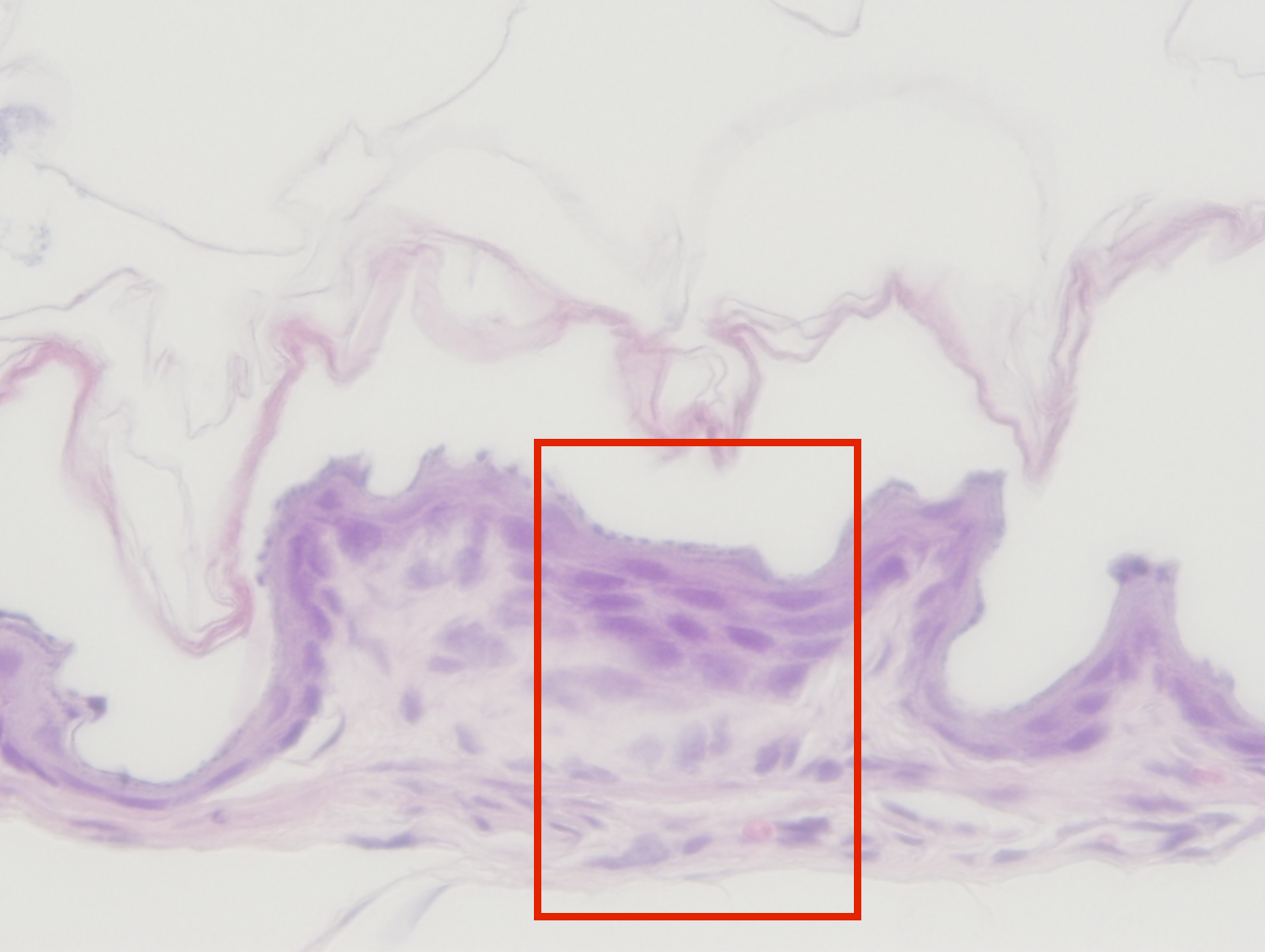

Supplement: Supplementary file 19 — Source Data for Figure 5 [file EMBJ-42-e113349-s009.zip › EMBOJ-2022-113349_SourceDataForFigure 5/5G/Forestomach_HE_6_ WT_X40mh_Marked.tif]

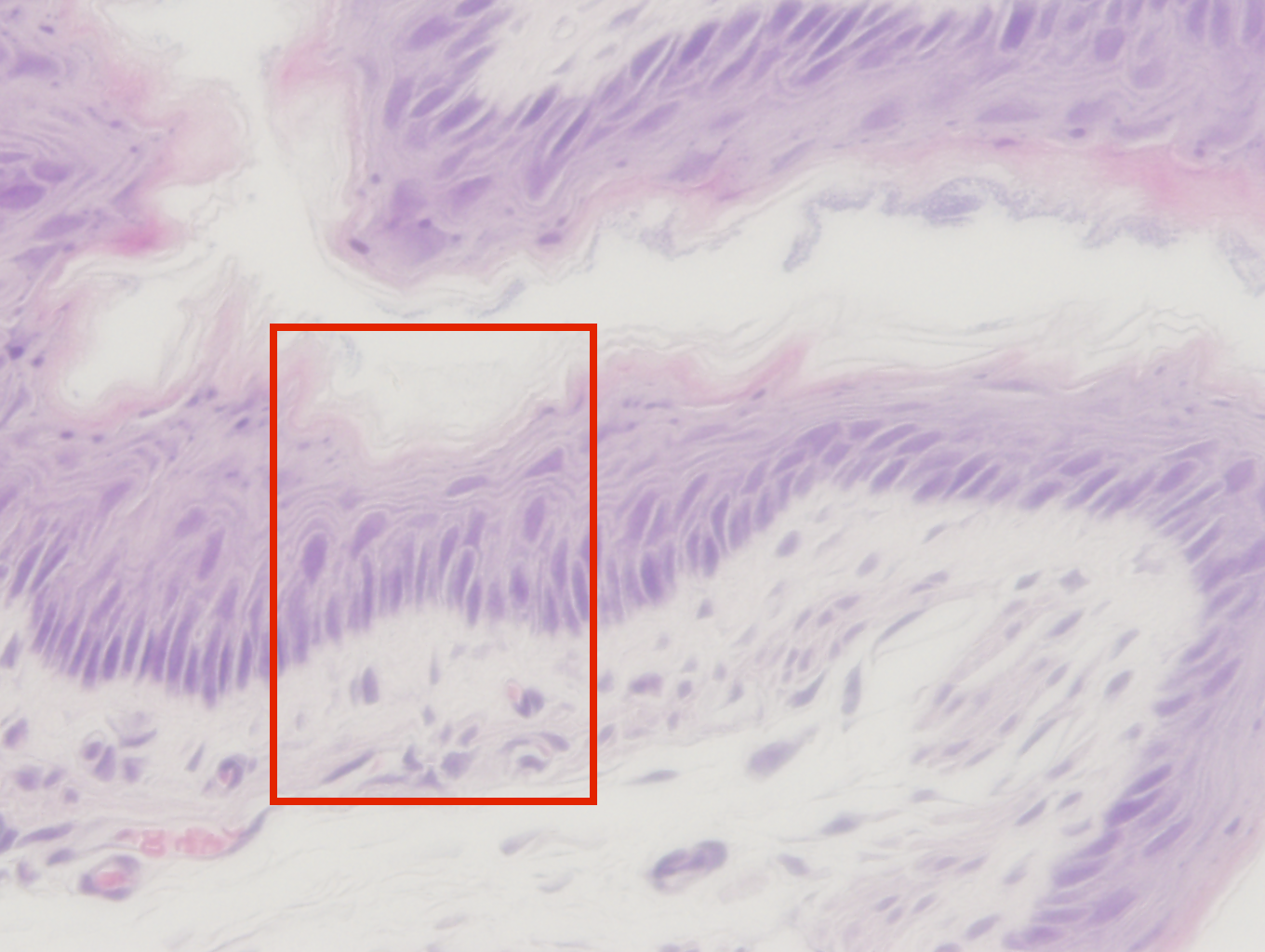

Supplement: Supplementary file 19 — Source Data for Figure 5 [file EMBJ-42-e113349-s009.zip › EMBOJ-2022-113349_SourceDataForFigure 5/5G/Esophagus_HE_6_ WT_X40m_Marked.tif]

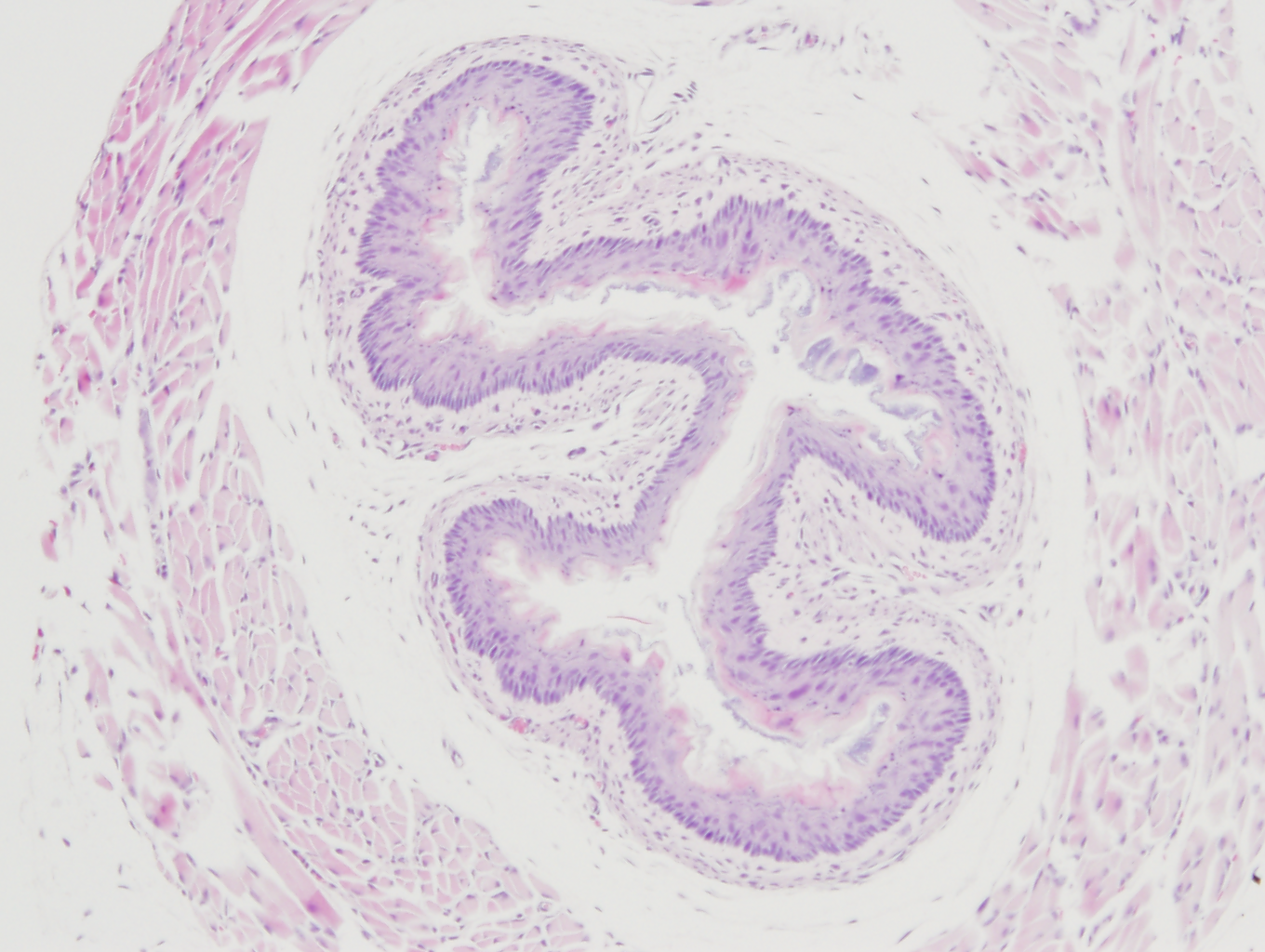

Supplement: Supplementary file 19 — Source Data for Figure 5 [file EMBJ-42-e113349-s009.zip › EMBOJ-2022-113349_SourceDataForFigure 5/5G/Esophagus_HE_6_ WT_X10m.tif]

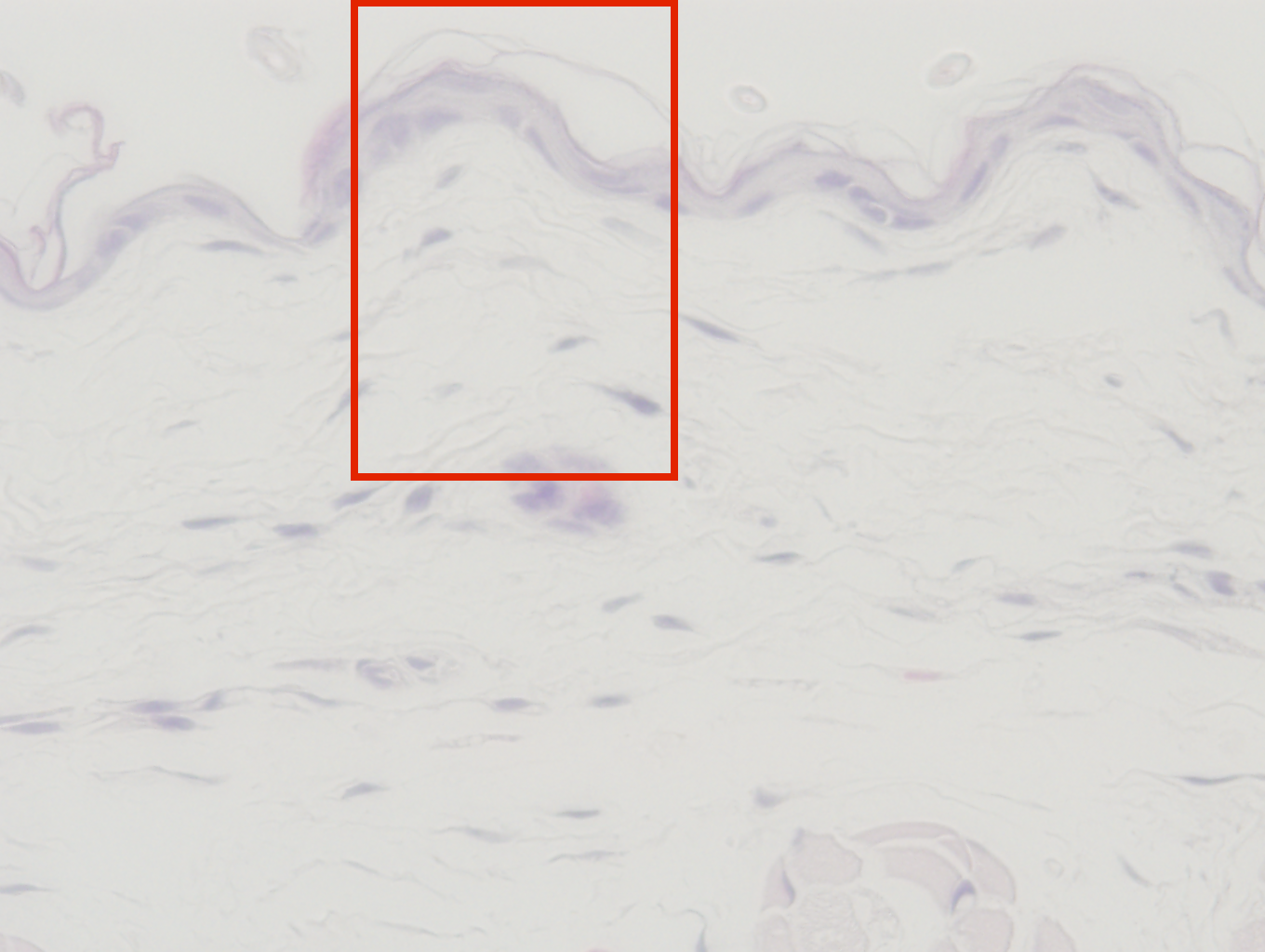

Supplement: Supplementary file 19 — Source Data for Figure 5 [file EMBJ-42-e113349-s009.zip › EMBOJ-2022-113349_SourceDataForFigure 5/5G/Skin_HE_7_ S351E_X40mh_Marked.tif]

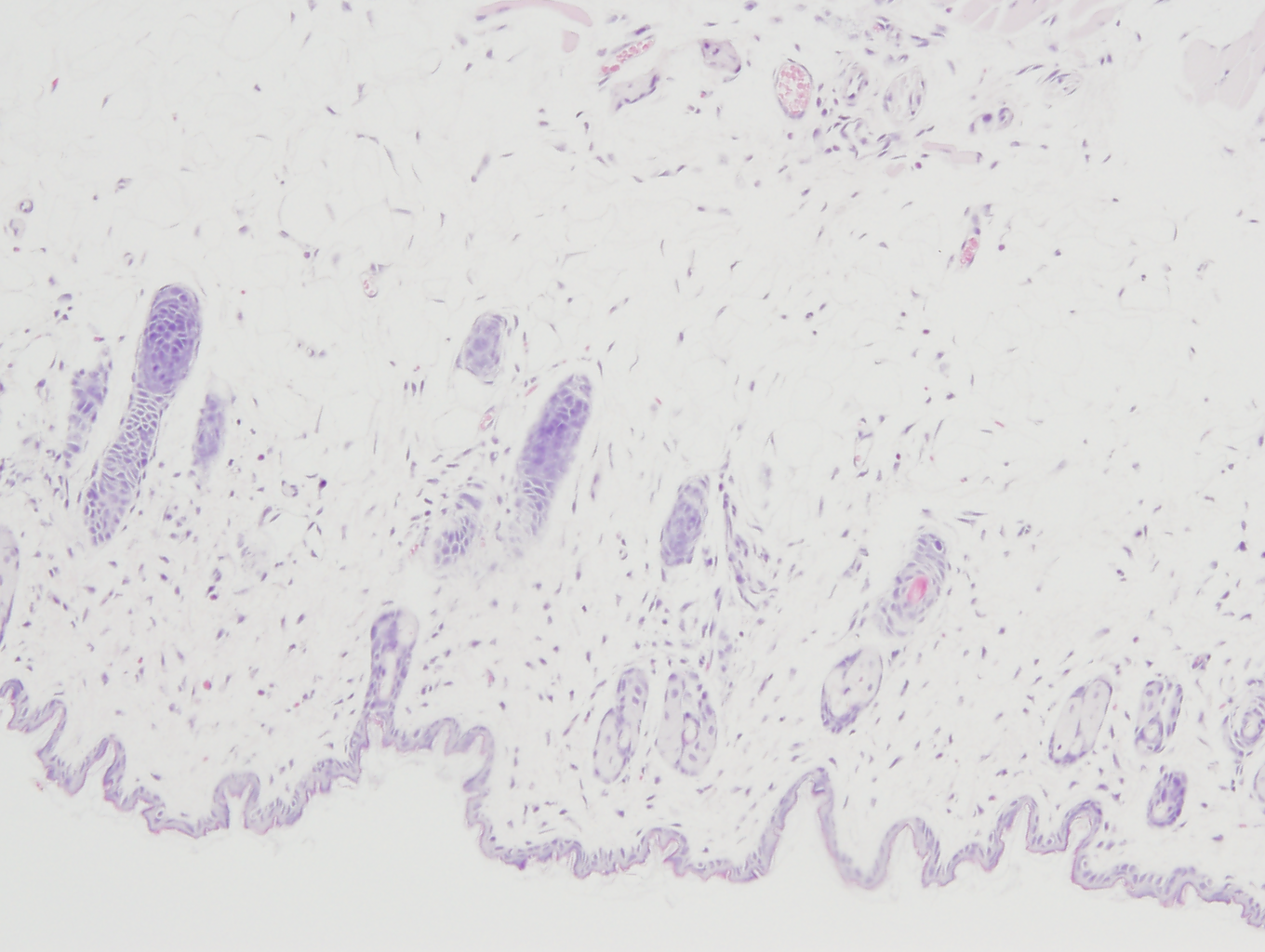

Supplement: Supplementary file 19 — Source Data for Figure 5 [file EMBJ-42-e113349-s009.zip › EMBOJ-2022-113349_SourceDataForFigure 5/5G/Skin_HE_6_ WT_X10mh.tif]

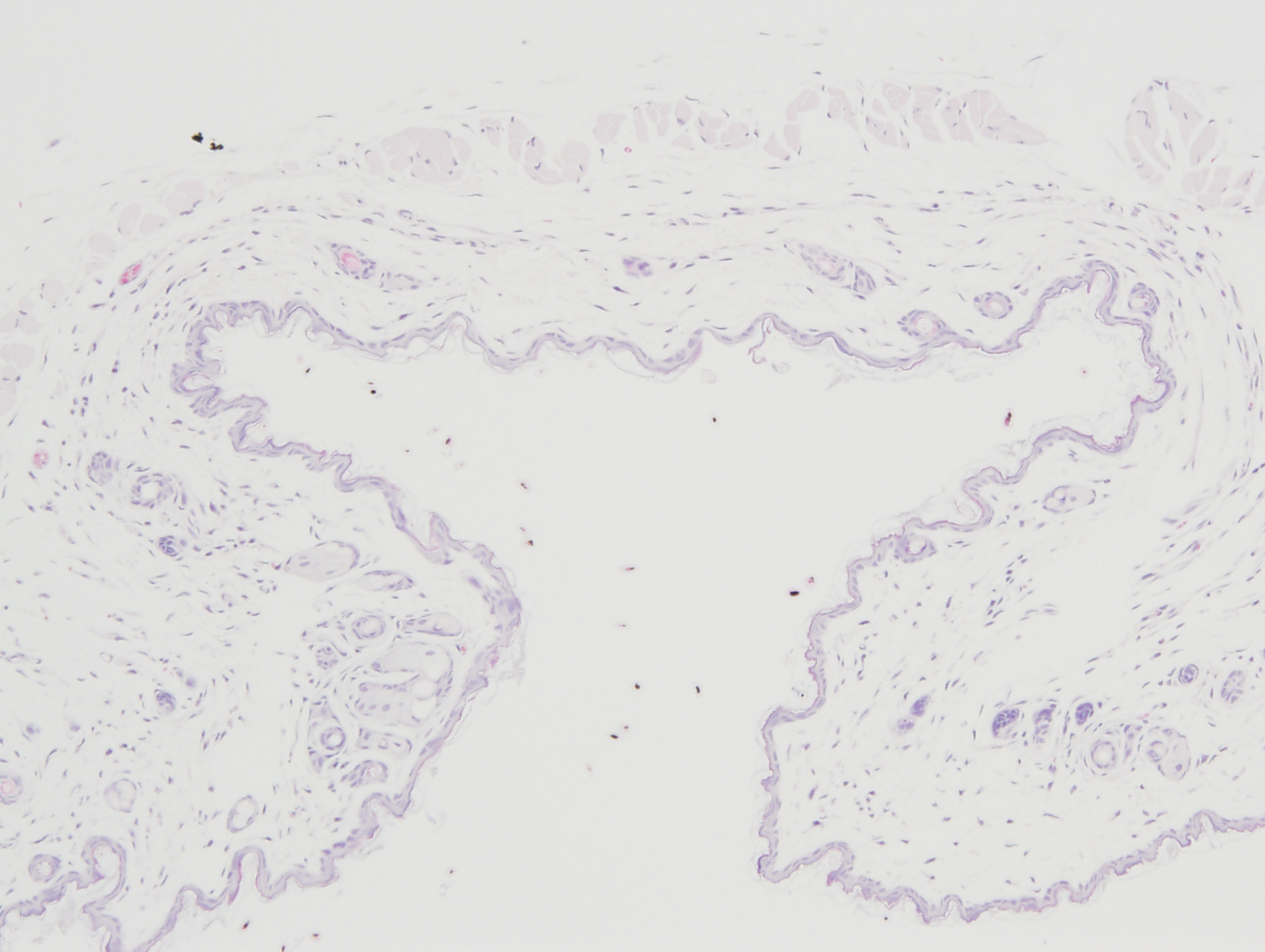

Supplement: Supplementary file 19 — Source Data for Figure 5 [file EMBJ-42-e113349-s009.zip › EMBOJ-2022-113349_SourceDataForFigure 5/5G/Skin_HE_7_ S351E_X10mh.tif]

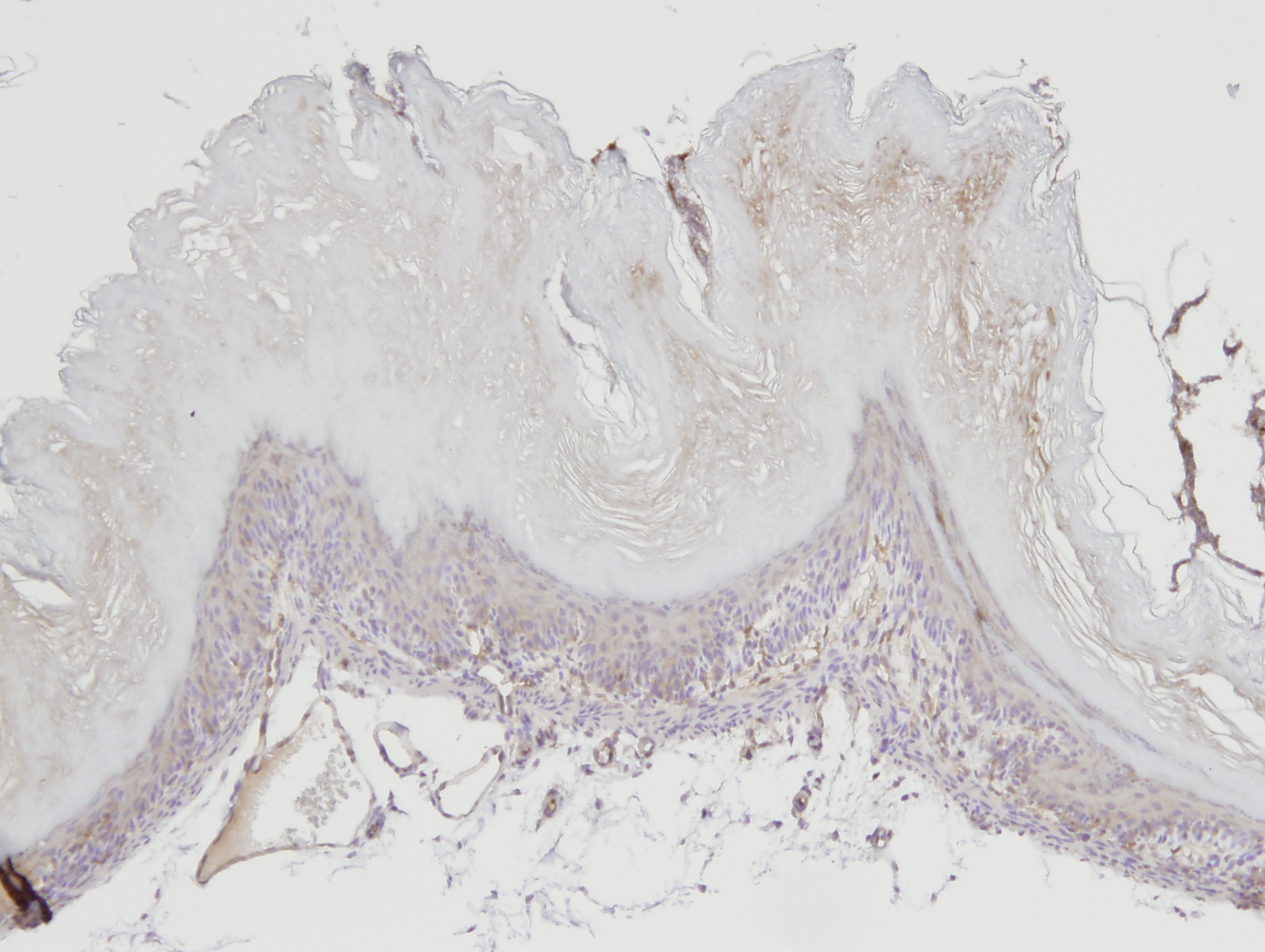

Supplement: Supplementary file 19 — Source Data for Figure 5 [file EMBJ-42-e113349-s009.zip › EMBOJ-2022-113349_SourceDataForFigure 5/5H/Forestomach_NQ01_7_ S351E_X10mh.tif]

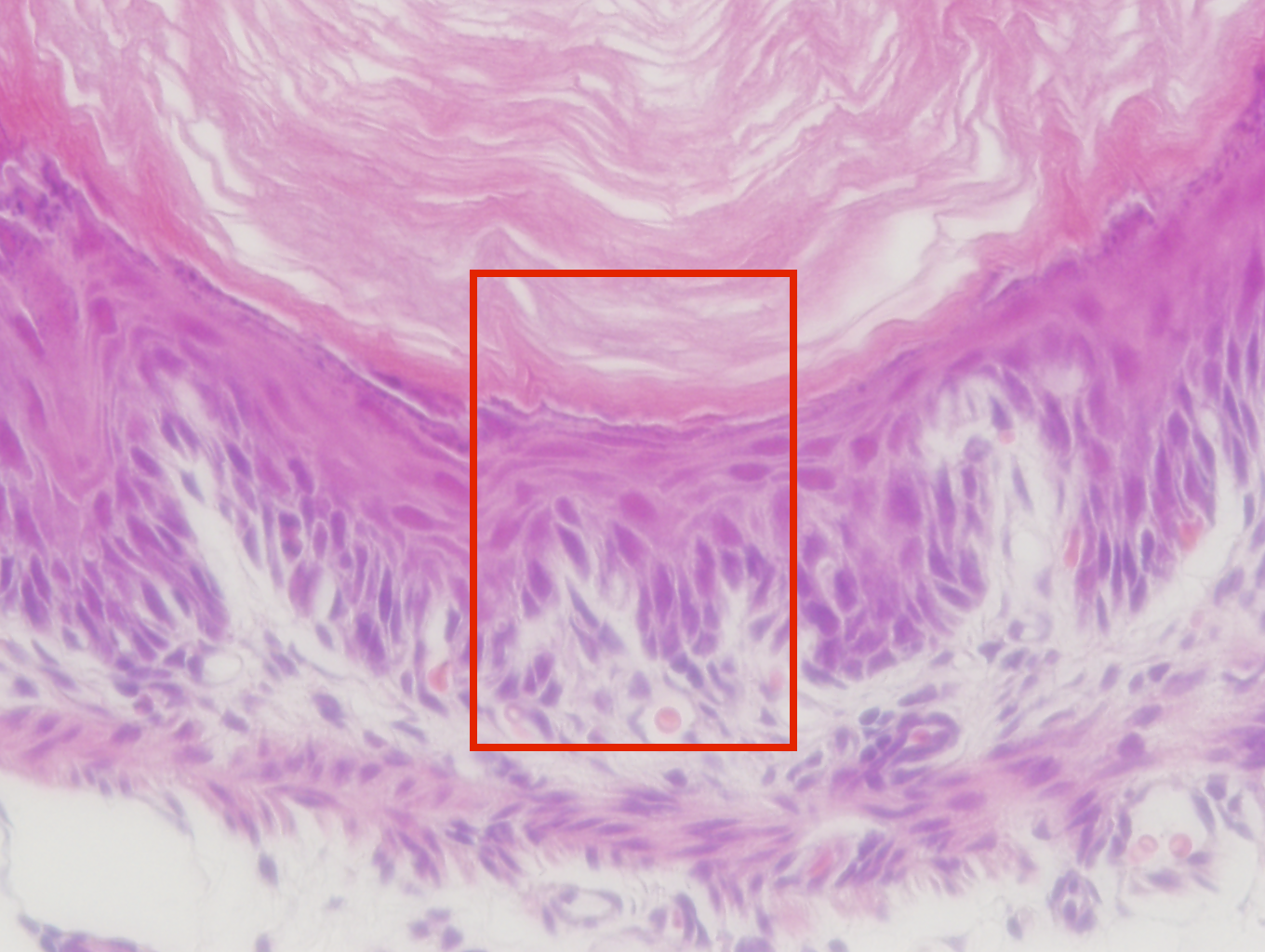

Supplement: Supplementary file 19 — Source Data for Figure 5 [file EMBJ-42-e113349-s009.zip › EMBOJ-2022-113349_SourceDataForFigure 5/5G/Forestomach_HE_7_ S351E_X40lh_Marked.tif]

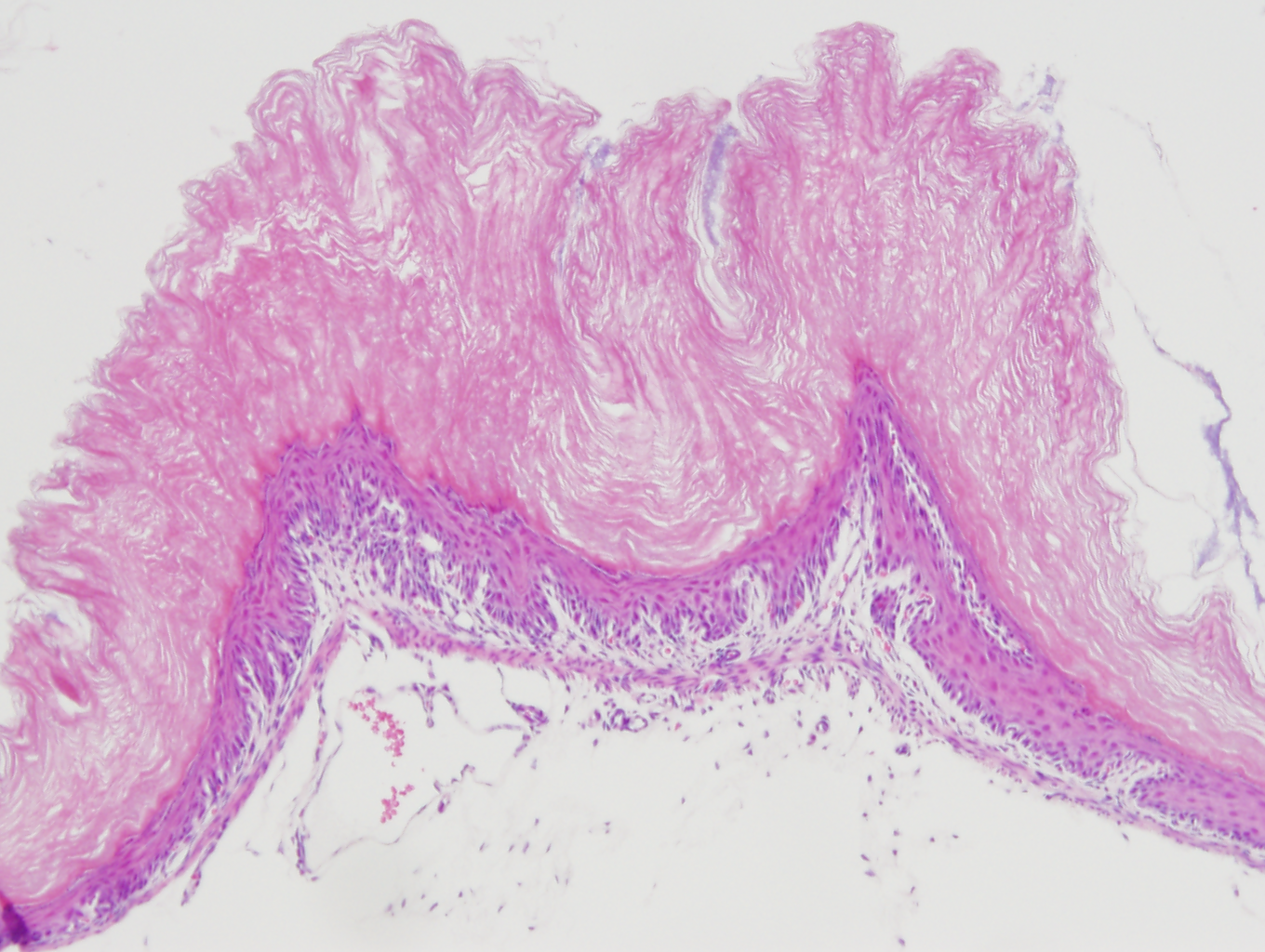

Supplement: Supplementary file 19 — Source Data for Figure 5 [file EMBJ-42-e113349-s009.zip › EMBOJ-2022-113349_SourceDataForFigure 5/5G/Forestomach_HE_7_ S351E_X10lh.tif]

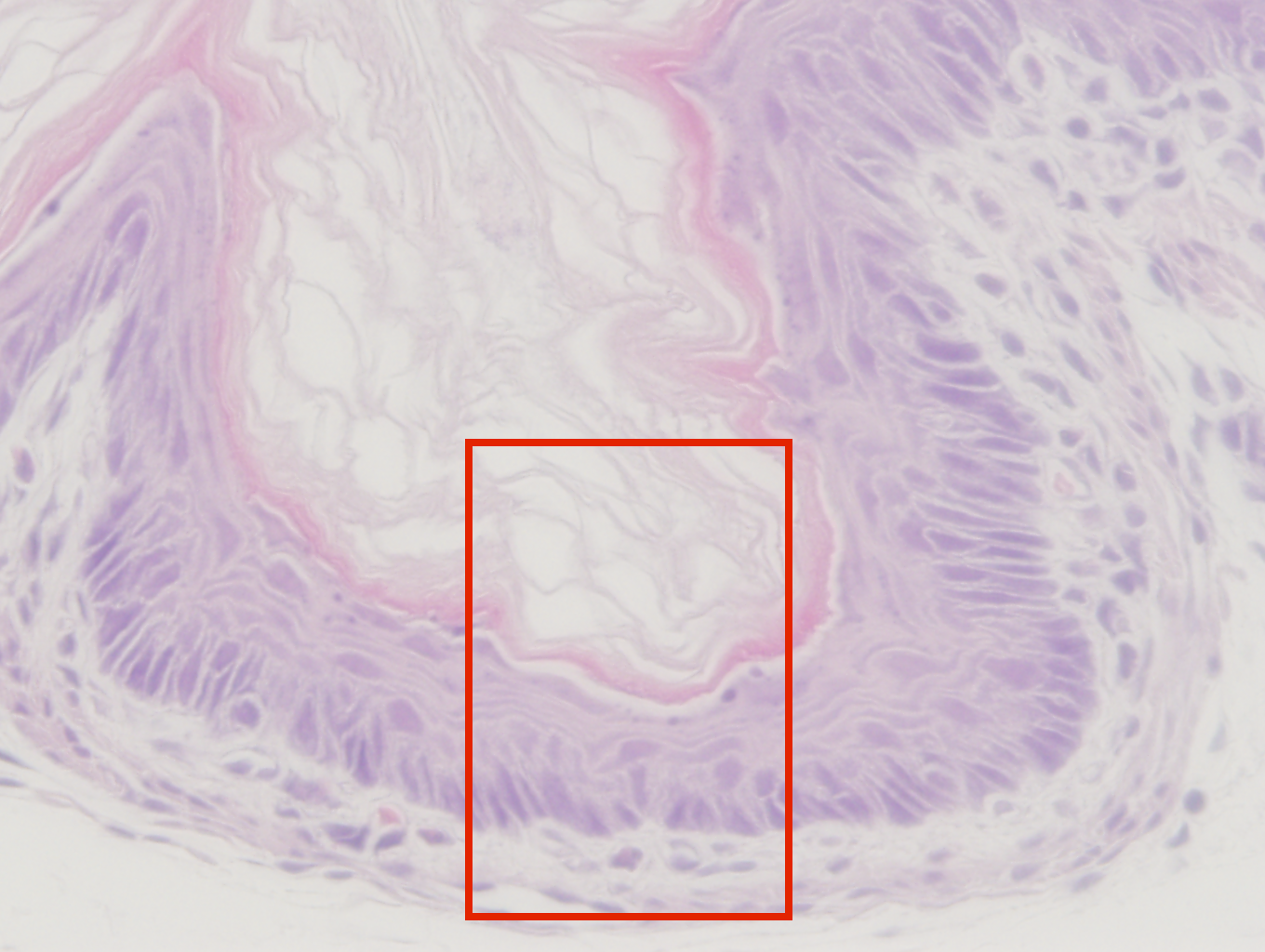

Supplement: Supplementary file 19 — Source Data for Figure 5 [file EMBJ-42-e113349-s009.zip › EMBOJ-2022-113349_SourceDataForFigure 5/5G/Esophagus_HE_7_ S531E_X40l_Marked.tif]

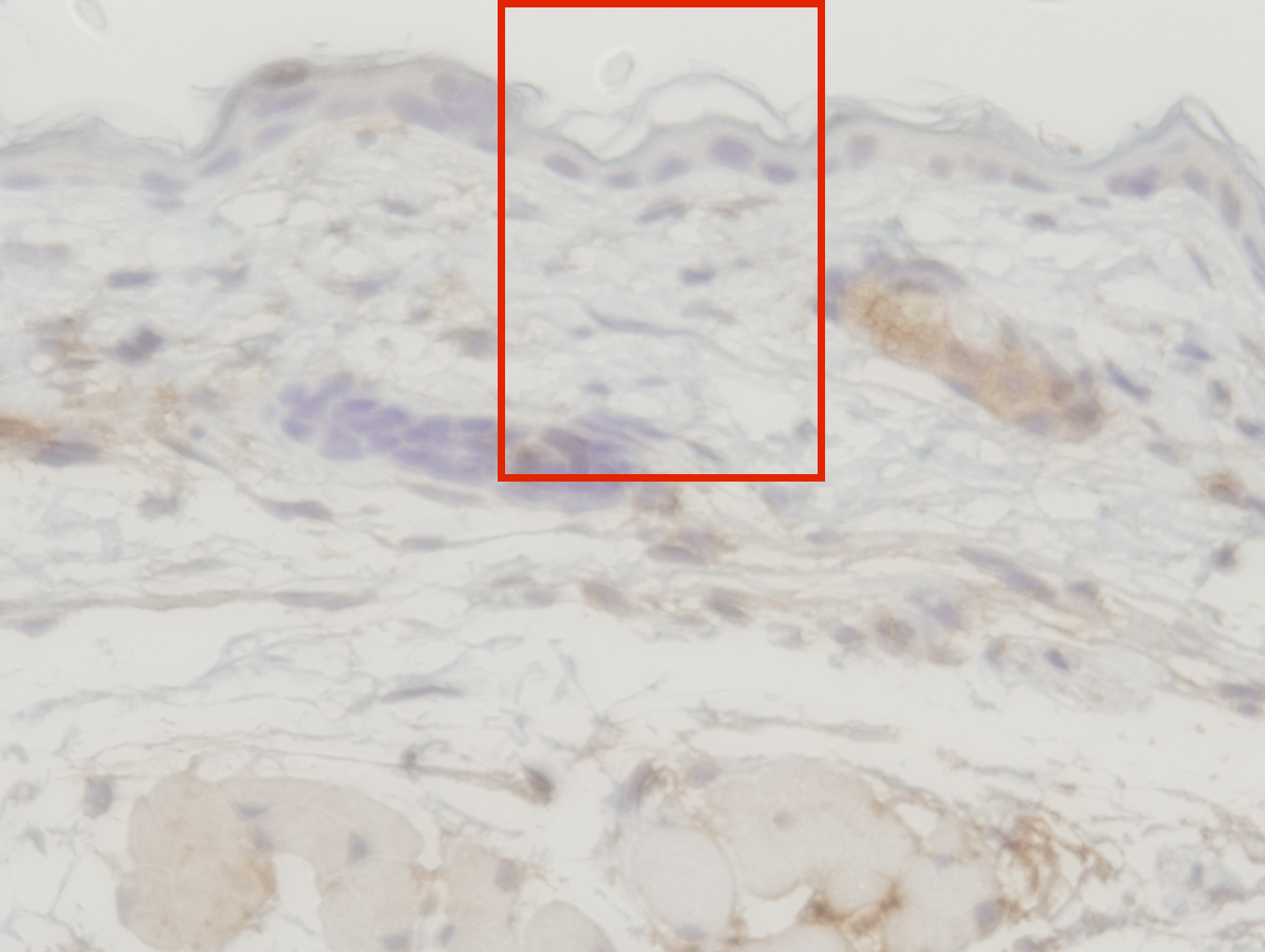

Supplement: Supplementary file 19 — Source Data for Figure 5 [file EMBJ-42-e113349-s009.zip › EMBOJ-2022-113349_SourceDataForFigure 5/5H/Skin_NQ01_7_ S351E_X40mh_Marked.tif]

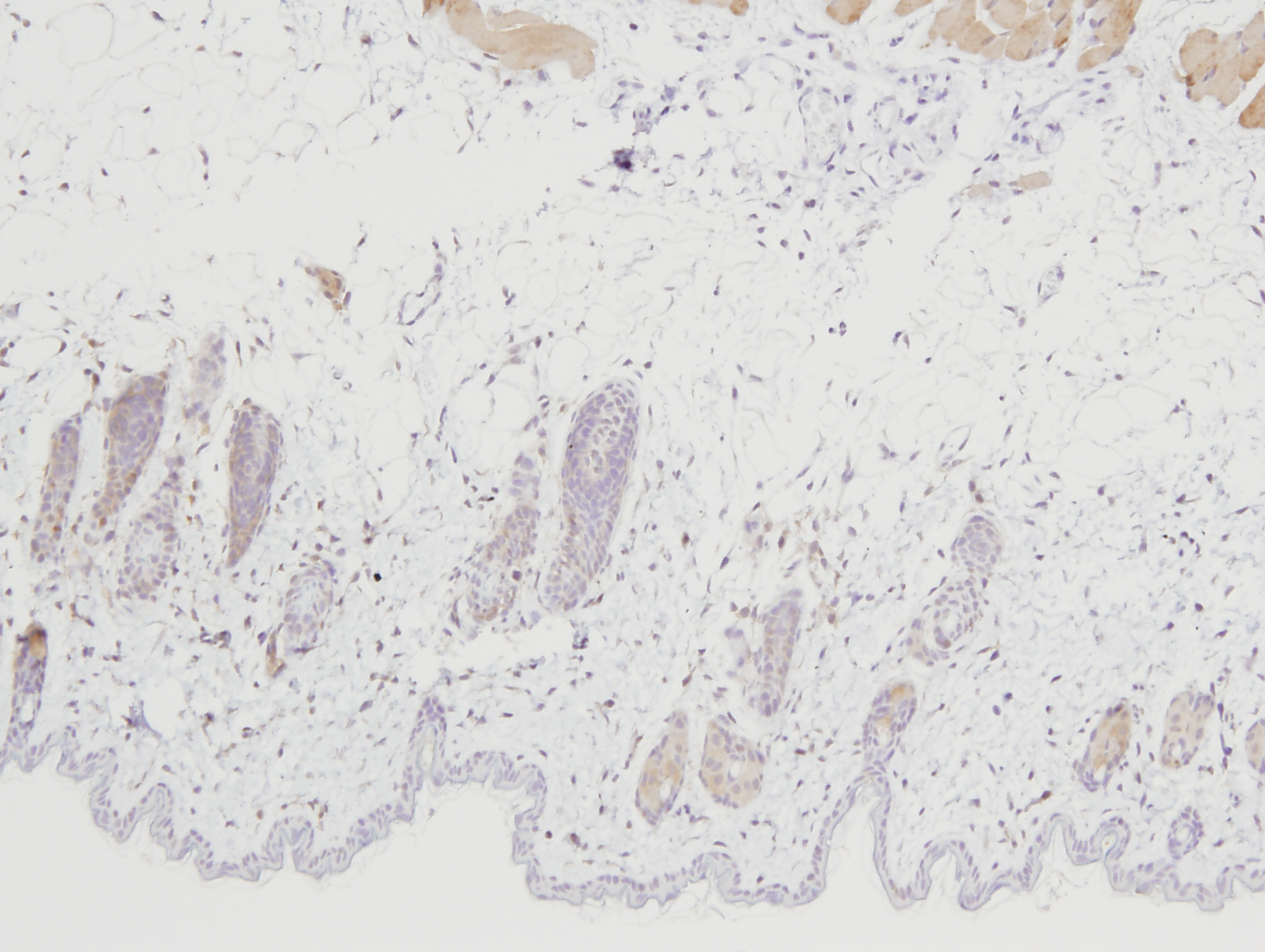

Supplement: Supplementary file 19 — Source Data for Figure 5 [file EMBJ-42-e113349-s009.zip › EMBOJ-2022-113349_SourceDataForFigure 5/5H/Skin_NQ01_6_ WT_X10mh.tif]
