# Supplementary material for: Phosphorylation of phase‐separated p62 bodies by ULK1 activates a redox‐independent stress response
Source: EMBO J. 2023 Jun 12;42(14):e113349. doi: 10.15252/embj.2022113349 (PMC10350833; doi:10.15252/embj.2022113349)
Supplement: Supplementary file 19 — Source Data for Figure 5 [file EMBJ-42-e113349-s009.zip › EMBOJ-2022-113349_SourceDataForFigure 5/5H/README_Fig 5h_230214.docx]

Source files for Figure 5h

For high magnification pictures, cropped regions were used for generating the figure. Brightness/contrast of each figure was adjusted using the Photoshop software.
