# Supplementary material for: Phosphorylation of phase‐separated p62 bodies by ULK1 activates a redox‐independent stress response
Source: EMBO J. 2023 Jun 12;42(14):e113349. doi: 10.15252/embj.2022113349 (PMC10350833; doi:10.15252/embj.2022113349)
Supplement: Supplementary file 20 [file EMBJ-42-e113349-s017.pdf]

THE EMBO JOURNAL

Phosphorylation of phase-separated p62 bodies by ULK1 activates a redox-independent stress response

Masaaki Komatsu, Ryo Ikeda, Daisuke Noshiro, Hideaki Morishita, Shuhei Takada, Shun Kageyama, Yuko Fujioka, Tomoko Funakoshi, Satoko Komatsu-Hirota, Ritsuko Arai, Elena Ryzhii, Manabu Abe, Hozumi Motohashi, Tomoaki Koga, Mitsuyoshi Nakao, Kenji Sakimura, Arata Horii, Satoshi Waguri, Yoshinobu Ichimura, and Nobuo Noda
DOI: 10.15252/embj.2022113349

Corresponding author(s): Masaaki Komatsu (mkomatsu@juntendo.ac.jp) , Yoshinobu Ichimura (yichimu@juntendo.ac.jp), Nobuo Noda (nn@igm.hokudai.ac.jp)

Review Timeline:

Submission Date:	20th Dec 22
Editorial Decision:	31st Jan 23
Revision Received:	17th Apr 23
Editorial Decision:	28th Apr 23
Revision Received:	2nd May 23
Accepted:	10th May 23

Editor: William Teale

Transaction Report:

(Note: With the exception of the correction of typographical or spelling errors that could be a source of ambiguity, letters and reports are not edited. Depending on transfer agreements, referee reports obtained elsewhere may or may not be included in this compilation. Referee reports are anonymous unless the Referee chooses to sign their reports.)

Dear Masaaki,

Thank you again for the submission of your manuscript entitled "Phosphorylation of phase-separated p62 bodies by ULK1 activates a redox-independent stress response" (EMBOJ-2022-113349) and for your patience during the review process. We have now received the reports from the referees, which I copy to the bottom of this email.

As you can see from their comments, while the referees are broadly supportive of publication, all of them propose modifications to certain experiments and ways in which conclusions may be either efficiently extended or provided with additional support. These comments will require your attention before your manuscript can be published in The EMBO Journal.

Based on the overall interest expressed in the reports, I would like to invite you to address the comments of all referees in a revised version of the manuscript. I should add that it is The EMBO Journal policy to allow only a single major round of revision and that it is therefore important to resolve the main concerns at this stage. Please, follow the instructions below when preparing your manuscript for resubmission. Please let me know if you would like to go through any of the referees' comments with me. I would be a pleasure for me to discuss your work with you over Zoom.

I would also like to point out that as a matter of policy, competing manuscripts published during this period will not be taken into consideration in our assessment of the novelty presented by your study ("scooping" protection). We have extended this 'scooping protection policy' beyond the usual 3 month revision timeline to cover the period required for a full revision to address the essential experimental issues. Please contact me if you see a paper with related content published elsewhere to discuss the appropriate course of action.

When preparing your letter of response to the referees' comments, please bear in mind that this will form part of the Review Process File, and will therefore be available online to the community. For more details on our Transparent Editorial Process, please visit our website: https://www.embopress.org/page/journal/14602075/authorguide#transparentprocess

Again, please contact me at any time during revision if you need any help or have further questions.

Thank you very much again for the opportunity to consider your work for publication. I look forward to your revision.

Best regards,

William

William Teale, Ph.D.
Editor
The EMBO Journal

When submitting your revised manuscript, please carefully review the instructions below and include the following items:

1) a .docx formatted version of the manuscript text (including legends for main figures, EV figures and tables). Please make sure that the changes are highlighted to be clearly visible.

2) individual production quality figure files as .eps, .tif, .jpg (one file per figure).

3) a .docx formatted letter INCLUDING the reviewers' reports and your detailed point-by-point response to their comments. As part of the EMBO Press transparent editorial process, the point-by-point response is part of the Review Process File (RPF), which will be published alongside your paper.

4) a complete author checklist, which you can download from our author guidelines (https://wol-prod-cdn.literatumonline.com/pb-assets/embo-site/Author Checklist%20-%20EMBO%20J-1561436015657.xlsx). Please insert information in the checklist that is also reflected in the manuscript. The completed author checklist will also be part of the RPF.

5) Please note that all corresponding authors are required to supply an ORCID ID for their name upon submission of a revised manuscript.

6) We require a 'Data Availability' section after the Materials and Methods. Before submitting your revision, primary datasets produced in this study need to be deposited in an appropriate public database, and the accession numbers and database listed under 'Data Availability'. Please remember to provide a reviewer password if the datasets are not yet public (see https://www.embopress.org/page/journal/14602075/authorguide#datadeposition). If no data deposition in external databases is

needed for this paper, please then state in this section: This study includes no data deposited in external repositories. Note that the Data Availability Section is restricted to new primary data that are part of this study.

Note - All links should resolve to a page where the data can be accessed.

7) When assembling figures, please refer to our figure preparation guideline in order to ensure proper formatting and readability in print as well as on screen:
http://bit.ly/EMBOPressFigurePreparationGuideline

Please remember: Digital image enhancement is acceptable practice, as long as it accurately represents the original data and conforms to community standards. If a figure has been subjected to significant electronic manipulation, this must be noted in the figure legend or in the 'Materials and Methods' section. The editors reserve the right to request original versions of figures and the original images that were used to assemble the figure.

8) For data quantification: please specify the name of the statistical test used to generate error bars and P values, the number (n) of independent experiments (specify technical or biological replicates) underlying each data point and the test used to calculate p-values in each figure legend. The figure legends should contain a basic description of n, P and the test applied. Graphs must include a description of the bars and the error bars (s.d., s.e.m.).

9) We would also encourage you to include the source data for figure panels that show essential data. Numerical data can be provided as individual .xls or .csv files (including a tab describing the data). For 'blots' or microscopy, uncropped images should be submitted (using a zip archive or a single pdf per main figure if multiple images need to be supplied for one panel). Additional information on source data and instruction on how to label the files are available at .

10) We replaced Supplementary Information with Expanded View (EV) Figures and Tables that are collapsible/expandable online (see examples in https://www.embopress.org/doi/10.15252/embj.201695874). A maximum of 5 EV Figures can be typeset. EV Figures should be cited as 'Figure EV1, Figure EV2" etc. in the text and their respective legends should be included in the main text after the legends of regular figures.

- For the figures that you do NOT wish to display as Expanded View figures, they should be bundled together with their legends in a single PDF file called *Appendix*, which should start with a short Table of Content. Appendix figures should be referred to in the main text as: "Appendix Figure S1, Appendix Figure S2" etc. See detailed instructions regarding expanded view here: .

- Additional Tables/Datasets should be labelled and referred to as Table EV1, Dataset EV1, etc. Legends have to be provided in a separate tab in case of .xls files. Alternatively, the legend can be supplied as a separate text file (README) and zipped together with the Table/Dataset file.

11) At EMBO Press we ask authors to provide source data for the main manuscript figures. Our source data coordinator will contact you to discuss which figure panels we would need source data for and will also provide you with helpful tips on how to upload and organize the files.

12) Our journal encourages inclusion of *data citations in the reference list* to directly cite datasets that were re-used and obtained from public databases. Data citations in the article text are distinct from normal bibliographical citations and should directly link to the database records from which the data can be accessed. In the main text, data citations are formatted as follows: "Data ref: Smith et al, 2001" or "Data ref: NCBI Sequence Read Archive PRJNA342805, 2017". In the Reference list, data citations must be labeled with "[DATASET]". A data reference must provide the database name, accession number/identifiers and a resolvable link to the landing page from which the data can be accessed at the end of the reference. Further instructions are available at .

Additional instructions for preparing your revised manuscript:

Please make sure you upload a letter of response to the referees' comments together with the revised manuscript.

Please also check that the title and abstract of the manuscript are brief, yet explicit, even to non-specialists.

When assembling figures, please refer to our figure preparation guideline in order to ensure proper formatting and readability in print as well as on screen:
https://bit.ly/EMBOPressFigurePreparationGuideline
See also guidelines for figure legends: https://www.embopress.org/page/journal/14602075/authorguide#figureformat

At EMBO Press we ask authors to provide source data for the main manuscript figures. Our source data coordinator will contact

you to discuss which figure panels we would need source data for and will also provide you with helpful tips on how to upload and organize the files.

IMPORTANT: When you send the revision we will require
- a point-by-point response to the referees' comments, with a detailed description of the changes made (as a word file).
- a word file of the manuscript text.
- individual production quality figure files (one file per figure)
- a complete author checklist, which you can download from our author guidelines (https://www.embopress.org/page/journal/14602075/authorguide).
- Expanded View files (replacing Supplementary Information)
Please see out instructions to authors
https://www.embopress.org/page/journal/14602075/authorguide#expandedview

Please remember: Digital image enhancement is acceptable practice, as long as it accurately represents the original data and conforms to community standards. If a figure has been subjected to significant electronic manipulation, this must be noted in the figure legend or in the 'Materials and Methods' section. The editors reserve the right to request original versions of figures and the original images that were used to assemble the figure.

Further information is available in our Guide For Authors: https://www.embopress.org/page/journal/14602075/authorguide

We realize that it is difficult to revise to a specific deadline. In the interest of protecting the conceptual advance provided by the work, we recommend a revision within 3 months (1st May 2023). Please discuss the revision progress ahead of this time with the editor if you require more time to complete the revisions. Use the link below to submit your revision:

https://emboj.msubmit.net/cgi-bin/main.plex

Referee #1:

NRF2 is a transcription fact, regulating a cellular defense system. Under normal conditions, the primary E3 ubiquitin ligase complex (KEAP1-Culline 3-Rbx) constantly targets NRF2 for ubiquitylation and degradation by the26 S proteasome. Under oxidative stress, cysteine residues in KEAP1 sense the redox imbalance, leading to diminished ubiquitylation and thus the stabilization of NRF2. Following the discovery of this redox-dependent NRF2 activation, another non-canonical mechanism of NRF2 activation was discovery, which involves p62. Therefore, although the concept of p62 phosphorylation and recruitment of KEAP1 in p62 bodies is not novel, but the authors delivered several important points. (1) the authors have demonstrated that ULK1 is the kinase for Serine 349 of p62 both in vitro and in vivo. (2) direct interaction of p62 with ULK1 is demonstrated. (3) The knock-in mouse with a phosphomimetic mutation at Ser349, but not those with a phosphodefective mutation, exhibits the persistent activation of NRF2, which phenocopies the systemic KEAP1 knockout mice, i.e. these mice suffered hyperkeratosis and consequently obstruction of the esophagus and forestomach, as well as growth retardation due to malnutrition (death at day 3 in KEAP1 KO mice). This is the first in vivo study demonstrating the importance of p62 phosphorylation at ser 349 in controlling the NRF2 pathway in whole animal. It is not a trivial task. Furthermore, the experiments are well-designed, and the data are in high quality, I suggest acceptance of the manuscript after a minor revision.
It would be interesting to perform data analyses dissecting mutation status in p62 (especially serine 349), ULK1 and KEAP1, and correlation the mutation status of these proteins with the NRF2 expression level, sensitivity of the cancer cells to therapeutic drugs, as well as patient prognosis.
Fig. 2b: For ULK1 association with p62 in the droplet form. It would be better to include a negative control, such as an irrelevant kinase.
Fig. 2e: similar to Fig. 2b, an irrelevant kinase as a negative control will make the colocalization of ULK1 with p62 bodies more meaningful/convincing.
Fig. 4C Have the authors performed the same experiment using p62 WT and p62-S349E, rather than adding S349E to S403E S407E? is the p62-S349E alone sufficient to change the biophysical properties of p62 bodies?

Referee #2:

In this manuscript Ikeda et al. explores redox-independent NRF2 activation through the formation of p62 bodies which contain S349 phosphorylated p62. The authors show ULK1 to be an important kinase responsible for this phosphorylation and shows that the phosphorylation at S349 is important to retain KEAP1 in the p62 body which in turn allows NRF2 activation. Heterozygous phosphomimetic p62S351E/+ knock-in mice phenocopy KEAP1 knockouts while the phosphor-deficient

p62S351A has no obvious effects. It is an engaging study where the authors have discovered and described a regulatory role of ULK1 in p62 bodies on a molecular level and also have convincing in vivo data from p62 phosphomutant mice, indicating physiological relevance of the finding. With some experiments and changes I find the manuscript very suitable for publication in EMBO J.

Suggested changes to improve the manuscript:
• In their experiment in fig 1f-where the authors show the in vitro phosphorylation of p62 by ULK1 and Atg1 it will be helpful to show on the blots with arrows if there are dimers formed. There are several unexplained bands upon blotting for p62 (pS349). The authors may add a few lines to specify clearly if formation of dimers are affected by phosphorylation at S349.
• How does the S349 phosphorylation affect LC3 binding, Ub binding, p62 and KEAP1 levels in cells? The mice data in fig 5f suggests it may affect KEAP1 levels.
• Previous papers have identified several ULK1 mediated phosphorylation sites in the UBA domain of p62. How do these phosphorylations affect the phosphorylation by ULK1 at S349 and vice versa? Repeating the in vitro phosphorylation assay in Fig1f with phospho-defective mutants may help to understand if and how they are influenced by each other.
• Does the S349E mutation have any effect on p62 condensate formation in the assay shown in Figure 2A, ULK1 localisation to condensates (as in fig 2B)?
• Colocalization of KEAP1 to condensates should be added to the analysis of fig4A in addition to the circularity of condensates shown in fig 4B. This may support the role of S349 phosphorylation in retaining KEAP1 in p62 bodies.
• The data in figure 4C shows clearly how the morphology of the condensates change in the triple mutant p62(S349ES403ES407E). However, the morphology of S349E by itself is missing.

Minor comments
• Typing error at line 181 -KEPA instead of KEAP
• Atg1 should be consistently in lowercase in Figures/manuscript to keep nomenclature uniform for mammalian and yeast proteins
• Figure 4b: axis label is missing
• Are there any nonspecific bands in the KEAP blot in fig 5F? if so then please use * to clarify the non-specific band.

Referee #3:

Synopsis
In their manuscript, Ikeda et al describe an elegant study, in which they look at the consequence and the impact of the direct interaction between the autophagy cargo adaptor protein p62/SQSTM1 and the major autophagy-specific kinase ULK1 that phosphorylates human p62/SQSTM1 on Ser349 (amongst other sites). This post-translational modification of p62/SQSTM1 increases its interaction with the E3 ligase KEAP1 responsible for the tonic degradation of the transcription factor NRF2 involved in oxidative stress response. Stronger binding between p62/SQSTM1 segregates KEAP1 in p62/SQSTM1 bodies leading to KEAP1 degradation and consequently NRF2 stabilization and activation under normal conditions. Permanent increase in KEAP1 binding and its degradation in a p62/SQSTM1-dependent fashion has a profound effect on mouse development, underscoring the pivotal role the ULK1-p62-KEAP1-NRF2 pathway plays in the maintenance of the organismal homeostasis. The study uses advanced technology, and its clear strength lies in demonstrating the consequence of the gain-of-function mutation in p62/SQSTM1 in the mice in vivo. Below, I am listing several points whose resolution could help further strengthen the quality of this interesting paper.

Specific comments
1. Interaction of Atg1/ULK1 with p62/SQSTM1
• Using purified proteins and high-speed atomic force microscopy, the authors demonstrated a direct, dynamic interaction between truncated p62/SQSTM1 and ULK1 (also the yeast homologue Atg1). The interaction is proposed to be driven by the intrinsically disordered regions (IDRs) of the respective proteins (Atg1/ULK1 and p62/SQSTM1) and possibly other regions. The spectacular video sequences suggest the transient nature of the described interactions. Is that at all possible to determine the Kd values for those binding pairs using a more classical biochemical method? Since p62/SQSTM1 also binds FIP200 directly via its FIP200-interacting region, it would seem to be important to know the relative contribution of ULK1:p62/SQSTM1 vs. ULK1:FIP200:p62/SQSTM1 complexes. Knowing affinities of the interactions, hypothesis generation would be made possible.
• In addition, the paper would benefit from a multiple alignment of Atg1/ULK1/ULK2 proteins to discuss which conserved sequences/motifs may drive the direct binding between p62/SQSTM1 and the kinases of the Atg1 family.

2. Use of a small molecule inhibitor to probe for the functional significance of ULK1 in p62/SQSTM1 bodies
• The authors made use of the published ULK1/2 inhibitor MRT68921 to investigate the role of the ULK1/2 catalytical activity in the formation of p62/SQSTM1 bodies. Beside an impact on the size of p62/SQSTM1 bodies, inhibition of ULK1/2 kinase activity impacted the abundance of KEAP1 in the bodies which is regulated by hospho-p62/SQSTM1. The inhibitor was used at 2.5 uM for 6 hrs which seems reasonable given the published cellular effects on autophagy observed at 1 uM (Petherick et al 2015

JBC). Yet, it would be advisable to titrate the compound to reasses key results at a arrange of concentrations of MRT68921 (3-5 dilutions). This would: 1) demonstrate specificity of the compound in the cellular p62/SQSTM1 body formation assay, and 2) contribute to better characterization of the compound itseöf, which might be more frequently used by other investigators in the autophagy and oxidative stress response fields following this publication. This would be a gold standard for using pharmacological agents in research.

• MRT68921 is not strictly specific to ULK1/2 and significantly inhibits a dozen or so kinases at 1 uM. Amongst them there is NUAK1 which is strongly inhibited by this small molecule (Petherick et al 2015 JBC). Recently, NUAK1 has been studied in the context of NRF2-mediated response to oxidative stress (Port et al. 2018 CancerDisc): "NUAK1 is activated by oxidative stress and ... this activation is required to facilitate nuclear import of ... NRF2: Activation of NUAK1 coordinates PP1β inhibition with AKT activation in order to suppress GSK3β-dependent inhibition of NRF2 nuclear import." Therefore, it is likely that MRT68921's polypharmacology will make interpretation of the results obtained by Ikeda et al in this manuscript problematic. Perhaps, a knockout of NUAK1 in cells could be attempted to test the effect of MRT68921 on the repression of expression of NRF2-dependent genes in a more controlled way. Port et al. (2018 CancerDisc) observed that NUAK1 depletion phenocopies NRF2 inactivation, so that it would be important to see if MRT68921 can further enhance the NRF2-inhibitory effect caused by the NUAK1 depletion.

• The authors use levels of NRF2-dependent gene expression as evidence for NRF2 stabilization and activation. Would it be possible to demonstrate increased NRF2 levels in nuclear extracts to have direct evidence for NRF2 stabilization?

3. Confirmation of the phenomena described in the cellular assays on the tissue level in vivo

• This study is outstanding because it starts with a biochemical finding that translates into a stunning mouse phenotype, illustrating the significance of the ULK1:p62/SQSTM1:KEAP1:NRF2 signaling axis in maintaining homeostasis. The analyses of the status of different tissues and organs following NRF2 gain of function presented in the manuscript is adequate to explain why the mice with the gain-of-function mutation for p62/SQSTM1 develop a disease phenotype. However, the original premises of the study, i.e. (transient) binding between ULK1 and p62/SQSTM1. Increase sequestration of KEAP1 by phosphor-p62/SQSTM1 have not been confirmed. The livers of the mice, including organ enlargement, may provide a good model to assess the effect of the phosphomimetic mutation on p62/SQSTM1 body formation and/or co-localization of p62/SQSTM1 with ULK1/2 and KEAP1. An attempt to see increased p62/SQSTM1:KEAP1 co-localization in the livers of the sick mice would strongly corroborate the NRF2 gain-of-function gene expression data and loop back to the biochemical data presented at the beginning of the study.

Response to the comments of Reviewer #1

General comment

NRF2 is a transcription fact, regulating a cellular defense system. Under normal conditions, the primary E3 ubiquitin ligase complex (KEAP1-Culline 3-Rbx) constantly targets NRF2 for ubiquitylation and degradation by the26 S proteasome. Under oxidative stress, cysteine residues in KEAP1 sense the redox imbalance, leading to diminished ubiquitylation and thus the stabilization of NRF2. Following the discovery of this redox-dependent NRF2 activation, another non-canonical mechanism of NRF2 activation was discovery, which involves p62. Therefore, although the concept of p62 phosphorylation and recruitment of KEAP1 in p62 bodies is not novel, but the authors delivered several important points. (1) the authors have demonstrated that ULK1 is the kinase for Serine 349 of p62 both in vitro and in vivo. (2) direct interaction of p62 with ULK1 is demonstrated. (3) The knock-in mouse with a phosphomimetic mutation at Ser349, but not those with a phosphodefective mutation, exhibits the persistent activation of NRF2, which phenocopies the systemic KEAP1 knockout mice, i.e. these mice suffered hyperkeratosis and consequently obstruction of the esophagus and forestomach, as well as growth retardation due to malnutrition (death at day 3 in KEAP1 KO mice). This is the first in vivo study demonstrating the importance of p62 phosphorylation at ser 349 in controlling the NRF2 pathway in whole animal. It is not a trivial task. Furthermore, the experiments are well-designed, and the data are in high quality, I suggest acceptance of the manuscript after a minor revision.

Reply

We thank Reviewer #1 for their positive evaluation of our manuscript.

Comment-1

It would be interesting to perform data analyses dissecting mutation status in p62 (especially serine 349), ULK1 and KEAP1, and correlation the mutation status of these proteins with the NRF2 expression level, sensitivity of the cancer cells to therapeutic drugs, as well as patient prognosis.

Reply-1

Using liver hepatocellular carcinoma TCGA PanCancer data (http://www.cbioportal.org/study/summary?id=lihc_tcga_pan_can_atlas_2018), we examined gene expression of NRF2 targets, and copy number variations and mutations in the *p62/SQSTM1*, *ULK1*, and *KEAP1* genes in patients with hepatocellular carcinoma (HCC). Data analysis was performed on 369 HCC cases for which RNA-seq, copy number, and mutation

data were all available. We found the Cys331Ser mutation of *p62/SQSTM1* in only one case, in addition to 17 cases with a *KEAP1* mutation, 12 with a *NFE2L2/NRF2* mutation, and one with *KEAP1*/*NFE2L2* double mutations. During the analysis, we noticed significant co-occurrence of copy number gain in the *ULK1* and *SQSTM1/p62* genes (Figure for Reviewers A). Further, on the basis of comment 3 of Reviewer #3, we also analyzed the expression levels of p62 and KEAP1, the phosphorylation state of p62, and the localization of KEAP1 to p62 bodies in HCC, a tumor characterized by p62 accumulation (Ichimura *et al*, 2013; Inami *et al*, 2011). We confirmed that p62 accumulated in the tumor region of HCC, in particular NASH-derived HCC, but not in the non-tumor region (Figure for Reviewers B). Notably, the amount of Ser349-phosphorylated p62 was also increased in the corresponding tumor region (Figure for Reviewers B). Consistent with a previous report (Saito *et al*, 2016), immunofluorescence analysis with clinical specimens from patients with NASH-derived HCC revealed that p62-positive Mallory-Denk bodies contained Ser349-phosphorylated p62 and KEAP1 (Figure for Reviewers C); these structures are a prominent feature of steatohepatitis and HCC. These data suggest the activation of ULK1 and/or overexpression of ULK1, resulting in the phosphorylation of p62 at Ser349. However, since our ULK1 and ULK2 antibodies were unavailable for either immunoblot analysis or immunofluorescent analysis of clinical specimens, we could not prove the hypothesis. Because the analysis of tumorigenesis and tumor development was not the primary focus of this study, we would like to use the data in a future study.

Comment-2

Fig. 2b: For ULK1 association with p62 in the droplet form. It would be better to include a negative control, such as an irrelevant kinase.

Reply-2

According to the suggestion, we used SNAP and protein kinase A as negative controls and confirmed that their localization in p62 condensates was low. We provided the data in Fig. EV2B of the revised manuscript.

Comment-3

Fig. 2e: similar to Fig. 2b, an irrelevant kinase as a negative control will make the colocalization of ULK1 with p62 bodies more meaningful/convincing.

Reply-3

As a representative negative example (meaning no effect on size or number of p62 bodies, or on p62 phosphorylation state), we investigated the localization of serine-threonine-protein kinase LATS1 in Huh-1 cells and found that GFP-tagged LATS1 was diffusely localized in the cytoplasm. We utilized LATS1 as a negative control (Fig. EV2A in the revised manuscript).

Comment-4

Fig. 4C Have the authors performed the same experiment using p62 WT and p62-S349E, rather than adding S349E to S403E S407E? is the p62-S349E alone sufficient to change the biophysical properties of p62 bodies?

Reply-4

In accordance with this suggestion, we carried out an *in vitro* LLPS assay with wild-type p62 and $p62^{S349E}$ and confirmed that the addition of KEAP1 to $p62^{S349E}$, but not to wild-type p62, significantly changed the biophysical properties of p62 condensates as was observed for $p62^{S349E\ S403E\ S407E}$, indicating that the single S349E mutation is sufficient to change the biophysical properties of p62 condensates. We provided the data in Figure 4D of the revised manuscript.

<u>Response to the comments of Reviewer #2</u>

General comment

In this manuscript Ikeda et al. explores redox-independent NRF2 activation through the formation of p62 bodies which contain S349 phosphorylated p62. The authors show ULK1 to be an important kinase responsible for this phosphorylation and shows that the phosphorylation at S349 is important to retain KEAP1 in the p62 body which in turn allows NRF2 activation. Heterozygous phosphomimetic p62S351E/+ knock-in mice phenocopy KEAP1 knockouts while the phosphor-deficient p62S351A has no obvious effects. It is an engaging study where the authors have discovered and described a regulatory role of ULK1 in p62 bodies on a molecular level and also have convincing in vivo data from p62 phosphomutant mice, indicating physiological relevance of the finding. With some experiments and changes I find the manuscript very suitable for publication in EMBO J.

Reply

We thank Reviewer #2 for their positive evaluation and valuable comments on our manuscript.

Major comments

Comment-1

• *In their experiment in fig 1f-where the authors show the in vitro phosphorylation of p62 by ULK1 and Atg1 it will be helpful to show on the blots with arrows if there are dimers formed. There are several unexplained bands upon blotting for p62 (pS349). The authors may add a few lines to specify clearly if formation of dimers are affected by phosphorylation at S349.*

Reply-1

Thank you for the suggestion. Our HS-AFM of p62 (268–440 aa) visualized the homo-dimeric structure mediated by the dimerization of the UBA domain (Fig. EV1B). Thus, as pointed out by the reviewer, the slower migrating bands may represent homo-oligomers of p62 (268–440 aa) and p62 (320-440 aa) recombinants. We described this possibility in the legend of Figure 1F in the revised manuscript (Page 27, line 765-766 in the revised manuscript).

Comment-2

• *How does the S349 phosphorylation affect LC3 binding, Ub binding, p62 and KEAP1 levels in cells? The mice data in fig 5f suggests it may affect KEAP1 levels.*

Reply-2

We appreciate this helpful comment. KEAP1 is incorporated into p62 bodies (Kageyama *et al*, 2021), and the complex of KEAP1 and p62 is degraded by autophagy (Taguchi *et al*, 2012). Thus, phosphorylation of p62 at Ser349, which increases the binding affinity of p62 to KEAP1, should promote the degradation of KEAP1. We described the aforementioned possibility in the results and discussion sections of the revised manuscript (Page 10, line 285-290 and Page 12, line 356-359 in the revised manuscript).

Comment-3

• *Previous papers have identified several ULK1 mediated phosphorylation sites in the UBA domain of p62. How do these phosphorylations affect the phosphorylation by ULK1 at S349 and vice versa? Repeating the in vitro phosphorylation assay in Fig1f with phospho-defective mutants may help to understand if and how they are influenced by each other.*

Reply-3

Thank you for this valuable comment. As pointed out, ULK1 also phosphorylates Ser403 and Ser407 within the UBA of p62 (Lim *et al*, 2015; Ro *et al*, 2014). It is unclear whether the

phosphorylation of UBA affects Ser349 and vice versa. To address this issue, we simply expressed wild-type p62, $p62^{S349E}$, $p62^{S349A}$, $p62^{S403E\ S407E}$, or $p62^{S403A\ S407A}$ mutant in *p62*-deficient Huh-1 cells and investigated the phosphorylation states of Ser349, Ser403, and Ser407. As shown in Appendix Figure S2, while the $p62^{S349E}$ mutant had no effect on the phosphorylation of the UBA domain, the $p62^{S403E\ S407E}$ mutant enhanced the phosphorylation of Ser349. Considering that the phosphorylation of S403 and S407 is thought to promote the LLPS of p62, it is plausible that Ser349 phosphorylation of p62 occurs within p62 bodies, which is supported by our study.

Comment-4

• *Does the S349E mutation have any effect on p62 condensate formation in the assay shown in Figure 2A, ULK1 localisation to condensates (as in fig 2B)?*

Reply-4

We added the data of $p62^{S349E}$ condensates (Figure 2A in the revised manuscript). We also examined Atg1 and ULK1 localization in the $p62^{S349E}$ condensates, as shown in Fig. EV2A of the revised manuscript. While the S349E mutation slightly but significantly increased the size of the p62 condensates (Figure 2A of the revised manuscript), it did not affect Atg1 or ULK1 localization (Fig. EV2A of the revised manuscript).

Comment-5

• *Colocalization of KEAP1 to condensates should be added to the analysis of fig4A in addition to the circularity of condensates shown in fig 4B. This may support the role of S349 phosphorylation in retaining KEAP1 in p62 bodies.*

Reply-5

In accordance with the comment, we measured the signal intensity of mCherry-KEAP1 on p62 bodies consisting of wild-type p62 or a series of mutants. We found that the intensity of mCherry-KEAP1 on $p62^{S349E}$ bodies was higher than that on p62 bodies comprised of wild-type p62 or $p62^{S349A}$ (Figure 4C in the revised manuscript). There was no significant difference in mCherry-KEAP1 intensity between wild-type p62 and $p62^{S349A}$ bodies (Figure 4C in the revised manuscript). These results imply that Ser349 phosphorylation of p62 promotes retention of KEAP1 in p62 bodies.

Comment-6

• *The data in figure 4C shows clearly how the morphology of the condensates change in the*

triple mutant p62(S349ES403ES407E). However, the morphology of S349E by itself is missing.

Reply-6

This was also pointed out by Reviewer #1 (comment 4). In accordance with this suggestion, we carried out an *in vitro* LLPS assay with wild-type p62 and $p62^{S349E}$ and confirmed that the addition of KEAP1 to $p62^{S349E}$, but not to wild-type p62, significantly changed the biophysical properties of p62 condensates as was observed for $p62^{S349E\ S403E\ S407E}$, indicating that the single S349E mutation is sufficient to alter these properties. We provided the data in Figure 4D of the revised manuscript.

Minor comments

Comment-1

• *Typing error at line 181 -KEPA instead of KEAP*

Reply-1

We corrected the typo.

Comment-2

• *Atg1 should be consistently in lowercase in Figures/manuscript to keep nomenclature uniform for mammalian and yeast proteins*

Reply-2

We sincerely apologize for this inappropriate notation. We substituted ATG1 with Atg1 throughout the manuscript and Figures.

Comment-3

• *Figure 4b: axis label is missing*

Reply-3

We labeled the axis with the word "Circularity."

Comment-4

• *Are there any nonspecific bands in the KEAP blot in fig 5F? if so then please use * to clarify the non-specific band.*

Reply-4

Both bands disappeared following gene targeting of mouse *Keap1* (Taguchi *et al.*, 2012), implying that they are specific. Since there is no variant of mouse *Keap1* in the Ensemble database, faster or slower migrating bands may represent post-translational modification of KEAP1. In the legends (Figure 5F and Fig. EV5E), we state that both bands represent post-translational modification products of *Keap1*.

Response to the comments of Reviewer #3

General comment

Synopsis

In their manuscript, Ikeda et al describe an elegant study, in which they look at the consequence and the impact of the direct interaction between the autophagy cargo adaptor protein p62/SQSTM1 and the major autophagy-specific kinase ULK1 that phosphorylates human p62/SQSTM1 on Ser349 (amongst other sites). This post-translational modification of p62/SQSTM1 increases its interaction with the E3 ligase KEAP1 responsible for the tonic degradation of the transcription factor NRF2 involved in oxidative stress response. Stronger binding between p62/SQSTM1 segregates KEAP1 in p62/SQSTM1 bodies leading to KEAP1 degradation and consequently NRF2 stabilization and activation under normal conditions. Permanent increase in KEAP1 binding and its degradation in a p62/SQSTM1-dependent fashion has a profound effect on mouse development, underscoring the pivotal role the ULK1-p62-KEAP1-NRF2 pathway plays in the maintenance of the organismal homeostasis. The study uses advanced technology, and its clear strength lies in demonstrating the consequence of the gain-of-function mutation in p62/SQSTM1 in the mice in vivo. Below, I am listing several points whose resolution could help further strengthen the quality of this interesting paper.

Reply

We thank Reviewer #3 for their valuable comments and insightful suggestions.

Specific comments

Comment-1

1. Interaction of Atg1/ULK1 with p62/SQSTM1

• Using purified proteins and high-speed atomic force microscopy, the authors demonstrated a direct, dynamic interaction between truncated p62/SQSTM1 and ULK1 (also the yeast homologue Atg1). The interaction is proposed to be driven by the intrinsically disordered regions (IDRs) of the respective proteins (Atg1/ULK1 and p62/SQSTM1) and possibly other regions. The spectacular video sequences suggest the transient nature of the described interactions. Is that at all possible to determine the Kd values for those binding pairs using a

more classical biochemical method? Since p62/SQSTM1 also binds FIP200 directly via its FIP200-interacting region, it would seem to be important to know the relative contribution of ULK1:p62/SQSTM1 vs. ULK1:FIP200:p62/SQSTM1 complexes. Knowing affinities of the interactions, hypothesis generation would be made possible.

• In addition, the paper would benefit from a multiple alignment of Atg1/ULK1/ULK2 proteins to discuss which conserved sequences/motifs may drive the direct binding between p62/SQSTM1 and the kinases of the Atg1 family.

Reply-1

• In accordance with the former comment, we conducted an *in vitro* binding assay using purified p62, ULK1, and FIP200 Claw based on the methods used to characterize the FIP200 Claw–p62 interaction (Turco *et al*, 2019). As shown in the revised Fig. EV3B and C, ULK1 alone bound to p62 and that FIP200 did not interfere with ULK1 for p62-binding.

• In accordance with the latter comment, we performed an alignment of the intrinsically disordered regions (IDRs) of ULK1, ULK2, and Atg1, but did not find any highly conserved regions. As shown by HS-AFM analysis (Fig. 1D), IDR-mediated interaction between ULK1 and p62 is transient and rather weak. In such cases it is generally difficult to find conserved binding sequences/motifs due to the extremely low sequence identity. We described the aforementioned possibility in the discussion section of the revised manuscript (Page 11-12, line 346-350 in the revised manuscript).

Comment-2

2. Use of a small molecule inhibitor to probe for the functional significance of ULK1 in p62/SQSTM1 bodies

• The authors made use of the published ULK1/2 inhibitor MRT68921 to investigate the role of the ULK1/2 catalytical activity in the formation of p62/SQSTM1 bodies. Beside an impact on the size of p62/SQSTM1 bodies, inhibition of ULK1/2 kinase activity impacted the abundance of KEAP1 in the bodies which is regulated by hospho-p62/SQSTM1. The inhibitor was used at 2.5 uM for 6 hrs which seems reasonable given the published cellular effects on autophagy observed at 1 uM (Petherick et al 2015 JBC). Yet, it would be advisable to titrate the compound to reasses key results at a arrange of concentrations of MRT68921 (3-5 dilutions). This would: 1) demonstrate specificity of the compound in the cellular p62/SQSTM1 body formation assay, and 2) contribute to better characterization of the compound itseöf, which might be more frequently used by other investigators in the autophagy and oxidative stress response fields following this publication. This would be a gold standard for using pharmacological agents in research.

• MRT68921 is not strictly specific to ULK1/2 and significantly inhibits a dozen or so kinases at 1 uM. Amongst them there is NUAK1 which is strongly inhibited by this small molecule (Petherick et al 2015 JBC). Recently, NUAK1 has been studied in the context of NRF2-mediated response to oxidative stress (Port et al. 2018 CancerDisc): "NUAK1 is activated by oxidative stress and ... this activation is required to facilitate nuclear import of ... NRF2: Activation of NUAK1 coordinates PP1β inhibition with AKT activation in order to suppress GSK3β-dependent inhibition of NRF2 nuclear import." Therefore, it is likely that MRT68921's polypharmacology will make interpretation of the results obtained by Ikeda et al in this manuscript problematic. Perhaps, a knockout of NUAK1 in cells could be attempted to test the effect of MRT68921 on the repression of expression of NRF2-dependent genes in a more controlled way. Port et al. (2018 CancerDisc) observed that NUAK1 depletion phenocopies NRF2 inactivation, so that it would be important to see if MRT68921 can further enhance the NRF2-inhibitory effect caused by the NUAK1 depletion.

• The authors use levels of NRF2-dependent gene expression as evidence for NRF2 stabilization and activation. Would it be possible to demonstrate increased NRF2 levels in nuclear extracts to have direct evidence for NRF2 stabilization?

Reply-2

• In addition to treating Huh-1 cells with 2.5 μM MRT68921, as in the original manuscript, we treated the cells with 1.0 μM MRT68921 for 6 h and tested the effect on NRF2 activity. Despite the lower concentration, the gene expression of NRF2 targets was suppressed by the treatment of MRT68291 (Fig. EV4 of the revised manuscript).

• To circumvent the problem of the polypharmacological effect of MRT68921, we also used ULK-101, a more specific inhibitor of ULK1 and ULK2 (Martin *et al*, 2018). Huh-1 cells were cultured in the presence or absence of 7.5 μM ULK-101 for 6 h; these are considered to be an appropriate concentration and treatment time (Perez *et al*, 2022; Zhang *et al*, 2022a; Zhang *et al*, 2022b). We then evaluated the effect on NRF2 activation. As shown in Fig. EV4 in the revised manuscript, the treatment of Huh-1 cells with ULK-101 suppressed the gene expression of NRF2 targets.

• We previously elucidated in detail that the S349 phosphorylation of p62 promotes nuclear translocation of NRF2 (Ichimura *et al.*, 2013; Kageyama *et al.*, 2021; Saito *et al.*, 2016), and the mechanism has been verified by others. Therefore, in this study we showed the results of NRF2 activation, *i.e.*, gene expression of NRF2 targets, using real-time PCR, which has higher sensitivity than immunoblotting with anti-NRF2 antibody.

Comment-3

3. Confirmation of the phenomena described in the cellular assays on the tissue level in vivo

• This study is outstanding because it starts with a biochemical finding that translates into a stunning mouse phenotype, illustrating the significance of the ULK1:p62/SQSTM1:KEAP1:NRF2 signaling axis in maintaining homeostasis. The analyses of the status of different tissues and organs following NRF2 gain of function presented in the manuscript is adequate to explain why the mice with the gain-of-function mutation for p62/SQSTM1 develop a disease phenotype. However, the original premises of the study, i.e. (transient) binding between ULK1 and p62/SQSTM1. Increase sequestration of KEAP1 by phosphor-p62/SQSTM1 have not been confirmed. The livers of the mice, including organ enlargement, may provide a good model to assess the effect of the phosphomimetic mutation on p62/SQSTM1 body formation and/or co-localization of p62/SQSTM1 with ULK1/2 and KEAP1. An attempt to see increased p62/SQSTM1:KEAP1 co-localization in the livers of the sick mice would strongly corroborate the NRF2 gain-of-function gene expression data and loop back to the biochemical data presented at the beginning of the study.

Reply-3

• Instead of analyses with the mouse disease model, we analyzed the expression levels of p62 and KEAP1, the phosphorylation state of p62, and the localization of KEAP1 to p62 bodies in human hepatocellular carcinoma (HCC), a tumor characterized by p62 accumulation (Ichimura *et al.*, 2013; Inami *et al.*, 2011). We confirmed that p62 accumulated in the tumor region of HCC, in particular NASH-derived HCC, but not in the non-tumor region (Figure for Reviewers B). Notably, the amount of Ser349-phosphorylated p62 was also increased in the corresponding tumor region (Figure for Reviewers B). Consistent with a previous report (Saito *et al.*, 2016), immunofluorescence analysis with clinical specimens from patients with NASH-derived HCC revealed that p62-positive Mallory-Denk bodies contained Ser349-phosphorylated p62 and KEAP1 (Figure for Reviewers C); these structures are a prominent feature of steatohepatitis and HCC. These data suggest the presence of ULK1 activation and/or ULK1 overexpression, resulting in the phosphorylation of p62 at Ser349. However, since our ULK1 and ULK2 antibodies were unavailable for either immunoblot analysis or immunofluorescent analysis of clinical specimens, we could not prove the hypothesis. Because the analysis of tumorigenesis and tumor development was not the primary focus of this study, we would like to use the data in a future study.

References

Ichimura Y, Waguri S, Sou YS, Kageyama S, Hasegawa J, Ishimura R, Saito T, Yang Y, Kouno T, Fukutomi T et al (2013) Phosphorylation of p62 activates the Keap1-Nrf2 pathway during selective autophagy. Mol Cell 51: 618-631

Inami Y, Waguri S, Sakamoto A, Kouno T, Nakada K, Hino O, Watanabe S, Ando J, Iwadate M, Yamamoto M et al (2011) Persistent activation of Nrf2 through p62 in hepatocellular carcinoma cells. J Cell Biol 193: 275-284

Kageyama S, Gudmundsson SR, Sou YS, Ichimura Y, Tamura N, Kazuno S, Ueno T, Miura Y, Noshiro D, Abe M et al (2021) p62/SQSTM1-droplet serves as a platform for autophagosome formation and anti-oxidative stress response. Nat Commun 12: 16

Lim J, Lachenmayer ML, Wu S, Liu W, Kundu M, Wang R, Komatsu M, Oh YJ, Zhao Y, Yue Z (2015) Proteotoxic stress induces phosphorylation of p62/SQSTM1 by ULK1 to regulate selective autophagic clearance of protein aggregates. PLoS Genet 11: e1004987

Martin KR, Celano SL, Solitro AR, Gunaydin H, Scott M, O'Hagan RC, Shumway SD, Fuller P, MacKeigan JP (2018) A Potent and Selective ULK1 Inhibitor Suppresses Autophagy and Sensitizes Cancer Cells to Nutrient Stress. iScience 8: 74-84

Perez RE, Saleiro D, Ilut L, Schiltz GE, Eckerdt F, Fish EN, Platanias LC (2022) Regulation of IFNalpha-induced expression of the short ACE2 isoform by ULK1. Mol Immunol 147: 1-9

Ro SH, Semple IA, Park H, Park H, Park HW, Kim M, Kim JS, Lee JH (2014) Sestrin2 promotes Unc-51-like kinase 1 mediated phosphorylation of p62/sequestosome-1. FEBS J 281: 3816-3827

Saito T, Ichimura Y, Taguchi K, Suzuki T, Mizushima T, Takagi K, Hirose Y, Nagahashi M, Iso T, Fukutomi T et al (2016) p62/Sqstm1 promotes malignancy of HCV-positive hepatocellular carcinoma through Nrf2-dependent metabolic reprogramming. Nat Commun 7: 12030

Taguchi K, Fujikawa N, Komatsu M, Ishii T, Unno M, Akaike T, Motohashi H, Yamamoto M (2012) Keap1 degradation by autophagy for the maintenance of redox homeostasis. Proc Natl Acad Sci U S A 109: 13561-13566

Turco E, Witt M, Abert C, Bock-Bierbaum T, Su MY, Trapannone R, Sztacho M, Danieli A, Shi X, Zaffagnini G et al (2019) FIP200 Claw Domain Binding to p62 Promotes Autophagosome Formation at Ubiquitin Condensates. Mol Cell 74: 330-346 e311

Zhang Y, Song XL, Yu B, Foong LC, Shu Y, Mai CW, Hu J, Dong B, Xue W, Chua CW (2022a) TP53 loss-of-function causes vulnerability to autophagy inhibition in aggressive prostate cancer. Int J Urol 29: 1085-1094

Zhang Y, Zhang C, Li J, Jiang M, Guo S, Yang G, Zhang L, Wang F, Yi S, Wang J et al (2022b) Inhibition of AKT induces p53/SIRT6/PARP1-dependent parthanatos to suppress tumor growth. Cell Commun Signal 20: 93

Dear Masaaki,

Thank you for submitting the revised version of your manuscript, which takes into account the concerns of all referees. As you will see below, one referee replied with a re-review report and was entirely satisfied with your revised manuscript.

Before I can formally accept your manuscript for publication, however, there are some remaining editorial points which need to be addressed. In this regard would you please:

Re-colour the red text in the manuscript black,
• rename the Conflict of Interest Section as the "DISCLOSURE AND COMPETING INTERESTS STATEMENT" according to the instructions on our website,
• remove the AC/CRediT section from the manuscript text,
• rearrange the callouts so the callout for Fig. EV1C comes after EV1B; there is a callout missing for Fig. EV1D; Appendix Fig. S1C and D is called out, but the figure has no labeled panels,
• supply raw data for EV figure 3B,
• rename movie EV1-EV10 with the corresponding callouts, with the legends removed from the Appendix; instead and zip each legend with its movie file, and
• reorder the sections according to the instructions to authors on our website.

We include a synopsis of the paper (see http://emboj.embopress.org/). Please provide me with a general summary statement and 3-5 bullet points that capture the key findings of the paper.
We also need a summary figure for the synopsis. The size should be 550 wide by [200-400] high (pixels). You can also use something from the figures if that is easier.

EMBO Press is an editorially independent publishing platform for the development of EMBO scientific publications.

Best wishes,

William

William Teale, PhD
Editor
The EMBO Journal
w.teale@embojournal.org

Instructions for preparing your revised manuscript:

Please check that the title and abstract of the manuscript are brief, yet explicit, even to non-specialists.

When assembling figures, please refer to our figure preparation guideline in order to ensure proper formatting and readability in print as well as on screen:
https://bit.ly/EMBOPressFigurePreparationGuideline
See also figure legend guidelines: https://www.embopress.org/page/journal/14602075/authorguide#figureformat

IMPORTANT: When you send the revision we will require
- a point-by-point response to the referees' comments, with a detailed description of the changes made (as a word file).
- a word file of the manuscript text.
- individual production quality figure files (one file per figure)
- a complete author checklist, which you can download from our author guidelines (https://www.embopress.org/page/journal/14602075/authorguide).
- Expanded View files (replacing Supplementary Information)
Please see out instructions to authors
https://www.embopress.org/page/journal/14602075/authorguide#expandedview

Please remember: Digital image enhancement is acceptable practice, as long as it accurately represents the original data and conforms to community standards. If a figure has been subjected to significant electronic manipulation, this must be noted in the figure legend or in the 'Materials and Methods' section. The editors reserve the right to request original versions of figures and the original images that were used to assemble the figure.

Further information is available in our Guide For Authors: https://www.embopress.org/page/journal/14602075/authorguide

We realize that it is difficult to revise to a specific deadline. In the interest of protecting the conceptual advance provided by the work, we recommend a revision within 3 months (27th Jul 2023). Please discuss the revision progress ahead of this time with the editor if you require more time to complete the revisions. Use the link below to submit your revision:

https://emboj.msubmit.net/cgi-bin/main.plex

Referee #3:

This is a revision, and I am satisfied with the additional experimental data and explanations provided by the authors. I can recommend this manuscript for acceptance by the EMBO J.

Response to the comments of Reviewer #3

Referee #3:

This is a revision, and I am satisfied with the additional experimental data and explanations provided by the authors. I can recommend this manuscript for acceptance by the EMBO J.

Reply

We thank Reviewer #3 for their positive evaluation of our manuscript.

Dear Masaaki,

I am pleased to inform you that your manuscript has been accepted for publication in the EMBO Journal.

Congratulations on the publication of a really nice study.

Please note that it is EMBO Journal policy for the transcript of the editorial process (containing referee reports and your response letter) to be published as an online supplement to each paper. If you do NOT want this, you will need to inform the Editorial Office via email immediately. More information is available here:
https://www.embopress.org/page/journal/14602075/authorguide#transparentprocess

Your manuscript will be processed for publication in the journal by EMBO Press. Manuscripts in the PDF and electronic editions of The EMBO Journal will be copy edited, and you will be provided with page proofs prior to publication. Please note that supplementary information is not included in the proofs.

You will be contacted by Wiley Author Services to complete licensing and payment information. The required 'Page Charges Authorization Form' is available here: https://www.embopress.org/pb-assets/embo-site/tej_apc.pdf - please download and complete the form and return to embopressproduction@wiley.com

EMBO Press participates in many Publish and Read agreements that allow authors to publish Open Access with reduced/no publication charges. Check your eligibility: https://authorservices.wiley.com/author-resources/Journal-Authors/open-access/affiliation-policies-payments/index.html

Should you be planning a Press Release on your article, please get in contact with embojournal@wiley.com as early as possible, in order to coordinate publication and release dates.

If you have any questions, please do not hesitate to call or email the Editorial Office. Thank you for your contribution to The EMBO Journal.

Best wishes,

William

William Teale, PhD
Editor
The EMBO Journal
w.teale@embojournal.org

** Click here to be directed to your login page: https://emboj.msubmit.net

EMBO Press Author Checklist

Corresponding Author Name: Masaaki Komatsu, Nobuo N Noda, Yoshinobu Ichimmura	
Journal Submitted to: The EMBO Journal	
Manuscript Number: EMBOJ-2022-113349	

USEFUL LINKS FOR COMPLETING THIS FORM
The EMBO Journal - Author Guidelines
EMBO Reports - Author Guidelines
Molecular Systems Biology - Author Guidelines
EMBO Molecular Medicine - Author Guidelines

Reporting Checklist for Life Science Articles (updated January
This checklist is adapted from Materials Design Analysis Reporting (MDAR) Checklist for Authors. MDAR establishes a minimum set of requirements in transparent reporting in the life sciences (see Statement of Task: 10.31222/osf.io/9sm4x). Please follow the journal's guidelines in preparing your
Please note that a copy of this checklist will be published alongside your article.

Abridged guidelines for figures
1. Data
The data shown in figures should satisfy the following conditions:
- the data were obtained and processed according to the field's best practice and are presented to reflect the results of the experiments in an accurate and unbiased manner.
- ideally, figure panels should include only measurements that are directly comparable to each other and obtained with the same assay.
- plots include clearly labeled error bars for independent experiments and sample sizes. Unless justified, error bars should not be shown for technical
- if n<5, the individual data points from each experiment should be plotted. Any statistical test employed should be justified.
- Source Data should be included to report the data underlying figures according to the guidelines set out in the authorship guidelines on Data

2. Captions
Each figure caption should contain the following information, for each panel where they are relevant:
- a specification of the experimental system investigated (eg cell line, species name).
- the assay(s) and method(s) used to carry out the reported observations and measurements.
- an explicit mention of the biological and chemical entity(ies) that are being measured.
- an explicit mention of the biological and chemical entity(ies) that are altered/varied/perturbed in a controlled manner.
- the exact sample size (n) for each experimental group/condition, given as a number, not a range;
- a description of the sample collection allowing the reader to understand whether the samples represent technical or biological replicates (including how many animals, litters, cultures, etc.).
- a statement of how many times the experiment shown was independently replicated in the laboratory.
- definitions of statistical methods and measures:
  - common tests, such as t-test (please specify whether paired vs. unpaired), simple χ2 tests, Wilcoxon and Mann-Whitney tests, can be unambiguously identified by name only, but more complex techniques should be described in the methods section;
  - are tests one-sided or two-sided?
  - are there adjustments for multiple comparisons?
  - exact statistical test results, e.g., P values = x but not P values < x;
  - definition of 'center values' as median or average;
  - definition of error bars as s.d. or s.e.m.

Please complete ALL of the questions below.
Select "Not Applicable" only when the requested information is not relevant for your study.

Materials

Newly Created Materials	Information included in the manuscript?	In which section is the information available? (Reagents and Tools Table, Materials and Methods, Figures, Data Availability Section)
New materials and reagents need to be available; do any restrictions apply?	Yes	Resources table

Antibodies	Information included in the manuscript?	In which section is the information available? (Reagents and Tools Table, Materials and Methods, Figures, Data Availability Section)
For **antibodies** provide the following information:<br>- Commercial antibodies: RRID (if possible) or supplier name, catalogue number and or/clone number<br>- Non-commercial: RRID or citation	Yes	Material and Methods, and Resources table

DNA and RNA sequences	Information included in the manuscript?	In which section is the information available? (Reagents and Tools Table, Materials and Methods, Figures, Data Availability Section)
**Short novel DNA or RNA including primers, probes**: provide the sequences.	Yes	Materials and Methods

Cell materials	Information included in the manuscript?	In which section is the information available? (Reagents and Tools Table, Materials and Methods, Figures, Data Availability Section)
**Cell lines:** Provide species information, strain. Provide accession number in repository **OR** supplier name, catalog number, clone number, and/**OR** RRID.	Yes	Resources table
**Primary cultures:** Provide species, strain, sex of origin, genetic modification status.	Not Applicable	
Report if the cell lines were recently **authenticated** (e.g., by STR profiling) and tested for mycoplasma contamination.	Yes	Materials and Methods

Experimental animals	Information included in the manuscript?	In which section is the information available? (Reagents and Tools Table, Materials and Methods, Figures, Data Availability Section)
**Laboratory animals or Model organisms:** Provide species, strain, sex, age, genetic modification status. Provide accession number in repository **OR** supplier name, catalog number, clone number, **OR** RRID.	Yes	Materials and Methods, and Figure legends
**Animal observed in or captured from the field:** Provide species, sex, and age where possible.	Not Applicable	
Please detail **housing and husbandry conditions**.	Yes	Materials and Methods

Plants and microbes	Information included in the manuscript?	In which section is the information available? (Reagents and Tools Table, Materials and Methods, Figures, Data Availability Section)
**Plants:** provide species and strain, ecotype and cultivar where relevant, unique accession number if available, and source (including location for collected wild specimens).	Not Applicable	
**Microbes:** provide species and strain, unique accession number if available, and source.	Not Applicable	

Human research participants	Information included in the manuscript?	In which section is the information available? (Reagents and Tools Table, Materials and Methods, Figures, Data Availability Section)
If collected and within the bounds of privacy constraints report on age, sex and gender or ethnicity for all study participants.	Not Applicable	

Core facilities	Information included in the manuscript?	In which section is the information available? (Reagents and Tools Table, Materials and Methods, Figures, Data Availability Section)
If your work benefited from core facilities, was their service mentioned in the acknowledgments section?	Not Applicable	

Design

Study protocol	Information included in the manuscript?	In which section is the information available? (Reagents and Tools Table, Materials and Methods, Figures, Data Availability Section)
If study protocol has been **pre-registered, provide DOI in the manuscript**. For clinical trials, provide the trial registration number **OR** cite DOI.	Not Applicable	
Report the **clinical trial registration number** (at ClinicalTrials.gov or equivalent), where applicable.	Not Applicable	

Laboratory protocol	Information included in the manuscript?	In which section is the information available? (Reagents and Tools Table, Materials and Methods, Figures, Data Availability Section)
Provide DOI OR other citation details if **external detailed step-by-step protocols** are available.	Not Applicable	

Experimental study design and statistics	Information included in the manuscript?	In which section is the information available? (Reagents and Tools Table, Materials and Methods, Figures, Data Availability Section)
Include a statement about **sample size** estimate even if no statistical methods were used.	Yes	Figure legends
Were any steps taken to minimize the effects of subjective bias when allocating animals/samples to treatment (e.g. **randomization procedure**)? If yes, have they been described?	Not Applicable	
Include a statement about **blinding** even if no blinding was done.	Not Applicable	
Describe **inclusion/exclusion criteria** if samples or animals were excluded from the analysis. Were the criteria pre-established?<br><br>If sample or data points were omitted from analysis, report if this was due to attrition or intentional exclusion and provide justification.	Not Applicable	
For every figure, are **statistical tests** justified as appropriate? Do the data meet the assumptions of the tests (e.g., normal distribution)? Describe any methods used to assess it. Is there an estimate of variation within each group of data? Is the variance similar between the groups that are being statistically compared?	Yes	Materials and Methods, and Figure legends

Sample definition and in-laboratory replication	Information included in the manuscript?	In which section is the information available? (Reagents and Tools Table, Materials and Methods, Figures, Data Availability Section)
In the figure legends: state number of times the experiment was **replicated** in laboratory.	Yes	Figure legends
In the figure legends: define whether data describe **technical or biological replicates**.	Yes	Figure legends

Ethics

Ethics	Information included in the manuscript?	In which section is the information available? (Reagents and Tools Table, Materials and Methods, Figures, Data Availability Section)
Studies involving **human participants**: State details of **authority granting ethics approval** (IRB or equivalent committee(s), provide reference number for approval.	Not Applicable	
Studies involving **human participants**: Include a statement confirming that **informed consent** was obtained from all subjects and that the experiments conformed to the principles set out in the WMA Declaration of Helsinki and the Department of Health and Human Services Belmont Report.	Not Applicable	
Studies involving **human participants:** For publication of **patient photos**, include a statement confirming that consent to publish was obtained.	Not Applicable	
Studies involving experimental **animals**: State details of **authority granting ethics approval** (IRB or equivalent committee(s), provide reference number for approval. Include a statement of compliance with ethical regulations.	Yes	Materials and Methods
Studies involving **specimen and field samples:** State if relevant **permits** obtained, provide details of authority approving study; if none were required, explain why.	Not Applicable	

Dual Use Research of Concern (DURC)	Information included in the manuscript?	In which section is the information available? (Reagents and Tools Table, Materials and Methods, Figures, Data Availability Section)
Could your study fall under dual use research restrictions? Please check biosecurity documents and list of **select agents and toxins** (CDC): https://www.selectagents.gov/sat/list.htm	Not Applicable	
If you used a select agent, is the security level of the lab appropriate and reported in the manuscript?	Not Applicable	
If a study is subject to dual use research of concern regulations, is the name of the **authority granting approval and reference number** for the regulatory approval provided in the manuscript?	Not Applicable	

Reporting

The MDAR framework recommends adoption of discipline-specific guidelines, established and endorsed through community initiatives. Journals have their own policy about requiring specific guidelines and recommendations to complement MDAR.

Adherence to community standards	Information included in the manuscript?	In which section is the information available? (Reagents and Tools Table, Materials and Methods, Figures, Data Availability Section)
State if relevant guidelines or checklists (e.g., **ICMJE, MIBBI, ARRIVE, PRISMA**) have been followed or provided.	Not Applicable	
For **tumor marker prognostic studies,** we recommend that you follow the **REMARK** reporting guidelines (see link list at top right). See author guidelines, under 'Reporting Guidelines'. Please confirm you have followed these guidelines.	Not Applicable	
For **phase II and III randomized controlled trials**, please refer to the **CONSORT** flow diagram (see link list at top right) and submit the CONSORT checklist (see link list at top right) with your submission. See author guidelines, under 'Reporting Guidelines'. Please confirm you have submitted this list.	Not Applicable	

Data Availability

Data availability	Information included in the manuscript?	In which section is the information available? (Reagents and Tools Table, Materials and Methods, Figures, Data Availability Section)
Have **primary datasets** been deposited according to the journal's guidelines (see 'Data Deposition' section) and the respective accession numbers provided in the Data Availability Section?	Yes	Data Availability
Were **human clinical and genomic datasets** deposited in a public access-controlled repository in accordance to ethical obligations to the patients and to the applicable consent agreement?	Not Applicable	
Are **computational models** that are central and integral to a study available without restrictions in a machine-readable form? Were the relevant accession numbers or links provided?	Not Applicable	
If publicly available data were reused, provide the respective **data citations in the reference list**.	Not Applicable	
