# Supplementary material for: Phosphorylation of phase‐separated p62 bodies by ULK1 activates a redox‐independent stress response
Source: EMBO J. 2023 Jun 12;42(14):e113349. doi: 10.15252/embj.2022113349 (PMC10350833; doi:10.15252/embj.2022113349)
Supplement: Supplementary file 14 — Source Data for Expanded View and Appendix [file EMBJ-42-e113349-s005.zip › EMBOJ-2022-113349_SourceDataForExpandedView/Figure EV3-20230502.zip!/Figure EV3/EV3A/README_Fig. EV3.docx]

Source files for Figure EV3

For high magnification pictures, cropped regions were used for generating the figure. Brightness/contrast of each figure was adjusted using the Photoshop software.
