# Supplementary material for: Phosphorylation of phase‐separated p62 bodies by ULK1 activates a redox‐independent stress response
Source: EMBO J. 2023 Jun 12;42(14):e113349. doi: 10.15252/embj.2022113349 (PMC10350833; doi:10.15252/embj.2022113349)
Supplement: Supplementary file 14 — Source Data for Expanded View and Appendix [file EMBJ-42-e113349-s005.zip › EMBOJ-2022-113349_SourceDataForExpandedView/Figure EV3-20230502.zip!/Figure EV3/EV3B/README_Fig EV3B.docx]

Source files for Figure EV3B

FV31S-SW software was used for generating the figure. Image size, 212.132 μm × 212.132 μm (512 × 512 pixel).
