# Supplementary material for: Phosphorylation of phase‐separated p62 bodies by ULK1 activates a redox‐independent stress response
Source: EMBO J. 2023 Jun 12;42(14):e113349. doi: 10.15252/embj.2022113349 (PMC10350833; doi:10.15252/embj.2022113349)
Supplement: Supplementary file 14 — Source Data for Expanded View and Appendix [file EMBJ-42-e113349-s005.zip › EMBOJ-2022-113349_SourceDataForExpandedView/Figure EV2-20230417T091624Z-001.zip!/Figure EV2/README_Fig EV2.docx]

Source files for Figure EV2

FV31S-SW software was used for generating the figure. Image size, 53.033 μm × 53.033 μm (512 × 512 pixel).
